# Supplementary material for: Network-based drug repurposing for novel coronavirus 2019-nCoV/SARS-CoV-2
Source: Cell Discov. 2020 Mar 16;6:14. doi: 10.1038/s41421-020-0153-3 (PMC7073332; doi:10.1038/s41421-020-0153-3)
Supplement: Supplementary file 1 — Supplementary Table S1-S6 [file 41421_2020_153_MOESM1_ESM.pdf]

Supplementary Information

Network-based Drug Repurposing for Novel Coronavirus 2019-nCoV

Yadi Zhou[1,#], Yuan Hou[1,#], Jiayu Shen[1], Yin Huang[1], William Martin[1], Feixiong Cheng[1-3,*]
[1]Genomic Medicine Institute, Lerner Research Institute, Cleveland Clinic, Cleveland, OH 44195, USA
[2]Department of Molecular Medicine, Cleveland Clinic Lerner College of Medicine, Case Western Reserve University, Cleveland, OH 44195, USA
[3]Case Comprehensive Cancer Center, Case Western Reserve University School of Medicine, Cleveland, OH 44106, USA

[#]Equal contribution
*Correspondence to:
Feixiong Cheng, PhD
Lerner Research Institute
Cleveland Clinic
Tel: +1-216-444-7654; Fax: +1-216-636-0009
Email: chengf@ccf.org

Supplementary Table S1. Genome information of 15 coronaviruses used for phylogenetic analyses.
Supplementary Table S2. Protein sequence identities across 5 protein regions in 15 coronaviruses.
Supplementary Table S3. HCoV-associated host proteins with references.
Supplementary Table S4. Repurposable drugs predicted by network-based approaches.
Supplementary Table S5. Network proximity results for 2,938 drugs against pan-human coronavirus (CoV) and individual CoVs.
Supplementary Table S6. Network-predicted drug combinations for all the drug pairs from the top 16 high-confidence repurposable drugs.

Supplementary Table S1. Genome information of 15 coronaviruses used for phylogenetic analyses.

GenBank ID	Coronavirus	Identity %	Host	Location discovered
MN908947	2019-nCoV[Wuhan-Hu-1]	100	Human	China
MN938384	2019-nCoV[HKU-SZ-002a]	99.99	Human	China
MN975262	2019-nCoV[HKU-SZ-005b]	99.98	Human	China
AY390556	SARS-CoV[GZ02]	79.7	Human	China
AY485277	SARS-CoV[Sino1-11]	79.7	Human	China
AY508724	SARS-CoV[NS-1]	79.69	Human	China
AY278489	SARS-CoV[GD01]	79.65	Human	China
KT006149	MERS-CoV[ChinaGD01]	54.1	Human	China
KM027262	MERS-CoV[2014KSA_683]	54.02	Human	Kingdom of Saudi Arabia
DQ415904	HCoV-HKU1	52.41	Human	China
KJ958219	HCoV-OC43	51.08	Human	China
AC_000192	MHV	50.23	Mouse	USA
MG772808	HCoV-NL63	49.43	Human	South Korea
KY421672	IBV	48.38	Avian	China
MN306046	HCoV-229E	48.09	Human	USA

Supplementary Table S2. Protein sequence identities across 5 protein regions in 15 coronaviruses.

Coronavirus	GenBank ID	Identity %	Protein
2019-nCoV[Wuhan-Hu-1]	QHD43415.1	100	ORF1ab
2019-nCoV[HKU-SZ-002a]	QHN73794.1	100	ORF1ab
2019-nCoV[HKU-SZ-005b]	QHN73809.1	99.99	ORF1ab
SARS-CoV[GZ02]	AAS00002.1	86.51	ORF1ab
SARS-CoV[Sino1-11]	AAR23243.1	86.44	ORF1ab
SARS-CoV[GD01]	AAP51225.1	86.35	ORF1ab
SARS-CoV[NS-1]	AAR91584.1	86.35	ORF1ab
MERS-CoV[ChinaGD01]	AKJ80135.2	48	ORF1ab
MERS-CoV[2014KSA_683]	AIL23987.1	47.97	ORF1ab
HCoV-OC43	AIX09804.1	44.93	ORF1ab
HCoV-HKU1	ABD75567.1	44.69	ORF1ab
MHV	YP_209229.2	44.64	ORF1ab
HCoV-NL63	AVA26872.1	40.05	ORF1ab
HCoV-229E	QEG03783.1	39.95	ORF1ab
IBV	ATJ01091.1	27.86	ORF1ab
2019-nCoV[Wuhan-Hu-1]	QHD43416.1	100	Spike
2019-nCoV[HKU-SZ-005b]	QHN73810.1	100	Spike
2019-nCoV[HKU-SZ-002a]	QHN73795.1	100	Spike
SARS-CoV[GZ02]	AAS00003.1	77.62	Spike
SARS-CoV[GD01]	AAP51227.1	77.54	Spike
SARS-CoV[Sino1-11]	AAR23250.1	77.46	Spike
SARS-CoV[NS-1]	AAR91586.1	77.38	Spike
MHV	YP_209233.1	32.12	Spike
MERS-CoV[ChinaGD01]	AKJ80137.2	31.93	Spike
MERS-CoV[2014KSA_683]	AID55073.1	31.93	Spike
HCoV-OC43	AIX09807.1	30.84	Spike
HCoV-HKU1	ABD75561.1	30.17	Spike
HCoV-229E	QEG03785.1	27.37	Spike
IBV	ATJ01093.1	27.14	Spike
HCoV-NL63	AVA26873.1	26.89	Spike
2019-nCoV[Wuhan-Hu-1]	QHD43418.1	100	Envelope protein
2019-nCoV[HKU-SZ-002a]	QHN73797.1	100	Envelope protein
2019-nCoV[HKU-SZ-005b]	QHN73812.1	100	Envelope protein
SARS-CoV[NS-1]	AAR91589.1	96	Envelope protein
SARS-CoV[GZ02]	AAS00006.1	96	Envelope protein
SARS-CoV[GD01]	AAP51230.1	94.67	Envelope protein
SARS-CoV[Sino1-11]	AAR23247.1	94.59	Envelope protein
MERS-CoV[2014KSA_683]	AIL23994.1	36	Envelope protein
MERS-CoV[ChinaGD01]	AKJ80142.1	36	Envelope protein
HCoV-HKU1	ABD75563.1	29.33	Envelope protein
HCoV-229E	QEG03787.1	27.14	Envelope protein
MHV	YP_209236.1	21.33	Envelope protein
IBV	ATJ01096.1	20.27	Envelope protein
HCoV-OC43	AIX09809.1	18.67	Envelope protein
HCoV-NL63	AVA26875.1	17.14	Envelope protein
2019-nCoV[Wuhan-Hu-1]	QHD43419.1	100	Membrane
2019-nCoV[HKU-SZ-002a]	QHN73798.1	100	Membrane
2019-nCoV[HKU-SZ-005b]	QHN73813.1	100	Membrane
SARS-CoV[GD01]	AAP51231.1	89.59	Membrane
SARS-CoV[GZ02]	AAS00007.1	89.59	Membrane
SARS-CoV[NS-1]	AAR91590.1	89.59	Membrane

SARS-CoV[Sino1-11]	AAR23248.1	89.14	Membrane
MERS-CoV[2014KSA_683]	AIL23995.1	39.27	Membrane
MERS-CoV[ChinaGD01]	AKJ80143.1	39.27	Membrane
HCoV-OC43	AIX09810.1	38.29	Membrane
MHV	YP_209237.1	37.1	Membrane
HCoV-HKU1	ABD75564.1	35.29	Membrane
IBV	ATJ01097.1	30.56	Membrane
HCoV-229E	QEG03788.1	28.77	Membrane
HCoV-NL63	AVA26876.1	27.73	Membrane
2019-nCoV[Wuhan-Hu-1]	QHD43423.2	100	Nucleocapsid
2019-nCoV[HKU-SZ-002a]	QHN73802.1	100	Nucleocapsid
2019-nCoV[HKU-SZ-005b]	QHN73817.1	100	Nucleocapsid
SARS-CoV[GD01]	AAP51234.1	89.74	Nucleocapsid
SARS-CoV[GZ02]	AAS00011.1	89.74	Nucleocapsid
SARS-CoV[Sino1-11]	AAR23249.1	89.74	Nucleocapsid
SARS-CoV[NS-1]	AAR91593.1	89.74	Nucleocapsid
MERS-CoV[2014KSA_683]	AIL23996.1	48.85	Nucleocapsid
MERS-CoV[ChinaGD01]	AKJ80144.1	48.85	Nucleocapsid
MHV	YP_209238.1	35.31	Nucleocapsid
HCoV-HKU1	ABD75565.1	35.22	Nucleocapsid
HCoV-OC43	AIX09811.1	34.79	Nucleocapsid
IBV	ATJ01100.1	29.46	Nucleocapsid
HCoV-NL63	AVA26877.1	28.03	Nucleocapsid
HCoV-229E	QEG03789.1	27.35	Nucleocapsid

Supplementary Table S3. HCoV-associated host proteins with references.

Coronavirus	Host Protein	Host Gene ID	PubMed ID
IBV	NONO	4841	23637410
IBV	GSK3A	2931	23637410
IBV	GSK3B	2932	23637410
IBV	PABPC1	26986	23637410
IBV	PABPC4	8761	23637410
IBV	HNRNPA1	3178	23637410
IBV	HNRNPA2B1	3181	23637410
IBV	NPM1	4869	23637410
IBV	G3BP1	10146	23637410
IBV	G3BP2	9908	23637410
IBV	RPL19	6143	23637410
IBV	PARP1	142	23637410
IBV	NCL	4691	23637410
IBV	DDX1	1653	23637410
SARS	RYBP	23429	22046132
SARS	PPIA	5478	22046132
SARS	NOMO3	408050	22046132
SARS	FKBP1A	2280	22046132
SARS	PPIG	9360	22046132
SARS	MARK3	4140	22046132
SARS	PPIH	10465	22046132
SARS	RCAN3	11123	22046132
SARS	HGS	9146	22046132
SARS	BAG6	7917	22046132
SARS	DDAH2	23564	22046132
SARS	CAMLG	819	22046132
SARS	CHMP2B	25978	22046132
SARS	SNAP47	116841	22046132
SARS	MKRN2	23609	22046132
SARS	TPSAB1	7177	22046132
SARS	SERPING1	710	22046132
SARS	MKRN3	7681	22046132
SARS	PSMA2	5683	22046132
SARS	ABHD17A	81926	22046132
SARS	PFDN5	5204	22046132
SARS	MIF4GD	57409	22046132
SARS	NDUFA10	4705	22046132
SARS	VKORC1	79001	22046132
SARS	LAS1L	81887	22046132
SARS	H2AFY2	55506	22046132
SARS	RPS20	6224	22046132
SARS	CHEK2	11200	22046132
SARS	TERF1	7013	22046132
SARS	DCTN2	10540	22046132
SARS	DDX5	1655	22046132
SARS	C11orf74	119710	22046132
SARS	EIF3F	8665	22046132
SARS	EEF1A1	1915	22046132
SARS	CAV1	857	22046132
SARS	IKBKB	3551	22046132
SARS	UBE2I	7329	22046132
SARS	SGTA	6449	22046132
SARS	ATP6V1G1	9550	22046132

SARS	BTF3	689	22046132
SARS	ATF5	22809	22046132
SARS	ND4L	4539	22046132
SARS	COX2	4513	22046132
SARS	HNRNPA1	3178	22046132
SARS	ACE2	59272	22046132
SARS	CLEC4G	339390	22046132
SARS	CD209	30835	22046132
SARS	CLEC4M	10332	22046132
SARS	IRF3	3661	22046132
SARS	KPNA2	3838	22046132
SARS	SFTPD	6441	22046132
SARS	PPIA	5478	22046132
SARS	BCL2L1	598	22046132
SARS	BCL2L2	599	22046132
SARS	MCL1	4170	22046132
SARS	BCL2A1	597	22046132
SARS	BCL2	596	22046132
MERS	SKP2	6502	31852899
MERS	KPNA4	3840	29370303
MERS	PRKRA	8575	24522921
MERS	CD9	928	28759649
MERS	TMPRSS2	7113	28759649
SARS	IKBKB	3551	17705188
HCoV-229E	ANPEP	290	28643204
HCoV-NL63	ACE2	59272	28643204
IBV	ZCRB1	85437	28643204
MERS	DPP4	1803	28643204
MHV	HNRNPA1	3178	28643204
MHV	SYNCRIP	10492	28643204
MHV	PTBP1	5725	28643204
MHV	CEACAM1	634	28643204
SARS	ZCRB1	85437	28643204
SARS	ACE2	59272	28643204
IBV	ANXA2	302	28643204
MHV	HNRNPA2B1	3181	28643204
MHV	HNRNPA3	220988	28643204
MHV	ACO2	50	28643204
MHV	DNAJB1	3337	28643204
MHV	HSPD1	3329	28643204
MHV	HSPA9	3313	28643204
MHV	COPB2	9276	30632963
MHV	RPL13A	23521	30632963
MHV	EIF3E	3646	30632963
MHV	EIF3I	8668	30632963
MHV	NMT1	4836	30632963
MHV	CHMP4B	128866	30632963
MHV	EIF3F	8665	30632963
MHV	GBF1	8729	30632963
MHV	RRM2	6241	30632963
MHV	KIF11	3832	30632963
MHV	PSMD1	5707	30632963
MHV	SRP54	6729	30632963
MHV	NUDCD1	84955	30632963
MHV	NACA	4666	30632963
MHV	SNX9	51429	30632963

MHV	BTF3	689	30632963
MHV	SCFD1	23256	30632963
MHV	PSMC2	5701	30632963
MHV	TFEB	7942	30632963
MHV	TWF2	11344	30632963
MHV	YKT6	10652	30632963
MHV	KPNB1	3837	30632963
MHV	STX5	6811	30632963
MHV	STAT5A	6776	30632963
MHV	RSL24D1	51187	30632963
MHV	ACBD5	91452	30632963
SARS	PHB	5245	STRING Viruses
SARS	JUN	3725	STRING Viruses
SARS	STAT3	6774	STRING Viruses
SARS	PPP1CA	5499	STRING Viruses
SARS	SPECC1	92521	STRING Viruses
SARS	FGL2	10875	STRING Viruses
SARS	XPO1	7514	STRING Viruses
HCoV-229E	TGFB1	7040	STRING Viruses
HCoV-229E	FGL2	10875	STRING Viruses
MHV	SMAD3	4088	STRING Viruses

Supplementary Table S4. Repurposable drugs predicted by network-based approaches.

DB ID	Name	Z-score	P-value	GSEA score	Literature evidence
DB01029	Irbesartan	-5.98	0.001	NA	25929767, 26086883, 24717262
DB02152	K-252a	-4.22	0.006	NA	
DB08813	Nadroparin	-3.46	0.005	NA	
DB00890	Dienestrol	-3.30	0.003	0	
DB00539	Toremifene	-3.23	0.011	NA	27362232, 29566060, 24841273, 24841269
DB03963	S-(Dimethylarsenic)Cysteine	-3.00	0	NA	
DB08005	4-{[5-chloro-4-(1H-indol-3-yl)pyrimidin-2-yl]amino}-N-ethylpiperidine-1-carboxamide	-2.81	0.013	NA	
DB04930	Permethrin	-2.67	0.011	NA	
DB01357	Mestranol	-2.64	0.01	2	
DB01744	Camphor	-2.64	0.007	NA	27823881,30165308
DB00286	Conjugated estrogens	-2.60	0.007	NA	
DB02187	Equilin	-2.52	0.012	3	27169275
DB00563	Methotrexate	-2.46	0.012	2	
DB00544	Fluorouracil	-2.44	0.009	NA	
DB07931	Hexestrol	-2.44	0.008	0	27169275
DB00244	Mesalazine	-2.44	0.005	3	25914477
DB01033	Mercaptopurine	-2.44	0.02	1	18313035, 19374142, 25542975
DB00715	Paroxetine	-2.42	0.014	2	29272110
DB00852	Pseudoephedrine	-2.42	0.014	NA	
DB01769	3-sulfino-L-alanine	-2.41	0.015	NA	
DB00614	Furazolidone	-2.39	0.01	2	
DB04824	Phenolphthalein	-2.38	0.013	NA	
DB00877	Sirolimus	-2.35	0.006	3	23135723, 26276683, 29143192, 24105455
DB00570	Vinblastine	-2.28	0.014	2	
DB00812	Phenylbutazone	-2.25	0.02	NA	
DB04575	Quinestrol	-2.23	0.015	NA	
DB04938	Ospemifene	-2.23	0.015	NA	
DB09092	Xanthinol	-2.23	0.015	NA	
DB09317	Synthetic Conjugated Estrogens, A	-2.23	0.015	NA	
DB09318	Synthetic Conjugated Estrogens, B	-2.23	0.015	NA	
DB01183	Naloxone	-2.22	0.021	1	
DB00242	Cladribine	-2.21	0.02	NA	
DB02615	Compound 19	-2.20	0.016	NA	
DB01275	Hydralazine	-2.20	0.013	0	
DB08231	Myristic acid	-2.19	0.018	NA	
DB00170	Menadione	-2.17	0.012	1	
DB01248	Docetaxel	-2.17	0.014	NA	
DB05465	Tandutinib	-2.11	0.02	NA	
DB08896	Regorafenib	-2.06	0.017	NA	
DB08888	Ocriplasmin	-2.04	0.032	NA	
DB00361	Vinorelbine	-2.03	0.02	NA	
DB07502	4-bromo-6-(6-hydroxy-1,2-benzisoxazol-3-yl)benzene-1,3-diol	-2.02	0.019	NA	
DB06871	17-METHYL-17-ALPHA-DIHYDROEQUILENIN	-2.02	0.021	NA	
DB06898	4-(2-amino-1-methyl-1H-imidazo[4,5-b]pyridin-6-yl)phenol	-2.02	0.021	NA	
DB07086	4-[(1S,2S,5S)-5-(HYDROXYMETHYL)-8-METHYL-3-OXABICYCLO[3.3.1]NON-7-EN-2-YL]PHENOL	-2.02	0.021	NA	
DB07087	4-[(1S,2S,5S,9R)-5-(HYDROXYMETHYL)-8,9-DIMETHYL-3-OXABICYCLO[3.3.1]NON-7-EN-2-YL]PHENOL	-2.02	0.021	NA	
DB07195	4-[(1S,2S,5S)-5-(HYDROXYMETHYL)-6,8,9-TRIMETHYL-3-OXABICYCLO[3.3.1]NON-7-EN-2-YL]PHENOL	-2.02	0.021	NA	
DB07678	(9ALPHA,13BETA,17BETA)-2-[(1Z)-BUT-1-EN-1-YL]ESTRA-1,3,5(10)-TRIENE-3,17-DIOL	-2.02	0.021	NA	
DB07707	(9BETA,11ALPHA,13ALPHA,14BETA,17ALPHA)-11-(METHOXYMETHYL)ESTRA-1(10),2,4-TRIENE-3,17-DIOL	-2.02	0.021	NA	
DB07708	3-CHLORO-2-(4-HYDROXYPHENYL)-2H-INDAZOL-5-OL	-2.02	0.021	NA	
DB07712	3-ETHYL-2-(4-HYDROXYPHENYL)-2H-INDAZOL-5-OL	-2.02	0.021	NA	
DB07932	dimethyl (1R,4S)-5,6-bis(4-hydroxyphenyl)-7-oxabicyclo[2.2.1]hepta-2,5-diene-2,3-dicarboxylate	-2.02	0.021	NA	
DB08048	4-(6-HYDROXY-1H-INDAZOL-3-YL)BENZENE-1,3-DIOL	-2.02	0.021	NA	

DB08320	DIETHYL (1R,2S,3R,4S)-5,6-BIS(4-HYDROXYPHENYL)-7-OXABICYCLO[2.2.1]HEPT-5-ENE-2,3-DICARBOXYLATE	-2.02	0.021	NA	
DB08398	2-Amino-1-methyl-6-phenylimidazo(4,5-b)pyridine	-2.02	0.021	NA	
DB08595	4-[(1S,2R,5S)-4,4,8-TRIMETHYL-3-OXABICYCLO[3.3.1]NON-7-EN-2-YL]PHENOL	-2.02	0.021	NA	
DB03909	Adenosine-5'-[Beta, Gamma-Methylene]Triphosphate	-1.98	0.016	NA	
DB04967	Lucanthone	-1.97	0.019	NA	
DB01136	Carvedilol	-1.94	0.023	NA	12535832
DB02513	Thymol	-1.93	0.001	NA	28886313
DB01394	Colchicine	-1.92	0.027	0	28795759
DB00431	Lindane	-1.92	0.018	NA	
DB00098	Antithymocyte immunoglobulin (rabbit)	-1.91	0.016	NA	
DB00321	Amitriptyline	-1.89	0.03	2	
DB00668	Epinephrine	-1.88	0.031	NA	
DB00970	Dactinomycin	-1.88	0.027	NA	1335030
DB07886	Hydrocortisone	-1.88	0.013	NA	16891588
DB00294	Etonogestrel	-1.82	0.023	NA	
DB00304	Desogestrel	-1.82	0.023	NA	
DB02538	N-[4-(2-Methylimidazo[1,2-a]Pyridin-3-Yl)-2-Pyrimidinyl]Acetamide	-1.77	0.027	NA	
DB04006	[2-Amino-6-(2,6-Difluoro-Benzoyl)-Imidazo[1,2-a]Pyridin-3-Yl]-Phenyl-Methanone	-1.77	0.027	NA	
DB04518	3-[4-(2,4-Dimethyl-Thiazol-5-Yl)-Pyrimidin-2-Ylamino]-Phenol	-1.77	0.027	NA	
DB07790	N-(2-METHOXYETHYL)-4-({4-[2-METHYL-1-(1-METHYLETHYL)-1H-IMIDAZOL-5-YL]PYRIMIDIN-2-YL}AMINO)BENZENESULFONAMIDE	-1.77	0.027	NA	
DB08285	(2R)-2-{[4-(benzylamino)-8-(1-methylethyl)pyrazolo[1,5-a][1,3,5]triazin-2-yl]amino}butan-1-ol	-1.77	0.027	NA	
DB00714	Apomorphine	-1.74	0.033	3	
DB01420	Testosterone propionate	-1.74	0.027	NA	
DB01065	Melatonin	-1.72	0.029	2	25262626, 20070490
DB06710	Methyltestosterone	-1.71	0.024	NA	
DB00108	Natalizumab	-1.69	0.034	NA	
DB00947	Fulvestrant	-1.67	0.031	3	
DB08900	Teduglutide	-1.66	0.004	NA	
DB00338	Omeprazole	-1.65	0.031	0	
DB00367	Levonorgestrel	-1.64	0.025	2	
DB06287	Temsirolimus	-1.62	0.043	NA	
DB01103	Quinacrine	-1.62	0.044	1	23301007, 31307979, 27890675
DB06782	Dimercaprol	-1.60	0.041	NA	
DB00624	Testosterone	-1.60	0.035	0	29557189, 30522976
DB06822	Tinzaparin	-1.60	0.043	NA	
DB00700	Eplerenone	-1.59	0.028	NA	19213804
DB00162	Vitamin A	-1.58	0.045	NA	8379807, 7751694
DB09148	Florbetaben (18F)	-1.58	0.034	NA	
DB09149	Florbetapir (18F)	-1.58	0.034	NA	
DB09151	Flutemetamol (18F)	-1.58	0.034	NA	
DB02091	4-(2,4-Dimethyl-Thiazol-5-Yl)-Pyrimidin-2-Ylamine	-1.57	0.044	NA	
DB02915	4-(2,4-Dimethyl-1,3-thiazol-5-yl)-N-[4-(trifluoromethyl)phenyl]-2-pyrimidinamine	-1.57	0.044	NA	
DB06844	4-[(7-OXO-7H-THIAZOLO[5,4-E]INDOL-8-YLMETHYL)-AMINO]-N-PYRIDIN-2-YL-BENZENESULFONAMIDE	-1.57	0.044	NA	
DB06948	2-ANILINO-6-CYCLOHEXYLMETHOXYPURINE	-1.57	0.044	NA	
DB07137	(2S)-N-[(3Z)-5-CYCLOPROPYL-3H-PYRAZOL-3-YLIDENE]-2-[4-(2-OXOIMIDAZOLIDIN-1-YL)PHENYL]PROPANAMIDE	-1.57	0.044	NA	
DB07203	6-CYCLOHEXYLMETHOXY-2-(3'-CHLOROANILINO) PURINE	-1.57	0.044	NA	
DB07539	4-{5-[(Z)-(2-IMINO-4-OXO-1,3-THIAZOLIDIN-5-YLIDENE)METHYL]FURAN-2-YL}BENZOIC ACID	-1.57	0.044	NA	
DB07852	1-(3,5-DICHLOROPHENYL)-5-METHYL-1H-1,2,4-TRIAZOLE-3-CARBOXYLIC ACID	-1.57	0.044	NA	
DB08178	4-(4-methoxy-1H-pyrrolo[2,3-b]pyridin-3-yl)pyrimidin-2-amine	-1.57	0.044	NA	
DB08182	4-(4-propoxy-1H-pyrrolo[2,3-b]pyridin-3-yl)pyrimidin-2-amine	-1.57	0.044	NA	

DB08218	HYDROXY(OXO)(3-{[(2Z)-4-[3-(1H-1,2,4-TRIAZOL-1-YLMETHYL)PHENYL]PYRIMIDIN-2(5H)-YLIDENE]AMINO}PHENYL)AMMONIUM	-1.57	0.044	NA	
DB08219	4-METHYL-5-{(2E)-2-[(4-MORPHOLIN-4-YLPHENYL)IMINO]-2,5-DIHYDROPYRIMIDIN-4-YL}-1,3-THIAZOL-2-AMINE	-1.57	0.044	NA	
DB08233	6-CYCLOHEXYLMETHYLOXY-2-(4'-HYDROXYANILINO)PURINE	-1.57	0.044	NA	
DB08241	4-(6-CYCLOHEXYLMETHOXY-9H-PURIN-2-YLAMINO)--BENZAMIDE	-1.57	0.044	NA	
DB08248	3-(6-CYCLOHEXYLMETHOXY-9H-PURIN-2-YLAMINO)-BENZENESULFONAMIDE	-1.57	0.044	NA	
DB08527	1-[4-(AMINOSULFONYL)PHENYL]-1,6-DIHYDROPYRAZOLO[3,4-E]INDAZOLE-3-CARBOXAMIDE	-1.57	0.044	NA	
DB01370	Aluminium	-1.57	0.042	NA	
DB02197	4-[(4-Imidazo[1,2-a]Pyridin-3-Ylpyrimidin-2-Yl)Amino]Benzenesulfonamide	-1.56	0.042	NA	
DB00269	Chlorotrianisene	-1.55	0.047	NA	
DB01878	Benzophenone	-1.55	0.047	NA	
DB04020	4-(2-{[4-{[3-(4-Chlorophenyl)Propyl]Sulfanyl}-6-(1-Piperazinyl)-1,3,5-Triazin-2-Yl]Amino}Ethyl)Phenol	-1.55	0.047	NA	
DB05414	Pipendoxifene	-1.55	0.047	NA	
DB06401	Bazedoxifene	-1.55	0.047	NA	
DB06937	4-(6-HYDROXY-BENZO[D]ISOXAZOL-3-YL)BENZENE-1,3-DIOL	-1.55	0.047	NA	
DB07036	(3aS,4R,9bR)-2,2-difluoro-4-(4-hydroxyphenyl)-6-(methoxymethyl)-1,2,3,3a,4,9b-hexahydrocyclopenta[c]chromen-8-ol	-1.55	0.047	NA	
DB07567	(2R,3R,4S)-3-(4-HYDROXYPHENYL)-4-METHYL-2-[4-(2-PYRROLIDIN-1-YLETHOXY)PHENYL]CHROMAN-6-OL	-1.55	0.047	NA	
DB07638	(3AS,4R,9BR)-2,2-DIFLUORO-4-(4-HYDROXYPHENYL)-1,2,3,3A,4,9B-HEXAHYDROCYCLOPENTA[C]CHROMEN-8-OL	-1.55	0.047	NA	
DB07757	(9aS)-4-bromo-9a-butyl-7-hydroxy-1,2,9,9a-tetrahydro-3H-fluoren-3-one	-1.55	0.047	NA	
DB07933	Erteberel	-1.55	0.047	NA	
DB07991	N-[(1R)-3-(4-HYDROXYPHENYL)-1-METHYLPROPYL]-2-(2-PHENYL-1H-INDOL-3-YL)ACETAMIDE	-1.55	0.047	NA	
DB08020	(3AS,4R,9BR)-4-(4-HYDROXYPHENYL)-6-(METHOXYMETHYL)-1,2,3,3A,4,9B-HEXAHYDROCYCLOPENTA[C]CHROMEN-8-OL	-1.55	0.047	NA	
DB08737	(3AS,4R,9BR)-4-(4-HYDROXYPHENYL)-1,2,3,3A,4,9B-HEXAHYDROCYCLOPENTA[C]CHROMEN-9-OL	-1.55	0.047	NA	
DB09535	Octocrylene	-1.55	0.047	NA	
DB07715	Emodin	-1.54	0.035	NA	21050882, 16940925, 21356245, 16730806, 24071990
DB00851	Dacarbazine	-1.53	0.036	0	
DB06412	Oxymetholone	-1.53	0.041	NA	12815555
DB00396	Progesterone	-1.53	0.034	1	
DB07471	5-[5,6-BIS(METHYLOXY)-1H-BENZIMIDAZOL-1-YL]-3-{[1-(2-CHLOROPHENYL)ETHYL]OXY}-2-THIOPHENECARBOXAMIDE	-1.52	0.036	NA	
DB02901	Stanolone	-1.52	0.043	NA	
DB03801	Lysine Nz-Carboxylic Acid	-1.51	0.04	NA	
DB08683	REL-(9R,12S)-9,10,11,12-TETRAHYDRO-9,12-EPOXY-1H-DIINDOLO[1,2,3-FG:3',2',1'-KL]PYRROLO[3,4-I][1,6]BENZODIAZOCINE-1,3(2H)-DIONE	-1.51	0.045	NA	
DB09257	Gimeracil	-1.50	0.009	NA	

Supplementary Table S5. Network proximity results for 2,938 drugs against pan-human coronavirus (CoV) and individual CoVs.

DrugBank ID	Name	Z-score					P value				
		Pan	SARS	MERS	IBV	MHV	Pan	SARS	MERS	IBV	MHV
DB01029	Irbesartan	-5.984	-3.788	-1.329	-4.894	-4.102	0.001	0.003	0.041	0.001	0.007
DB02152	K-252a	-4.223	-2.34	-0.55	-3.611	-5.469	0.006	0.017	0.141	0.003	0.003
DB08813	Nadroparin	-3.462	-2.652	-1.585	-2.633	-2.976	0.005	0.005	0.041	0.007	0.006
DB00890	Dienestrol	-3.298	-1.215	-2.438	-3.191	-3.256	0.003	0.06	0.011	0.002	0.005
DB00539	Toremifene	-3.226	-2.137	0.018	-2.164	-3.939	0.011	0.014	0.396	0.018	0.012
DB03963	S-(Dimethylarsenic)Cysteine	-3.001	-1.553	0.034	0.227	-3.258	0	0.056	0.148	0.415	0.001
DB08005	4-{[5-chloro-4-(1H-indol-3-yl)pyrimidin-2-yl]amino}-N-ethylpiperidine-1-carboxamide	-2.812	-1.428	-1.025	-3.059	-3.381	0.013	0.042	0.092	0.002	0.008
DB04930	Permethrin	-2.671	-1.119	-1.013	-3.732	-2.511	0.011	0.085	0.074	0.004	0.012
DB01357	Mestranol	-2.644	-1.314	-0.651	-4.268	-2.845	0.01	0.071	0.196	0.003	0.005
DB01744	Camphor	-2.636	-1.67	-1.099	-5.031	-2.888	0.007	0.04	0.093	0.002	0.008
DB00286	Conjugated estrogens	-2.604	-1.64	0.302	-4.199	-2.702	0.007	0.045	0.345	0.003	0.01
DB02187	Equilin	-2.524	-2.076	-0.533	-4.281	-3.653	0.012	0.028	0.22	0.001	0.003
DB00563	Methotrexate	-2.457	-1.743	-2.286	-1.716	-1.329	0.012	0.035	0.007	0.043	0.076
DB00544	Fluorouracil	-2.443	-1.598	-2.495	-3.177	-2.001	0.009	0.049	0.007	0.006	0.028
DB07931	Hexestrol	-2.443	-1.291	-0.333	-4.18	-3.1	0.008	0.067	0.288	0.002	0.006
DB00244	Mesalazine	-2.438	-2.496	-0.603	-0.009	-1.066	0.005	0.003	0.163	0.443	0.106
DB01033	Mercaptopurine	-2.437	-0.753	-2.796	-2.549	-0.47	0.02	0.164	0.001	0.012	0.248
DB00715	Paroxetine	-2.419	-1.315	-1.106	-1.052	-1.328	0.014	0.065	0.084	0.111	0.069
DB00852	Pseudoephedrine	-2.415	-1.827	-0.563	-1.05	-1.159	0.014	0.033	0.234	0.109	0.101
DB01769	3-sulfino-L-alanine	-2.41	-1.399	-0.142	-2.765	-1.798	0.015	0.05	0.359	0.003	0.027
DB00614	Furazolidone	-2.389	-1.677	-2.532	-3.48	-2.115	0.01	0.029	0.008	0.011	0.015
DB04824	Phenolphthalein	-2.375	-1.552	-0.094	-3.13	-3.168	0.013	0.05	0.314	0.006	0.003
DB00877	Sirolimus	-2.354	-0.9	1.807	-1.041	-2.253	0.006	0.158	0.959	0.105	0.015
DB00570	Vinblastine	-2.281	-1.63	-0.296	-2.935	-1.595	0.014	0.032	0.297	0.004	0.04
DB00812	Phenylbutazone	-2.25	-0.596	2.416	-3.053	-2.945	0.02	0.174	0.99	0.004	0.005
DB04575	Quinestrol	-2.226	-1.519	-0.325	-4.306	-2.603	0.015	0.04	0.297	0.01	0.007
DB04938	Ospemifene	-2.226	-1.519	-0.325	-4.306	-2.603	0.015	0.04	0.297	0.01	0.007
DB09092	Xanthinol	-2.226	-1.519	-0.325	-4.306	-2.603	0.015	0.04	0.297	0.01	0.007
DB09317	Synthetic Conjugated Estrogens, A	-2.226	-1.519	-0.325	-4.306	-2.603	0.015	0.04	0.297	0.01	0.007
DB09318	Synthetic Conjugated Estrogens, B	-2.226	-1.519	-0.325	-4.306	-2.603	0.015	0.04	0.297	0.01	0.007
DB01183	Naloxone	-2.22	-0.624	-0.057	-3.147	-2.11	0.021	0.203	0.354	0.004	0.011
DB00242	Cladribine	-2.214	-0.471	-2.311	-2.873	-1.398	0.02	0.237	0.015	0.005	0.064
DB02615	Compound 19	-2.204	-1.202	-0.866	-4.336	-2.812	0.016	0.071	0.101	0	0.005
DB01275	Hydralazine	-2.203	-1.334	-1.919	-1.127	-0.4	0.013	0.071	0.021	0.094	0.264
DB08231	Myristic acid	-2.19	-2.145	1.411	0.502	-2.117	0.018	0.019	0.883	0.653	0.028
DB00170	Menadione	-2.174	-0.526	-1.844	-0.459	-0.62	0.012	0.222	0.025	0.239	0.197
DB01248	Docetaxel	-2.17	-1.66	0.027	0.13	-2.159	0.014	0.02	0.389	0.482	0.014
DB05465	Tandutinib	-2.11	-0.494	-0.661	-1.587	-2.228	0.02	0.231	0.214	0.041	0.015
DB08896	Regorafenib	-2.059	-0.73	0.47	-0.396	-3.275	0.017	0.154	0.582	0.297	0.008
DB08888	Ocriplasmin	-2.038	-0.885	0.652	-2.752	-2.986	0.032	0.12	0.43	0.008	0.005
DB00361	Vinorelbine	-2.028	-0.664	-1.699	-1.879	-1.45	0.02	0.132	0.031	0.025	0.052
DB07502	4-bromo-6-(6-hydroxy-1,2-benzisoxazol-3-yl)benzene-1,3-diol	-2.02	-1.373	-0.983	-3.697	-1.956	0.019	0.054	0.117	0.003	0.017
DB06871	17-METHYL-17-ALPHA-DIHYDROEQUILENIN	-2.016	-1.281	-0.171	-4.07	-2.662	0.021	0.057	0.345	0	0.008
DB06898	4-(2-amino-1-methyl-1H-imidazo[4,5-b]pyridin-6-yl)phenol	-2.016	-1.281	-0.171	-4.07	-2.662	0.021	0.057	0.345	0	0.008

DB07086	4-[(1S,2S,5S)-5-(HYDROXYMETHYL)-8-METHYL-3-OXABICYCLO[3.3.1]NON-7-EN-2-YL]PHENOL	-2.016	-1.281	-0.171	-4.07	-2.662	0.021	0.057	0.345	0	0.008
DB07087	4-[(1S,2S,5S,9R)-5-(HYDROXYMETHYL)-8,9-DIMETHYL-3-OXABICYCLO[3.3.1]NON-7-EN-2-YL]PHENOL	-2.016	-1.281	-0.171	-4.07	-2.662	0.021	0.057	0.345	0	0.008
DB07195	4-[(1S,2S,5S)-5-(HYDROXYMETHYL)-6,8,9-TRIMETHYL-3-OXABICYCLO[3.3.1]NON-7-EN-2-YL]PHENOL	-2.016	-1.281	-0.171	-4.07	-2.662	0.021	0.057	0.345	0	0.008
DB07678	(9ALPHA,13BETA,17BETA)-2-[(1Z)-BUT-1-EN-1-YL]ESTRA-1,3,5(10)-TRIENE-3,17-DIOL	-2.016	-1.281	-0.171	-4.07	-2.662	0.021	0.057	0.345	0	0.008
DB07707	(9BETA,11ALPHA,13ALPHA,14BETA,17ALPHA)-11-(METHOXYMETHYL)ESTRA-1(10),2,4-TRIENE-3,17-DIOL	-2.016	-1.281	-0.171	-4.07	-2.662	0.021	0.057	0.345	0	0.008
DB07708	3-CHLORO-2-(4-HYDROXYPHENYL)-2H-INDAZOL-5-OL	-2.016	-1.281	-0.171	-4.07	-2.662	0.021	0.057	0.345	0	0.008
DB07712	3-ETHYL-2-(4-HYDROXYPHENYL)-2H-INDAZOL-5-OL	-2.016	-1.281	-0.171	-4.07	-2.662	0.021	0.057	0.345	0	0.008
DB07932	dimethyl (1R,4S)-5,6-bis(4-hydroxyphenyl)-7-oxabicyclo[2.2.1]hepta-2,5-diene-2,3-dicarboxylate	-2.016	-1.281	-0.171	-4.07	-2.662	0.021	0.057	0.345	0	0.008
DB08048	4-(6-HYDROXY-1H-INDAZOL-3-YL)BENZENE-1,3-DIOL	-2.016	-1.281	-0.171	-4.07	-2.662	0.021	0.057	0.345	0	0.008
DB08320	DIETHYL (1R,2S,3R,4S)-5,6-BIS(4-HYDROXYPHENYL)-7-OXABICYCLO[2.2.1]HEPT-5-ENE-2,3-DICARBOXYLATE	-2.016	-1.281	-0.171	-4.07	-2.662	0.021	0.057	0.345	0	0.008
DB08398	2-Amino-1-methyl-6-phenylimidazo(4,5-b)pyridine	-2.016	-1.281	-0.171	-4.07	-2.662	0.021	0.057	0.345	0	0.008
DB08595	4-[(1S,2R,5S)-4,4,8-TRIMETHYL-3-OXABICYCLO[3.3.1]NON-7-EN-2-YL]PHENOL	-2.016	-1.281	-0.171	-4.07	-2.662	0.021	0.057	0.345	0	0.008
DB03909	Adenosine-5'-[Beta, Gamma-Methylene]Triphosphate	-1.977	-1.998	-0.681	0.984	-1.045	0.016	0.013	0.104	0.706	0.092
DB04967	Lucanthone	-1.967	-0.462	1.272	-3.566	-1.348	0.019	0.26	0.882	0	0.063
DB01136	Carvedilol	-1.944	-1.16	0.338	-0.943	-1.153	0.023	0.097	0.548	0.109	0.074
DB02513	Thymol	-1.926	-1.821	-1.283	-1.915	-1.948	0.001	0.011	0.128	0.003	0.001
DB01394	Colchicine	-1.919	-1.583	-1.525	-2.157	-1.53	0.027	0.034	0.043	0.016	0.05
DB00431	Lindane	-1.917	-1.553	-0.284	-3.128	-2.083	0.018	0.056	0.27	0.002	0.017
DB00098	Antithymocyte immunoglobulin (rabbit)	-1.911	1.204	-3.221	-2.34	-1.439	0.016	0.851	0.002	0.012	0.053
DB00321	Amitriptyline	-1.89	-0.179	1.483	1.131	-2.334	0.03	0.336	0.918	0.87	0.022
DB00668	Epinephrine	-1.88	-1.024	-0.093	-0.995	-1.616	0.031	0.142	0.529	0.132	0.055
DB00970	Dactinomycin	-1.879	-1.529	0.651	-2.826	-0.699	0.027	0.035	0.444	0.003	0.155
DB07886	Hydrocortisone	-1.876	-1.789	0.208	-0.722	-2.04	0.013	0.01	0.189	0.165	0.01
DB00294	Etonogestrel	-1.821	-0.87	-0.77	-3.368	-2.358	0.023	0.129	0.167	0.004	0.013
DB00304	Desogestrel	-1.821	-0.87	-0.77	-3.368	-2.358	0.023	0.129	0.167	0.004	0.013
DB02538	N-[4-(2-Methylimidazo[1,2-a]Pyridin-3-Yl)-2-Pyrimidinyl]Acetamide	-1.769	-1.216	0.024	-2.264	-2.202	0.027	0.057	0.427	0.011	0.016
DB04006	[2-Amino-6-(2,6-Difluoro-Benzoyl)-Imidazo[1,2-a]Pyridin-3-Yl]-Phenyl-Methanone	-1.769	-1.216	0.024	-2.264	-2.202	0.027	0.057	0.427	0.011	0.016
DB04518	3-[4-(2,4-Dimethyl-Thiazol-5-Yl)-Pyrimidin-2-Ylamino]-Phenol	-1.769	-1.216	0.024	-2.264	-2.202	0.027	0.057	0.427	0.011	0.016

DB07790	N-(2-METHOXYETHYL)-4-({4-[2-METHYL-1-(1-METHYLETHYL)-1H-IMIDAZOL-5-YL]PYRIMIDIN-2-YL}AMINO)BENZENESULFONAMIDE	-1.769	-1.216	0.024	-2.264	-2.202	0.027	0.057	0.427	0.011	0.016
DB08285	(2R)-2-{[4-(benzylamino)-8-(1-methylethyl)pyrazolo[1,5-a][1,3,5]triazin-2-yl]amino}butan-1-ol	-1.766	-0.75	-1.268	-2.488	-1.104	0.027	0.159	0.061	0.006	0.085
DB00714	Apomorphine	-1.737	-1.352	0.559	0.35	-0.632	0.033	0.065	0.647	0.59	0.202
DB01420	Testosterone propionate	-1.736	-0.619	-0.939	-3.533	-1.653	0.027	0.178	0.123	0.004	0.035
DB01065	Melatonin	-1.72	-1.423	-0.002	-1.182	-1.422	0.029	0.056	0.4	0.103	0.058
DB06710	Methyltestosterone	-1.706	-0.877	-1.222	-3.214	-1.943	0.024	0.119	0.07	0.002	0.02
DB00108	Natalizumab	-1.692	-0.45	0.736	-3.471	-1.654	0.034	0.216	0.653	0	0.044
DB00947	Fulvestrant	-1.671	-0.497	-0.991	-2.337	-2.578	0.031	0.22	0.105	0.01	0.009
DB08900	Teduglutide	-1.656	-1.58	-1.436	-1.742	-1.847	0.004	0.044	0.085	0.007	0.002
DB00338	Omeprazole	-1.652	-0.599	1.045	-0.394	-0.525	0.031	0.23	0.767	0.279	0.26
DB00367	Levonorgestrel	-1.635	-0.486	-1.269	-3.168	-1.901	0.025	0.191	0.068	0.003	0.021
DB06287	Temsirolimus	-1.623	-0.383	0.159	-0.806	-1.277	0.043	0.314	0.202	0.156	0.069
DB01103	Quinacrine	-1.62	-0.526	-0.089	-0.723	-0.726	0.044	0.241	0.378	0.173	0.188
DB06782	Dimercaprol	-1.603	-0.976	-1.473	-0.787	-1.403	0.041	0.09	0.056	0.161	0.035
DB00624	Testosterone	-1.602	-0.875	-1.498	-2.498	-1.825	0.035	0.128	0.043	0.01	0.031
DB06822	Tinzaparin	-1.602	0.092	-1.135	-3.614	-2.424	0.043	0.449	0.058	0.004	0.011
DB00700	Eplerenone	-1.588	-1.176	0.063	-0.933	-1.76	0.028	0.08	0.179	0.118	0.015
DB01427	Amrinone	-1.583	-1.261	-1.288	-0.075	-0.859	0.05	0.095	0.073	0.444	0.162
DB00162	Vitamin A	-1.58	-0.65	-0.384	-1.741	-1.297	0.045	0.163	0.27	0.033	0.072
DB09148	Florbetaben (18F)	-1.578	-1.519	-0.325	-1.828	-1.872	0.034	0.04	0.297	0.024	0.02
DB09149	Florbetapir (18F)	-1.578	-1.519	-0.325	-1.828	-1.872	0.034	0.04	0.297	0.024	0.02
DB09151	Flutemetamol (18F)	-1.578	-1.519	-0.325	-1.828	-1.872	0.034	0.04	0.297	0.024	0.02
DB02091	4-(2,4-Dimethyl-Thiazol-5-Yl)-Pyrimidin-2-Ylamine	-1.57	-0.882	-1.694	-2.361	-2.01	0.044	0.119	0.028	0.009	0.02
DB02915	4-(2,4-Dimethyl-1,3-thiazol-5-yl)-N-[4-(trifluoromethyl)phenyl]-2-pyrimidinamine	-1.57	-0.882	-1.694	-2.361	-2.01	0.044	0.119	0.028	0.009	0.02
DB06844	4-[(7-OXO-7H-THIAZOLO[5,4-E]INDOL-8-YLMETHYL)-AMINO]-N-PYRIDIN-2-YL-BENZENESULFONAMIDE	-1.57	-0.882	-1.694	-2.361	-2.01	0.044	0.119	0.028	0.009	0.02
DB06948	2-ANILINO-6-CYCLOHEXYLMETHOXYPURINE	-1.57	-0.882	-1.694	-2.361	-2.01	0.044	0.119	0.028	0.009	0.02
DB07137	(2S)-N-[(3Z)-5-CYCLOPROPYL-3H-PYRAZOL-3-YLIDENE]-2-[4-(2-OXOIMIDAZOLIDIN-1-YL)PHENYL]PROPANAMIDE	-1.57	-0.882	-1.694	-2.361	-2.01	0.044	0.119	0.028	0.009	0.02
DB07203	6-CYCLOHEXYLMETHOXY-2-(3'-CHLOROANILINO) PURINE	-1.57	-0.882	-1.694	-2.361	-2.01	0.044	0.119	0.028	0.009	0.02
DB07539	4-{5-[(Z)-(2-IMINO-4-OXO-1,3-THIAZOLIDIN-5-YLIDENE)METHYL]FURAN-2-YL}BENZOIC ACID	-1.57	-0.882	-1.694	-2.361	-2.01	0.044	0.119	0.028	0.009	0.02
DB07852	1-(3,5-DICHLOROPHENYL)-5-METHYL-1H-1,2,4-TRIAZOLE-3-CARBOXYLIC ACID	-1.57	-0.882	-1.694	-2.361	-2.01	0.044	0.119	0.028	0.009	0.02
DB08178	4-(4-methoxy-1H-pyrrolo[2,3-b]pyridin-3-yl)pyrimidin-2-amine	-1.57	-0.882	-1.694	-2.361	-2.01	0.044	0.119	0.028	0.009	0.02
DB08182	4-(4-propoxy-1H-pyrrolo[2,3-b]pyridin-3-yl)pyrimidin-2-amine	-1.57	-0.882	-1.694	-2.361	-2.01	0.044	0.119	0.028	0.009	0.02

DB08218	HYDROXY(OXO)(3-{[(2Z)-4-[3-(1H-1,2,4-TRIAZOL-1-YLMETHYL)PHENYL]PYRIMIDIN-2(5H)-YLIDENE]AMINO}PHENYL)AMMONIUM	-1.57	-0.882	-1.694	-2.361	-2.01	0.044	0.119	0.028	0.009	0.02
DB08219	4-METHYL-5-{(2E)-2-[(4-MORPHOLIN-4-YLPHENYL)IMINO]-2,5-DIHYDROPYRIMIDIN-4-YL}-1,3-THIAZOL-2-AMINE	-1.57	-0.882	-1.694	-2.361	-2.01	0.044	0.119	0.028	0.009	0.02
DB08233	6-CYCLOHEXYLMETHYLOXY-2-(4'-HYDROXYANILINO)PURINE	-1.57	-0.882	-1.694	-2.361	-2.01	0.044	0.119	0.028	0.009	0.02
DB08241	4-(6-CYCLOHEXYLMETHOXY-9H-PURIN-2-YLAMINO)--BENZAMIDE	-1.57	-0.882	-1.694	-2.361	-2.01	0.044	0.119	0.028	0.009	0.02
DB08248	3-(6-CYCLOHEXYLMETHOXY-9H-PURIN-2-YLAMINO)-BENZENESULFONAMIDE	-1.57	-0.882	-1.694	-2.361	-2.01	0.044	0.119	0.028	0.009	0.02
DB08527	1-[4-(AMINOSULFONYL)PHENYL]-1,6-DIHYDROPYRAZOLO[3,4-E]INDAZOLE-3-CARBOXAMIDE	-1.57	-0.882	-1.694	-2.361	-2.01	0.044	0.119	0.028	0.009	0.02
DB01370	Aluminium	-1.565	-1.071	0.319	-0.894	-1.555	0.042	0.077	0.401	0.133	0.047
DB02197	4-[(4-Imidazo[1,2-a]Pyridin-3-Ylpyrimidin-2-Yl)Amino]Benzenesulfonamide	-1.56	-1.094	-0.171	-2.348	-2.061	0.042	0.083	0.345	0.01	0.017
DB00269	Chlorotrianisene	-1.553	-0.532	-0.983	-2.922	-2.234	0.047	0.184	0.117	0.01	0.012
DB01878	Benzophenone	-1.553	-0.532	-0.983	-2.922	-2.234	0.047	0.184	0.117	0.01	0.012
DB04020	4-(2-{[4-{[3-(4-Chlorophenyl)Propyl]Sulfanyl}-6-(1-Piperazinyl)-1,3,5-Triazin-2-Yl]Amino}Ethyl)Phenol	-1.553	-0.532	-0.983	-2.922	-2.234	0.047	0.184	0.117	0.01	0.012
DB05414	Pipendoxifene	-1.553	-0.532	-0.983	-2.922	-2.234	0.047	0.184	0.117	0.01	0.012
DB06401	Bazedoxifene	-1.553	-0.532	-0.983	-2.922	-2.234	0.047	0.184	0.117	0.01	0.012
DB06937	4-(6-HYDROXY-BENZO[D]ISOXAZOL-3-YL)BENZENE-1,3-DIOL	-1.553	-0.532	-0.983	-2.922	-2.234	0.047	0.184	0.117	0.01	0.012
DB07036	(3aS,4R,9bR)-2,2-difluoro-4-(4-hydroxyphenyl)-6-(methoxymethyl)-1,2,3,3a,4,9b-hexahydrocyclopenta[c]chromen-8-ol	-1.553	-0.532	-0.983	-2.922	-2.234	0.047	0.184	0.117	0.01	0.012
DB07567	(2R,3R,4S)-3-(4-HYDROXYPHENYL)-4-METHYL-2-[4-(2-PYRROLIDIN-1-YLETHOXY)PHENYL]CHROMAN-6-OL	-1.553	-0.532	-0.983	-2.922	-2.234	0.047	0.184	0.117	0.01	0.012
DB07638	(3AS,4R,9BR)-2,2-DIFLUORO-4-(4-HYDROXYPHENYL)-1,2,3,3A,4,9B-HEXAHYDROCYCLOPENTA[C]CHROMEN-8-OL	-1.553	-0.532	-0.983	-2.922	-2.234	0.047	0.184	0.117	0.01	0.012
DB07757	(9aS)-4-bromo-9a-butyl-7-hydroxy-1,2,9,9a-tetrahydro-3H-fluoren-3-one	-1.553	-0.532	-0.983	-2.922	-2.234	0.047	0.184	0.117	0.01	0.012
DB07933	Erteberel	-1.553	-0.532	-0.983	-2.922	-2.234	0.047	0.184	0.117	0.01	0.012
DB07991	N-[(1R)-3-(4-HYDROXYPHENYL)-1-METHYLPROPYL]-2-(2-PHENYL-1H-INDOL-3-YL)ACETAMIDE	-1.553	-0.532	-0.983	-2.922	-2.234	0.047	0.184	0.117	0.01	0.012
DB08020	(3AS,4R,9BR)-4-(4-HYDROXYPHENYL)-6-(METHOXYMETHYL)-1,2,3,3A,4,9B-HEXAHYDROCYCLOPENTA[C]CHROMEN-8-OL	-1.553	-0.532	-0.983	-2.922	-2.234	0.047	0.184	0.117	0.01	0.012

DB08737	(3AS,4R,9BR)-4-(4-HYDROXYPHENYL)-1,2,3,3A,4,9B-HEXAHYDROCYCLOPENTA[C]CHROMEN-9-OL	-1.553	-0.532	-0.983	-2.922	-2.234	0.047	0.184	0.117	0.01	0.012
DB09535	Octocrylene	-1.553	-0.532	-0.983	-2.922	-2.234	0.047	0.184	0.117	0.01	0.012
DB07715	Emodin	-1.537	-0.291	-1.986	-2.183	-1.745	0.035	0.268	0.023	0.016	0.029
DB00851	Dacarbazine	-1.53	-0.798	-0.147	-1.119	-0.408	0.036	0.171	0.125	0.08	0.259
DB06412	Oxymetholone	-1.526	-0.069	-2.646	-3.399	-1.196	0.041	0.354	0.006	0.007	0.059
DB00396	Progesterone	-1.525	-0.896	0.441	-1.028	-1.524	0.034	0.113	0.57	0.117	0.051
DB07471	5-[5,6-BIS(METHYLOXY)-1H-BENZIMIDAZOL-1-YL]-3-{[1-(2-CHLOROPHENYL)ETHYL]OXY}-2-THIOPHENECARBOXAMIDE	-1.521	-1.423	-0.867	-1.573	-0.51	0.036	0.04	0.143	0.04	0.202
DB02901	Stanolone	-1.516	-0.434	-1.395	-2.901	-1.55	0.043	0.226	0.06	0.006	0.037
DB03801	Lysine Nz-Carboxylic Acid	-1.509	-0.669	-0.304	-2.857	-2.39	0.04	0.163	0.307	0.005	0.013
DB00808	Indapamide	-1.506	-0.681	1.124	-0.658	-1.131	0.055	0.199	0.808	0.174	0.104
DB08683	REL-(9R,12S)-9,10,11,12-TETRAHYDRO-9,12-EPOXY-1H-DIINDOLO[1,2,3-FG:3',2',1'-KL]PYRROLO[3,4-I][1,6]BENZODIAZOCINE-1,3(2H)-DIONE	-1.506	-0.836	0.291	-2.352	-2.036	0.045	0.137	0.548	0.011	0.019
DB09257	Gimeracil	-1.501	-1.369	-1.157	-1.418	-1.685	0.009	0.106	0.102	0.016	0.002
DB01169	Arsenic trioxide	-1.492	-1.212	-1.406	-2.131	-0.943	0.04	0.056	0.062	0.005	0.123
DB02709	Resveratrol	-1.492	-0.995	-0.037	-2.178	-1.589	0.034	0.089	0.403	0.022	0.043
DB04224	Oleic Acid	-1.488	-0.897	1.589	-1.503	0.01	0.06	0.158	0.921	0.053	0.44
DB04407	4-[4-(4-Methyl-2-Methylamino-Thiazol-5-Yl)-Pyrimidin-2-Ylamino]-Phenol	-1.477	-1.896	-0.052	-1.452	-0.773	0.034	0.021	0.295	0.046	0.113
DB04574	Estrone sulfate	-1.475	-0.52	-1.047	-3.255	-1.762	0.037	0.212	0.103	0.005	0.025
DB00746	Deferoxamine	-1.473	-0.946	-1.87	-1.388	-1.618	0.046	0.102	0.032	0.077	0.032
DB07485	4,4'-cyclohexane-1,1-diyldiphenol	-1.473	-0.604	-0.889	-2.619	-2.056	0.041	0.169	0.137	0.011	0.014
DB00337	Pimecrolimus	-1.463	-0.7	0.274	-0.032	-0.948	0.058	0.202	0.394	0.383	0.125
DB00389	Carbimazole	-1.459	-1.276	-0.671	-1.335	-1.546	0.016	0.13	0.291	0.064	0.004
DB00984	Nandrolone phenpropionate	-1.459	-0.364	-0.983	-3.697	-1.956	0.052	0.247	0.117	0.003	0.017
DB07558	acetylleucyl-leucyl-norleucinal	-1.458	-1.002	0.083	-0.314	-0.492	0.04	0.122	0.136	0.305	0.236
DB02235	L-methionine (R)-S-oxide	-1.456	-1.37	-0.226	-1.557	-1.57	0.049	0.076	0.329	0.045	0.03
DB06714	Propylhexedrine	-1.449	-1.369	-0.778	-1.418	-1.607	0.03	0.106	0.258	0.016	0.007
DB00288	Amcinonide	-1.448	-1.056	0.262	-0.899	-1.733	0.046	0.12	0.21	0.112	0.016
DB00850	Perphenazine	-1.44	-1.082	1.125	0.002	-1.069	0.044	0.081	0.821	0.426	0.093
DB01380	Cortisone acetate	-1.439	-1.328	0.863	-0.258	-1.885	0.062	0.066	0.689	0.312	0.023
DB07460	2-({5-CHLORO-2-[(2-METHOXY-4-MORPHOLIN-4-YLPHENYL)AMINO]PYRIMIDIN-4-YL}AMINO)-N-METHYLBENZAMIDE	-1.435	-0.768	0.338	-0.519	-0.724	0.058	0.166	0.236	0.226	0.173
DB00305	Mitomycin	-1.434	-1.416	-0.78	-1.445	-1.62	0.013	0.054	0.264	0.014	0.008
DB04205	Thymidine-3',5'-Diphosphate	-1.432	-0.085	-0.167	-3.193	-1.503	0.031	0.431	0.103	0	0.036
DB06202	Lasofoxifene	-1.426	-0.618	-0.575	-2.584	-1.784	0.044	0.214	0.178	0.01	0.023
DB00061	Pegademase	-1.416	-1.519	0.411	-1.828	-1.385	0.049	0.04	0.328	0.024	0.043
DB00717	Norethisterone	-1.416	-0.477	-1.034	-2.881	-1.945	0.048	0.204	0.102	0.004	0.023
DB09495	Avobenzone	-1.41	-1.37	-0.78	-1.445	-1.62	0.02	0.093	0.264	0.014	0.008
DB00864	Tacrolimus	-1.408	-2.018	-0.16	1.039	1.25	0.062	0.016	0.291	0.806	0.875
DB00945	Acetylsalicylic acid	-1.406	-0.708	-0.885	-1.646	-0.966	0.044	0.155	0.147	0.04	0.119
DB06718	Stanozolol	-1.405	-0.039	-0.352	-3.335	-1.782	0.049	0.384	0.253	0.006	0.022
DB01366	Procaterol	-1.4	-0.56	-1.099	0.295	-1.629	0.058	0.235	0.06	0.531	0.027
DB03428	SU9516	-1.395	-0.717	-1.114	-1.636	-1.091	0.049	0.16	0.088	0.029	0.069
DB08355	1-methyl-8-(phenylamino)-4,5-dihydro-1H-pyrazolo[4,3-h]quinazoline-3-carboxylic acid	-1.393	-0.913	-0.356	-1.511	-0.67	0.051	0.111	0.262	0.04	0.154

DB01915	S-Hydroxycysteine	-1.383	-2.236	2.471	-0.81	-0.189	0.064	0.011	0.981	0.152	0.351
DB01259	Lapatinib	-1.373	-0.84	0.036	-0.863	-1.264	0.041	0.139	0.428	0.147	0.077
DB00005	Etanercept	-1.36	-1.561	-0.398	-0.053	0.023	0.072	0.052	0.2	0.391	0.423
DB11581	Venetoclax	-1.358	-1.097	0.165	-1.14	0.186	0.055	0.104	0.183	0.076	0.479
DB00522	Bentiromide	-1.348	-1.132	-0.951	-1.204	-1.543	0.007	0.122	0.146	0.026	0.002
DB00535	Cefdinir	-1.348	-0.534	1.894	-3.152	-2.296	0.052	0.191	0.978	0.006	0.014
DB08361	2-{[(1R,2S)-2-aminocyclohexyl]amino}-4-[(3-methylphenyl)amino]pyrimidine-5-carboxamide	-1.347	-1.831	-1.031	0.115	0.043	0.064	0.023	0.073	0.455	0.452
DB09063	Ceritinib	-1.343	-0.842	0.663	0.55	-1.646	0.05	0.129	0.679	0.662	0.052
DB04794	Bifonazole	-1.33	-0.367	-0.944	-2.62	-1.23	0.061	0.263	0.092	0.005	0.053
DB07592	(2R)-3-Methyl-1-phenyl-2-butanyl [(2S)-1-oxo-2-hexanyl]carbamate	-1.329	-0.131	0.515	-2.435	-0.617	0.051	0.382	0.549	0.009	0.192
DB07164	N-cyclopropyl-4-pyrazolo[1,5-b]pyridazin-3-ylpyrimidin-2-amine	-1.327	-0.909	-0.542	-1.132	-1.057	0.052	0.106	0.222	0.085	0.067
DB00002	Cetuximab	-1.326	-1.038	0.504	-0.39	-1.032	0.06	0.104	0.552	0.259	0.097
DB01493	Ethylestrenol	-1.324	0.092	-0.974	-3.229	-1.598	0.062	0.464	0.108	0.004	0.039
DB01406	Danazol	-1.322	-0.362	0.779	-0.966	-1.629	0.064	0.275	0.726	0.128	0.038
DB09042	Tedizolid phosphate	-1.321	-1.132	-0.563	-1.204	-1.543	0.013	0.122	0.335	0.026	0.002
DB09244	Pirlindole	-1.321	-1.132	-0.563	-1.204	-1.543	0.013	0.122	0.335	0.026	0.002
DB00957	Norgestimate	-1.319	-0.461	-0.79	-3.706	-1.503	0.054	0.243	0.158	0.004	0.036
DB01431	Allylestrenol	-1.319	-0.461	-0.79	-3.706	-1.503	0.054	0.243	0.158	0.004	0.036
DB11064	Homosalate	-1.319	-0.461	-0.79	-3.706	-1.503	0.054	0.243	0.158	0.004	0.036
DB03796	Palmitic Acid	-1.313	-0.691	0.613	1.082	-1.553	0.083	0.198	0.604	0.781	0.051
DB00749	Etodolac	-1.3	-0.474	1.566	-2.424	-0.604	0.06	0.248	0.905	0.005	0.186
DB00472	Fluoxetine	-1.297	-0.208	-1.008	-0.07	-0.98	0.06	0.337	0.119	0.399	0.119
DB09070	Tibolone	-1.29	-0.238	-0.047	-2.777	-1.654	0.049	0.291	0.377	0.007	0.036
DB09033	Vedolizumab	-1.28	-0.198	1.184	-3.826	-2.612	0.056	0.33	0.849	0	0.009
DB01718	Cetrimonium	-1.278	-0.651	-1.312	-2.493	-0.975	0.054	0.165	0.075	0.008	0.111
DB00348	Nitisinone	-1.276	-1.232	-0.664	-1.415	-1.525	0.037	0.132	0.284	0.011	0.008
DB00719	Azatadine	-1.276	-1.232	-0.664	-1.415	-1.525	0.037	0.132	0.284	0.011	0.008
DB00748	Carbinoxamine	-1.276	-1.232	-0.664	-1.415	-1.525	0.037	0.132	0.284	0.011	0.008
DB00792	Tripelennamine	-1.276	-1.232	-0.664	-1.415	-1.525	0.037	0.132	0.284	0.011	0.008
DB00902	Methdilazine	-1.276	-1.232	-0.664	-1.415	-1.525	0.037	0.132	0.284	0.011	0.008
DB01084	Emedastine	-1.276	-1.232	-0.664	-1.415	-1.525	0.037	0.132	0.284	0.011	0.008
DB01237	Bromodiphenhydramine	-1.276	-1.232	-0.664	-1.415	-1.525	0.037	0.132	0.284	0.011	0.008
DB01615	Aceprometazine	-1.276	-1.232	-0.664	-1.415	-1.525	0.037	0.132	0.284	0.011	0.008
DB01619	Phenindamine	-1.276	-1.232	-0.664	-1.415	-1.525	0.037	0.132	0.284	0.011	0.008
DB04837	Clofedanol	-1.276	-1.232	-0.664	-1.415	-1.525	0.037	0.132	0.284	0.011	0.008
DB04890	Bepotastine	-1.276	-1.232	-0.664	-1.415	-1.525	0.037	0.132	0.284	0.011	0.008
DB06282	Levocetirizine	-1.276	-1.232	-0.664	-1.415	-1.525	0.037	0.132	0.284	0.011	0.008
DB06766	Alcaftadine	-1.276	-1.232	-0.664	-1.415	-1.525	0.037	0.132	0.284	0.011	0.008
DB08802	Isothipendyl	-1.276	-1.232	-0.664	-1.415	-1.525	0.037	0.132	0.284	0.011	0.008
DB09555	Dexchlorpheniramine maleate	-1.276	-1.232	-0.664	-1.415	-1.525	0.037	0.132	0.284	0.011	0.008
DB11591	Bilastine	-1.276	-1.232	-0.664	-1.415	-1.525	0.037	0.132	0.284	0.011	0.008
DB06888	(13R,15S)-13-METHYL-16-OXA-8,9,12,22,24-PENTAAZAHEXACYCLO[15.6.2.16,9.1,12,15.0,2,7.0,21,25]HEPTACOSA-1(24),2,4,6,17(25),18,20-HEPTAENE-23,26-DIONE	-1.272	-0.869	0.265	-1.759	-1.678	0.066	0.111	0.525	0.028	0.029
DB07540	4-{5-[(1Z)-1-(2-IMINO-4-OXO-1,3-THIAZOLIDIN-5-YLIDENE)ETHYL]-2-FURYL}BENZENESULFONAMIDE	-1.272	-0.869	0.265	-1.759	-1.678	0.066	0.111	0.525	0.028	0.029
DB07686	4-{[5-(CYCLOHEXYLAMINO)[1,2,4]TRIAZOLO[1,5-A]PYRIMIDIN-7-YL]AMINO}BENZENESULFONAMIDE	-1.272	-0.869	0.265	-1.759	-1.678	0.066	0.111	0.525	0.028	0.029

DB07687	4-({5-[(4-AMINOCYCLOHEXYL)AMINO][1,2,4]TRIAZOLO[1,5-A]PYRIMIDIN-7-YL}AMINO)BENZENESULFONAMIDE	-1.272	-0.869	0.265	-1.759	-1.678	0.066	0.111	0.525	0.028	0.029
DB08814	Triflusal	-1.272	0.105	-0.422	-2.993	-1.717	0.088	0.461	0.251	0.004	0.037
DB11611	Lifitegrast	-1.263	0.437	-0.267	-3.013	-3.21	0.019	0.695	0.106	0.003	0
DB00811	Ribavirin	-1.255	-0.445	-2.591	-1.752	0.142	0.091	0.271	0.009	0.028	0.465
DB00823	Ethynodiol diacetate	-1.25	-0.32	-0.962	-3.76	-1.648	0.054	0.274	0.125	0.005	0.026
DB04552	Niflumic acid	-1.25	-2.073	1.824	-0.304	0.411	0.085	0.01	0.932	0.28	0.565
DB00727	Nitroglycerin	-1.243	-1.061	-0.95	-1.25	-1.453	0.035	0.196	0.165	0.024	0.009
DB00883	Isosorbide dinitrate	-1.243	-1.061	-0.95	-1.25	-1.453	0.035	0.196	0.165	0.024	0.009
DB01612	Amyl Nitrite	-1.243	-1.061	-0.95	-1.25	-1.453	0.035	0.196	0.165	0.024	0.009
DB08047	4-[1-allyl-7-(trifluoromethyl)-1H-indazol-3-yl]benzene-1,3-diol	-1.243	-0.414	-0.734	-2.543	-1.491	0.054	0.257	0.178	0.008	0.037
DB08773	RALOXIFENE CORE	-1.243	-0.414	-0.734	-2.543	-1.491	0.054	0.257	0.178	0.008	0.037
DB00051	Adalimumab	-1.242	-1.692	-1.262	-0.515	0.73	0.078	0.039	0.073	0.236	0.694
DB00603	Medroxyprogesterone acetate	-1.24	-0.682	-1.301	-2.944	-1.839	0.058	0.173	0.063	0.005	0.022
DB09213	Dexibuprofen	-1.237	-0.601	1.408	0.242	-1.462	0.089	0.223	0.885	0.521	0.041
DB04491	Diisopropylphosphono Group	-1.233	-1.211	0.199	-1.211	-0.778	0.014	0.045	0.469	0.053	0.122
DB01219	Dantrolene	-1.231	-0.839	1.384	-0.787	-1.538	0.027	0.121	0.852	0.15	0.023
DB06832	2-(3-FLUORO-4-HYDROXYPHENYL)-7-VINYL-1,3-BENZOXAZOL-5-OL	-1.231	-0.377	-0.758	-2.547	-1.719	0.055	0.271	0.169	0.008	0.021
DB06875	3-(3-FLUORO-4-HYDROXYPHENYL)-7-HYDROXY-1-NAPHTHONITRILE	-1.231	-0.377	-0.758	-2.547	-1.719	0.055	0.271	0.169	0.008	0.021
DB06927	[5-HYDROXY-2-(4-HYDROXYPHENYL)-1-BENZOFURAN-7-YL]ACETONITRILE	-1.231	-0.377	-0.758	-2.547	-1.719	0.055	0.271	0.169	0.008	0.021
DB07009	2-(5-HYDROXY-NAPHTHALEN-1-YL)-1,3-BENZOOXAZOL-6-OL	-1.231	-0.377	-0.758	-2.547	-1.719	0.055	0.271	0.169	0.008	0.021
DB07119	1-CHLORO-6-(4-HYDROXYPHENYL)-2-NAPHTHOL	-1.231	-0.377	-0.758	-2.547	-1.719	0.055	0.271	0.169	0.008	0.021
DB07150	4-(4-HYDROXYPHENYL)-1-NAPHTHALDEHYDE OXIME	-1.231	-0.377	-0.758	-2.547	-1.719	0.055	0.271	0.169	0.008	0.021
DB07198	5-HYDROXY-2-(4-HYDROXYPHENYL)-1-BENZOFURAN-7-CARBONITRILE	-1.231	-0.377	-0.758	-2.547	-1.719	0.055	0.271	0.169	0.008	0.021
DB07230	3-BROMO-6-HYDROXY-2-(4-HYDROXYPHENYL)-1H-INDEN-1-ONE	-1.231	-0.377	-0.758	-2.547	-1.719	0.055	0.271	0.169	0.008	0.021
DB07236	3-(6-HYDROXY-NAPHTHALEN-2-YL)-BENZO[D]ISOOXAZOL-6-OL	-1.231	-0.377	-0.758	-2.547	-1.719	0.055	0.271	0.169	0.008	0.021
DB07268	2-({2-[(3-HYDROXYPHENYL)AMINO]PYRIMIDIN-4-YL}AMINO)BENZAMIDE	-1.224	-1.134	0.186	-1.731	-0.145	0.064	0.053	0.407	0.022	0.34
DB09462	Glycerin	-1.215	-1.947	0.505	1.01	-0.242	0.092	0.019	0.558	0.801	0.338
DB04815	Clioquinol	-1.212	-0.87	-0.546	-0.975	0.071	0.06	0.11	0.232	0.129	0.429
DB06713	Norelgestromin	-1.206	-0.212	-0.663	-3.215	-1.414	0.057	0.303	0.191	0.005	0.043
DB02216	S-Methylcysteine	-1.205	-0.628	-0.335	-2.971	-2.169	0.02	0.183	0.077	0.002	0.001
DB00588	Fluticasone propionate	-1.204	-0.494	0.291	-1.953	-1.276	0.091	0.251	0.229	0.021	0.068
DB04817	Metamizole	-1.199	-1.184	-0.279	-1.563	-1.26	0.006	0.034	0.437	0.002	0.007
DB01051	Novobiocin	-1.194	-1.132	-0.592	-1.273	-1.427	0.061	0.174	0.321	0.021	0.009
DB01241	Gemfibrozil	-1.192	-1.005	1.304	0.987	-0.948	0.091	0.124	0.834	0.659	0.133
DB01196	Estramustine	-1.191	-0.223	-1.459	-2.36	-1.669	0.056	0.31	0.046	0.011	0.033
DB00584	Enalapril	-1.185	-0.743	0.414	-1.197	-1.289	0.015	0.141	0.546	0.058	0.021
DB08951	Indoprofen	-1.184	-0.328	-0.76	-1.656	-0.738	0.066	0.258	0.157	0.032	0.149
DB01178	Chlormezanone	-1.182	-1.159	-0.371	-1.119	-1.272	0.007	0.033	0.395	0.023	0.017
DB01185	Fluoxymesterone	-1.182	-0.283	-1.433	-3.22	-1.653	0.052	0.271	0.048	0.004	0.031
DB06905	(2S,3S,4E,6E,8S,9S)-3-amino-9-methoxy-2,6,8-trimethyl-10-phenyldeca-4,6-dienoic acid	-1.182	-0.244	0.641	-0.965	-1.895	0.091	0.352	0.389	0.109	0.016
DB01269	Panitumumab	-1.173	-0.884	0.411	-0.176	-1.872	0.071	0.121	0.328	0.292	0.02
DB09559	Necitumumab	-1.173	-0.884	0.411	-0.176	-1.872	0.071	0.121	0.328	0.292	0.02

DB00784	Mefenamic acid	-1.17	-0.967	1.886	-1.088	-1.851	0.1	0.137	0.948	0.098	0.033
DB01133	Tiludronic acid	-1.163	-0.436	-1.671	0.571	-1.213	0.104	0.282	0.018	0.572	0.073
DB01041	Thalidomide	-1.159	-0.642	-1.498	-1.801	-1.414	0.068	0.19	0.048	0.027	0.057
DB03721	N-acetyl-alpha-neuraminic acid	-1.156	-0.882	1.963	-1.61	0.174	0.101	0.142	0.952	0.039	0.471
DB07533	4-{5-[(Z)-(2-IMINO-4-OXO-1,3-THIAZOLIDIN-5-YLIDENE)METHYL]-2-FURYL}-N-METHYLBENZENESULFONAMIDE	-1.145	-0.956	-0.758	-1.396	-1.012	0.063	0.123	0.169	0.055	0.08
DB07534	4-{5-[(Z)-(2-IMINO-4-OXO-1,3-THIAZOLIDIN-5-YLIDENE)METHYL]FURAN-2-YL}BENZENESULFONAMIDE	-1.145	-0.956	-0.758	-1.396	-1.012	0.063	0.123	0.169	0.055	0.08
DB07538	4-{5-[(Z)-(2-IMINO-4-OXO-1,3-THIAZOLIDIN-5-YLIDENE)METHYL]FURAN-2-YL}-2-(TRIFLUOROMETHYL)BENZENESULFONAMIDE	-1.145	-0.956	-0.758	-1.396	-1.012	0.063	0.123	0.169	0.055	0.08
DB01296	Glucosamine	-1.142	-0.36	-1.711	-1.56	-0.177	0.109	0.307	0.022	0.029	0.346
DB03467	Naringenin	-1.127	-1.096	-0.171	-0.375	-1.174	0.093	0.105	0.246	0.206	0.083
DB00492	Fosinopril	-1.126	-1.054	-0.371	-1.119	-1.272	0.031	0.099	0.395	0.023	0.017
DB00519	Trandolapril	-1.126	-1.054	-0.371	-1.119	-1.272	0.031	0.099	0.395	0.023	0.017
DB00542	Benazepril	-1.126	-1.054	-0.371	-1.119	-1.272	0.031	0.099	0.395	0.023	0.017
DB01340	Cilazapril	-1.126	-1.054	-0.371	-1.119	-1.272	0.031	0.099	0.395	0.023	0.017
DB01348	Spirapril	-1.126	-1.054	-0.371	-1.119	-1.272	0.031	0.099	0.395	0.023	0.017
DB08836	Temocapril	-1.126	-1.054	-0.371	-1.119	-1.272	0.031	0.099	0.395	0.023	0.017
DB13166	Zofenopril	-1.126	-1.054	-0.371	-1.119	-1.272	0.031	0.099	0.395	0.023	0.017
DB08217	S-[(1-Hydroxy-2,2,5,5-tetramethyl-2,5-dihydro-1H-pyrrol-3-yl)methyl] methanesulfonothioate	-1.117	-1.125	1.416	-1.392	-2.069	0.001	0.009	0.886	0.024	0
DB03297	Benzylsulfinic Acid	-1.097	-0.988	0.286	-1.357	-0.961	0.006	0.034	0.591	0.019	0.032
DB04186	N'-(Pyrrolidino[2,1-B]Isoindolin-4-On-8-Yl)-N-(Pyridin-2-Yl)Urea	-1.097	-0.588	-0.299	-1.371	-1.122	0.091	0.203	0.294	0.05	0.076
DB00221	Isoetarine	-1.094	-0.4	-1.145	0.357	-1.436	0.114	0.288	0.054	0.565	0.049
DB07347	4-(2-Aminoethyl)Benzenesulfonyl Fluoride	-1.091	-0.161	0.249	-1.628	-1.899	0.091	0.395	0.219	0.031	0.017
DB01257	Eculizumab	-1.085	-0.942	-0.34	-1.057	-1.212	0.072	0.186	0.414	0.037	0.019
DB01649	7-methyl-GpppA	-1.085	-0.793	1.554	-1.209	-2.281	0.021	0.135	0.891	0.078	0
DB01960	7n-Methyl-8-Hydroguanosine-5'-Diphosphate	-1.085	-0.793	1.554	-1.209	-2.281	0.021	0.135	0.891	0.078	0
DB04573	Estriol	-1.084	-0.123	-1.837	-2.194	-1.39	0.069	0.353	0.028	0.014	0.048
DB00052	Somatotropin	-1.083	-0.823	-0.604	0.077	-1.481	0.009	0.089	0.052	0.314	0.005
DB00169	Cholecalciferol	-1.083	-0.535	1.234	0.229	-1.371	0.098	0.249	0.817	0.509	0.045
DB00839	Tolazamide	-1.08	-0.894	-0.258	-0.968	-1.313	0.049	0.225	0.437	0.036	0.001
DB04845	Ixabepilone	-1.079	-0.707	-0.875	-0.998	-2.288	0.042	0.174	0.157	0.085	0.001
DB00236	Pipobroman	-1.078	-1.038	-0.377	-1.137	-1.372	0.033	0.159	0.412	0.026	0.002
DB00414	Acetohexamide	-1.078	-1.038	-0.377	-1.137	-1.372	0.033	0.159	0.412	0.026	0.002
DB00916	Metronidazole	-1.078	-1.038	-0.377	-1.137	-1.372	0.033	0.159	0.412	0.026	0.002
DB09330	Osimertinib	-1.074	-0.653	0.652	0.424	-2.129	0.093	0.177	0.432	0.579	0.015
DB08441	6-BROMO-13-THIA-2,4,8,12,19-PENTAAZATRICYCLO[12.3.1.1~3,7~]NONADECA-1(18),3(19),4,6,14,16-HEXAENE 13,13-DIOXIDE	-1.072	-0.729	-0.786	-1.894	-1.094	0.068	0.187	0.159	0.019	0.069
DB01306	Insulin Aspart	-1.065	-1.195	0.296	0.283	-1.315	0.004	0.009	0.542	0.514	0
DB01307	Insulin Detemir	-1.065	-1.195	0.296	0.283	-1.315	0.004	0.009	0.542	0.514	0
DB01309	Insulin Glulisine	-1.065	-1.195	0.296	0.283	-1.315	0.004	0.009	0.542	0.514	0
DB09564	Insulin Degludec	-1.065	-1.195	0.296	0.283	-1.315	0.004	0.009	0.542	0.514	0
DB03271		-1.063	-0.042	0.971	-0.921	-2.84	0.087	0.476	0.745	0.114	0
DB00655	Estrone	-1.059	-0.108	-1.217	-1.916	-1.896	0.068	0.368	0.086	0.017	0.018
DB04652	Corticosterone	-1.058	-0.93	0.757	1.573	-2.521	0.12	0.145	0.656	0.916	0.003

DB06698	Betahistine	-1.058	-0.97	-0.645	-1.176	-1.26	0.077	0.193	0.263	0.021	0.007
DB04751	Purvalanol A	-1.057	-0.376	0.089	-0.789	-1.343	0.074	0.244	0.453	0.154	0.058
DB00240	Alclometasone	-1.056	-0.844	-0.111	-1.159	-1.169	0.049	0.099	0.142	0.066	0.035
DB00592	Piperazine	-1.056	-0.887	-0.34	-1.057	-1.212	0.097	0.256	0.414	0.037	0.019
DB00596	Ulobetasol	-1.056	-0.844	-0.111	-1.159	-1.169	0.049	0.099	0.142	0.066	0.035
DB00663	Flumethasone	-1.056	-0.844	-0.111	-1.159	-1.169	0.049	0.099	0.142	0.066	0.035
DB00769	Hydrocortamate	-1.056	-0.844	-0.111	-1.159	-1.169	0.049	0.099	0.142	0.066	0.035
DB00838	Clocortolone	-1.056	-0.844	-0.111	-1.159	-1.169	0.049	0.099	0.142	0.066	0.035
DB00873	Loteprednol	-1.056	-0.844	-0.111	-1.159	-1.169	0.049	0.099	0.142	0.066	0.035
DB01263	Posaconazole	-1.056	-0.887	-0.34	-1.057	-1.212	0.097	0.256	0.414	0.037	0.019
DB01384	Paramethasone	-1.056	-0.844	-0.111	-1.159	-1.169	0.049	0.099	0.142	0.066	0.035
DB06781	Difluprednate	-1.056	-0.844	-0.111	-1.159	-1.169	0.049	0.099	0.142	0.066	0.035
DB08906	Fluticasone furoate	-1.056	-0.844	-0.111	-1.159	-1.169	0.049	0.099	0.142	0.066	0.035
DB13158	Clobetasone	-1.056	-0.844	-0.111	-1.159	-1.169	0.049	0.099	0.142	0.066	0.035
DB01593	Zinc	-1.055	-1.733	-0.009	-0.036	-0.107	0.126	0.057	0.45	0.384	0.38
DB01277	Mecasermin	-1.054	-0.366	0.246	1.488	-1.955	0.115	0.303	0.206	0.838	0.009
DB07336	4-[3-(1H-BENZIMIDAZOL-2-YL)-1H-INDAZOL-6-YL]-2-METHOXYPHENOL	-1.044	-0.266	-0.052	-2.309	-1.074	0.094	0.29	0.287	0.01	0.086
DB03365	4-[3-Hydroxyanilino]-6,7-Dimethoxyquinazoline	-1.038	-0.293	-0.282	-2.025	-0.605	0.096	0.274	0.221	0.018	0.16
DB00552	Pentostatin	-1.035	-0.896	-1.547	-1.422	-1.324	0.019	0.118	0.002	0.001	0.005
DB01805	Monoisopropylphosphorylserine	-1.03	-1.258	0.761	-1.233	-0.636	0.015	0.018	0.735	0.044	0.151
DB06237	Avanafil	-1.03	-0.916	-0.279	-0.983	-1.174	0.102	0.256	0.437	0.112	0.029
DB00571	Propranolol	-1.02	-1.415	-1.095	1.158	-0.063	0.137	0.07	0.087	0.846	0.395
DB06317	Elotuzumab	-1.018	-0.833	-0.258	-0.968	-1.219	0.093	0.277	0.437	0.036	0.003
DB00648	Mitotane	-1.014	0.399	0.222	-2.313	-1.219	0.099	0.6	0.476	0.016	0.077
DB07664	5-AMINO-3-{[4-(AMINOSULFONYL)PHENYL]AMINO}-N-(2,6-DIFLUOROPHENYL)-1H-1,2,4-TRIAZOLE-1-CARBOTHIOAMIDE	-1.012	-0.556	-1.04	-1.67	-0.8	0.076	0.2	0.113	0.038	0.149
DB09031	Miltefosine	-1.003	-1.836	-1.025	-1.235	-0.354	0.077	0.023	0.075	0.079	0.247
DB07431	(3R)-3-(aminomethyl)-9-methoxy-1,2,3,4-tetrahydro-5H-[1]benzothieno[3,2-e][1,4]diazepin-5-one	-1	-0.538	-0.819	-1.982	-1.145	0.079	0.232	0.153	0.017	0.063
DB06689	Ethanolamine oleate	-0.998	-0.634	2.244	0.359	-0.724	0.135	0.223	0.985	0.554	0.195
DB08348	N~2~,N~2~-DIMETHYL-N~1~-(6-OXO-5,6-DIHYDROPHENANTHRIDIN-2-YL)GLYCINAMIDE	-0.995	-0.043	-1.727	-2.458	0.028	0.137	0.431	0.032	0.006	0.444
DB03490	3-Pyridin-4-Yl-2,4-Dihydro-Indeno[1,2-.C.]Pyrazole	-0.991	-0.196	-0.983	-1.759	-1.678	0.098	0.315	0.117	0.028	0.029
DB04288	2-[Trans-(4-Aminocyclohexyl)Amino]-6-(Benzyl-Amino)-9-Cyclopentylpurine	-0.991	-0.196	-0.983	-1.759	-1.678	0.098	0.315	0.117	0.028	0.029
DB07024	2-(3,4-DIHYDROXYPHENYL)-8-(1,1-DIOXIDOISOTHIAZOLIDIN-2-YL)-3-HYDROXY-6-METHYL-4H-CHROMEN-4-ONE	-0.991	-0.196	-0.983	-1.759	-1.678	0.098	0.315	0.117	0.028	0.029
DB07936	N-(4-{[(3S)-3-(dimethylamino)pyrrolidin-1-yl]carbonyl}phenyl)-5-fluoro-4-[2-methyl-1-(1-methylethyl)-1H-imidazol-5-yl]pyrimidin-2-amine	-0.991	-0.196	-0.983	-1.759	-1.678	0.098	0.315	0.117	0.028	0.029
DB08122	N-METHYL-4-{[(2-OXO-1,2-DIHYDRO-3H-INDOL-3-YLIDENE)METHYL]AMINO}BENZENESULFONAMIDE	-0.991	-0.196	-0.983	-1.759	-1.678	0.098	0.315	0.117	0.028	0.029

DB08123	N-METHYL-{4-[2-(7-OXO-6,7-DIHYDRO-8H-[1,3]THIAZOLO[5,4-E]INDOL-8-YLIDENE)HYDRAZINO]PHENYL}METHANE SULFONAMIDE	-0.991	-0.196	-0.983	-1.759	-1.678	0.098	0.315	0.117	0.028	0.029
DB08124	3-{[(2,2-DIOXIDO-1,3-DIHYDRO-2-BENZOTHIEN-5-YL)AMINO]METHYLENE}-5-(1,3-OXAZOL-5-YL)-1,3-DIHYDRO-2H-INDOL-2-ONE	-0.991	-0.196	-0.983	-1.759	-1.678	0.098	0.315	0.117	0.028	0.029
DB08125	4-{[(2-OXO-1,2-DIHYDRO-3H-INDOL-3-YLIDENE)METHYL]AMINO}-N-(1,3-THIAZOL-2-YL)BENZENESULFONAMIDE	-0.991	-0.196	-0.983	-1.759	-1.678	0.098	0.315	0.117	0.028	0.029
DB08126	3-{[4-([AMINO(IMINO)METHYL]AMINOSULFONYL)ANILINO]METHYLENE}-2-OXO-2,3-DIHYDRO-1H-INDOLE	-0.991	-0.196	-0.983	-1.759	-1.678	0.098	0.315	0.117	0.028	0.029
DB08138	{[(2,6-difluorophenyl)carbonyl]amino}-N-(4-fluorophenyl)-1H-pyrazole-3-carboxamide	-0.991	-0.196	-0.983	-1.759	-1.678	0.098	0.315	0.117	0.028	0.029
DB08141	4-{[(2,6-difluorophenyl)carbonyl]amino}-N-[(3S)-piperidin-3-yl]-1H-pyrazole-3-carboxamide	-0.991	-0.196	-0.983	-1.759	-1.678	0.098	0.315	0.117	0.028	0.029
DB00974	Edetic Acid	-0.988	-0.852	-0.238	-0.955	-1.287	0.041	0.206	0.461	0.043	0.002
DB01411	Pranlukast	-0.988	0.122	-1.488	-0.76	-1.098	0.106	0.456	0.057	0.174	0.065
DB09039	Eliglustat	-0.988	-0.852	-0.238	-0.955	-1.287	0.041	0.206	0.461	0.043	0.002
DB00634	Sulfacetamide	-0.984	-0.875	-0.029	-0.841	-1.253	0.009	0.08	0.527	0.05	0
DB01131	Proguanil	-0.984	-0.875	-0.029	-0.841	-1.253	0.009	0.08	0.527	0.05	0
DB02015	Dihydrofolic Acid	-0.984	-0.875	-0.029	-0.841	-1.253	0.009	0.08	0.527	0.05	0
DB08741	5-[[(2R)-2-cyclopropyl-7,8-dimethoxy-2H-chromen-5-yl]methyl]pyrimidine-2,4-diamine	-0.984	-0.875	-0.029	-0.841	-1.253	0.009	0.08	0.527	0.05	0
DB08878	Aminopterin	-0.984	-0.875	-0.029	-0.841	-1.253	0.009	0.08	0.527	0.05	0
DB09086	Eugenol	-0.978	0.143	-1.436	-3.016	-1.174	0.107	0.479	0.051	0.009	0.077
DB01686	N,N-dimethylarginine	-0.977	0.324	1.041	-0.519	-3.333	0.119	0.559	0.74	0.219	0
DB02207	7-Nitroindazole	-0.977	0.324	1.041	-0.519	-3.333	0.119	0.559	0.74	0.219	0
DB02234	S-Ethylisothiourea	-0.977	0.324	1.041	-0.519	-3.333	0.119	0.559	0.74	0.219	0
DB03100	6-Nitroindazole	-0.977	0.324	1.041	-0.519	-3.333	0.119	0.559	0.74	0.219	0
DB04534	5-Nitroindazole	-0.977	0.324	1.041	-0.519	-3.333	0.119	0.559	0.74	0.219	0
DB07001	(3S,5E)-3-propyl-3,4-dihydrothieno[2,3-f][1,4]oxazepin-5(2H)-imine	-0.977	0.324	1.041	-0.519	-3.333	0.119	0.559	0.74	0.219	0
DB07388	ETHYL 4-[(4-METHYLPYRIDIN-2-YL)AMINO]PIPERIDINE-1-CARBOXYLATE	-0.977	0.324	1.041	-0.519	-3.333	0.119	0.559	0.74	0.219	0
DB00764	Mometasone	-0.972	-0.568	0.198	-1.743	-1.117	0.111	0.243	0.198	0.028	0.083
DB02930	Adenosine 5'-[??-thio]triphosphate	-0.968	-0.863	0.157	0.074	-1.384	0.01	0.074	0.478	0.333	0.006
DB00046	Insulin Lispro	-0.966	-0.97	1.04	1.231	-1.049	0.099	0.124	0.774	0.753	0.093
DB00047	Insulin glargine	-0.966	-0.97	1.04	1.231	-1.049	0.099	0.124	0.774	0.753	0.093
DB00867	Ritodrine	-0.966	-0.355	-2.146	0.061	-1.657	0.137	0.311	0.009	0.421	0.02
DB03382	S-oxy-L-cysteine	-0.959	-1.07	-0.477	1.496	-0.002	0.141	0.107	0.108	0.93	0.41
DB01125	Anisindione	-0.958	-0.852	-0.238	-0.955	-1.198	0.058	0.206	0.461	0.043	0.013
DB02366	CRA_10762	-0.958	-0.607	0.429	-0.9	-1.271	0.036	0.201	0.554	0.093	0.025
DB09269	Phenylacetic acid	-0.95	-0.823	-0.084	-0.818	-1.18	0.031	0.148	0.509	0.069	0.002
DB05482	7-ethyl-10-hydroxycamptothecin	-0.947	-0.094	0.321	-3.462	-1.539	0.046	0.478	0.521	0	0.007

DB06884	4-HYDROXY-N'-(4-ISOPROPYLBENZYL)BENZOHYDRAZIDE	-0.947	-0.686	0.756	0.697	-1.69	0.073	0.168	0.635	0.548	0.011
DB01331	Cefoxitin	-0.946	-0.912	-0.735	-1	-1.139	0.064	0.215	0.202	0.041	0.028
DB00827	Cinoxacin	-0.943	-0.791	-0.071	-1.329	-1.193	0.037	0.209	0.494	0.002	0.005
DB07177	(5E,13E)-11-HYDROXY-9,15-DIOXOPROSTA-5,13-DIEN-1-OIC ACID	-0.943	-0.779	-0.313	-0.918	-1.198	0.048	0.235	0.404	0.042	0.005
DB09343	Tipiracil	-0.943	-0.791	-0.071	-0.865	-1.193	0.037	0.209	0.494	0.045	0.005
DB05804	Prasterone sulfate	-0.941	-0.78	-0.645	-0.968	-1.14	0.072	0.254	0.224	0.036	0.036
DB04185	Norvaline	-0.939	-0.795	-0.321	-0.9	-1.123	0.061	0.207	0.378	0.055	0.014
DB05676	Apremilast	-0.937	-0.959	-2.067	-0.395	-0.269	0.142	0.13	0.013	0.245	0.31
DB01428	Oxybenzone	-0.935	0.18	-1.425	-2.7	-1.152	0.093	0.499	0.063	0.009	0.066
DB01524	Androstenediol	-0.935	-0.084	-1.541	-2.265	-1.602	0.077	0.333	0.045	0.018	0.033
DB07402	Azapropazone	-0.935	-0.856	0.198	-1.383	-1.087	0.031	0.125	0.62	0.001	0.012
DB01058	Praziquantel	-0.934	-0.858	-0.169	-0.928	-1.168	0.036	0.15	0.487	0.034	0.009
DB02703	Fusidic acid	-0.934	-0.858	-0.169	-0.928	-1.168	0.036	0.15	0.487	0.034	0.009
DB02950	Hymenialdisine	-0.934	-0.492	-0.62	-1.841	-1.015	0.087	0.243	0.212	0.019	0.08
DB07220	N-[5-(1,1-DIOXIDOISOTHIAZOLIDIN-2-YL)-1H-INDAZOL-3-YL]-2-(4-PIPERIDIN-1-YLPHENYL)ACETAMIDE	-0.934	-0.492	-0.62	-1.452	-1.256	0.087	0.243	0.212	0.046	0.051
DB08880	Teriflunomide	-0.934	-0.858	-0.611	-0.928	-1.075	0.036	0.15	0.241	0.034	0.022
DB08299	4-[8-(3-nitrophenyl)-1,7-naphthyridin-6-yl]benzoic acid	-0.926	-0.54	1.064	-1.096	-0.758	0.076	0.218	0.784	0.07	0.124
DB00923	Ceforanide	-0.925	-0.737	-0.778	-0.943	-1.07	0.113	0.317	0.321	0.044	0.05
DB00291	Chlorambucil	-0.922	0.091	-1.057	-2.067	-1.657	0.099	0.444	0.083	0.015	0.038
DB00351	Megestrol acetate	-0.919	-0.33	1.022	-1.357	-1.217	0.151	0.316	0.76	0.056	0.08
DB00216	Eletriptan	-0.917	-1.136	1.017	1.165	-0.123	0.123	0.088	0.767	0.832	0.366
DB01759	Kojic acid	-0.917	-0.824	-0.469	-1.03	-1.149	0.11	0.266	0.323	0.028	0.015
DB00601	Linezolid	-0.916	-0.738	-0.309	-0.894	-1.217	0.043	0.264	0.396	0.038	0.003
DB01247	Isocarboxazid	-0.916	-0.738	-0.309	-0.894	-1.217	0.043	0.264	0.396	0.038	0.003
DB01626	Pargyline	-0.916	-0.738	-0.309	-0.894	-1.217	0.043	0.264	0.396	0.038	0.003
DB02095	Isatin	-0.916	-0.738	-0.309	-0.894	-1.217	0.043	0.264	0.396	0.038	0.003
DB02211	N-Methyl-N-Propargyl-1(R)-Aminoindan	-0.916	-0.738	-0.309	-0.894	-1.217	0.043	0.264	0.396	0.038	0.003
DB04307	5-Hydroxy-N-Propargyl-1(R)-Aminoindan	-0.916	-0.738	-0.309	-0.894	-1.217	0.043	0.264	0.396	0.038	0.003
DB04818	Iproniazid	-0.916	-0.738	-0.309	-0.894	-1.217	0.043	0.264	0.396	0.038	0.003
DB09245	Toloxatone	-0.916	-0.738	-0.309	-0.894	-1.217	0.043	0.264	0.396	0.038	0.003
DB09248	Mebanazine	-0.916	-0.738	-0.309	-0.894	-1.217	0.043	0.264	0.396	0.038	0.003
DB09250	Pheniprazine	-0.916	-0.738	-0.309	-0.894	-1.217	0.043	0.264	0.396	0.038	0.003
DB08819	Tafluprost	-0.915	-0.856	-0.307	-1	-1.227	0.096	0.278	0.427	0.041	0.013
DB00428	Streptozocin	-0.914	-0.795	0.583	-1.198	-0.879	0.011	0.068	0.688	0.023	0.044
DB01407	Clenbuterol	-0.914	-0.567	-3.435	-0.126	-1.497	0.154	0.239	0	0.37	0.046
DB06243	Eflornithine	-0.911	-0.728	-0.071	-0.865	-1.193	0.064	0.286	0.494	0.045	0.005
DB00444	Teniposide	-0.909	-0.337	-0.771	-1.197	-0.267	0.151	0.311	0.101	0.081	0.328
DB01012	Cinacalcet	-0.906	-0.767	-0.022	-0.778	-1.176	0.088	0.271	0.549	0.164	0.008
DB09074	Olaparib	-0.906	-0.379	-1.351	-2.414	1.703	0.151	0.298	0.037	0.003	0.934
DB04474	1-Anilino-8-Naphthalene Sulfonate	-0.905	-1.277	1.627	0.69	-0.203	0.106	0.052	0.889	0.515	0.344
DB01795	Phenyl Boronic Acid	-0.904	-0.153	1.696	-2.019	-1.377	0.093	0.378	0.916	0.027	0.038
DB01324	Polythiazide	-0.903	-0.743	-0.169	-0.928	-1.262	0.054	0.302	0.487	0.034	0.003
DB09345	Pramocaine	-0.903	-0.8	-0.169	-0.928	-1.168	0.054	0.23	0.487	0.034	0.009
DB07506	L-BENZYLSUCCINIC ACID	-0.902	-0.741	-0.416	-0.592	-1.158	0.012	0.151	0.298	0.187	0.002
DB01157	Trimetrexate	-0.901	-0.609	1.383	0.422	-0.906	0.154	0.213	0.861	0.524	0.146
DB04555	Cytidine-5'-Diphosphate	-0.9	-0.632	-2.752	-0.364	-0.633	0.011	0.146	0	0.186	0.157
DB00263	Sulfisoxazole	-0.898	-0.855	-0.049	-0.814	-1.115	0.046	0.159	0.497	0.068	0.011
DB01230	Pemoline	-0.898	-0.796	-0.238	-0.955	-1.11	0.119	0.275	0.461	0.043	0.029
DB01463	Fencamfamin	-0.898	-0.796	-0.238	-0.955	-1.11	0.119	0.275	0.461	0.043	0.029
DB06147	Sulfathiazole	-0.898	-0.855	-0.049	-0.814	-1.115	0.046	0.159	0.497	0.068	0.011

DB00082	Pegvisomant	-0.895	-0.845	-0.275	-0.54	-1.158	0.009	0.053	0.356	0.162	0.005
DB09098	Somatrem	-0.895	-0.845	-0.275	-0.54	-1.158	0.009	0.053	0.356	0.162	0.005
DB00730	Thiabendazole	-0.893	-0.268	1.715	-1.175	-0.888	0.164	0.331	0.945	0.087	0.134
DB03380	L-tyrosinamide	-0.893	-0.709	-1.469	-0.643	-1.171	0.005	0.158	0.002	0.116	0
DB01367	Rasagiline	-0.89	-0.621	-0.645	-0.507	-0.976	0.064	0.194	0.086	0.199	0.083
DB03397	Uridine-Diphosphate-N-Acetylglucosamine	-0.884	-0.822	-0.038	-0.733	-0.955	0.031	0.091	0.49	0.076	0.025
DB02827	7-(1,1-Dioxo-1h-Benzo[D]Isothiazol-3-Yloxymethyl)-2-(Oxalyl-Amino)-4,7-Dihydro-5h-Thieno[2,3-C]Pyran-3-Carboxylic Acid	-0.882	-0.28	-0.085	-0.103	-1.649	0.071	0.346	0.131	0.307	0.007
DB03670	2-(Oxalyl-Amino)-4,5,6,7-Tetrahydro-Thieno[2,3-C]Pyridine-3-Carboxylic Acid	-0.882	-0.28	-0.085	-0.103	-1.649	0.071	0.346	0.131	0.307	0.007
DB04204	[(4-{4-[4-(Difluoro-Phosphono-Methyl)-Phenyl]-Butyl}-Phenyl)-Difluoro-Methyl]-Phosphonic Acid	-0.882	-0.28	-0.085	-0.103	-1.649	0.071	0.346	0.131	0.307	0.007
DB07130	4-BROMO-3-(CARBOXYMETHOXY)-5-PHENYLTHIOPHENE-2-CARBOXYLIC ACID	-0.882	-0.28	-0.085	-0.103	-1.649	0.071	0.346	0.131	0.307	0.007
DB08003	ISOTHIAZOLIDINONE ANALOG	-0.882	-0.28	-0.085	-0.103	-1.649	0.071	0.346	0.131	0.307	0.007
DB13144	Lenograstim	-0.877	-0.818	0.074	-1.308	-0.988	0.039	0.125	0.565	0.006	0.025
DB01281	Abatacept	-0.876	-0.61	-0.595	-1.317	-1.053	0.052	0.369	0.189	0	0.016
DB06681	Belatacept	-0.876	-0.61	-0.595	-1.317	-1.053	0.052	0.369	0.189	0	0.016
DB09046	Metreleptin	-0.871	-0.815	-0.388	-0.655	-0.932	0.026	0.106	0.29	0.119	0.036
DB01752	S-adenosyl-L-homocysteine	-0.868	-1.551	0.796	1.116	0	0.15	0.044	0.677	0.75	0.402
DB01168	Procarbazine	-0.866	-0.644	0.02	-0.616	-1.194	0.034	0.232	0.53	0.204	0.007
DB05630	Sodium stibogluconate	-0.866	-0.275	0.425	-3.18	-1.415	0.019	0.401	0.585	0	0.002
DB04447	1,4-Dithiothreitol	-0.864	-1.678	-0.048	0.709	0.596	0.157	0.032	0.264	0.71	0.648
DB09075	Edoxaban	-0.863	-0.79	-0.049	-0.814	-1.115	0.081	0.229	0.497	0.068	0.011
DB00354	Buclizine	-0.862	-0.795	-0.398	-0.967	-1.06	0.139	0.281	0.373	0.04	0.02
DB00443	Betamethasone	-0.861	-0.16	-1.11	-1.437	-0.765	0.134	0.336	0.091	0.061	0.149
DB01392	Yohimbine	-0.859	-0.923	2.846	0.124	-0.873	0.176	0.149	0.998	0.465	0.155
DB06729	Sulfaphenazole	-0.857	0.196	-0.307	-1.458	-1.156	0.123	0.515	0.307	0.047	0.069
DB01954	Rolipram	-0.849	-0.708	1.008	-1.205	-0.818	0.024	0.13	0.8	0.04	0.089
DB01959	3,5-Dimethyl-1-(3-Nitrophenyl)-1h-Pyrazole-4-Carboxylic Acid Ethyl Ester	-0.849	-0.708	1.008	-1.205	-0.818	0.024	0.13	0.8	0.04	0.089
DB03849	Cilomilast	-0.849	-0.708	1.008	-1.205	-0.818	0.024	0.13	0.8	0.04	0.089
DB02644	N-Omega-Propyl-L-Arginine	-0.848	0.312	0.916	-0.717	-2.622	0.15	0.584	0.693	0.152	0.001
DB03449	N-(4-(2-((3-Chlorophenylmethyl)Amino)Ethyl)Phenyl)-2-Thiophecarboxamidine	-0.848	0.312	0.916	-0.717	-2.622	0.15	0.584	0.693	0.152	0.001
DB07002	4-({4-[(4-methoxypyridin-2-yl)amino]piperidin-1-yl}carbonyl)benzonitrile	-0.848	0.312	0.916	-0.717	-2.622	0.15	0.584	0.693	0.152	0.001
DB08750	1-[4-(AMINOMETHYL)BENZOYL]-5'-FLUORO-1'H-SPIRO[PIPERIDINE-4,2'-QUINAZOLIN]-4'-AMINE	-0.848	0.312	0.916	-0.717	-2.622	0.15	0.584	0.693	0.152	0.001
DB01164	Calcium Chloride	-0.847	-0.623	-0.464	-0.762	-1.024	0.068	0.314	0.269	0.071	0.014
DB00019	Pegfilgrastim	-0.843	-0.778	0.176	-1.312	-0.669	0.035	0.106	0.484	0.031	0.146
DB00072	Trastuzumab	-0.843	-0.575	0.706	-0.455	-0.566	0.098	0.195	0.654	0.254	0.21
DB00099	Filgrastim	-0.843	-0.778	0.176	-1.312	-0.669	0.035	0.106	0.484	0.031	0.146
DB00626	Bacitracin	-0.843	-0.109	0.847	0.396	-1.757	0.122	0.352	0.519	0.577	0.019
DB09030	Vorapaxar	-0.843	-0.863	0.079	-0.677	-0.821	0.064	0.095	0.554	0.106	0.081
DB04105	N-Heptylformamide	-0.842	-0.658	0.048	-0.824	-1.148	0.054	0.241	0.541	0.053	0.005
DB00500	Tolmetin	-0.84	-0.281	1.076	-0.696	-1.195	0.159	0.324	0.763	0.131	0.082
DB09477	Enalaprilat	-0.839	-0.767	0.106	-0.647	-0.866	0.105	0.252	0.592	0.237	0.1
DB09105	Asfotase alfa	-0.838	-0.711	0.041	-0.7	-1.032	0.053	0.18	0.569	0.098	0.012

DB00962	Zaleplon	-0.834	-0.862	0.455	-0.695	-0.851	0.047	0.064	0.705	0.088	0.066
DB02383	Tolrestat	-0.833	-0.889	0.934	-1.268	-1.326	0.038	0.06	0.816	0.028	0.004
DB02712	Sorbinil	-0.833	-0.889	0.934	-1.268	-1.326	0.038	0.06	0.816	0.028	0.004
DB04126	N-[1-Hydroxycarboxyethyl-Carbonyl]Leucylamino-2-Methyl-Butane	-0.833	-0.112	0.3	-2.863	-1.022	0.064	0.476	0.513	0	0.047
DB07450	(R)-minalrestat	-0.833	-0.889	0.934	-1.268	-1.326	0.038	0.06	0.816	0.028	0.004
DB08772	3,4-DIHYDRO-4-OXO-3-((5-TRIFLUOROMETHYL-2-BENZOTHIAZOLYL)METHYL)-1-PHTHALAZINE ACETIC ACID	-0.833	-0.889	0.934	-1.268	-1.326	0.038	0.06	0.816	0.028	0.004
DB02132	Zenarestat	-0.832	-0.771	0.074	-0.662	-1.099	0.029	0.095	0.545	0.099	0.017
DB06837	(2R)-N~4~-hydroxy-2-(3-hydroxybenzyl)-N~1~-[(1S,2R)-2-hydroxy-2,3-dihydro-1H-inden-1-yl]butanediamide	-0.832	-0.712	-0.269	-0.872	-1.058	0.098	0.274	0.421	0.043	0.019
DB00783	Estradiol	-0.829	-0.549	-0.531	-0.01	-0.929	0.095	0.202	0.236	0.454	0.119
DB00928	Azacitidine	-0.828	0.173	-2.263	-2.229	0.043	0.113	0.521	0.013	0.015	0.463
DB01284	Tetracosactide	-0.827	-0.57	-0.604	-1.342	-0.946	0.116	0.389	0.203	0.001	0.052
DB02458	S-(2,4-dinitrophenyl)glutathione	-0.827	-0.783	-0.307	-0.895	-1.002	0.139	0.235	0.403	0.046	0.04
DB02643	N-Dodecyl-N,N-Dimethyl-3-Ammonio-1-Propanesulfonate	-0.826	-0.657	-0.493	-0.629	-1.09	0.034	0.193	0.24	0.103	0.01
DB02196	Uridine-Diphosphate-N-Acetylgalactosamine	-0.824	-0.744	-0.673	-0.714	-0.912	0.035	0.115	0.137	0.072	0.03
DB04630	Aldosterone	-0.823	-0.641	0.351	0.41	-1.801	0.11	0.186	0.501	0.424	0.009
DB04480	3-(4-Fluorophenyl)-2-(6-Methylpyridin-2-Yl)-5,6-Dihydro-4h-Pyrrolo[1,2-B]Pyrazole	-0.82	-0.262	0.168	0.402	-1.114	0.149	0.342	0.194	0.523	0.087
DB00987	Cytarabine	-0.818	0.575	-0.659	-1.866	-0.932	0.169	0.772	0.104	0.028	0.132
DB01440	gamma-Hydroxybutyric acid	-0.818	-0.661	-0.212	-0.854	-1.017	0.107	0.306	0.435	0.052	0.025
DB01861	Uridine diphosphate glucose	-0.818	-0.661	-0.212	-0.854	-1.017	0.107	0.306	0.435	0.052	0.025
DB03056	4-Piperidino-Piperidine	-0.818	-0.677	0.116	-0.894	-1.017	0.145	0.338	0.575	0.038	0.03
DB02056	Prostaglandin D2	-0.815	-0.691	-0.338	-0.817	-1.029	0.04	0.166	0.32	0.051	0.01
DB00691	Moexipril	-0.813	-0.779	0.325	-0.52	-0.92	0.107	0.203	0.653	0.274	0.052
DB00977	Ethinylestradiol	-0.813	0.085	-1.188	-1.464	-1.232	0.128	0.447	0.084	0.057	0.061
DB01382	Glymidine	-0.812	-0.663	0.21	-0.618	-0.956	0.092	0.301	0.623	0.225	0.044
DB01643	Thymidine monophosphate	-0.811	0.342	0.968	-3.166	-1.405	0.133	0.62	0.733	0	0.044
DB07711	(2S,3R)-3-(6-amino-9H-purin-9-yl)nonan-2-ol	-0.809	-0.65	-1.525	-1.343	-1.112	0.064	0.217	0.001	0.002	0.005
DB03059	Acetoacetyl-CoA	-0.808	-0.53	0.074	-0.662	-1.211	0.06	0.328	0.556	0.088	0.003
DB03680	Tartronate	-0.805	-0.707	-0.318	-0.87	-0.964	0.152	0.316	0.395	0.059	0.043
DB04454	Alpha-Aminobutyric Acid	-0.805	-0.754	-0.043	-0.679	-0.989	0.063	0.122	0.478	0.095	0.025
DB00086	Streptokinase	-0.804	-0.314	0.586	-0.859	-1.161	0.083	0.335	0.598	0.086	0.051
DB02984	4-[3-Methylsulfanylanilino]-6,7-Dimethoxyquinazoline	-0.804	-0.701	0.89	-0.208	-1.678	0.134	0.145	0.542	0.311	0.029
DB02580	Pentaglyme	-0.803	-1.186	1.894	-0.245	0.111	0.167	0.088	0.935	0.246	0.44
DB06688	Sipuleucel-T	-0.802	-0.746	-0.433	-0.725	-0.875	0.084	0.187	0.313	0.089	0.061
DB00042	Botulinum Toxin Type B	-0.799	-0.194	0.34	0.619	-1.98	0.12	0.397	0.488	0.514	0.005
DB02407	6-O-Cyclohexylmethyl Guanine	-0.798	-0.184	-1.349	-1.396	-1.012	0.106	0.343	0.059	0.055	0.08
DB06944	N-(3-cyclopropyl-1H-pyrazol-5-yl)-2-(2-naphthyl)acetamide	-0.798	-0.184	-1.349	-1.396	-1.012	0.106	0.343	0.059	0.055	0.08
DB08572	4-{[4-AMINO-6-(CYCLOHEXYLMETHOXY)-5-NITROSOPYRIMIDIN-2-YL]AMINO}BENZAMIDE	-0.798	-0.184	-1.349	-1.396	-1.012	0.106	0.343	0.059	0.055	0.08
DB03209	Oteracil	-0.796	-0.672	0.148	-0.655	-1.05	0.082	0.227	0.596	0.119	0.018
DB05258	Interferon alfa	-0.796	-0.706	-0.17	-0.455	-0.943	0.025	0.128	0.384	0.167	0.02
DB04377	Meglutol	-0.795	-0.74	0.16	-0.622	-0.885	0.052	0.11	0.609	0.122	0.033
DB06706	Isometheptene	-0.794	-0.721	0.112	-0.895	-1.002	0.18	0.307	0.58	0.046	0.04

DB00837	Progabide	-0.792	-0.774	0.686	-0.624	-0.932	0.044	0.09	0.751	0.108	0.03
DB00998	Frovatriptan	-0.792	-0.709	0.272	-0.443	-0.849	0.061	0.145	0.618	0.265	0.071
DB01586	Ursodeoxycholic acid	-0.792	-0.465	-0.742	-0.468	-1.309	0.066	0.268	0.098	0.164	0.013
DB01080	Vigabatrin	-0.791	-0.781	0.702	-0.647	-0.939	0.033	0.085	0.749	0.093	0.034
DB01590	Everolimus	-0.791	-0.334	0.076	0.381	-0.44	0.175	0.315	0.175	0.505	0.253
DB02062	N-[3-[(1-Aminoethyl)(Hydroxy)Phosphoryl]-2-(1,1'-Biphenyl-4-Ylmethyl)Propanoyl]Alanine	-0.791	-0.585	-2.304	0.737	-0.225	0.089	0.205	0.002	0.579	0.356
DB02210	Hexane-1,6-Diol	-0.791	-0.921	0.076	-0.713	-0.231	0.178	0.135	0.255	0.164	0.327
DB00787	Acyclovir	-0.79	-0.589	-0.859	-0.505	-1.231	0.027	0.208	0.017	0.157	0
DB00841	Dobutamine	-0.789	-0.134	0.94	-0.852	-1.223	0.134	0.362	0.768	0.127	0.083
DB03385	4-Methylimidazole	-0.789	-0.629	-0.108	-1.265	-1.113	0.082	0.266	0.464	0.007	0.009
DB03837	Morpholine-4-Carboxylic Acid (1-(3-Benzenesulfonyl-1-Phenethylallylcarbamoyl)-3-Methylbutyl)-Amide	-0.789	-0.324	1.235	-1.287	0.012	0.144	0.326	0.799	0.087	0.411
DB00796	Candesartan cilexetil	-0.788	-0.031	0.245	-0.077	-1.092	0.162	0.396	0.455	0.386	0.08
DB03247	Flavin mononucleotide	-0.787	-0.609	-0.924	-0.119	-0.571	0.196	0.22	0.096	0.335	0.22
DB08870	Brentuximab vedotin	-0.787	-0.551	-0.039	-0.745	-1.066	0.175	0.421	0.523	0.085	0.023
DB09324	Sulbactam	-0.787	-0.621	-0.039	-0.745	-0.959	0.175	0.345	0.523	0.085	0.064
DB00178	Ramipril	-0.786	-0.704	0.404	-0.339	-0.815	0.108	0.227	0.681	0.425	0.078
DB00211	Midodrine	-0.786	-1.218	-0.593	-0.86	-0.629	0.118	0.091	0.221	0.128	0.146
DB06757	Manganese	-0.784	-0.214	0.007	-0.53	-2.159	0.191	0.351	0.152	0.2	0.008
DB06756	Glycine betaine	-0.783	-0.732	-0.446	-0.598	-0.685	0.037	0.185	0.068	0.164	0.132
DB00074	Basiliximab	-0.782	-1.07	0.011	-0.003	0.379	0.184	0.115	0.341	0.413	0.53
DB00551	Acetohydroxamic acid	-0.782	-0.625	0.024	-1.307	-1.058	0.06	0.242	0.529	0.005	0.016
DB03643	CRA_1144	-0.782	-0.705	-0.337	-0.548	-0.912	0.039	0.124	0.285	0.12	0.05
DB00008	Peginterferon alfa-2a	-0.78	-0.476	-0.638	0.05	-0.819	0.064	0.242	0.037	0.3	0.096
DB00011	Interferon alfa-n1	-0.78	-0.476	-0.638	0.05	-0.819	0.064	0.242	0.037	0.3	0.096
DB00018	Interferon alfa-n3	-0.78	-0.476	-0.638	0.05	-0.819	0.064	0.242	0.037	0.3	0.096
DB00022	Peginterferon alfa-2b	-0.78	-0.476	-0.638	0.05	-0.819	0.064	0.242	0.037	0.3	0.096
DB00034	Interferon Alfa-2a, Recombinant	-0.78	-0.476	-0.638	0.05	-0.819	0.064	0.242	0.037	0.3	0.096
DB00060	Interferon beta-1a	-0.78	-0.476	-0.638	0.05	-0.819	0.064	0.242	0.037	0.3	0.096
DB00068	Interferon beta-1b	-0.78	-0.476	-0.638	0.05	-0.819	0.064	0.242	0.037	0.3	0.096
DB00069	Interferon alfacon-1	-0.78	-0.476	-0.638	0.05	-0.819	0.064	0.242	0.037	0.3	0.096
DB00105	Interferon alfa-2b	-0.78	-0.476	-0.638	0.05	-0.819	0.064	0.242	0.037	0.3	0.096
DB02139	(2e)-N-Allyl-4-{[3-(4-Bromophenyl)-5-Fluoro-1-Methyl-1h-Indazol-6-Yl]Oxy}-N-Methyl-2-Buten-1-Amine	-0.78	0.006	-0.886	-0.891	-1.379	0.163	0.48	0.152	0.119	0.035
DB02339	Allyl-{6-[3-(4-Bromo-Phenyl)-Benzofuran-6-Yloxy]-Hexyl-}-Methyl-Amin	-0.78	0.006	-0.886	-0.891	-1.379	0.163	0.48	0.152	0.119	0.035
DB02544	N-(6-{[3-(4-Bromophenyl)-1,2-Benzisothiazol-6-Yl]Oxy}Hexyl)-N-Methylprop-2-En-1-Amine	-0.78	0.006	-0.886	-0.891	-1.379	0.163	0.48	0.152	0.119	0.035
DB03234	(4'-{[Allyl(Methyl)Amino]Methyl}-1,1'-Biphenyl-4-Yl)(4-Bromophenyl)Methanone	-0.78	0.006	-0.886	-0.891	-1.379	0.163	0.48	0.152	0.119	0.035
DB03748	Methyl-[4-(4-Piperidine-1-Ylmethyl-Phenyl)-Cyclohexyl]-Carbaminic Acid-(4-Chlorophenyl)-Ester	-0.78	0.006	-0.886	-0.891	-1.379	0.163	0.48	0.152	0.119	0.035
DB03771	Allyl-{4-[3-(4-Bromo-Phenyl)-Benzofuran-6-Yloxy]-but-2-Enyl}-Methyl-Amine	-0.78	0.006	-0.886	-0.891	-1.379	0.163	0.48	0.152	0.119	0.035
DB03888	N-Allyl-6-{[3-(4-bromophenyl)-1-methyl-1H-indazol-6-yl]oxy}-N-methyl-1-hexanamine	-0.78	0.006	-0.886	-0.891	-1.379	0.163	0.48	0.152	0.119	0.035

DB08458	(4-BROMOPHENYL)[4-({(2E)-4-[CYCLOPROPYL(METHYL)AMINO]BUT-2-ENYL}OXY)PHENYL]METHANONE	-0.78	0.006	-0.886	-0.891	-1.379	0.163	0.48	0.152	0.119	0.035
DB04400	L-erythro-7,8-dihydrobiopterin	-0.779	0.353	1.332	-0.734	-2.771	0.152	0.627	0.837	0.157	0.001
DB02315	Cyclic GMP	-0.775	-0.114	0.271	-1.789	-1.28	0.071	0.459	0.482	0.009	0.027
DB00489	Sotalol	-0.774	-0.061	-1.829	0.343	-1.451	0.181	0.441	0.02	0.53	0.044
DB06043	Olaratumab	-0.771	-0.632	0.466	-0.303	-1.295	0.042	0.182	0.701	0.239	0
DB00672	Chlorpropamide	-0.768	0.067	-1.06	-1.828	-1.141	0.129	0.506	0.075	0.024	0.064
DB04493	Fructose-6-Phosphate	-0.767	-0.644	0.544	-0.424	-0.434	0.196	0.216	0.555	0.197	0.252
DB06650	Ofatumumab	-0.767	-0.694	-0.113	-0.871	-0.993	0.213	0.335	0.492	0.051	0.043
DB08935	Obinutuzumab	-0.767	-0.694	-0.113	-0.871	-0.993	0.213	0.335	0.492	0.051	0.043
DB02910	Octanoyl-Coenzyme A	-0.766	-0.73	0.179	-0.596	-0.886	0.056	0.112	0.586	0.106	0.039
DB12332	Rucaparib	-0.766	-0.292	-1.67	-1.673	1.229	0.187	0.345	0.032	0.041	0.86
DB08009	5-[(E)-(5-CHLORO-2-OXO-1,2-DIHYDRO-3H-INDOL-3-YLIDENE)METHYL]-N-[2-(DIETHYLAMINO)ETHYL]-2,4-DIMETHYL-1H-PYRROLE-3-CARBOXAMIDE	-0.765	-0.309	-0.68	0.522	-1.633	0.146	0.298	0.135	0.64	0.034
DB08605	6-METHYL-2(PROPANE-1-SULFONYL)-2H-THIENO[3,2-D][1,2,3]DIAZABORININ-1-OL	-0.764	-0.598	0.934	-1.309	-1.159	0.059	0.201	0.814	0.016	0.003
DB01436	Alfacalcidol	-0.763	0.164	0.8	-0.742	-0.473	0.185	0.537	0.656	0.158	0.251
DB02363	2'-Monophosphoadenosine-5'-Diphosphate	-0.762	-0.64	0.649	-0.606	-1.215	0.048	0.174	0.746	0.113	0.004
DB02854	Aetiocholanolone	-0.76	-1.051	0.22	0.762	-0.925	0.19	0.108	0.427	0.549	0.122
DB00179	Masoprocol	-0.758	-0.395	-2.312	-0.686	0.057	0.121	0.243	0.011	0.184	0.454
DB02613	Decylamine-N,N-Dimethyl-N-Oxide	-0.758	-0.689	-0.157	-0.52	-0.806	0.083	0.2	0.44	0.261	0.091
DB08936	Chlorcyclizine	-0.758	-0.698	-0.54	-0.552	-0.631	0.076	0.138	0.078	0.19	0.184
DB00871	Terbutaline	-0.757	0.842	-1.747	-0.92	-1.883	0.185	0.762	0.025	0.108	0.017
DB00350	Minoxidil	-0.756	-0.626	1.147	-1.324	-0.865	0.057	0.177	0.868	0.008	0.039
DB00487	Pefloxacin	-0.755	-0.469	0.407	-1.08	-0.308	0.147	0.26	0.504	0.091	0.333
DB04325	Phenethylamine	-0.755	-0.758	0.111	-0.612	-0.94	0.057	0.094	0.563	0.118	0.044
DB04360	Benzo[B]Thiophene-2-Boronic Acid	-0.755	-0.548	0.341	-1.334	-0.889	0.047	0.243	0.633	0.021	0.049
DB02010	Staurosporine	-0.747	-0.83	0.025	-1.146	-0.168	0.11	0.131	0.428	0.094	0.375
DB02767	3-Hydroxy-Myristic Acid	-0.747	-0.552	0.586	0.307	-1.196	0.083	0.224	0.688	0.593	0.024
DB04654	4-PIPERIDIN-4-YLBUTANAL	-0.745	-0.87	0.428	-0.4	-0.659	0.068	0.031	0.688	0.184	0.141
DB08995	Diosmin	-0.744	-0.792	-0.83	-0.143	-0.694	0.178	0.156	0.168	0.347	0.171
DB00357	Aminoglutethimide	-0.743	-0.495	0.35	-0.705	-1.238	0.065	0.348	0.642	0.067	0.002
DB00855	Aminolevulinic acid	-0.74	-0.555	0.034	-0.762	-0.924	0.201	0.405	0.551	0.071	0.048
DB02115	Daidzin	-0.74	-0.392	0.183	0.014	-1.359	0.086	0.309	0.464	0.27	0.009
DB03904	Urea	-0.738	-0.83	0.4	-2.535	-1.16	0.143	0.123	0.365	0.007	0.069
DB00146	Calcifediol	-0.737	-0.265	0.478	0.345	-1.314	0.079	0.371	0.593	0.465	0.005
DB00910	Paricalcitol	-0.737	-0.265	0.478	0.345	-1.314	0.079	0.371	0.593	0.465	0.005
DB01070	Dihydrotachysterol	-0.737	-0.265	0.478	0.345	-1.314	0.079	0.371	0.593	0.465	0.005
DB04014	Alsterpaullone	-0.737	-0.321	-0.167	-0.996	-1.064	0.121	0.265	0.353	0.103	0.091
DB05295	Eldecalcitol	-0.737	-0.265	0.478	0.345	-1.314	0.079	0.371	0.593	0.465	0.005
DB06410	Doxercalciferol	-0.737	-0.265	0.478	0.345	-1.314	0.079	0.371	0.593	0.465	0.005
DB00315	Zolmitriptan	-0.736	-0.73	1.233	0.195	-0.552	0.071	0.108	0.851	0.557	0.188
DB00952	Naratriptan	-0.736	-0.73	1.233	0.195	-0.552	0.071	0.108	0.851	0.557	0.188
DB07637	Dibromotyrosine	-0.736	-0.588	0.156	-0.702	-1.07	0.079	0.283	0.598	0.08	0.007
DB01031	Ethinamate	-0.734	-0.542	0.42	-1.315	-1.059	0.096	0.332	0.684	0.006	0.009
DB01650	trans-2-hydroxycinnamic acid	-0.734	-0.542	0.42	-1.315	-1.059	0.096	0.332	0.684	0.006	0.009
DB02362	Aminobenzoic acid	-0.734	-0.542	0.42	-1.315	-1.059	0.096	0.332	0.684	0.006	0.009
DB03728	p-Chlorobenzoic acid	-0.734	-0.542	0.42	-1.315	-1.059	0.096	0.332	0.684	0.006	0.009
DB04500	Acedoben	-0.734	-0.542	0.42	-1.315	-1.059	0.096	0.332	0.684	0.006	0.009
DB06263	Amrubicin	-0.734	-0.671	0.246	-1.332	-0.39	0.068	0.156	0.529	0.02	0.321

DB08155	N-{2-[4-(AMINOSULFONYL)PHENYL]ETHYL}ACETAMIDE	-0.734	-0.542	0.42	-1.315	-1.059	0.096	0.332	0.684	0.006	0.009
DB08156	3-[4-(AMINOSULFONYL)PHENYL]PROPANOIC ACID	-0.734	-0.542	0.42	-1.315	-1.059	0.096	0.332	0.684	0.006	0.009
DB08157	ETHYL 3-[4-(AMINOSULFONYL)PHENYL]PROPANOATE	-0.734	-0.542	0.42	-1.315	-1.059	0.096	0.332	0.684	0.006	0.009
DB08748	4-(DIMETHYLAMINO)BENZOIC ACID	-0.734	-0.542	0.42	-1.315	-1.059	0.096	0.332	0.684	0.006	0.009
DB09047	Finafloxacin	-0.734	-0.671	0.246	-1.332	-0.39	0.068	0.156	0.529	0.02	0.321
DB09353	Octasulfur	-0.734	-0.542	0.42	-1.315	-1.059	0.096	0.332	0.684	0.006	0.009
DB09472	Sodium sulfate	-0.734	-0.542	0.42	-1.315	-1.059	0.096	0.332	0.684	0.006	0.009
DB00147	Pyridoxal	-0.732	-0.57	0.396	-0.489	-1.044	0.157	0.347	0.672	0.301	0.019
DB01020	Isosorbide Mononitrate	-0.732	-0.597	-0.624	-0.793	-0.917	0.176	0.395	0.195	0.064	0.05
DB07688	4-{[5-(CYCLOHEXYLOXY)[1,2,4]TRIAZOLO[1,5-A]PYRIMIDIN-7-YL]AMINO}BENZENESULFONAMIDE	-0.731	-0.244	-0.872	-0.787	-0.67	0.144	0.303	0.128	0.148	0.154
DB06751	Drotaverine	-0.73	-0.689	0.722	-0.151	-1.017	0.078	0.143	0.709	0.22	0.054
DB00365	Grepafloxacin	-0.729	-0.576	0.041	-0.7	-0.929	0.156	0.345	0.569	0.098	0.028
DB00976	Telithromycin	-0.729	-0.576	0.041	-0.7	-0.929	0.156	0.345	0.569	0.098	0.028
DB06708	Lumefantrine	-0.729	-0.576	0.041	-0.7	-0.929	0.156	0.345	0.569	0.098	0.028
DB08903	Bedaquiline	-0.729	-0.576	0.041	-0.7	-0.929	0.156	0.345	0.569	0.098	0.028
DB09102	Daclatasvir	-0.729	-0.576	0.041	-0.7	-0.929	0.156	0.345	0.569	0.098	0.028
DB09352	Hydroxyamphetamine	-0.729	-0.576	0.041	-0.7	-0.929	0.156	0.345	0.569	0.098	0.028
DB11614	Rupatadine	-0.729	-0.588	-0.075	-0.755	-0.913	0.188	0.339	0.495	0.073	0.05
DB00548	Azelaic acid	-0.728	-0.642	-0.114	-0.687	-0.975	0.189	0.327	0.482	0.157	0.035
DB01046	Lubiprostone	-0.727	-0.669	0.138	-0.672	-0.94	0.089	0.19	0.606	0.093	0.022
DB00181	Baclofen	-0.725	-0.173	-0.484	-1.796	-0.663	0.12	0.312	0.227	0.026	0.149
DB00192	Indecainide	-0.724	-0.656	0.108	-0.672	-1.02	0.146	0.266	0.569	0.103	0.03
DB00218	Moxifloxacin	-0.724	-0.429	0.417	-1.434	-0.415	0.136	0.273	0.567	0.036	0.264
DB00680	Moricizine	-0.724	-0.656	0.108	-0.672	-1.02	0.146	0.266	0.569	0.103	0.03
DB00776	Oxcarbazepine	-0.724	-0.656	0.108	-0.672	-1.02	0.146	0.266	0.569	0.103	0.03
DB00868	Benzonatate	-0.724	-0.656	0.108	-0.672	-1.02	0.146	0.266	0.569	0.103	0.03
DB00978	Lomefloxacin	-0.724	-0.429	0.417	-1.434	-0.415	0.136	0.273	0.567	0.036	0.264
DB01056	Tocainide	-0.724	-0.656	0.108	-0.672	-1.02	0.146	0.266	0.569	0.103	0.03
DB01320	Fosphenytoin	-0.724	-0.656	0.108	-0.672	-1.02	0.146	0.266	0.569	0.103	0.03
DB03808	Hexamidine	-0.724	-0.888	0.389	0.299	0.069	0.134	0.104	0.523	0.364	0.473
DB04576	Fleroxacin	-0.724	-0.429	0.417	-1.434	-0.415	0.136	0.273	0.567	0.036	0.264
DB08168	7-AMINO-4-METHYL-CHROMEN-2-ONE	-0.724	-0.995	0.537	-1.062	-0.03	0.197	0.135	0.52	0.109	0.409
DB00816	Orciprenaline	-0.723	-0.334	-1.814	0.143	-1.32	0.16	0.365	0.006	0.459	0.03
DB00901	Bitolterol	-0.723	-0.334	-1.814	0.143	-1.32	0.16	0.365	0.006	0.459	0.03
DB08066	N-[3-(1H-BENZIMIDAZOL-2-YL)-1H-PYRAZOL-4-YL]BENZAMIDE	-0.723	0.168	-0.757	-1.733	-0.946	0.147	0.489	0.155	0.025	0.096
DB13139	Levosalbutamol	-0.723	-0.334	-1.814	0.143	-1.32	0.16	0.365	0.006	0.459	0.03
DB02742	Kifunensine	-0.722	-0.55	-0.479	-0.68	-0.855	0.128	0.306	0.238	0.079	0.054
DB01005	Hydroxyurea	-0.719	-0.621	0.562	-0.35	-1.171	0.039	0.149	0.722	0.227	0.001
DB02110	Protoporphyrin Ix Containing Co	-0.718	-0.787	1.378	-0.998	-0.563	0.048	0.075	0.883	0.042	0.161
DB03413	Deoxyuridine-5'-Diphosphate	-0.718	-0.636	0.118	-0.687	-0.842	0.189	0.292	0.573	0.099	0.067
DB01656	Roflumilast	-0.717	-0.318	1.025	-0.866	-0.624	0.167	0.311	0.753	0.105	0.187
DB03606	(S)-Rolipram	-0.717	-0.318	1.025	-0.866	-0.624	0.167	0.311	0.753	0.105	0.187
DB04149	(R)-Rolipram	-0.717	-0.318	1.025	-0.866	-0.624	0.167	0.311	0.753	0.105	0.187
DB01011	Metyrapone	-0.715	-0.378	0.695	-0.154	-0.879	0.238	0.57	0.738	0.549	0.08
DB05314		-0.715	-0.048	1.766	0.714	-1.456	0.155	0.443	0.917	0.568	0.025

DB08094	(4-AMINO-2-{[1-(METHYLSULFONYL)PIPERIDIN-4-YL]AMINO}PYRIMIDIN-5-YL)(2,3-DIFLUORO-6-METHOXYPHENYL)METHANONE	-0.715	-0.832	0.161	0.135	-0.791	0.183	0.154	0.514	0.492	0.158
DB03559	Cyclohexylformamide	-0.714	-0.489	0.485	-0.45	-1	0.106	0.337	0.675	0.253	0.024
DB00615	Rifabutin	-0.713	-1.202	-0.866	-0.488	-0.124	0.126	0.071	0.101	0.218	0.332
DB09120	Zucapsaicin	-0.712	-0.582	0.055	-0.65	-0.97	0.123	0.291	0.561	0.082	0.029
DB00335	Atenolol	-0.711	0.188	-1.823	0.324	-1.74	0.187	0.537	0.014	0.509	0.021
DB02764		-0.711	-0.758	0.111	-0.612	-0.801	0.096	0.094	0.563	0.118	0.085
DB03976	Phosphorylisopropane	-0.711	-0.758	0.635	-0.275	-0.801	0.096	0.094	0.74	0.363	0.085
DB00872	Conivaptan	-0.71	-0.591	-0.489	-0.692	-0.825	0.097	0.239	0.233	0.075	0.069
DB06212	Tolvaptan	-0.71	-0.591	-0.489	-0.692	-0.825	0.097	0.239	0.233	0.075	0.069
DB07584	N-[2-(5-methyl-4H-1,2,4-triazol-3-yl)phenyl]-7H-pyrrolo[2,3-d]pyrimidin-4-amine	-0.71	-1.373	-0.359	-0.596	0.267	0.155	0.054	0.215	0.196	0.51
DB07585	5-(5-chloro-7H-pyrrolo[2,3-d]pyrimidin-4-yl)-4,5,6,7-tetrahydro-1H-imidazo[4,5-c]pyridine	-0.71	-1.373	-0.359	-0.596	0.267	0.155	0.054	0.215	0.196	0.51
DB07162	4-(3-amino-1H-indazol-5-yl)-N-tert-butylbenzenesulfonamide	-0.709	0.029	-0.995	0.238	-1.4	0.191	0.452	0.129	0.447	0.044
DB00558	Zanamivir	-0.707	-0.681	0.086	-0.633	-0.777	0.119	0.162	0.56	0.101	0.104
DB03262	Al-6619, [2h-Thieno[3,2-E]-1,2-Thiazine-6-Sulfonamide,2-(3-Hydroxyphenyl)-3-(4-Morpholinyl)-, 1,1-Dioxide]	-0.707	-0.512	0.604	-0.965	-1.053	0.119	0.333	0.732	0.064	0.011
DB03598	Al-6629, [2h-Thieno[3,2-E]-1,2-Thiazine-6-Sulfonamide,2-(3-Methoxyphenyl)-3-(4-Morpholinyl)-, 1,1-Dioxide]	-0.707	-0.512	0.604	-0.965	-1.053	0.119	0.333	0.732	0.064	0.011
DB08694	9-amino-5-(2-aminopyrimidin-4-yl)pyrido[3',2':4,5]pyrrolo[1,2-c]pyrimidin-4-ol	-0.706	0.436	-2.13	0.162	-1.166	0.196	0.631	0.008	0.416	0.072
DB02648	(3-Carboxy-2-(R)-Hydroxy-Propyl)-Trimethyl-Ammonium	-0.704	-0.495	0.075	-0.65	-1.074	0.149	0.391	0.552	0.084	0.008
DB09130	Copper	-0.703	-1.542	0.025	-0.122	-0.425	0.177	0.067	0.473	0.334	0.23
DB06809	Plerixafor	-0.7	-0.017	-2.535	-0.624	-0.363	0.15	0.443	0.002	0.151	0.266
DB00825	Levomenthol	-0.697	-0.442	-0.346	-0.718	-1.144	0.078	0.285	0.257	0.1	0.011
DB00140	Riboflavin	-0.696	-0.694	-0.381	-0.474	-0.325	0.151	0.156	0.261	0.236	0.271
DB02054	Gabaculine	-0.691	-0.328	1.465	-1.308	-1.588	0.11	0.336	0.887	0.043	0.01
DB06914	1-({2-[2-(4-CHLOROPHENYL)ETHYL]-1,3-DIOXOLAN-2-YL}METHYL)-1H-IMIDAZOLE	-0.691	-0.477	0.909	-0.123	-1.212	0.107	0.251	0.781	0.244	0.035
DB00533	Rofecoxib	-0.69	-0.623	1.474	-0.975	-1.112	0.189	0.221	0.864	0.083	0.079
DB04092	Apstatin	-0.689	-0.708	1.362	0.191	-0.876	0.144	0.152	0.849	0.331	0.105
DB09302	Alirocumab	-0.688	-0.456	0.138	-1.265	-0.94	0.138	0.463	0.606	0.006	0.022
DB09303	Evolocumab	-0.688	-0.456	0.138	-1.265	-0.94	0.138	0.463	0.606	0.006	0.022
DB00639	Butoconazole	-0.687	-0.726	-1.06	-1.002	-0.41	0.152	0.154	0.075	0.077	0.224
DB04545	Afegostat	-0.687	0.345	0.492	0.338	-2.463	0.153	0.701	0.583	0.372	0
DB00281	Lidocaine	-0.686	-0.13	1.587	1.187	-1.308	0.178	0.366	0.912	0.856	0.061
DB00320	Dihydroergotamine	-0.686	-0.65	2.645	0.652	-0.113	0.213	0.206	0.994	0.699	0.39
DB00012	Darbepoetin alfa	-0.684	-0.398	-0.082	-0.373	-1.092	0.105	0.415	0.383	0.192	0.011
DB00016	Erythropoietin	-0.684	-0.398	-0.082	-0.373	-1.092	0.105	0.415	0.383	0.192	0.011
DB00301	Flucloxacillin	-0.684	-0.47	-0.841	-0.54	-0.904	0.152	0.427	0.018	0.162	0.052
DB00560	Tigecycline	-0.684	-0.47	-0.841	-0.54	-0.904	0.152	0.427	0.018	0.162	0.052
DB04933	Eritoran	-0.684	-0.499	-0.082	-0.373	-1.092	0.105	0.319	0.383	0.192	0.011
DB08740	CYCLOHEXYLMETHYL-2,3-DIHYDROXY-5-METHYL-HEXYLAMIDE	-0.684	-0.701	-0.082	-1.191	-0.935	0.105	0.146	0.383	0.025	0.03
DB08894	Peginesatide	-0.684	-0.398	-0.082	-0.373	-1.092	0.105	0.415	0.383	0.192	0.011

DB08923		-0.684	-0.398	-0.082	-0.373	-1.092	0.105	0.415	0.383	0.192	0.011
DB09107	Methoxy polyethylene glycol-epoetin beta	-0.684	-0.398	-0.082	-0.373	-1.092	0.105	0.415	0.383	0.192	0.011
DB00282	Pamidronic acid	-0.682	-0.521	0.16	-0.622	-0.885	0.167	0.366	0.609	0.122	0.033
DB00710	Ibandronate	-0.682	-0.521	0.16	-0.622	-0.885	0.167	0.366	0.609	0.122	0.033
DB00861	Diflunisal	-0.682	-1.375	2.21	-1.274	-0.156	0.203	0.06	0.979	0.074	0.347
DB00884	Risedronic acid	-0.682	-0.521	0.16	-0.622	-0.885	0.167	0.366	0.609	0.122	0.033
DB06255	Incadronic acid	-0.682	-0.521	0.16	-0.622	-0.885	0.167	0.366	0.609	0.122	0.033
DB11620	Neridronic Acid	-0.682	-0.521	0.16	-0.622	-0.885	0.167	0.366	0.609	0.122	0.033
DB00196	Fluconazole	-0.681	-0.259	-0.093	-0.515	-0.789	0.176	0.259	0.298	0.205	0.129
DB03403	Cytidine-5'-Monophosphate	-0.681	0.178	-3.088	-0.134	-0.932	0.103	0.674	0	0.231	0.065
DB08910	Pomalidomide	-0.681	-0.752	-2.159	-0.002	-0.769	0.198	0.182	0.009	0.349	0.155
DB00026	Anakinra	-0.68	-0.562	0.118	-0.687	-0.842	0.251	0.368	0.573	0.099	0.067
DB00705	Delavirdine	-0.68	-0.572	1.131	0.055	-0.794	0.069	0.186	0.836	0.488	0.065
DB03316	1,4-Diethylene Dioxide	-0.68	-0.673	1.205	-1.125	-0.555	0.124	0.156	0.833	0.055	0.19
DB03861	(2r,3r,4s,5r)-2-Acetamido-3,4-Dihydroxy-5-Hydroxymethyl-Piperidinium	-0.68	-0.49	-0.596	-0.771	-0.905	0.18	0.394	0.165	0.068	0.068
DB01022	Phylloquinone	-0.679	-0.632	0.118	-0.643	-0.922	0.113	0.232	0.567	0.116	0.032
DB05266	Ibudilast	-0.679	0.147	1.181	-0.679	-1.102	0.197	0.509	0.807	0.136	0.09
DB07771	[(3,7,11-TRIMETHYL-DODECA-2,6,10-TRIENYLOXYCARBAMOYL)-METHYL]-PHOSPHONIC ACID	-0.677	0.246	1.035	1.126	-1.273	0.205	0.532	0.744	0.709	0.061
DB08371	PARA-(BENZOYL)-PHENYLALANINE	-0.676	-0.352	-0.145	-0.149	-0.628	0.172	0.311	0.12	0.31	0.18
DB00667		-0.675	-0.473	0.12	-0.368	-0.82	0.212	0.469	0.573	0.394	0.086
DB00927	Famotidine	-0.675	-0.612	0.396	-0.606	-0.218	0.17	0.193	0.473	0.182	0.283
DB05318		-0.675	-0.516	0.618	-1.35	-0.862	0.09	0.252	0.75	0.01	0.045
DB05891		-0.675	-0.473	0.12	-0.368	-0.82	0.212	0.469	0.573	0.394	0.086
DB11320		-0.675	-0.473	0.12	-0.368	-0.82	0.212	0.469	0.573	0.394	0.086
DB03978	Tyrosinal	-0.668	-0.431	0.17	-0.054	-0.902	0.108	0.268	0.465	0.285	0.086
DB07562	N-[4-(2,4-DIMETHYL-THIAZOL-5-YL)-PYRIMIDIN-2-YL]-N',N'-DIMETHYL-BENZENE-1,4-DIAMINE	-0.668	0.237	-1.207	-1.478	-0.476	0.157	0.531	0.087	0.045	0.204
DB07644	5-[(1S)-1-(3-chlorophenyl)ethoxy]quinazoline-2,4-diamine	-0.668	-0.689	1.036	0.045	-1.11	0.098	0.134	0.805	0.281	0.028
DB01528	4-Methyl-2,5-dimethoxyamphetamine	-0.667	-0.61	0.087	-0.667	-1.093	0.17	0.243	0.567	0.099	0.016
DB02059	Adenosine-5-Diphosphoribose	-0.667	-0.633	0.495	-0.352	-1.156	0.201	0.217	0.359	0.296	0.089
DB06210	Eltrombopag	-0.667	-0.392	0.005	-0.536	-1.349	0.102	0.34	0.437	0.144	0
DB11294	Coccidioides immitis spherule	-0.666	-0.553	-1.398	-0.014	-0.573	0.138	0.27	0.024	0.368	0.221
DB00024	Thyrotropin alfa	-0.665	-0.622	0.276	-0.46	-0.859	0.135	0.26	0.62	0.184	0.071
DB00557	Hydroxyzine	-0.665	-0.528	-0.305	-0.765	-0.994	0.175	0.371	0.36	0.063	0.021
DB02726	2-Phosphoglycolic Acid	-0.664	-0.812	0.093	-0.2	-0.551	0.227	0.176	0.233	0.327	0.224
DB04957	Azimilide	-0.663	-0.676	0.646	-0.278	-0.763	0.113	0.133	0.726	0.205	0.088
DB04200	Matairesinol	-0.661	-0.562	-0.15	-0.14	-1.086	0.072	0.198	0.334	0.287	0.009
DB01108	Trilostane	-0.66	0.523	-0.281	-2.264	-0.982	0.159	0.677	0.287	0.012	0.096
DB06774	Capsaicin	-0.66	-1.071	0.22	-0.906	-0.177	0.219	0.108	0.411	0.126	0.338
DB07677	2-methyl-3,5,7,8-tetrahydro-4H-thiopyrano[4,3-d]pyrimidin-4-one	-0.66	0.221	-1.931	-2.051	0.566	0.185	0.604	0.01	0.011	0.698
DB08058	4-[3-(1,4-diazepan-1-ylcarbonyl)-4-fluorobenzyl]phthalazin-1(2H)-one	-0.66	0.221	-1.931	-2.051	0.566	0.185	0.604	0.01	0.011	0.698
DB03731	S-2-(Boronoethyl)-L-Cysteine	-0.659	-0.574	0.254	-1.333	-1.218	0.145	0.229	0.59	0.007	0.007
DB03758	Radicicol	-0.659	-0.875	-0.783	0.247	-1.017	0.157	0.108	0.112	0.525	0.116
DB08752	N-[(1S)-2-[(4-cyano-1-methylpiperidin-4-yl)amino]-1-(cyclohexylmethyl)-2-oxoethyl]morpholine-4-carboxamide	-0.659	-0.662	0.584	-0.342	-0.629	0.108	0.144	0.697	0.191	0.175

DB00807	Proparacaine	-0.657	-0.382	0.424	-0.662	-1.084	0.106	0.376	0.674	0.09	0.005
DB09292	Sacubitril	-0.657	-0.691	-2.082	0.283	-0.324	0.096	0.12	0.003	0.514	0.364
DB06439	Tyloxapol	-0.656	-0.588	0.156	-0.702	-0.829	0.155	0.283	0.598	0.08	0.064
DB07691	2-({[3-(3,4-dihydroisoquinolin-2(1H)-ylsulfonyl)phenyl]carbonyl}amino)benzoic acid	-0.656	-0.104	1.059	1.146	-2.158	0.212	0.423	0.768	0.708	0.004
DB00741	Hydrocortisone	-0.652	-0.086	-0.812	-1.34	-1.101	0.14	0.367	0.158	0.06	0.077
DB00625	Efavirenz	-0.65	-0.511	0.398	-0.428	-1.087	0.122	0.29	0.669	0.185	0.002
DB01181	Ifosfamide	-0.65	-0.511	0.398	-0.428	-1.087	0.122	0.29	0.669	0.185	0.002
DB01220	Rifaximin	-0.65	-0.511	0.398	-0.428	-1.087	0.122	0.29	0.669	0.185	0.002
DB01272	Alglucosidase alfa	-0.65	-0.527	0.138	-0.672	-0.94	0.187	0.376	0.606	0.093	0.022
DB08807	Bopindolol	-0.65	-0.31	-2.416	0.305	-1.731	0.216	0.328	0	0.507	0.026
DB08864	Rilpivirine	-0.65	-0.511	0.398	-0.428	-1.087	0.122	0.29	0.669	0.185	0.002
DB00473	Hexylcaine	-0.649	-0.51	0.496	-0.69	-0.984	0.185	0.346	0.695	0.121	0.034
DB08871	Eribulin	-0.649	-0.151	0.02	-0.94	-0.29	0.199	0.4	0.155	0.133	0.316
DB06725	Lornoxicam	-0.648	-0.367	1.524	-1.327	-1.263	0.109	0.31	0.883	0.035	0.029
DB01929	5-Chloryl-2,4,6-Quinazolinetriamine	-0.647	-0.409	-0.062	0.098	-1.18	0.103	0.316	0.4	0.53	0.019
DB01958	5-[4-Tert-Butylphenylsulfanyl]-2,4-Quinazolinediamine	-0.647	-0.409	-0.062	0.098	-1.18	0.103	0.316	0.4	0.53	0.019
DB02001	5-(4-Morpholin-4-Yl-Phenylsulfanyl)-2,4-Quinazolinediamine	-0.647	-0.409	-0.062	0.098	-1.18	0.103	0.316	0.4	0.53	0.019
DB02402	5-(4-Methoxyphenoxy)-2,4-Quinazolinediamine	-0.647	-0.409	-0.062	0.098	-1.18	0.103	0.316	0.4	0.53	0.019
DB04163	5-Phenylsulfanyl-2,4-Quinazolinediamine	-0.647	-0.409	-0.062	0.098	-1.18	0.103	0.316	0.4	0.53	0.019
DB07862	7-(1-ETHYL-PROPYL)-7H-PYRROLO-[3,2-F]QUINAZOLINE-1,3-DIAMINE	-0.647	-0.409	-0.062	0.098	-1.18	0.103	0.316	0.4	0.53	0.019
DB06726	Bufuralol	-0.646	-0.13	-1.896	0.362	-1.174	0.223	0.396	0.015	0.516	0.076
DB00004	Denileukin diftitox	-0.643	-0.26	0.405	0.442	-1.838	0.207	0.339	0.54	0.397	0.01
DB00041	Aldesleukin	-0.643	-0.26	0.405	0.442	-1.838	0.207	0.339	0.54	0.397	0.01
DB00537	Ciprofloxacin	-0.643	-0.452	0.67	-0.957	0.042	0.19	0.279	0.621	0.11	0.446
DB01208	Sparfloxacin	-0.643	-0.452	0.67	-0.957	0.042	0.19	0.279	0.621	0.11	0.446
DB04608	9-HYDROXY-4-PHENYL-6H-PYRROLO[3,4-C]CARBAZOLE-1,3-DIONE	-0.643	-0.659	0.392	-0.352	-0.782	0.215	0.193	0.278	0.282	0.16
DB08569	3-PYRIDIN-4-YL-2,4-DIHYDRO-INDENO[1,2-.C.] PYRAZOLE	-0.642	-1.198	-0.386	-0.156	-0.362	0.149	0.075	0.189	0.326	0.22
DB02597	[2(R,S)-2-Sulfanylheptanoyl]-Phe-Ala	-0.639	-0.589	-2.259	1.044	0.045	0.122	0.188	0.003	0.827	0.573
DB03401	1D-myo-inositol 1,4,5-trisphosphate	-0.638	-0.493	0.397	-0.459	-0.942	0.13	0.298	0.653	0.163	0.035
DB03227	Nicotinamide Mononucleotide	-0.637	-0.598	0.855	-1.248	-0.716	0.097	0.176	0.752	0.03	0.104
DB07795	Fisetin	-0.637	-0.264	-0.377	-0.755	-0.009	0.156	0.295	0.257	0.165	0.406
DB08883	Perampanel	-0.637	-0.506	0.079	-0.677	-0.821	0.29	0.463	0.554	0.106	0.081
DB00866	Alprenolol	-0.635	0.237	-2.127	-1.217	-1.667	0.144	0.532	0.011	0.077	0.033
DB05219	Crisaborole	-0.635	-0.328	1.348	-0.862	-0.411	0.225	0.316	0.847	0.095	0.261
DB09227	Barnidipine	-0.634	-0.441	0.017	-0.302	-0.805	0.123	0.275	0.412	0.196	0.093
DB01442	MMDA	-0.633	-0.415	0.074	-0.544	-1.41	0.151	0.329	0.505	0.12	0
DB08040	N-[(2R)-2-benzyl-4-(hydroxyamino)-4-oxobutanoyl]-L-alanine	-0.633	-0.1	-2.203	-0.061	0.517	0.217	0.412	0.004	0.31	0.62
DB06228	Rivaroxaban	-0.63	-0.463	0.743	-0.331	-0.774	0.109	0.254	0.768	0.177	0.107
DB03349	8-Bromo-Adenosine-5'-Monophosphate	-0.628	-0.615	0.736	-0.296	-0.781	0.106	0.162	0.725	0.193	0.09
DB03975	Mercuribenzoic Acid	-0.628	-0.284	0.829	-1.276	-1.367	0.128	0.403	0.766	0.024	0.01
DB03314	5-fluorotryptophan	-0.626	-0.457	-0.011	-0.062	-0.841	0.12	0.254	0.404	0.286	0.058
DB03886	Biopterin	-0.626	-0.442	0.455	-0.445	-1.191	0.136	0.317	0.697	0.157	0.011

DB08810	Cinitapride	-0.626	-0.552	-0.169	-0.662	-1.203	0.164	0.275	0.394	0.098	0.003
DB00175	Pravastatin	-0.625	-0.579	0.421	-0.368	-0.784	0.15	0.196	0.667	0.179	0.071
DB03785	(3r,5r)-7-((1r,2r,6s,8r,8as)-2,6-Dimethyl-8-{[(2r)-2-Methylbutanoyl]Oxy}-1,2,6,7,8,8a-Hexahydronaphthalen-1-Yl)-3,5-Dihydroxyheptanoic Acid	-0.625	-0.579	0.421	-0.368	-0.784	0.15	0.196	0.667	0.179	0.071
DB01106	Levocabastine	-0.624	-0.526	-0.168	-0.292	-0.623	0.17	0.279	0.387	0.316	0.18
DB05381	Histamine	-0.623	0.364	1.075	-0.388	-0.641	0.222	0.599	0.781	0.274	0.203
DB07283	9-benzyl-2,3,4,9-tetrahydro-1H-carbazole-8-carboxylic acid	-0.623	-0.542	0.912	-0.564	-0.909	0.178	0.282	0.82	0.125	0.048
DB01412	Theobromine	-0.622	-0.46	-0.208	-0.281	-0.817	0.134	0.258	0.317	0.189	0.092
DB02109	Hadacidin	-0.622	-0.461	0.424	-0.414	-0.972	0.113	0.283	0.608	0.169	0.019
DB03510	6-O-Phosphoryl Inosine Monophosphate	-0.622	-0.461	0.424	-0.414	-0.972	0.113	0.283	0.608	0.169	0.019
DB07247	[2'-HYDROXY-3'-(1H-PYRROLO[3,2-C]PYRIDIN-2-YL)-BIPHENYL-3-YLMETHYL]-UREA	-0.622	-0.502	0.403	-0.433	-1.01	0.103	0.277	0.658	0.152	0.027
DB00937	Diethylpropion	-0.621	-0.411	0.015	-0.687	-0.849	0.28	0.467	0.508	0.085	0.058
DB05578	Ramucirumab	-0.621	-0.502	0.711	-1	-0.76	0.116	0.275	0.75	0.042	0.085
DB00616	Candoxatril	-0.618	-0.483	-2.221	0.664	0.004	0.173	0.255	0.004	0.558	0.498
DB00886	Omapatrilat	-0.618	-0.483	-2.221	0.664	0.004	0.173	0.255	0.004	0.558	0.498
DB03633	Lpc-Ether	-0.618	-0.537	0.205	-1.278	-1.173	0.16	0.292	0.576	0.009	0.003
DB08626	Thiorphan	-0.618	-0.483	-2.221	0.664	0.004	0.173	0.255	0.004	0.558	0.498
DB00262	Carmustine	-0.617	-0.479	0.511	-0.43	-1.223	0.144	0.296	0.688	0.157	0.006
DB02267	Argininosuccinate	-0.617	-0.389	0.205	0.048	-1.136	0.154	0.314	0.454	0.306	0.031
DB04880	Enoximone	-0.617	-0.088	-0.239	-0.451	-1.323	0.16	0.661	0.334	0.159	0.002
DB07405	1-(6-CYANO-3-PYRIDYLCARBONYL)-5',8'-DIFLUOROSPIRO[PIPERIDINE-4,2'(1'H)-QUINAZOLINE]-4'-AMINE	-0.617	0.43	0.911	-0.411	-2.435	0.231	0.622	0.718	0.252	0.002
DB09090	Pinaverium	-0.617	-0.408	1.002	-0.451	-0.999	0.16	0.363	0.82	0.159	0.03
DB00065	Infliximab	-0.616	-0.581	-2.189	-0.598	-0.685	0.109	0.185	0.004	0.164	0.132
DB06674	Golimumab	-0.616	-0.581	-2.189	-0.598	-0.685	0.109	0.185	0.004	0.164	0.132
DB08904	Certolizumab pegol	-0.616	-0.581	-2.189	-0.598	-0.685	0.109	0.185	0.004	0.164	0.132
DB02918	Zardaverine	-0.614	0.064	1.171	-1.113	-1.382	0.123	0.578	0.821	0.063	0.016
DB00521	Carteolol	-0.613	0.02	-1.826	0.286	-1.348	0.231	0.477	0.012	0.498	0.038
DB00789	Gadopentetic acid	-0.613	-0.568	0.112	-0.577	-0.843	0.226	0.342	0.689	0.145	0.122
DB01193	Acebutolol	-0.613	0.02	-1.826	0.286	-1.348	0.231	0.477	0.012	0.498	0.038
DB01214	Metipranolol	-0.613	0.02	-1.826	0.286	-1.348	0.231	0.477	0.012	0.498	0.038
DB01291	Pirbuterol	-0.613	0.02	-1.826	0.286	-1.348	0.231	0.477	0.012	0.498	0.038
DB01297	Practolol	-0.613	0.02	-1.826	0.286	-1.348	0.231	0.477	0.012	0.498	0.038
DB02640	Fumagillin	-0.613	-0.525	0.562	-0.35	-0.869	0.108	0.232	0.722	0.227	0.034
DB04846	Celiprolol	-0.613	0.02	-1.826	0.286	-1.348	0.231	0.477	0.012	0.498	0.038
DB08952	Indenolol	-0.613	0.02	-1.826	0.286	-1.348	0.231	0.477	0.012	0.498	0.038
DB09351	Levobetaxolol	-0.613	0.02	-1.826	0.286	-1.348	0.231	0.477	0.012	0.498	0.038
DB00020	Sargramostim	-0.611	-0.058	-0.202	1.229	-1.722	0.235	0.416	0.135	0.755	0.029
DB01234	Dexamethasone	-0.608	0.021	-0.857	-0.612	-2.046	0.162	0.456	0.122	0.215	0.019
DB03310	Glutathione disulfide	-0.607	-0.473	0.254	-0.538	-1.059	0.202	0.319	0.59	0.14	0.02
DB04395	Phosphoaminophosphonic Acid-Adenylate Ester	-0.606	-0.797	-1.073	0.381	-0.558	0.146	0.151	0.109	0.584	0.226
DB04674	2-HYDROXY-3,5-DIIODOBENZOIC ACID	-0.606	-0.479	0.306	-0.524	-0.895	0.161	0.316	0.628	0.131	0.046
DB01836	[4-(6-Chloro-Naphthalene-2-Sulfonyl)-Piperazin-1-Yl]-(3,4,5,6-Tetrahydro-2h-[1,4']Bipyridinyl-4-Yl)-Methanone	-0.601	-0.605	0.749	-0.295	-0.772	0.121	0.173	0.742	0.198	0.105
DB02068	Delta-Amino Valeric Acid	-0.601	-0.509	-0.033	-0.67	-0.85	0.262	0.383	0.492	0.096	0.054

DB02269	[4-({[5-Benzyloxy-1-(3-Carbamimidoyl-Benzyl)-1h-Indole-2-Carbonyl]-Amino}-Methyl)-Phenyl]-Trimethyl-Ammonium	-0.601	-0.605	0.749	-0.295	-0.772	0.121	0.173	0.742	0.198	0.105
DB02483	Etheno-Nad	-0.601	-0.573	0.883	-0.821	-1.342	0.156	0.206	0.749	0.112	0.01
DB08228	5,8-dimethoxy-1,4-dimethylquinolin-2(1H)-one	-0.599	-0.452	0.216	-0.602	-0.891	0.174	0.381	0.632	0.085	0.043
DB08744	6-methoxy-9-methyl[1,3]dioxolo[4,5-h]quinolin-8(9H)-one	-0.599	-0.452	0.216	-0.602	-0.891	0.174	0.381	0.632	0.085	0.043
DB00671	Cefixime	-0.598	0.143	2.04	-1.902	-1.17	0.145	0.479	0.994	0.024	0.072
DB00882	Clomifene	-0.598	0.239	0.378	-0.387	-1.083	0.167	0.534	0.571	0.283	0.095
DB07302	(9S,10E,12Z)-9-hydroxyoctadeca-10,12-dienoic acid	-0.598	-0.053	0.216	0.672	-1.596	0.219	0.469	0.414	0.587	0.007
DB08931	Riociguat	-0.598	-0.425	-0.057	-0.62	-0.921	0.198	0.381	0.43	0.107	0.027
DB01270	Ranibizumab	-0.597	-0.523	-0.113	-0.433	-0.631	0.204	0.314	0.399	0.2	0.165
DB09083	Ivabradine	-0.596	-0.371	0.053	-0.691	-0.941	0.209	0.462	0.54	0.095	0.031
DB00435	Nitric Oxide	-0.595	-0.498	-0.208	-0.73	-0.833	0.254	0.385	0.394	0.076	0.061
DB06987	(R)-Atenolol	-0.595	-0.407	0.667	-0.915	-0.555	0.147	0.368	0.725	0.073	0.228
DB07642	5-{[1-(2-fluorobenzyl)piperidin-4-yl]methoxy}quinazoline-2,4-diamine	-0.595	-0.487	0.351	-0.37	-0.923	0.134	0.266	0.627	0.178	0.024
DB09158	Trypan blue free acid	-0.595	-0.353	-0.439	-0.516	-0.839	0.137	0.385	0.08	0.195	0.064
DB01143	Amifostine	-0.593	-0.404	0.942	-0.259	-0.766	0.156	0.296	0.793	0.2	0.102
DB02052	Indirubin-3'-Monoxime	-0.593	-0.168	-0.235	-1.141	-0.588	0.164	0.316	0.32	0.078	0.173
DB04444	Tetrafluoroaluminate Ion	-0.593	-0.477	0.086	0.496	-0.421	0.25	0.262	0.374	0.43	0.258
DB00452	Framycetin	-0.592	-0.412	-1.351	-0.918	0.07	0.203	0.289	0.058	0.13	0.453
DB02638	Terlipressin	-0.592	-0.483	-0.664	-0.639	-0.766	0.159	0.29	0.132	0.108	0.081
DB02018	Amido Phenyl Pyruvic Acid	-0.59	-0.9	0.214	0.294	0.024	0.174	0.093	0.483	0.377	0.451
DB00195	Betaxolol	-0.588	0.081	-1.915	0.226	-1.366	0.233	0.516	0.012	0.47	0.05
DB00612	Bisoprolol	-0.588	0.081	-1.915	0.226	-1.366	0.233	0.516	0.012	0.47	0.05
DB01102	Arbutamine	-0.588	0.081	-1.915	0.226	-1.366	0.233	0.516	0.012	0.47	0.05
DB01203	Nadolol	-0.588	0.081	-1.915	0.226	-1.366	0.233	0.516	0.012	0.47	0.05
DB01218	Halofantrine	-0.588	-0.734	-1.121	-0.926	-0.246	0.162	0.164	0.065	0.11	0.271
DB01274	Arformoterol	-0.588	0.081	-1.915	0.226	-1.366	0.233	0.516	0.012	0.47	0.05
DB01955	1,4-Butanediol	-0.588	-0.608	1.162	-0.133	-0.565	0.14	0.168	0.849	0.358	0.201
DB08808	Bupranolol	-0.588	0.081	-1.915	0.226	-1.366	0.233	0.516	0.012	0.47	0.05
DB08893	Mirabegron	-0.588	0.081	-1.915	0.226	-1.366	0.233	0.516	0.012	0.47	0.05
DB09080	Olodaterol	-0.588	0.081	-1.915	0.226	-1.366	0.233	0.516	0.012	0.47	0.05
DB09082	Vilanterol	-0.588	0.081	-1.915	0.226	-1.366	0.233	0.516	0.012	0.47	0.05
DB00070	Hyaluronidase (ovine)	-0.587	-0.314	0.296	0.283	-1.315	0.201	0.427	0.542	0.514	0
DB02333	Deoxyuridine-5'-Triphosphate	-0.587	-0.36	0.344	-1.537	-1.284	0.155	0.311	0.52	0.042	0.027
DB06205	Vorhyaluronidase alfa	-0.587	-0.314	0.296	0.283	-1.315	0.201	0.427	0.542	0.514	0
DB04566	Inosinic Acid	-0.583	-0.476	-1.395	-0.157	0.348	0.236	0.242	0.059	0.283	0.531
DB00138	Cystine	-0.582	-0.32	0.26	-0.405	-0.869	0.236	0.491	0.613	0.266	0.057
DB00207	Azithromycin	-0.582	-0.223	1.112	0.009	0.246	0.251	0.341	0.768	0.399	0.5
DB02795	P-Anisic Acid	-0.582	-0.367	0.769	-1.036	-0.942	0.196	0.412	0.759	0.065	0.04
DB03661	Cysteinesulfonic Acid	-0.582	-0.806	-0.134	-0.199	0.608	0.238	0.162	0.173	0.31	0.645
DB07248	7-PYRIDIN-2-YL-N-(3,4,5-TRIMETHOXYPHENYL)-7H-PYRROLO[2,3-D]PYRIMIDIN-2-AMINE	-0.582	0.418	-1.654	-0.822	-0.362	0.231	0.612	0.026	0.143	0.29
DB01246	Alimemazine	-0.581	-0.416	0.355	-0.396	-0.826	0.217	0.349	0.644	0.187	0.07
DB04456	Arsenous acid	-0.581	-0.515	1.948	0.63	-1.131	0.155	0.218	0.936	0.691	0.036
DB00135	Tyrosine	-0.58	-0.421	0.269	-0.572	-1.545	0.251	0.289	0.432	0.156	0.036
DB06267	Udenafil	-0.579	-0.467	-0.363	-0.626	-0.76	0.212	0.384	0.268	0.104	0.112
DB09488	Acrivastine	-0.579	-0.467	-0.363	-0.626	-0.76	0.212	0.384	0.268	0.104	0.112
DB01791	Piclamilast	-0.578	-0.313	0.479	-0.956	-0.684	0.217	0.318	0.553	0.093	0.162
DB00274	Cefmetazole	-0.577	-0.47	0.276	-0.46	-0.859	0.261	0.421	0.62	0.184	0.071
DB00436	Bendroflumethiazide	-0.576	0.105	1.813	-0.672	-1.692	0.24	0.51	0.934	0.18	0.02
DB03884	Phenylpyruvic acid	-0.575	-0.414	0.098	-0.647	-0.959	0.231	0.422	0.545	0.106	0.038

DB07352	Apigenin	-0.575	-0.911	-2.495	-1.556	0.638	0.154	0.111	0.002	0.043	0.716
DB04310	2-[(Formyl-Hydroxy-Amino)-Methyl]-Heptanoic Acid [1-(2-Hydroxymethyl-Pyrrolidine-1-Carbonyl)-2-Methyl-Propyl]-Amide	-0.574	-0.356	-1.55	0.167	1.702	0.242	0.315	0.025	0.467	0.935
DB02375	Myricetin	-0.573	-0.947	-1.01	-0.188	0.091	0.227	0.114	0.118	0.407	0.493
DB09231	Benidipine	-0.57	-0.39	0.836	-0.014	-0.646	0.189	0.291	0.749	0.255	0.167
DB08221	N-{4-METHYL-3-[(3-PYRIMIDIN-4-YLPYRIDIN-2-YL)AMINO]PHENYL}-3-(TRIFLUOROMETHYL)BENZAMIDE	-0.569	0.043	0.459	-0.798	-1.136	0.175	0.537	0.576	0.102	0.054
DB00439	Cerivastatin	-0.568	-0.357	1.11	0.119	-1.104	0.161	0.323	0.818	0.302	0.042
DB02142	Pyridoxamine-5'-Phosphate	-0.568	-0.282	0.65	-0.127	-1.211	0.177	0.382	0.692	0.239	0.029
DB03660	4-Iodo-L-phenylalanine	-0.568	-0.246	0.066	-0.161	-0.981	0.171	0.422	0.43	0.239	0.049
DB07276	5-CYANO-N-(2,5-DIMETHOXYBENZYL)-6-ETHOXYPYRIDINE-2-CARBOXAMIDE	-0.568	-0.818	1.203	-0.16	0.105	0.234	0.139	0.83	0.323	0.442
DB02459	4-Guanidinobenzoic Acid	-0.567	0.046	1.598	-2.26	-1.455	0.251	0.474	0.883	0.014	0.046
DB13159		-0.566	-0.427	0.713	-1.042	-0.98	0.229	0.359	0.748	0.042	0.036
DB00946	Phenprocoumon	-0.565	-0.022	-2.148	0.013	-0.692	0.309	0.727	0	0.369	0.139
DB01153	Sertaconazole	-0.565	-0.527	-1.263	-0.949	-0.25	0.187	0.202	0.046	0.099	0.254
DB01418	Acenocoumarol	-0.565	-0.022	-2.148	0.013	-0.692	0.309	0.727	0	0.369	0.139
DB04881	Elacridar	-0.564	-0.421	-0.329	-0.559	-0.714	0.245	0.41	0.287	0.124	0.125
DB03083	IC261	-0.563	0.449	0.484	-2.29	-1.345	0.155	0.664	0.339	0.012	0.052
DB08180	2-[METHYL-(5-GERANYL-4-METHYL-PENT-3-ENYL)-AMINO]-ETHYL-DIPHOSPHATE	-0.563	-0.076	0.908	-0.164	-1.116	0.183	0.546	0.755	0.24	0.021
DB01207	Ridogrel	-0.562	-0.455	0.243	-0.509	-0.891	0.189	0.345	0.577	0.145	0.049
DB00562	Benzthiazide	-0.561	0.185	1.95	-1.227	-1.598	0.255	0.516	0.936	0.085	0.039
DB01282	Carbetocin	-0.561	-0.41	-0.541	-0.522	-0.699	0.206	0.333	0.177	0.125	0.109
DB04387	1-Hydroxy-2-Amino-3-Cyclohexylpropane	-0.56	-0.449	0.868	-0.526	-0.704	0.213	0.317	0.815	0.125	0.104
DB03052	Dazoxiben	-0.558	-0.432	-0.033	-0.67	-0.85	0.316	0.475	0.492	0.096	0.054
DB08604	Triclosan	-0.558	-0.45	-0.9	-0.187	-0.622	0.187	0.233	0.132	0.333	0.192
DB02515	3-Phosphoglycerol	-0.556	-0.357	0.128	-0.021	-0.782	0.151	0.319	0.454	0.285	0.1
DB00620	Triamcinolone	-0.555	0.043	-1.599	-2.001	-0.414	0.191	0.425	0.041	0.017	0.237
DB00938	Salmeterol	-0.555	0.021	-0.764	0.867	-1.301	0.249	0.457	0.125	0.767	0.056
DB04878	Voglibose	-0.555	-0.295	0.218	-0.664	-1.029	0.247	0.516	0.578	0.111	0.021
DB07853	[4-({4-[(5-CYCLOPROPYL-1H-PYRAZOL-3-YL)AMINO]QUINAZOLIN-2-YL}IMINO)CYCLOHEXA-2,5-DIEN-1-YL]ACETONITRILE	-0.555	-0.345	1.388	-1.228	-0.766	0.189	0.374	0.879	0.038	0.088
DB08278	1-(2-CYCLOPROPYLETHYL)-3-(1,1-DIOXIDO-2H-1,2,4-BENZOTHIADIAZIN-3-YL)-6-FLUORO-4-HYDROXYQUINOLIN-2(1H)-ONE	-0.555	-0.204	1.647	-0.101	-1.549	0.159	0.467	0.908	0.249	0.003
DB03981	1,4-Dideoxy-5-Dehydro-O2-Sulfo-Glucuronic Acid	-0.554	-0.467	0.652	-0.236	-0.722	0.202	0.338	0.735	0.359	0.114
DB04564	Gluconolactone	-0.553	-0.164	0.461	-0.455	-1.214	0.216	0.636	0.682	0.167	0.001
DB06720	Velaglucerase alfa	-0.553	-0.164	0.461	-0.455	-1.214	0.216	0.636	0.682	0.167	0.001
DB03602	S-Benzyl-Glutathione	-0.538	-0.352	0.338	-0.465	-0.871	0.206	0.408	0.639	0.148	0.042
DB01812	Adenosine-3'-5'-Diphosphate	-0.534	0.098	1.631	-1.432	-0.539	0.264	0.472	0.912	0.049	0.212
DB00290	Bleomycin	-0.533	-0.17	0.463	-0.865	-0.572	0.198	0.413	0.567	0.091	0.212
DB04707	Hydroxyfasudil	-0.533	-0.693	0.49	-0.954	-0.556	0.178	0.156	0.414	0.119	0.181
DB00820	Tadalafil	-0.532	-0.332	-0.04	-0.476	-0.998	0.22	0.423	0.429	0.17	0.024
DB02388	Cyclohexyl-{4-[5-(3,4-Dichlorophenyl)-2-Piperidin-4-Yl-3-Propyl-3h-Imidazol-4-Yl]-Pyrimidin-2-Yl}Amine	-0.532	-0.829	1.294	-0.017	0.311	0.266	0.149	0.865	0.399	0.548

DB08021	5-bromo-N-(3-chloro-2-(4-(prop-2-ynyl)piperazin-1-yl)phenyl)furan-2-carboxamide	-0.532	-0.829	1.294	-0.017	0.311	0.266	0.149	0.865	0.399	0.548
DB08025	N-{2'-[(4-FLUOROPHENYL)AMINO]-4,4'-BIPYRIDIN-2-YL}-4-METHOXYCYCLOHEXANECARBOXAMIDE	-0.532	-0.829	1.294	-0.017	0.311	0.266	0.149	0.865	0.399	0.548
DB00075	Muromonab	-0.531	0.084	0.016	-1.285	0.339	0.263	0.46	0.364	0.078	0.548
DB00111	Daclizumab	-0.531	-0.742	0.062	-0.066	0.298	0.258	0.185	0.349	0.389	0.511
DB00469	Tenoxicam	-0.531	-0.259	1.311	-1.303	-0.972	0.202	0.442	0.885	0.011	0.031
DB00814	Meloxicam	-0.531	-0.259	1.311	-1.303	-0.972	0.202	0.442	0.885	0.011	0.031
DB00821	Carprofen	-0.531	-0.259	1.311	-1.303	-0.972	0.202	0.442	0.885	0.011	0.031
DB00963	Bromfenac	-0.531	-0.259	1.311	-1.303	-0.972	0.202	0.442	0.885	0.011	0.031
DB01283	Lumiracoxib	-0.531	-0.259	1.311	-1.303	-0.972	0.202	0.442	0.885	0.011	0.031
DB01294	Bismuth subsalicylate	-0.531	-0.259	1.311	-1.303	-0.972	0.202	0.442	0.885	0.011	0.031
DB01399	Salsalate	-0.531	-0.259	1.311	-1.303	-0.972	0.202	0.442	0.885	0.011	0.031
DB01401	Choline magnesium trisalicylate	-0.531	-0.259	1.311	-1.303	-0.972	0.202	0.442	0.885	0.011	0.031
DB01419	Antrafenine	-0.531	-0.259	1.311	-1.303	-0.972	0.202	0.442	0.885	0.011	0.031
DB01424	Aminophenazone	-0.531	-0.259	1.311	-1.303	-0.972	0.202	0.442	0.885	0.011	0.031
DB01435	Antipyrine	-0.531	-0.259	1.311	-1.303	-0.972	0.202	0.442	0.885	0.011	0.031
DB01600	Tiaprofenic acid	-0.531	-0.259	1.311	-1.303	-0.972	0.202	0.442	0.885	0.011	0.031
DB04920	Clevidipine	-0.531	-0.256	2.223	-0.426	-1.15	0.207	0.392	0.955	0.182	0.029
DB05095	Cimicoxib	-0.531	-0.259	1.311	-1.303	-0.972	0.202	0.442	0.885	0.011	0.031
DB06802	Nepafenac	-0.531	-0.259	1.311	-1.303	-0.972	0.202	0.442	0.885	0.011	0.031
DB08942	Isoxicam	-0.531	-0.259	1.311	-1.303	-0.972	0.202	0.442	0.885	0.011	0.031
DB08976	Floctafenine	-0.531	-0.259	1.311	-1.303	-0.972	0.202	0.442	0.885	0.011	0.031
DB08981	Fenbufen	-0.531	-0.259	1.311	-1.303	-0.972	0.202	0.442	0.885	0.011	0.031
DB09212	Loxoprofen	-0.531	-0.259	1.311	-1.303	-0.972	0.202	0.442	0.885	0.011	0.031
DB09214	Dexketoprofen	-0.531	-0.259	1.311	-1.303	-0.972	0.202	0.442	0.885	0.011	0.031
DB00731	Nateglinide	-0.528	0.004	1.278	1.433	-1.351	0.256	0.509	0.833	0.88	0.031
DB01252	Mitiglinide	-0.528	0.004	1.278	1.433	-1.351	0.256	0.509	0.833	0.88	0.031
DB00066	Follitropin	-0.524	-0.262	-0.266	-0.505	-0.842	0.288	0.596	0.339	0.157	0.049
DB00094	Urofollitropin	-0.524	-0.262	-0.266	-0.505	-0.842	0.288	0.596	0.339	0.157	0.049
DB09460	Sodium carbonate	-0.523	0.103	2.025	-1.823	-1.274	0.21	0.523	0.943	0.025	0.041
DB01616	Alverine	-0.521	-0.385	0.686	-0.462	-0.824	0.331	0.508	0.814	0.458	0.13
DB03501	Galactose-uridine-5'-diphosphate	-0.521	-0.341	-0.612	-0.635	-0.699	0.262	0.415	0.155	0.108	0.106
DB00437	Allopurinol	-0.52	-0.511	0.732	-0.169	-0.677	0.203	0.234	0.756	0.253	0.132
DB00613	Amodiaquine	-0.519	-0.538	1.358	0.202	-0.632	0.273	0.246	0.841	0.448	0.192
DB02180	Myristoyl-Coa	-0.518	-0.306	1.449	-1.289	-0.741	0.182	0.334	0.885	0.06	0.112
DB01580	Oxprenolol	-0.515	0.092	-1.41	0.64	-1.426	0.282	0.521	0.06	0.686	0.048
DB03674		-0.515	-0.112	0.3	-1.063	-1.022	0.198	0.476	0.513	0.066	0.047
DB08464	METHYL 3-CHLORO-2-{3-[(2,5-DIHYDROXY-4-METHOXYPHENYL)AMINO]-3-OXOPROPYL}-4,6-DIHYDROXYBENZOATE	-0.514	-1.009	-0.866	-0.061	-0.124	0.174	0.098	0.101	0.359	0.332
DB08465	2-(3-AMINO-2,5,6-TRIMETHOXYPHENYL)ETHYL 5-CHLORO-2,4-DIHYDROXYBENZOATE	-0.514	-1.009	-0.866	-0.061	-0.124	0.174	0.098	0.101	0.359	0.332
DB00083	Botulinum toxin type A	-0.513	-0.339	1.324	0.229	-1.32	0.218	0.302	0.859	0.366	0.025
DB01785	Dimethylallyl Diphosphate	-0.513	-0.302	0.2	-0.428	-0.789	0.288	0.491	0.615	0.179	0.075
DB03317	Heme C	-0.513	-0.567	1.053	0.17	-0.458	0.17	0.183	0.837	0.577	0.266
DB02123	Glycochenodeoxycholic Acid	-0.511	-0.028	0	-0.185	-1.153	0.221	0.586	0.415	0.231	0.013
DB00410	Mupirocin	-0.51	-0.042	0.246	-0.823	-1.525	0.229	0.635	0.529	0.108	0.001
DB00488	Altretamine	-0.51	-0.321	0.188	-1.088	-0.787	0.428	0.409	0.492	0.123	0.381
DB08827	Lomitapide	-0.51	-0.27	-0.202	-0.904	-0.947	0.428	0.412	0.416	0.247	0.311
DB00405	Dexbrompheniramine	-0.509	-0.361	0.277	-0.464	-0.834	0.29	0.461	0.625	0.164	0.052
DB09081	Idebenone	-0.509	0	0.903	-0.13	-1.22	0.261	0.672	0.793	0.362	0.006
DB00341	Cetirizine	-0.508	0.144	0.035	0.072	-1.493	0.237	0.611	0.369	0.298	0.013

DB08601	tributylstannanyl	-0.508	-0.297	1.09	-0.832	0.724	0.25	0.344	0.785	0.14	0.694
DB03127	Benzamidine	-0.507	-1.022	-1.156	-1.193	0.022	0.205	0.122	0.074	0.079	0.387
DB02475	Deoxyguanidinoproclavaminic acid	-0.506	-0.624	1.632	0.447	-0.603	0.198	0.196	0.893	0.43	0.182
DB00222	Glimepiride	-0.503	-0.29	0.846	-0.019	-0.876	0.239	0.445	0.773	0.446	0.056
DB04883	Darusentan	-0.503	-0.5	0.917	-0.114	-0.704	0.198	0.24	0.788	0.264	0.139
DB06268	Sitaxentan	-0.503	-0.5	0.917	-0.114	-0.704	0.198	0.24	0.788	0.264	0.139
DB06403	Ambrisentan	-0.503	-0.5	0.917	-0.114	-0.704	0.198	0.24	0.788	0.264	0.139
DB08932	Macitentan	-0.503	-0.5	0.917	-0.114	-0.704	0.198	0.24	0.788	0.264	0.139
DB01295	Bevantolol	-0.502	0.135	-0.971	0.302	-1.333	0.247	0.541	0.127	0.507	0.047
DB06855	6-fluoro-2-(2-hydroxy-3-isobutoxy-phenyl)-1H-benzoimidazole-5-carboxamidine	-0.5	0.412	-0.495	-0.828	-1.183	0.214	0.699	0.054	0.103	0.049
DB06775	Carglumic acid	-0.499	-0.15	0.546	-0.433	-1.058	0.341	0.643	0.706	0.2	0.007
DB08298		-0.499	0.006	0.916	-1.094	-1.184	0.25	0.485	0.733	0.068	0.071
DB08794	Ethyl biscoumacetate	-0.499	0.724	-2.449	-0.731	-1.165	0.227	0.852	0.002	0.133	0.055
DB01280	Nelarabine	-0.498	-0.237	-0.091	-1.081	-0.559	0.25	0.562	0.42	0.024	0.203
DB01858	[1-(4-Fluorobenzyl)Cyclobutyl]Methyl (1s)-1-[Oxo(1h-Pyrazol-5-Ylamino)Acetyl]Pentylcarbamate	-0.498	-0.455	1.448	-0.063	-0.402	0.222	0.254	0.886	0.237	0.275
DB07513	7-[(3-CHLOROBENZYL)OXY]-4-[(METHYLAMINO)METHYL]-2H-CHROMEN-2-ONE	-0.498	0.723	1.089	0.523	-2.482	0.266	0.77	0.792	0.572	0.004
DB07967	9-CYCLOPENTYL-6-[2-(3-IMIDAZOL-1-YL-PROPOXY)-PHENYLAMINO]-9H-PURINE-2-CARBONITRILE	-0.498	-0.455	1.448	-0.063	-0.402	0.222	0.254	0.886	0.237	0.275
DB09052	Blinatumomab	-0.497	-0.11	-0.718	-0.219	-0.695	0.225	0.506	0.029	0.217	0.127
DB00895	Benzylpenicilloyl Polylysine	-0.495	-0.382	0.692	-0.642	-0.783	0.355	0.489	0.766	0.097	0.082
DB02897	Acetylphosphate	-0.495	-0.382	0.137	-0.642	-0.783	0.355	0.489	0.562	0.097	0.082
DB06692	Aprotinin	-0.495	-0.003	2.252	0.435	-0.814	0.279	0.458	0.967	0.456	0.138
DB02740	3-Indolebutyric Acid	-0.494	0.139	-4.197	0.923	-0.582	0.262	0.509	0	0.627	0.182
DB07713	(1S)-1-{[(4'-methoxy-1,1'-biphenyl-4-yl)sulfonyl]amino}-2-methylpropylphosphonic acid	-0.493	-0.344	0.1	0.103	-0.509	0.196	0.363	0.467	0.359	0.209
DB00716	Nedocromil	-0.492	-0.279	-0.033	0.143	-0.053	0.205	0.283	0.356	0.447	0.354
DB08660	1,2,5,8-tetrahydroxyanthracene-9,10-dione	-0.492	-1.12	-1.126	-0.908	-0.219	0.197	0.097	0.069	0.117	0.275
DB00461	Nabumetone	-0.491	-0.079	1.084	-1.306	-1.3	0.241	0.427	0.792	0.052	0.045
DB04600	4-[(3-BROMO-4-O-SULFAMOYLBENZYL)(4-CYANOPHENYL)AMINO]-4H-[1,2,4]-TRIAZOLE	-0.491	-0.276	1.263	-0.835	-0.979	0.221	0.453	0.874	0.114	0.047
DB04601	4-[(4-O-SULFAMOYLBENZYL)(4-CYANOPHENYL)AMINO]-4H-[1,2,4]-TRIAZOLE	-0.491	-0.276	1.263	-0.835	-0.979	0.221	0.453	0.874	0.114	0.047
DB07596	(17beta)-17-(cyanomethyl)-2-methoxyestra-1(10),2,4-trien-3-yl sulfamate	-0.491	-0.276	1.263	-0.835	-0.979	0.221	0.453	0.874	0.114	0.047
DB01782	2,6-Dihydroanthra/1,9-Cd/Pyrazol-6-One	-0.489	-0.066	0.316	0.187	-0.405	0.244	0.39	0.564	0.513	0.279
DB09242	Moxonidine	-0.488	-0.186	1.262	-0.091	-0.996	0.222	0.418	0.847	0.23	0.07
DB02112	Zk-806450	-0.487	-0.547	0.13	-0.013	-0.359	0.218	0.198	0.475	0.288	0.299
DB04424	RPR128515	-0.487	-0.547	0.13	-0.013	-0.359	0.218	0.198	0.475	0.288	0.299
DB06845	(S)-N-(4-carbamimidoylbenzyl)-1-(2-(cyclopentylamino)ethanoyl)pyrrolidine-2-carboxamide	-0.487	-0.547	0.13	-0.013	-0.359	0.218	0.198	0.475	0.288	0.299

DB06850	(S)-N-(4-carbamimidoylbenzyl)-1-(2-(cyclohexylamino)ethanoyl)pyrrolidine-2-carboxamide	-0.487	-0.547	0.13	-0.013	-0.359	0.218	0.198	0.475	0.288	0.299
DB06853	N-cycloheptylglycyl-N-(4-carbamimidoylbenzyl)-L-prolinamide	-0.487	-0.547	0.13	-0.013	-0.359	0.218	0.198	0.475	0.288	0.299
DB06858	N-cyclooctylglycyl-N-(4-carbamimidoylbenzyl)-L-prolinamide	-0.487	-0.547	0.13	-0.013	-0.359	0.218	0.198	0.475	0.288	0.299
DB07088	(S)-N-(4-carbamimidoylbenzyl)-1-(2-(cyclopentyloxy)ethanoyl)pyrrolidine-2-carboxamide	-0.487	-0.547	0.13	-0.013	-0.359	0.218	0.198	0.475	0.288	0.299
DB07091	(S)-N-(4-carbamimidoylbenzyl)-1-(2-(cyclohexyloxy)ethanoyl)pyrrolidine-2-carboxamide	-0.487	-0.547	0.13	-0.013	-0.359	0.218	0.198	0.475	0.288	0.299
DB07095	(S)-N-(4-carbamimidoylbenzyl)-1-(3-cyclopentylpropanoyl)pyrrolidine-2-carboxamide	-0.487	-0.547	0.13	-0.013	-0.359	0.218	0.198	0.475	0.288	0.299
DB07131	(S)-N-(4-carbamimidoylbenzyl)-1-(3-cyclohexylpropanoyl)pyrrolidine-2-carboxamide	-0.487	-0.547	0.13	-0.013	-0.359	0.218	0.198	0.475	0.288	0.299
DB08254	2-NAPHTHALENESULFONIC ACID	-0.487	-0.547	0.13	-0.013	-0.359	0.218	0.198	0.475	0.288	0.299
DB06288	Amisulpride	-0.485	-1	2.961	1.323	-0.531	0.282	0.12	0.995	0.883	0.221
DB00582	Voriconazole	-0.484	0.076	1.001	-0.127	-1.314	0.264	0.692	0.817	0.374	0.005
DB02049	2-{4-[4-(4-Chloro-Phenoxy)-Benzenesulfonyl]-Tetrahydro-Pyran-4-Yl}-N-Hydroxy-Acetamide	-0.484	-0.399	0.274	-0.544	-0.724	0.298	0.421	0.607	0.128	0.113
DB03958	7-methyl-guanosine-5'-triphosphate-5'-guanosine	-0.484	-0.451	0.872	-0.143	-0.649	0.219	0.266	0.773	0.233	0.152
DB00339	Pyrazinamide	-0.483	-0.136	0.455	-1.354	-1.226	0.232	0.516	0.638	0.025	0.016
DB01024	Mycophenolic acid	-0.481	-0.395	-1.379	-0.531	-0.269	0.219	0.251	0.053	0.228	0.304
DB06842	(4R)-4-(3-butoxy-4-methoxybenzyl)imidazolidin-2-one	-0.479	-0.141	0.883	-1.214	-0.636	0.25	0.501	0.782	0.042	0.157
DB00750	Prilocaine	-0.478	0.158	-1.859	0.398	-1.262	0.27	0.541	0.01	0.524	0.064
DB00035	Desmopressin	-0.477	-0.273	-0.705	-0.545	-0.674	0.266	0.409	0.115	0.14	0.131
DB00067	Vasopressin	-0.477	-0.273	-0.705	-0.545	-0.674	0.266	0.409	0.115	0.14	0.131
DB09232	Cilnidipine	-0.476	-0.345	0.417	-0.433	-0.834	0.261	0.402	0.675	0.154	0.067
DB00232	Methyclothiazide	-0.475	-0.193	1.44	-0.974	-1.084	0.26	0.494	0.893	0.089	0.025
DB01288	Fenoterol	-0.475	0.103	-2.094	0.185	-1.389	0.277	0.514	0.007	0.47	0.05
DB03693	N-(2-Aminoethyl)-5-Chloroisoquinoline-8-Sulfonamide	-0.474	0.015	-0.286	-2.023	-1.018	0.203	0.411	0.215	0.016	0.075
DB09019	Bromhexine	-0.474	-0.172	0.216	-0.46	-1.074	0.24	0.505	0.554	0.135	0.025
DB07530	(1R,3R)-5-[(2E)-3-{(1S,3R)-2,2,3-trimethyl-3-[6,6,6-trifluoro-5-hydroxy-5-(trifluoromethyl)hex-3-yn-1-yl]cyclopentyl}prop-2-en-1-ylidene]cyclohexane-1,3-diol	-0.47	0.137	1.083	1.256	-1.593	0.277	0.512	0.785	0.76	0.031
DB08742	1,3-CYCLOHEXANEDIOL, 4-METHYLENE-5-[(2E)-[(1S,3AS,7AS)-OCTAHYDRO-1-(5-HYDROXY-5-METHYL-1,3-HEXADIYNYL)-7A-METHYL-4H-INDEN-4-YLIDENE]ETHYLIDENE]-, (1R,3S,5Z)	-0.47	0.137	1.083	1.256	-1.593	0.277	0.512	0.785	0.76	0.031
DB00859	Penicillamine	-0.469	-0.145	-1.117	-0.476	-0.786	0.391	0.679	0.02	0.175	0.165
DB01997	3-Bromo-7-Nitroindazole	-0.469	0.799	1.037	-0.228	-3.064	0.261	0.751	0.78	0.305	0.001
DB02044	N-(3-(Aminomethyl)Benzyl)Acetamidine	-0.469	0.799	1.037	-0.228	-3.064	0.261	0.751	0.78	0.305	0.001
DB02335	2-Aminothiazoline	-0.469	0.799	1.037	-0.228	-3.064	0.261	0.751	0.78	0.305	0.001

DB02539		-0.469	0.799	1.037	-0.228	-3.064	0.261	0.751	0.78	0.305	0.001
DB02833	[4-(2-Amino-4-Methyl-Thiazol-5-Yl)-Pyrimidin-2-Yl]-(3-Nitro-Phenyl)-Amine	-0.469	-0.041	-0.87	-1.202	-0.117	0.204	0.38	0.144	0.079	0.363
DB03910	S,S'-(1,3-Phenylene-Bis(1,2-Ethanediyl))Bis-Isothiourea	-0.469	0.799	1.037	-0.228	-3.064	0.261	0.751	0.78	0.305	0.001
DB04223	Nitroarginine	-0.469	0.799	1.037	-0.228	-3.064	0.261	0.751	0.78	0.305	0.001
DB07003	(2S)-2-methyl-2,3-dihydrothieno[2,3-f][1,4]oxazepin-5-amine	-0.469	0.799	1.037	-0.228	-3.064	0.261	0.751	0.78	0.305	0.001
DB07389	N-[2-(6-AMINO-4-METHYLPYRIDIN-2-YL)ETHYL]-4-CYANOBENZAMIDE	-0.469	0.799	1.037	-0.228	-3.064	0.261	0.751	0.78	0.305	0.001
DB00693	Fluorescein	-0.468	0.245	1.028	-0.942	-1.357	0.235	0.701	0.792	0.084	0.009
DB02869	3-amino-5-phenylpentane	-0.465	-0.254	0.909	-0.558	-0.752	0.377	0.545	0.799	0.114	0.1
DB06803	Niclosamide	-0.465	0.167	-0.505	-0.595	-0.41	0.209	0.5	0.259	0.208	0.287
DB01359	Penbutolol	-0.462	0.028	-1.91	0.368	-1.052	0.279	0.458	0.009	0.528	0.099
DB03600	Capric acid	-0.462	-0.383	0.437	-0.332	-0.731	0.291	0.345	0.664	0.193	0.103
DB01549	Rolicyclidine	-0.459	-0.223	0.982	-0.511	-0.877	0.341	0.563	0.81	0.138	0.052
DB00356	Chlorzoxazone	-0.456	0.548	0.754	-0.134	-2.803	0.287	0.644	0.615	0.353	0.002
DB00983	Formoterol	-0.456	-0.426	-1.344	-0.335	0.021	0.196	0.242	0.064	0.289	0.389
DB13161	Nusinersen	-0.456	-0.051	2.18	-1.832	-1.255	0.273	0.436	0.983	0.017	0.058
DB00737	Meclizine	-0.455	0.044	-0.247	-0.199	-1.282	0.252	0.643	0.283	0.212	0.016
DB07863	2-chloro-5-nitro-N-phenylbenzamide	-0.454	-0.194	1.294	-0.015	0.003	0.295	0.369	0.886	0.395	0.418
DB08402	2-[(2,4-DICHLOROBENZOYL)AMINO]-5-(PYRIMIDIN-2-YLOXY)BENZOIC ACID	-0.454	-0.194	1.294	-0.015	0.003	0.295	0.369	0.886	0.395	0.418
DB02839	2,4-Dihydroxybenzoic Acid	-0.453	-0.147	0.728	-1.356	-1.01	0.285	0.536	0.748	0.024	0.027
DB01620	Pheniramine	-0.452	-0.034	0.828	0.191	-0.999	0.276	0.6	0.768	0.518	0.035
DB01905	2-(2-Hydroxy-5-Methoxy-Phenyl)-1h-Benzoimidazole-5-Carboxamidine	-0.451	-0.373	0.054	-0.169	-0.483	0.262	0.333	0.433	0.232	0.236
DB02526	CRA_10655	-0.451	-0.373	0.054	-0.169	-0.483	0.262	0.333	0.433	0.232	0.236
DB03876	Thieno[2,3-B]Pyridine-2-Carboxamidine	-0.451	-0.373	0.054	-0.169	-0.483	0.262	0.333	0.433	0.232	0.236
DB00968	Methyldopa	-0.447	0.571	-1.592	-0.317	-0.904	0.256	0.729	0.046	0.36	0.145
DB00697	Tizanidine	-0.445	-0.059	1.696	-0.118	-0.966	0.255	0.497	0.916	0.227	0.065
DB01009	Ketoprofen	-0.445	-0.179	0.659	-0.721	-0.342	0.285	0.366	0.613	0.116	0.286
DB08150	4-(4-chlorobenzyl)-1-(7H-pyrrolo[2,3-d]pyrimidin-4-yl)piperidin-4-aminium	-0.445	-1.064	0.318	0.036	-0.502	0.205	0.076	0.527	0.444	0.196
DB08756	(R)-TRANS-4-(1-AMINOETHYL)-N-(4-PYRIDYL) CYCLOHEXANECARBOXAMIDE	-0.442	-0.781	0.735	-0.535	-0.832	0.223	0.155	0.543	0.228	0.133
DB11619	Gestrinone	-0.442	-0.542	-1.006	-0.432	-0.135	0.211	0.205	0.08	0.229	0.312
DB08782	4-(2-AMINOETHYL)BENZENESULFONAMIDE	-0.441	-0.081	1.181	-1.022	-1.091	0.286	0.563	0.843	0.083	0.026
DB00870	Suprofen	-0.44	0.202	2.351	-1.327	-1.555	0.25	0.644	0.964	0.035	0.005
DB07482	(2R)-N-[(2R)-2-(DIHYDROXYBORYL)-1-L-PROLYLPYRROLIDIN-2-YL]-N-[(5R)-5-(DIHYDROXYBORYL)-1-L-PROLYLPYRROLIDIN-2-YL]-L-PROLINAMIDE	-0.438	-0.253	-0.158	-0.374	-0.621	0.356	0.497	0.368	0.183	0.159
DB08220	(8alpha,10alpha,13alpha,17beta)-17-[(4-hydroxyphenyl)carbonyl]androsta-3,5-diene-3-carboxylic acid	-0.438	0.393	0.818	0.846	-2.068	0.279	0.676	0.674	0.599	0.003
DB00264	Metoprolol	-0.435	0.155	-2.054	0.814	-0.899	0.311	0.526	0.01	0.761	0.119
DB00896	Rimexolone	-0.434	0.006	0.558	-0.884	-1.282	0.296	0.448	0.338	0.137	0.07

DB02266	Flufenamic Acid	-0.434	-1.117	0.965	-0.456	0.138	0.224	0.089	0.775	0.256	0.469
DB01592	Iron	-0.433	-0.779	-0.803	0.467	0.548	0.292	0.18	0.142	0.602	0.642
DB01120	Gliclazide	-0.432	-0.299	0.525	0.174	-0.298	0.301	0.434	0.662	0.538	0.394
DB08388	5-(2-ETHOXYETHYL)-5-[4-(4-FLUOROPHENOXY)PHENOXY]PYRIMIDINE-2,4,6(1H,3H,5H)-TRIONE	-0.431	-0.167	-0.105	-0.241	-0.727	0.27	0.489	0.344	0.205	0.099
DB01198	Zopiclone	-0.43	-0.528	1.917	-0.466	-0.578	0.262	0.24	0.946	0.166	0.167
DB03352	S-Arsonocysteine	-0.43	-0.057	1.247	0.065	-1.016	0.32	0.617	0.857	0.508	0.032
DB00553	Methoxsalen	-0.429	-0.27	0.189	-0.348	-0.821	0.276	0.389	0.535	0.167	0.08
DB00880	Chlorothiazide	-0.429	0.168	1.586	-0.821	-1.586	0.285	0.667	0.909	0.13	0.006
DB04270	(S)-3-(4-(2-Carbazol-9-Yl-Ethoxy)-Phenyl)-2-Ethoxy-Propionic Acid	-0.429	-0.301	1.124	1.441	-1.539	0.295	0.321	0.816	0.833	0.032
DB05416	Cardarine	-0.429	-0.301	1.124	1.441	-1.539	0.295	0.321	0.816	0.833	0.032
DB07053	2-{5-[3-(7-PROPYL-3-TRIFLUOROMETHYLBENZO[D]ISOXAZOL-6-YLOXY)PROPOXY]INDOL-1-YL}ETHANOIC ACID	-0.429	-0.301	1.124	1.441	-1.539	0.295	0.321	0.816	0.833	0.032
DB07070	(2S)-2-{3-[({[2-fluoro-4-(trifluoromethyl)phenyl]carbonyl}amino)methyl]-4-methoxybenzyl}butanoic acid	-0.429	-0.301	1.124	1.441	-1.539	0.295	0.321	0.816	0.833	0.032
DB08078	{4-[3-(4-acetyl-3-hydroxy-2-propylphenoxy)propoxy]phenoxy}acetic acid	-0.429	-0.301	1.124	1.441	-1.539	0.295	0.321	0.816	0.833	0.032
DB08915	Aleglitazar	-0.429	-0.301	1.124	1.441	-1.539	0.295	0.321	0.816	0.833	0.032
DB09006	Clinofibrate	-0.429	-0.301	1.124	1.441	-1.539	0.295	0.321	0.816	0.833	0.032
DB04948	Lofexidine	-0.427	-0.353	1.842	-0.387	-0.863	0.277	0.363	0.936	0.162	0.07
DB08588	2-({2-[(3R)-3-AMINOPIPERIDIN-1-YL]-4-OXOQUINAZOLIN-3(4H)-YL}METHYL)BENZONITRILE	-0.426	-0.225	-0.101	-0.368	-0.599	0.383	0.522	0.389	0.169	0.175
DB00388	Phenylephrine	-0.425	0.015	-1.002	0.464	-1.928	0.296	0.448	0.125	0.572	0.019
DB00525	Tolnaftate	-0.425	0.012	0.525	-0.127	-1.066	0.308	0.686	0.692	0.389	0.017
DB08073	(2S)-1-(1H-INDOL-3-YL)-3-{[5-(3-METHYL-1H-INDAZOL-5-YL)PYRIDIN-3-YL]OXY}PROPAN-2-AMINE	-0.423	-0.754	0.135	-0.32	-0.157	0.236	0.134	0.382	0.298	0.325
DB00960	Pindolol	-0.422	-0.306	-1.488	1.161	-1.045	0.296	0.329	0.041	0.839	0.107
DB00736	Esomeprazole	-0.421	-0.2	0.154	-0.92	-0.539	0.377	0.563	0.496	0.071	0.23
DB01438	Phenazopyridine	-0.418	-0.033	0.487	-0.175	-1.069	0.286	0.445	0.528	0.308	0.064
DB02836	Guanosine 5'-Diphosphate 2':3'-Cyclic Monophosphate	-0.417	-0.265	0.532	-0.976	0.026	0.211	0.293	0.353	0.102	0.398
DB04077		-0.416	-0.601	0.56	0.326	-0.285	0.296	0.249	0.584	0.513	0.319
DB07988	2-[3-(5-MERCAPTO-[1,3,4]THIADIAZOL-2YL)-UREIDO]-N-METHYL-3-PENTAFLUOROPHENYL-PROPIONAMIDE	-0.416	-0.221	-0.341	0.706	0.192	0.308	0.365	0.079	0.525	0.484
DB09128	Brexpiprazole	-0.416	-0.924	1.65	0.944	-1.365	0.305	0.141	0.904	0.698	0.063
DB00701	Amprenavir	-0.415	-0.325	-0.306	-1.125	-1.189	0.295	0.331	0.225	0.066	0.051
DB01004	Ganciclovir	-0.415	-0.156	0.513	-0.436	-0.83	0.358	0.571	0.693	0.15	0.063
DB01034	Cerulenin	-0.415	0.335	0.591	-1.34	-1.103	0.275	0.652	0.599	0.04	0.06
DB03424	Ubenimex	-0.415	-0.094	0.336	0.611	-0.26	0.316	0.411	0.472	0.484	0.331
DB04228	(2r)-Amino(3,5-Dihydroxyphenyl)Acetic Acid	-0.415	-0.156	0.513	-0.436	-0.83	0.358	0.571	0.693	0.15	0.063
DB00373	Timolol	-0.414	0.263	-1.941	0.253	-1.264	0.297	0.549	0.01	0.489	0.058
DB03144	N-Omega-Hydroxy-L-Arginine	-0.411	0.483	1.142	-0.54	-2.99	0.298	0.627	0.811	0.217	0.001
DB00650	Leucovorin	-0.409	-0.108	1.09	0.102	-1.291	0.278	0.467	0.832	0.326	0.017
DB00848	Levamisole	-0.409	-0.235	0.452	-0.185	-0.514	0.306	0.408	0.64	0.228	0.224
DB06813	Pralatrexate	-0.409	-0.108	1.09	0.102	-1.291	0.278	0.467	0.832	0.326	0.017

DB08734	6,6-DIMETHYL-1-[3-(2,4,5-TRICHLOROPHENOXY)PROPOXY]-1,6-DIHYDRO-1,3,5-TRIAZINE-2,4-DIAMINE	-0.409	-0.108	1.09	0.102	-1.291	0.278	0.467	0.832	0.326	0.017
DB07126	O6-CYCLOHEXYLMETHOXY-2-(4'-SULPHAMOYLANILINO) PURINE	-0.407	-0.385	-0.421	-0.676	-0.543	0.216	0.25	0.236	0.193	0.197
DB03747	3ar,5r,6s,7r,7ar-5-Hydroxymethyl-2-Methyl-5,6,7,7a-Tetrahydro-3ah-Pyrano[3,2-D]Thiazole-6,7-Diol	-0.406	0.001	0.266	0.019	-0.891	0.268	0.505	0.473	0.279	0.077
DB00117	Histidine	-0.405	0.077	1.355	-1.141	-1.811	0.282	0.527	0.833	0.062	0.011
DB02343	3,6,9,12,15-Pentaoxaheptadecane	-0.403	-0.269	0.965	-0.185	-0.725	0.282	0.401	0.756	0.222	0.103
DB00107	Oxytocin	-0.402	-0.07	-0.74	-0.241	-0.824	0.303	0.49	0.124	0.206	0.125
DB01863	Inositol 1,3,4,5-Tetrakisphosphate	-0.402	-0.605	-0.793	-0.454	0.345	0.231	0.186	0.127	0.25	0.578
DB00322	Floxuridine	-0.401	-0.238	0.562	-0.35	-0.718	0.384	0.544	0.722	0.227	0.093
DB00432	Trifluridine	-0.401	-0.238	0.562	-0.35	-0.718	0.384	0.544	0.722	0.227	0.093
DB00448	Lansoprazole	-0.401	0.232	0.833	-0.892	0.54	0.302	0.539	0.687	0.147	0.658
DB01037	Selegiline	-0.401	0.014	1.128	-0.501	-1.07	0.344	0.656	0.854	0.13	0.025
DB01101	Capecitabine	-0.401	-0.238	0.562	-0.35	-0.718	0.384	0.544	0.722	0.227	0.093
DB01276	Exenatide	-0.401	-0.238	-0.083	-0.35	-0.567	0.384	0.544	0.413	0.227	0.205
DB01632	5-O-phosphono-alpha-D-ribofuranosyl diphosphate	-0.401	-0.478	1.206	0.138	-0.504	0.278	0.231	0.83	0.329	0.205
DB02824	N-Pyridoxyl-Glycine-5-Monophosphate	-0.401	-0.108	0.929	-0.001	-1.069	0.276	0.526	0.797	0.294	0.037
DB03800	Deoxyuridine monophosphate	-0.401	-0.238	0.562	-0.35	-0.718	0.384	0.544	0.722	0.227	0.093
DB06655	Liraglutide	-0.401	-0.238	-0.083	-0.35	-0.567	0.384	0.544	0.413	0.227	0.205
DB09043	Albiglutide	-0.401	-0.238	-0.083	-0.35	-0.567	0.384	0.544	0.413	0.227	0.205
DB09045	Dulaglutide	-0.401	-0.238	-0.083	-0.35	-0.567	0.384	0.544	0.413	0.227	0.205
DB09265	Lixisenatide	-0.401	-0.238	-0.083	-0.35	-0.567	0.384	0.544	0.413	0.227	0.205
DB11256	Levomefolic acid	-0.401	-0.238	0.562	-0.35	-0.718	0.384	0.544	0.722	0.227	0.093
DB00446	Chloramphenicol	-0.399	-0.083	0.648	-0.315	-0.952	0.388	0.633	0.724	0.204	0.035
DB07776	Flavone	-0.398	-0.584	-1.511	-2.205	1.27	0.207	0.201	0.054	0.011	0.93
DB06953	2-CHLORO-5-(3-CHLORO-PHENYL)-6-[(4-CYANO-PHENYL)-(3-METHYL-3H-IMIDAZOL-4-YL)- METHOXYMETHYL]-NICOTINONITRILE	-0.397	0.814	1.593	0.48	-1.888	0.274	0.83	0.887	0.437	0.001
DB07216	(11S)-8-CHLORO-11-[1-(METHYLSULFONYL)PIPERIDIN-4-YL]-6-PIPERAZIN-1-YL-11H-BENZO[5,6]CYCLOHEPTA[1,2-B]PYRIDINE	-0.397	0.814	1.593	0.48	-1.888	0.274	0.83	0.887	0.437	0.001
DB07895	ALPHA-HYDROXYFARNESYLPHOSPHONIC ACID	-0.397	0.814	1.593	0.48	-1.888	0.274	0.83	0.887	0.437	0.001
DB08676	(20S)-19,20,22,23-TETRAHYDRO-19-OXO-5H,21H-18,20-ETHANO-12,14-ETHENO-6,10-METHENOBENZ[D]IMIDAZO[4,3-L][1,6,9,13]OXATRIAZACYCLONOADECOSINE-9-CARBONITRILE	-0.397	0.814	1.593	0.48	-1.888	0.274	0.83	0.887	0.437	0.001
DB00694	Daunorubicin	-0.396	0.097	-1.606	-1.854	-0.778	0.219	0.468	0.031	0.025	0.164
DB01917	Putrescine	-0.395	0.318	0.114	-0.039	-1.438	0.294	0.762	0.465	0.273	0.009
DB08301	N-({[4-(AMINOSULFONYL)PHENYL]AMINO}CARBONYL)-4-METHYLBENZENESULFONAMIDE	-0.395	-0.148	1.424	-0.062	-0.853	0.338	0.524	0.902	0.362	0.077
DB00932	Tipranavir	-0.393	-0.687	1.307	0.116	-0.029	0.305	0.181	0.851	0.343	0.416
DB08023	N-cyclohexyl-4-imidazo[1,2-a]pyridin-3-yl-N-methylpyrimidin-2-amine	-0.392	0.086	-0.418	0.474	-0.81	0.282	0.553	0.068	0.457	0.125

DB08026	2-{4-[(4-imidazo[1,2-a]pyridin-3-ylpyrimidin-2-yl)amino]piperidin-1-yl}-N-methylacetamide	-0.392	0.086	-0.418	0.474	-0.81	0.282	0.553	0.068	0.457	0.125
DB07545	N-{3-[(4-{[3-(TRIFLUOROMETHYL)PHENYL]AMINO}PYRIMIDIN-2-YL)AMINO]PHENYL}CYCLOPROPANECARBOXAMIDE	-0.389	-0.375	0.976	-0.551	-0.341	0.227	0.281	0.583	0.231	0.263
DB05251		-0.388	-0.09	-0.147	-0.236	-0.764	0.328	0.522	0.329	0.203	0.116
DB06196	Icatibant	-0.387	-0.176	0.563	-0.22	-0.733	0.413	0.574	0.695	0.382	0.108
DB08950	Indoramin	-0.384	-0.432	3.175	0.3	-1.404	0.297	0.275	0.993	0.357	0.062
DB04716	2-(1,1-DIMETHYLETHYL)9-FLUORO-3,6-DIHYDRO-7H-BENZ[H]-IMIDAZ[4,5-F]ISOQUINOLIN-7-ONE	-0.383	0.37	-1.581	-1.698	-0.763	0.258	0.604	0.044	0.024	0.161
DB00830	Phenmetrazine	-0.382	-0.175	0.201	-0.509	-0.892	0.346	0.571	0.538	0.125	0.048
DB08992	Eperisone	-0.382	-0.07	0.739	-0.441	-1.01	0.312	0.556	0.734	0.155	0.033
DB02471	Nojirimycine Tetrazole	-0.38	-0.172	0.977	-0.445	-0.837	0.368	0.564	0.812	0.166	0.048
DB01601	Lopinavir	-0.378	-0.156	0.067	-0.159	-0.702	0.35	0.533	0.442	0.238	0.119
DB04368	Bb-3497	-0.378	-0.494	0.383	0.272	-0.041	0.314	0.261	0.499	0.337	0.444
DB01556	Chlorphentermine	-0.377	-0.155	0.338	-0.465	-0.716	0.456	0.612	0.639	0.148	0.099
DB04695	Farnesyl thiopyrophosphate	-0.377	-0.172	0.576	-0.184	-0.496	0.347	0.452	0.653	0.218	0.221
DB07817	1-{3-[(4-pyridin-2-ylpiperazin-1-yl)sulfonyl]phenyl}-3-(1,3-thiazol-2-yl)urea	-0.377	0.077	-0.456	0.408	-0.57	0.289	0.537	0.061	0.419	0.199
DB07856	6-{4-[4-(4-CHLOROPHENYL)PIPERIDIN-4-YL]PHENYL}-9H-PURINE	-0.377	0.045	1.17	0.263	-0.903	0.248	0.421	0.831	0.473	0.108
DB01015	Sulfamethoxazole	-0.373	-0.321	0.713	-1.042	-0.98	0.448	0.463	0.748	0.042	0.036
DB04324	Ovalicin	-0.373	-0.171	1.042	0.045	-0.832	0.315	0.456	0.813	0.336	0.083
DB04339	Carbocisteine	-0.373	-0.321	0.713	-1.042	-0.98	0.448	0.463	0.748	0.042	0.036
DB06203	Alogliptin	-0.372	0.544	0.695	0.571	-1.322	0.29	0.711	0.671	0.583	0.057
DB08987	Etidocaine	-0.372	-0.135	1.146	0.761	-0.761	0.354	0.516	0.825	0.805	0.099
DB02287	2-(2-Hydroxy-Phenyl)-3h-Benzoimidazole-5-Carboxamidine	-0.371	-0.32	0.406	0.404	-0.231	0.311	0.318	0.54	0.389	0.33
DB02463	2-(2-Hydroxy-Phenyl)-1h-Indole-5-Carboxamidine	-0.371	-0.32	0.406	0.404	-0.231	0.311	0.318	0.54	0.389	0.33
DB03173	CRA_10433	-0.371	-0.32	0.406	0.404	-0.231	0.311	0.318	0.54	0.389	0.33
DB04215	CRA_9076	-0.371	-0.32	0.406	0.404	-0.231	0.311	0.318	0.54	0.389	0.33
DB04299	Maleic Acid	-0.371	0.444	0.792	-1.441	-0.785	0.318	0.697	0.708	0.049	0.13
DB04563	CRA_9678	-0.371	-0.32	0.406	0.404	-0.231	0.311	0.318	0.54	0.389	0.33
DB07229	3-{5-[AMINO(IMINIO)METHYL]-1H-INDOL-2-YL}-5-METHOXY-1,1'-BIPHENYL-2-OLATE	-0.371	-0.32	0.406	0.404	-0.231	0.311	0.318	0.54	0.389	0.33
DB04932	Defibrotide	-0.369	-0.068	-0.33	-0.426	-0.659	0.385	0.621	0.275	0.147	0.149
DB04954	Tecadenoson	-0.369	-0.068	-0.33	-0.426	-0.659	0.385	0.621	0.275	0.147	0.149
DB05511	Piclidenoson	-0.369	-0.068	-0.33	-0.426	-0.659	0.385	0.621	0.275	0.147	0.149
DB00204	Dofetilide	-0.368	-0.133	1.172	0.377	-0.886	0.404	0.562	0.9	0.792	0.076
DB05239	Cobimetinib	-0.366	-0.364	0.92	-0.226	0.17	0.309	0.318	0.705	0.276	0.498
DB06777	Chenodeoxycholic acid	-0.366	0.372	-0.242	0.224	-1.795	0.31	0.667	0.236	0.378	0.013
DB08911	Trametinib	-0.366	-0.364	0.92	-0.226	0.17	0.309	0.318	0.705	0.276	0.498
DB02994	Cacodylic acid	-0.364	-0.132	1.166	0.417	-1.266	0.304	0.438	0.795	0.431	0.038
DB04715	IMIDAZOPYRIDAZIN 1	-0.364	0.65	-2.307	-0.673	-0.469	0.326	0.804	0.002	0.142	0.247
DB01613	Erythrityl tetranitrate	-0.363	-0.052	-0.19	-0.336	-0.787	0.435	0.678	0.346	0.209	0.084
DB01819	Phosphoenolpyruvate	-0.362	-0.162	1.79	0.616	-1.382	0.32	0.429	0.909	0.51	0.03
DB06744	ginkgolide-B	-0.362	-0.169	0.025	0.208	-0.547	0.34	0.448	0.406	0.499	0.186
DB08232	[5-(5-Amino-1H-pyrrolo[3,2-b]pyridin-2-yl)-6-hydroxy-3'-nitro-3-biphenylyl]acetic acid	-0.362	-0.131	0.96	0.068	-0.944	0.31	0.445	0.773	0.279	0.09
DB01210	Levobunolol	-0.361	0.41	-1.908	0.357	-1.453	0.323	0.6	0.01	0.524	0.05

DB01271	Idursulfase	-0.361	-0.104	0.653	-0.241	-0.742	0.463	0.667	0.764	0.287	0.07
DB01279	Galsulfase	-0.361	-0.104	0.653	-0.241	-0.742	0.463	0.667	0.764	0.287	0.07
DB08705	6-(5-BROMO-2-HYDROXYPHENYL)-2-OXO-4-PHENYL-1,2-DIHYDROPYRIDINE-3-CARBONITRILE	-0.361	0.008	-2.405	0.824	-0.112	0.305	0.485	0.001	0.576	0.403
DB11362	Selexipag	-0.361	-0.198	-0.024	-0.241	-0.436	0.463	0.561	0.428	0.287	0.289
DB02848	{4-[3-(6,7-Diethoxy-Quinazolin-4-Ylamino)-Phenyl]-Thiazol-2-Yl}-Methanol	-0.36	-0.116	0.074	-0.107	-0.614	0.356	0.525	0.459	0.257	0.156
DB08820	Ivacaftor	-0.36	0.248	0.809	1.101	-1.821	0.317	0.691	0.713	0.728	0.001
DB09280	Lumacaftor	-0.36	0.248	0.809	1.101	-1.821	0.317	0.691	0.713	0.728	0.001
DB02320	1-N-Acetyl-Beta-D-Glucosamine	-0.359	0.001	0.411	0.228	-1.101	0.306	0.509	0.559	0.348	0.042
DB03067	4-{2,4-Bis[(3-Nitrobenzoyl)Amino]Phenoxy}Phthalic Acid	-0.359	0.001	0.411	0.228	-1.101	0.306	0.509	0.559	0.348	0.042
DB04044	4-{2-[(3-Nitrobenzoyl)Amino]Phenoxy}Phthalic Acid	-0.359	0.001	0.411	0.228	-1.101	0.306	0.509	0.559	0.348	0.042
DB06986	2-CHLORO-N-[(1R,2R)-1-HYDROXY-2,3-DIHYDRO-1H-INDEN-2-YL]-6H-THIENO[2,3-B]PYRROLE-5-CARBOXAMIDE	-0.359	0.001	0.411	0.228	-1.101	0.306	0.509	0.559	0.348	0.042
DB07807	(3R,4R,5R)-5-(HYDROXYMETHYL)-1-(3-PHENYLPROPYL)PIPERIDINE-3,4-DIOL	-0.359	0.001	0.411	0.228	-1.101	0.306	0.509	0.559	0.348	0.042
DB08322	2-DEOXY-3,4-BIS-O-[3-(4-HYDROXYPHENYL)PROPANOYL]-L-THREO-PENTARIC ACID	-0.359	0.001	0.411	0.228	-1.101	0.306	0.509	0.559	0.348	0.042
DB00323	Tolcapone	-0.356	0.297	0.74	0.041	-0.64	0.297	0.695	0.701	0.291	0.151
DB03026	Phosphoglycolohydroxamic Acid	-0.356	-0.065	0.212	0.281	-0.743	0.298	0.449	0.46	0.371	0.128
DB08872	Gabapentin enacarbil	-0.356	-0.006	-0.094	-0.291	-0.737	0.4	0.656	0.399	0.202	0.112
DB00511	Acetyldigitoxin	-0.354	-0.2	0.925	0.036	-0.721	0.459	0.563	0.792	0.374	0.09
DB01078	Deslanoside	-0.354	-0.2	0.925	0.036	-0.721	0.459	0.563	0.792	0.374	0.09
DB01345	Potassium cation	-0.354	-0.2	0.925	0.036	-0.721	0.459	0.563	0.792	0.374	0.09
DB01430	Almitrine	-0.354	-0.2	0.925	0.036	-0.721	0.459	0.563	0.792	0.374	0.09
DB02338	Nadph Dihydro-Nicotinamide-Adenine-Dinucleotidephosphate	-0.354	0.036	-0.257	-0.149	-0.885	0.34	0.621	0.273	0.251	0.074
DB07476	N-[4-(AMINOSULFONYL)PHENYL]-2-MERCAPTOBENZAMIDE	-0.353	-0.043	0.044	-1.337	-1.068	0.347	0.549	0.444	0.027	0.039
DB04451	4-Methylpiperazin-1-Yl Carbonyl Group	-0.352	-0.176	0.557	-0.403	-0.652	0.421	0.516	0.708	0.156	0.132
DB07675	(2S)-2-ETHOXY-3-{4-[2-(10H-PHENOXAZIN-10-YL)ETHOXY]PHENYL}PROPANOIC ACID	-0.352	0.006	1.08	1.178	-1.223	0.331	0.458	0.772	0.728	0.063
DB07842	(2S)-2-(4-ethylphenoxy)-3-phenylpropanoic acid	-0.352	0.006	1.08	1.178	-1.223	0.331	0.458	0.772	0.728	0.063
DB08121	(2S)-2-(biphenyl-4-yloxy)-3-phenylpropanoic acid	-0.352	0.006	1.08	1.178	-1.223	0.331	0.458	0.772	0.728	0.063
DB08760	(2S)-2-(4-chlorophenoxy)-3-phenylpropanoic acid	-0.352	0.006	1.08	1.178	-1.223	0.331	0.458	0.772	0.728	0.063
DB09198	Lobeglitazone	-0.352	0.006	1.08	1.178	-1.223	0.331	0.458	0.772	0.728	0.063
DB00847	Cysteamine	-0.351	1.251	1.463	0.014	-1.647	0.303	0.883	0.882	0.362	0.026
DB02073	Biliverdine IX Alpha	-0.351	-0.282	0.795	-0.19	-0.469	0.396	0.436	0.758	0.214	0.24
DB07420	(1R)-4-(3-phenoxyphenyl)-1-phosphonobutane-1-sulfonic acid	-0.351	0.025	1.207	-0.34	-0.963	0.395	0.671	0.863	0.166	0.039
DB08358	2-(2-QUINOLIN-3-YLPYRIDIN-4-YL)-1,5,6,7-TETRAHYDRO-4H-PYRROLO[3,2-C]PYRIDIN-4-ONE	-0.351	-1.101	1.418	-1.244	0.089	0.219	0.086	0.952	0.075	0.431
DB00152	Thiamine	-0.348	-0.483	-0.661	-0.532	0.478	0.224	0.203	0.18	0.225	0.637

DB03374	3,5-Diiodotyrosine	-0.347	-0.34	1.355	-0.124	-0.685	0.224	0.266	0.892	0.334	0.134
DB09026	Aliskiren	-0.347	-0.153	0.679	-0.221	-0.48	0.354	0.463	0.709	0.192	0.233
DB01536	Androstenedione	-0.344	-0.195	0.822	1.063	-1.354	0.332	0.36	0.713	0.788	0.069
DB04960	Tipifarnib	-0.344	0.13	0.971	0.109	-1.118	0.362	0.683	0.802	0.36	0.014
DB05829	Parathyroid hormone	-0.343	-0.256	0.712	-0.059	-0.609	0.372	0.42	0.74	0.315	0.164
DB06285	Teriparatide	-0.343	-0.256	0.712	-0.059	-0.609	0.372	0.42	0.74	0.315	0.164
DB00217	Bethanidine	-0.342	0.425	-0.081	0.401	-1.494	0.338	0.611	0.313	0.544	0.041
DB09552	Thonzonium	-0.342	-0.237	0.556	-0.363	-0.559	0.488	0.562	0.73	0.211	0.203
DB00888	Mechlorethamine	-0.341	-0.122	0.073	-1.002	-0.406	0.395	0.571	0.486	0.041	0.281
DB00951	Isoniazid	-0.341	0.051	1.084	-0.005	-0.89	0.415	0.686	0.836	0.407	0.06
DB07244	5-{4-[(3,5-DIFLUOROBENZYL)AMINO]PHENYL}-6-ETHYLPYRIMIDINE-2,4-DIAMINE	-0.34	-0.322	1.751	0.089	-0.755	0.332	0.33	0.922	0.35	0.101
DB07632	5-(2-chlorophenyl)-1,3,4-thiadiazole-2-sulfonamide	-0.34	0.379	0.39	-2.38	-1.25	0.322	0.62	0.534	0.007	0.057
DB08439	Parecoxib	-0.34	0.022	2.15	-1.716	-0.722	0.331	0.487	0.947	0.039	0.156
DB01030	Topotecan	-0.335	-0.919	0.215	-1.196	0.676	0.254	0.098	0.482	0.078	0.716
DB02126	4-Carboxycinnamic Acid	-0.334	0.75	0.646	-0.633	-1.199	0.317	0.818	0.607	0.147	0.059
DB07427	2-[(2-methoxy-5-methylphenoxy)methyl]pyridine	-0.334	0.75	0.646	-0.633	-1.199	0.317	0.818	0.607	0.147	0.059
DB07428	4-[(5-methoxy-2-methylphenoxy)methyl]pyridine	-0.334	0.75	0.646	-0.633	-1.199	0.317	0.818	0.607	0.147	0.059
DB07645	SEBACIC ACID	-0.334	0.75	0.646	-0.633	-1.199	0.317	0.818	0.607	0.147	0.059
DB08077	2-[4-({[(3,5-DICHLOROPHENYL)AMINO]CARBONYL}AMINO)PHENOXY]-2-METHYLPROPANOIC ACID	-0.334	0.75	0.646	-0.633	-1.199	0.317	0.818	0.607	0.147	0.059
DB08262	2,6-dicarboxynaphthalene	-0.334	0.75	0.646	-0.633	-1.199	0.317	0.818	0.607	0.147	0.059
DB08486	Efaproxiral	-0.334	0.75	0.646	-0.633	-1.199	0.317	0.818	0.607	0.147	0.059
DB08632	1,3,5-BENZENETRICARBOXYLIC ACID	-0.334	0.75	0.646	-0.633	-1.199	0.317	0.818	0.607	0.147	0.059
DB00569	Fondaparinux	-0.333	-0.097	0.777	-0.322	-0.747	0.402	0.582	0.762	0.166	0.107
DB01225	Enoxaparin	-0.333	-0.097	0.777	-0.322	-0.747	0.402	0.582	0.762	0.166	0.107
DB01460	Diethyltryptamine	-0.333	-0.144	0.448	-0.426	-0.651	0.459	0.581	0.671	0.168	0.135
DB02692		-0.333	0.768	1.551	-0.263	-2.981	0.326	0.73	0.908	0.315	0
DB04941	Crofelemer	-0.331	0.442	0.414	1.006	-1.914	0.337	0.717	0.481	0.729	0.005
DB01146	Diphenylpyraline	-0.33	-0.089	-0.044	-0.505	-0.853	0.401	0.599	0.424	0.147	0.058
DB03948	6-Chloropurine Riboside, 5'-Monophosphate	-0.329	0.105	-2.365	-0.438	0.248	0.348	0.545	0.004	0.207	0.526
DB07608	N-(5-{[(2S)-4-amino-2-(3-chlorophenyl)butanoyl]amino}-1H-indazol-3-yl)benzamide	-0.328	0.004	0.581	0.064	-0.951	0.441	0.656	0.661	0.366	0.042
DB00495	Zidovudine	-0.327	-0.86	-1.217	-0.998	1.03	0.323	0.145	0.053	0.131	0.799
DB01213	Fomepizole	-0.327	0.014	1.052	0.06	-0.795	0.378	0.614	0.827	0.445	0.092
DB04828	Zomepirac	-0.327	0.056	1.84	-0.868	-0.812	0.359	0.604	0.934	0.116	0.085
DB02300	Calcipotriol	-0.326	0.572	1.037	-0.198	-1.084	0.328	0.697	0.753	0.288	0.095
DB02639	4-methylumbelliferyl ??-D-glucoside	-0.326	0.01	1.267	-1.34	-0.734	0.341	0.571	0.85	0.04	0.105
DB03046	7-Methoxy-8-[1-(Methylsulfonyl)-1h-Pyrazol-4-Yl]Naphthalene-2-Carboximidamide	-0.326	0.189	-0.281	-0.178	-0.628	0.336	0.527	0.093	0.248	0.185
DB06816	Pyrvinium	-0.326	-0.24	-1.393	-1.371	0.402	0.243	0.297	0.054	0.059	0.589
DB05389	Tetrachlorodecaoxide	-0.321	-0.138	0.494	-0.286	-0.725	0.446	0.597	0.684	0.216	0.089
DB02633	Cibacron Blue	-0.32	0.546	0.602	-2.045	-1.775	0.338	0.776	0.594	0.009	0.009
DB00687	Fludrocortisone	-0.319	0.034	-1.333	-0.912	-0.314	0.24	0.419	0.066	0.115	0.25
DB01365	Mephentermine	-0.319	0.186	0.465	0.458	-1.964	0.337	0.52	0.56	0.575	0.018
DB04581	1-benzylimidazole	-0.319	0.267	1.626	0.9	-0.73	0.416	0.778	0.911	0.81	0.103
DB00706	Tamsulosin	-0.317	-0.796	3.513	0.904	-1.093	0.333	0.179	1	0.644	0.103
DB03615	Ribostamycin	-0.317	-0.062	0.296	-1.105	-0.919	0.443	0.619	0.542	0.033	0.036

DB08513	[4-({5-(AMINOCARBONYL)-4-[(3-METHYLPHENYL)AMINO]PYRIMIDIN-2-YL}AMINO)PHENYL]ACETIC ACID	-0.317	-0.587	0.763	-0.165	-0.105	0.237	0.173	0.469	0.332	0.345
DB06663	Pasireotide	-0.316	-0.029	0.265	-0.205	-0.802	0.381	0.537	0.531	0.224	0.094
DB06959	(2S)-1-(3H-Indol-3-yl)-3-{[5-(6-isoquinolinyl)-3-pyridinyl]oxy}-2-propanamine	-0.316	-0.231	1.099	-0.207	-0.924	0.239	0.308	0.803	0.297	0.102
DB07204	(1S)-1-(1H-INDOL-3-YLMETHYL)-2-(2-PYRIDIN-4-YL-[1,7]NAPHTYRIDIN-5-YLOXY)-EHYLAMINE	-0.316	-0.231	1.099	-0.207	-0.924	0.239	0.308	0.803	0.297	0.102
DB07583	(4R,2S)-5'-(4-(4-CHLOROBENZYLOXY)PYRROLIDIN-2-YLMETHANESULFONYL)ISOQUINOLINE	-0.316	-0.231	1.099	-0.207	-0.924	0.239	0.308	0.803	0.297	0.102
DB07854	N-METHYL-1-[4-(9H-PURIN-6-YL)PHENYL]METHANAMINE	-0.316	-0.231	1.099	-0.207	-0.924	0.239	0.308	0.803	0.297	0.102
DB07855	(S)-1-PHENYL-1-[4-(9H-PURIN-6-YL)PHENYL]METHANAMINE	-0.316	-0.231	1.099	-0.207	-0.924	0.239	0.308	0.803	0.297	0.102
DB07857	(2R)-2-(4-chlorophenyl)-2-[4-(1H-pyrazol-4-yl)phenyl]ethanamine	-0.316	-0.231	1.099	-0.207	-0.924	0.239	0.308	0.803	0.297	0.102
DB07860	(2R)-2-(4-CHLOROPHENYL)-2-PHENYLETHANAMINE	-0.316	-0.231	1.099	-0.207	-0.924	0.239	0.308	0.803	0.297	0.102
DB07996	5-(2-methylpiperazine-1-sulfonyl)isoquinoline	-0.316	-0.231	1.099	-0.207	-0.924	0.239	0.308	0.803	0.297	0.102
DB07997	N-[2-(METHYLAMINO)ETHYL]-5-ISOQUINOLINESULFONAMIDE	-0.316	-0.231	1.099	-0.207	-0.924	0.239	0.308	0.803	0.297	0.102
DB08113	3-pyridin-4-yl-1H-indazole	-0.316	-0.231	1.099	-0.207	-0.924	0.239	0.308	0.803	0.297	0.102
DB08114	5-benzyl-1,3-thiazol-2-amine	-0.316	-0.231	1.099	-0.207	-0.924	0.239	0.308	0.803	0.297	0.102
DB01628	Etoricoxib	-0.315	-0.255	0.76	-0.481	-0.813	0.267	0.279	0.614	0.223	0.138
DB00347	Trimethadione	-0.313	-0.225	-0.726	-0.781	-0.433	0.294	0.31	0.245	0.246	0.266
DB00593	Ethosuximide	-0.313	-0.225	-0.726	-0.781	-0.433	0.294	0.31	0.245	0.246	0.266
DB05246	Methsuximide	-0.313	-0.225	-0.726	-0.781	-0.433	0.294	0.31	0.245	0.246	0.266
DB01255	Lisdexamfetamine	-0.311	-0.232	0.287	-0.276	-1.106	0.365	0.414	0.559	0.182	0.034
DB08065	2-(1H-pyrazol-3-yl)-1H-benzimidazole	-0.311	-0.257	-0.703	-0.614	-0.421	0.236	0.297	0.116	0.186	0.223
DB00651	Dyphylline	-0.31	0.056	0.076	-0.621	-0.227	0.342	0.464	0.394	0.145	0.318
DB02076	6-phospho-D-gluconic acid	-0.31	-0.325	1.069	0.092	-0.465	0.389	0.352	0.818	0.315	0.241
DB04861	Nebivolol	-0.309	0.347	-1.716	0.543	-1.185	0.347	0.578	0.015	0.593	0.073
DB03575	Phencyclidine	-0.307	0.054	0.414	-0.216	-1.017	0.351	0.592	0.572	0.209	0.056
DB07242	(4R)-7,8-dichloro-1',9-dimethyl-1-oxo-1,2,4,9-tetrahydrospiro[beta-carboline-3,4'-piperidine]-4-carbonitrile	-0.307	0.405	-2.315	0.728	-0.947	0.344	0.714	0.003	0.548	0.104
DB01165	Ofloxacin	-0.305	0.045	0.117	-1.165	-0.135	0.335	0.467	0.415	0.083	0.371
DB01472	4-Methoxyamphetamine	-0.305	0.076	1.26	-0.417	-1.023	0.376	0.657	0.873	0.159	0.038
DB01839	Propylene glycol	-0.302	-0.076	1.005	0.002	-0.86	0.386	0.522	0.788	0.29	0.079
DB00991	Oxaprozin	-0.301	0.022	2.19	-1.456	-1.017	0.367	0.583	0.958	0.021	0.056
DB01364	Ephedrine	-0.301	0.385	-1.264	0.306	-1.125	0.351	0.613	0.066	0.509	0.086
DB04813	Bithionol	-0.301	0.408	0.789	0.373	-1.209	0.27	0.62	0.723	0.59	0.092
DB05105	Pleconaril	-0.301	0	0.565	-0.087	-1.187	0.371	0.548	0.626	0.271	0.035
DB01050	Ibuprofen	-0.299	-0.582	2.485	1.328	-0.002	0.345	0.238	0.997	0.886	0.42
DB01404	Ginseng	-0.298	0.269	1.113	0.35	-1.488	0.333	0.628	0.792	0.388	0.029
DB00276	Amsacrine	-0.295	-0.455	-0.833	-1.174	0.688	0.272	0.245	0.143	0.087	0.724
DB00686	Pentosan Polysulfate	-0.295	0.25	0.973	0.169	-1.47	0.338	0.663	0.75	0.351	0.019
DB00754	Ethotoin	-0.295	-0.055	1.33	0.169	-1.041	0.359	0.497	0.843	0.336	0.057
DB03754	Tromethamine	-0.295	-0.132	-0.714	0.759	0.129	0.342	0.39	0.072	0.584	0.457
DB13169	Nandrolone	-0.295	-0.474	-1.101	-0.342	-0.033	0.236	0.226	0.069	0.264	0.37
DB04743	Nimesulide	-0.294	-0.399	1.447	-0.318	0.239	0.34	0.3	0.87	0.273	0.492
DB06736	Aceclofenac	-0.294	-0.768	1.818	-0.201	0.082	0.236	0.158	0.98	0.33	0.44

DB09526	Hydroquinone	-0.294	0.296	-0.394	-0.972	-0.322	0.277	0.545	0.25	0.124	0.288
DB00198	Oseltamivir	-0.289	-0.076	1.407	-0.246	-0.772	0.387	0.542	0.869	0.217	0.087
DB00790	Perindopril	-0.287	-0.144	0.735	-0.191	-0.478	0.441	0.507	0.734	0.224	0.237
DB06836	N-(5-{4-Chloro-3-[(2-hydroxyethyl)sulfamoyl]phenyl}-4-methyl-1,3-thiazol-2-yl)acetamide	-0.287	0.434	0.433	0.363	-1.18	0.349	0.718	0.556	0.383	0.039
DB00223	Diflorasone	-0.286	0.009	-1.495	-1.118	0.082	0.254	0.404	0.054	0.082	0.443
DB00591	Fluocinolone acetonide	-0.286	0.009	-1.495	-1.118	0.082	0.254	0.404	0.054	0.082	0.443
DB00846	Flurandrenolide	-0.286	0.009	-1.495	-1.118	0.082	0.254	0.404	0.054	0.082	0.443
DB01260	Desonide	-0.286	0.009	-1.495	-1.118	0.082	0.254	0.404	0.054	0.082	0.443
DB01410	Ciclesonide	-0.286	0.009	-1.495	-1.118	0.082	0.254	0.404	0.054	0.082	0.443
DB00745	Modafinil	-0.285	-0.134	1.895	0.599	-1.264	0.356	0.415	0.931	0.608	0.045
DB01003	Cromoglicic acid	-0.285	0.051	1.319	-0.166	-0.708	0.406	0.589	0.857	0.22	0.136
DB06413	Armodafinil	-0.285	-0.134	1.895	0.599	-1.264	0.356	0.415	0.931	0.608	0.045
DB06605	Apixaban	-0.285	-0.04	0.165	0.077	-0.577	0.387	0.543	0.483	0.314	0.188
DB07277	2-(5-CHLORO-2-THIENYL)-N-{(3S)-1-[(1S)-1-METHYL-2-MORPHOLIN-4-YL-2-OXOETHYL]-2-OXOPYRROLIDIN-3-YL}ETHANESULFONAMIDE	-0.285	-0.04	0.165	0.077	-0.577	0.387	0.543	0.483	0.314	0.188
DB07278	2-(5-CHLORO-2-THIENYL)-N-{(3S)-1-[(1S)-1-METHYL-2-MORPHOLIN-4-YL-2-OXOETHYL]-2-OXOPYRROLIDIN-3-YL}ETHENESULFONAMIDE	-0.285	-0.04	0.165	0.077	-0.577	0.387	0.543	0.483	0.314	0.188
DB07629	N-((1R,2S)-2-(5-CHLORO-1H-INDOLE-2-CARBOXAMIDO)CYCLOHEXYL)-5-METHYL-4,5,6,7-TETRAHYDROTHIAZOLO[5,4-C]PYRIDINE-2-CARBOXAMIDE	-0.285	-0.04	0.165	0.077	-0.577	0.387	0.543	0.483	0.314	0.188
DB07974	1-{2-[(4-CHLOROPHENYL)AMINO]-2-OXOETHYL}-N-(1-ISOPROPYLPIPERIDIN-4-YL)-1H-INDOLE-2-CARBOXAMIDE	-0.285	-0.04	0.165	0.077	-0.577	0.387	0.543	0.483	0.314	0.188
DB08174	5-CHLORO-N-((1R,2S)-2-(4-(2-OXOPYRIDIN-1(2H)-YL)BENZAMIDO) CYCLOPENTYL)THIOPHENE-2-CARBOXAMIDE	-0.285	-0.04	0.165	0.077	-0.577	0.387	0.543	0.483	0.314	0.188
DB11166	Antithrombin Alfa	-0.285	-0.04	0.165	0.077	-0.577	0.387	0.543	0.483	0.314	0.188
DB01013	Clobetasol propionate	-0.284	0.002	-1.244	-0.922	0.023	0.289	0.404	0.075	0.116	0.417
DB01047	Fluocinonide	-0.283	0.081	-1.38	-1.393	0.153	0.246	0.448	0.062	0.048	0.459
DB04209	Dequalinium	-0.282	-0.585	-1.447	-0.084	0.42	0.255	0.16	0.057	0.404	0.614
DB09131	Cupric Chloride	-0.282	-0.164	1.522	-0.259	-0.558	0.479	0.511	0.893	0.2	0.197
DB06716	Fospropofol	-0.281	-0.198	0.719	0.749	-0.602	0.422	0.461	0.759	0.778	0.169
DB00480	Lenalidomide	-0.277	-0.625	-1.623	0.611	-0.189	0.335	0.23	0.027	0.588	0.355
DB00973	Ezetimibe	-0.277	-0.094	-0.044	0.399	-0.707	0.364	0.446	0.356	0.528	0.152
DB03152	B-2-Octylglucoside	-0.276	-0.095	0.619	-0.292	-0.587	0.496	0.573	0.727	0.222	0.178
DB04931	Afamelanotide	-0.276	0.406	0.732	-0.355	-0.125	0.429	0.785	0.731	0.175	0.467
DB00798	Gentamicin	-0.275	0.006	0.414	0.444	-0.857	0.378	0.532	0.552	0.441	0.1
DB09517	Sodium ferric gluconate complex	-0.275	0.637	1.043	0.08	-1.07	0.347	0.684	0.756	0.353	0.12
DB00054	Abciximab	-0.271	-0.339	0.217	0.545	0.786	0.35	0.308	0.422	0.645	0.731
DB01158	Bretylium	-0.271	0.474	-1.632	0.708	-1.013	0.351	0.631	0.022	0.644	0.099
DB07288	N-(4-chlorophenyl)-2-[(pyridin-4-ylmethyl)amino]benzamide	-0.271	0.217	-0.217	-0.218	-0.902	0.25	0.528	0.248	0.343	0.097
DB06218	Lacosamide	-0.27	0.607	2.198	-0.902	-0.948	0.36	0.688	0.972	0.137	0.128
DB04879	Vatalanib	-0.269	0.138	-0.071	0.347	-0.949	0.27	0.513	0.352	0.562	0.11
DB00171	ATP	-0.268	-1.06	0.823	0.838	0.279	0.299	0.112	0.748	0.796	0.565
DB02398	6-[N-(4-(Aminomethyl)Phenyl)Carbamyl]-2-Naphthalenecarboxamidine	-0.267	-0.058	0.974	0.102	-0.279	0.403	0.464	0.76	0.311	0.322
DB03535	Z-Pro-Prolinal	-0.267	0.029	0.007	-0.322	-0.554	0.477	0.664	0.414	0.166	0.198

DB06925	3-(2-AMINOQUINAZOLIN-6-YL)-4-METHYL-N-[3-(TRIFLUOROMETHYL)PHENYL]BENZAMIDE	-0.267	0.64	0.015	0.078	-1.221	0.36	0.707	0.172	0.398	0.068
DB00559	Bosentan	-0.264	-0.482	1.49	0.679	-0.753	0.353	0.264	0.861	0.495	0.161
DB01154	Thiamylal	-0.264	0.039	1.962	0.286	-0.921	0.442	0.633	0.934	0.574	0.05
DB03345	Mercaptoethanol	-0.264	-0.225	0.188	0.284	-0.343	0.4	0.394	0.455	0.356	0.29
DB01765	(5-Oxo-5,6-Dihydro-Indolo[1,2-a]Quinazolin-7-Yl)-Acetic Acid	-0.263	-0.691	-1.036	-0.717	-0.595	0.254	0.177	0.07	0.159	0.157
DB04066	para-Coumaric Acid	-0.263	-0.142	0.217	0.202	-1.128	0.314	0.339	0.476	0.5	0.089
DB01067	Glipizide	-0.261	0.337	1.185	1.569	-1.198	0.348	0.6	0.804	0.908	0.067
DB00032	Menotropins	-0.26	0.107	-0.057	-0.202	-0.701	0.449	0.699	0.387	0.233	0.116
DB00097	Choriogonadotropin alfa	-0.26	0.107	-0.057	-0.202	-0.701	0.449	0.699	0.387	0.233	0.116
DB05039	Indacaterol	-0.26	0.264	0.13	0.708	-1.329	0.363	0.544	0.254	0.658	0.063
DB01328	Cefonicid	-0.259	0.338	0.074	0.346	-0.994	0.353	0.628	0.36	0.398	0.089
DB04214	4-Nitrophenyl Phosphate	-0.259	0.157	1.967	-0.8	-1.327	0.368	0.528	0.934	0.114	0.061
DB01148	Flavoxate	-0.255	0.01	0.398	-0.277	-0.73	0.487	0.64	0.626	0.186	0.103
DB04001	6-(Oxalyl-Amino)-1h-Indole-5-Carboxylic Acid	-0.254	0.268	-0.178	-0.271	-0.704	0.376	0.638	0.12	0.253	0.149
DB01138	Sulfinpyrazone	-0.253	-0.411	2.722	-0.55	-0.445	0.363	0.294	0.985	0.165	0.257
DB04894	Vapreotide	-0.253	-0.015	-0.083	-0.031	-0.557	0.415	0.541	0.353	0.274	0.182
DB04306	5-[(4-Methylphenyl)Sulfanyl]-2,4-Quinazolinediamine	-0.252	0.61	0.363	1.027	-1.806	0.376	0.742	0.502	0.796	0.016
DB07821	(1R)-1,2,2-trimethylpropyl (R)-methylphosphinate	-0.252	-0.136	0.34	-0.197	-0.446	0.458	0.54	0.575	0.229	0.253
DB01397	Magnesium salicylate	-0.25	0.416	2.113	-2.279	-1.576	0.352	0.627	0.951	0.01	0.022
DB01398		-0.25	0.416	2.113	-2.279	-1.576	0.352	0.627	0.951	0.01	0.022
DB01892	Hyperforin	-0.25	-0.112	2.222	-0.691	-0.779	0.367	0.426	0.961	0.134	0.146
DB07876	(S)-2-METHYL-1-[(4-METHYL-5-ISOQUINOLINE)SULFONYL]-HOMOPIPERAZINE	-0.25	-0.536	0.889	-0.849	-0.752	0.258	0.197	0.605	0.137	0.13
DB07557	(5BETA)-PREGNANE-3,20-DIONE	-0.249	0.487	0.943	-0.225	-0.964	0.365	0.63	0.722	0.331	0.113
DB00912	Repaglinide	-0.248	0.24	1.344	1.857	-1.265	0.356	0.572	0.851	0.962	0.053
DB08868	Fingolimod	-0.247	-0.373	0.568	0.171	0.033	0.252	0.233	0.538	0.46	0.389
DB03338	Heptyl-Beta-D-Glucopyranoside	-0.244	0.191	1.614	0.459	-0.914	0.395	0.603	0.905	0.421	0.098
DB01767	Hemi-Babim	-0.242	-0.526	0.512	0.614	0.124	0.386	0.24	0.562	0.463	0.441
DB07234	3-{[(1R)-1-phenylethyl]amino}-4-(pyridin-4-ylamino)cyclobut-3-ene-1,2-dione	-0.242	0.206	0.326	-1.893	-0.261	0.273	0.512	0.513	0.017	0.301
DB00071	Insulin Pork	-0.239	-0.864	-1.277	0.788	1.271	0.352	0.145	0.072	0.731	0.877
DB01202	Levetiracetam	-0.238	0.059	0.467	-0.455	-0.899	0.468	0.613	0.628	0.162	0.079
DB01421	Paromomycin	-0.238	-0.1	1.184	0.535	-0.417	0.525	0.594	0.861	0.582	0.282
DB03856	L-Eflornithine	-0.238	-0.1	1.184	0.535	-0.417	0.525	0.594	0.861	0.582	0.282
DB04816	Dantron	-0.238	-0.1	1.184	0.535	-0.417	0.525	0.594	0.861	0.582	0.282
DB08972	Flumequine	-0.238	-0.1	1.184	0.535	-0.417	0.525	0.594	0.861	0.582	0.282
DB02116	Olomoucine	-0.237	0.355	-0.58	-0.925	-0.415	0.283	0.573	0.192	0.121	0.237
DB00359	Sulfadiazine	-0.236	-0.312	0.312	0.15	-0.654	0.281	0.287	0.465	0.473	0.16
DB06831	2-((9H-PURIN-6-YLTHIO)METHYL)-5-CHLORO-3-(2-METHOXYPHENYL)QUINAZOLIN-4(3H)-ONE	-0.236	0.054	0.133	-0.023	-0.597	0.444	0.616	0.462	0.286	0.177
DB08142	AT-7519	-0.236	-0.544	-0.726	-0.195	0.332	0.329	0.225	0.193	0.36	0.556
DB00249	Idoxuridine	-0.234	-0.397	0.672	0.704	0.053	0.444	0.294	0.663	0.656	0.575
DB01265	Telbivudine	-0.234	-0.397	0.672	0.704	0.053	0.444	0.294	0.663	0.656	0.575
DB03312	Brivudine	-0.234	-0.397	0.672	0.704	0.053	0.444	0.294	0.663	0.656	0.575
DB01132	Pioglitazone	-0.233	0.261	0.612	0.007	-0.771	0.371	0.536	0.556	0.42	0.164
DB03203	Sphingosine	-0.233	-0.017	-0.067	-0.071	-0.365	0.512	0.624	0.378	0.318	0.301
DB00150	L-Tryptophan	-0.232	-0.681	1.827	1.456	0.181	0.361	0.206	0.93	0.887	0.496
DB09301	Chondroitin sulfate	-0.232	-0.136	-0.513	0.66	0.146	0.366	0.385	0.065	0.511	0.473

DB00600	Monobenzone	-0.23	0.159	1.164	-1.405	-1.002	0.421	0.628	0.803	0.034	0.055
DB02744	RPR131247	-0.23	-0.069	0.792	-0.533	-0.308	0.355	0.401	0.677	0.243	0.301
DB01426	Ajmaline	-0.229	0.046	0.602	-0.04	-0.846	0.437	0.56	0.67	0.275	0.104
DB07859	4-(4-CHLOROPHENYL)-4-[4-(1H-PYRAZOL-4-YL)PHENYL]PIPERIDINE	-0.228	-0.434	1.46	0.136	-0.138	0.32	0.219	0.947	0.462	0.325
DB07947	ISOQUINOLINE-5-SULFONIC ACID (2-(2-(4-CHLOROBENZYLOXY)ETHYLAMINO)ETHYL)AMIDE	-0.228	-0.434	1.46	0.136	-0.138	0.32	0.219	0.947	0.462	0.325
DB08148	1-[4-(4-chlorophenyl)-1-(7H-pyrrolo[2,3-d]pyrimidin-4-yl)piperidin-4-yl]methanamine	-0.228	-0.434	1.46	0.136	-0.138	0.32	0.219	0.947	0.462	0.325
DB08149	1-[4-(4-chlorobenzyl)-1-(7H-pyrrolo[2,3-d]pyrimidin-4-yl)piperidin-4-yl]methanamine	-0.228	-0.434	1.46	0.136	-0.138	0.32	0.219	0.947	0.462	0.325
DB02431	Cytidine-5'-Triphosphate	-0.226	0.16	0.329	-0.505	-0.855	0.564	0.761	0.761	0.144	0.143
DB03367	PF-00356231	-0.224	-0.183	0.021	0.527	0.123	0.375	0.372	0.341	0.466	0.456
DB02761	S-Mercaptocysteine	-0.223	0.378	0.673	0.931	-1.552	0.369	0.661	0.614	0.62	0.018
DB03851	Carbazole Butanoic Acid	-0.223	-0.048	1.795	0.012	-0.682	0.422	0.487	0.925	0.274	0.162
DB06938	4-[[2-[[4-chloro-3-(trifluoromethyl)phenyl]amino]-3H-benzimidazol-5-yl]oxy]-N-methyl-pyridine-2-carboxamide	-0.223	-0.555	1.265	0.403	0.095	0.367	0.225	0.841	0.525	0.46
DB00573	Fenoprofen	-0.222	-0.025	1.608	0.856	-1.027	0.34	0.434	0.908	0.772	0.128
DB01884	2-Amino-3-Methyl-1-Pyrrolidin-1-Yl-Butan-1-One	-0.221	0.128	-0.662	-0.076	-0.403	0.492	0.681	0.036	0.256	0.275
DB04459	3,4-Dichloroisocoumarin	-0.221	-0.027	0.359	0.109	-0.496	0.393	0.515	0.531	0.316	0.221
DB07666	(3R,4S)-1-{6-[3-(METHYLSULFONYL)PHENYL]PYRIMIDIN-4-YL}-4-(2,4,5-TRIFLUOROPHENYL)PYRROLIDIN-3-AMINE	-0.221	0.128	-0.662	-0.076	-0.403	0.492	0.681	0.036	0.256	0.275
DB07901	5-CHLORO-6-METHYL-N-(2-PHENYLETHYL)-2-PYRIDIN-2-YLPYRIMIDIN-4-AMINE	-0.221	-0.014	1.023	0.028	-1.089	0.425	0.55	0.807	0.31	0.039
DB08445	(3R,4S)-1-[6-(6-METHOXYPYRIDIN-3-YL)PYRIMIDIN-4-YL]-4-(2,4,5-TRIFLUOROPHENYL)PYRROLIDIN-3-AMINE	-0.221	0.128	-0.662	-0.076	-0.403	0.492	0.681	0.036	0.256	0.275
DB01077	Etidronic acid	-0.22	0.137	-1.198	0.891	-1.101	0.392	0.526	0.081	0.613	0.087
DB03106	scyllo-inositol	-0.219	0.502	1.26	0.104	-1.626	0.425	0.808	0.832	0.32	0.005
DB03760	Dihydrolipoic Acid	-0.219	0.596	2.035	0.38	-2.27	0.392	0.772	0.943	0.383	0.001
DB01645	Genistein	-0.217	0.668	-0.781	-0.829	-0.857	0.281	0.727	0.172	0.156	0.117
DB02959	Oxitriptan	-0.214	-0.386	0.702	1.321	-0.84	0.379	0.28	0.614	0.784	0.156
DB08063	1-BENZYL-3-(4-METHOXYPHENYLAMINO)-4-PHENYLPYRROLE-2,5-DIONE	-0.214	0.743	0.184	-0.607	0.208	0.374	0.729	0.188	0.206	0.498
DB06908	(2S)-3-(1-{[2-(2-CHLOROPHENYL)-5-METHYL-1,3-OXAZOL-4-YL]METHYL}-1H-INDOL-5-YL)-2-ETHOXYPROPANOIC ACID	-0.213	-0.115	1.169	1.371	-1.279	0.366	0.395	0.804	0.791	0.061
DB06779	Dalteparin	-0.211	-0.303	1.286	0.52	0.002	0.387	0.33	0.824	0.429	0.42
DB07348	Brefeldin A	-0.211	0.17	1.255	-0.863	-0.607	0.454	0.616	0.866	0.086	0.173
DB01072	Atazanavir	-0.21	0.323	0.723	0.073	-1.215	0.376	0.577	0.689	0.327	0.08
DB05360		-0.21	0.426	-0.633	0.335	-0.762	0.41	0.729	0.131	0.503	0.132
DB06834	N-(2-hydroxy-1,1-dimethylethyl)-1-methyl-3-(1H-pyrrolo[2,3-b]pyridin-2-yl)-1H-indole-5-carboxamide	-0.21	-0.261	0.173	0.477	0.112	0.379	0.34	0.183	0.549	0.445
DB00712	Flurbiprofen	-0.209	0.364	1.114	-0.966	-1.118	0.374	0.616	0.792	0.087	0.089

DB06554	Gaboxadol	-0.209	-0.098	2.168	-0.34	-0.578	0.482	0.477	0.951	0.195	0.175
DB00782	Propantheline	-0.208	0.582	-0.189	-0.812	-0.677	0.359	0.67	0.191	0.149	0.183
DB00033	Interferon gamma-1b	-0.207	0.158	0.843	-0.145	-0.862	0.478	0.732	0.756	0.247	0.069
DB01408	Bambuterol	-0.207	0.414	-1.941	0.36	-1.195	0.381	0.613	0.007	0.528	0.071
DB02552	Geranyl Diphosphate	-0.205	0.2	0.196	0.037	-0.595	0.491	0.717	0.482	0.287	0.167
DB04714	ISOPENTENYL PYROPHOSPHATE	-0.205	0.2	0.196	0.037	-0.595	0.491	0.717	0.482	0.287	0.167
DB06711	Naphazoline	-0.205	0.001	2.537	0.382	-1.325	0.376	0.446	0.975	0.369	0.054
DB06830	(1-HYDROXYHEPTANE-1,1-DIYL)BIS(PHOSPHONIC ACID)	-0.205	0.2	0.196	0.037	-0.595	0.491	0.717	0.482	0.287	0.167
DB06931	(1-HYDROXYNONANE-1,1-DIYL)BIS(PHOSPHONIC ACID)	-0.205	0.2	0.196	0.037	-0.595	0.491	0.717	0.482	0.287	0.167
DB07873	Lauryl alcohol diphosphonic acid	-0.205	0.2	0.196	0.037	-0.595	0.491	0.717	0.482	0.287	0.167
DB08826	Deferiprone	-0.204	-0.017	0.878	0.251	-0.477	0.602	0.692	0.886	0.527	0.247
DB03044	1-(5-Tert-Butyl-2-P-Tolyl-2h-Pyrazol-3-Yl)-3-[4-(2-Morpholin-4-Yl-Ethoxy)-Naphthalen-1-Yl]-Urea	-0.202	0.194	1.193	-0.388	0.019	0.334	0.51	0.85	0.259	0.451
DB00621	Oxandrolone	-0.2	-0.091	-1.06	-0.589	0.321	0.333	0.419	0.075	0.159	0.614
DB00858	Drostanolone	-0.2	-0.091	-1.06	-0.589	0.321	0.333	0.419	0.075	0.159	0.614
DB01606	Tazobactam	-0.2	-0.091	-1.06	-0.589	0.321	0.333	0.419	0.075	0.159	0.614
DB01783	Pantothenic acid	-0.2	-0.091	-1.06	-0.589	0.321	0.333	0.419	0.075	0.159	0.614
DB04823	Oxyphenisatin	-0.2	-0.091	-1.06	-0.589	0.321	0.333	0.419	0.075	0.159	0.614
DB05016	Ataluren	-0.2	-0.091	-1.06	-0.589	0.321	0.333	0.419	0.075	0.159	0.614
DB05288	Anecortave acetate	-0.2	-0.091	-1.06	-0.589	0.321	0.333	0.419	0.075	0.159	0.614
DB06811	Polidocanol	-0.2	-0.091	-1.06	-0.589	0.321	0.333	0.419	0.075	0.159	0.614
DB08804	Nandrolone decanoate	-0.2	-0.091	-1.06	-0.589	0.321	0.333	0.419	0.075	0.159	0.614
DB08899	Enzalutamide	-0.2	-0.091	-1.06	-0.589	0.321	0.333	0.419	0.075	0.159	0.614
DB09015	Canrenoic acid	-0.2	-0.091	-1.06	-0.589	0.321	0.333	0.419	0.075	0.159	0.614
DB11371	Alfaxalone	-0.2	-0.091	-1.06	-0.589	0.321	0.333	0.419	0.075	0.159	0.614
DB11921	Deflazacort	-0.2	-0.091	-1.06	-0.589	0.321	0.333	0.419	0.075	0.159	0.614
DB13155	Esculin	-0.2	-0.091	-1.06	-0.589	0.321	0.333	0.419	0.075	0.159	0.614
DB00127	Spermine	-0.199	0.513	-0.859	1.609	-1.458	0.381	0.648	0.113	0.918	0.054
DB00205	Pyrimethamine	-0.199	0.215	0.625	-0.659	-0.397	0.302	0.511	0.589	0.195	0.235
DB04590	(2R)-({4-[AMINO(IMINO)METHYL]PHENYL}AMINO){5-ETHOXY-2-FLUORO-3-[(3R)-TETRAHYDROFURAN-3-YLOXY]PHENYL}ACETICACID	-0.199	-0.082	0.406	0.404	-0.231	0.399	0.421	0.54	0.389	0.33
DB06626	Axitinib	-0.199	-0.46	0.16	0.492	-0.21	0.34	0.243	0.512	0.679	0.306
DB04326	Dihydroxyacetone phosphate	-0.198	-0.038	0.709	-0.359	-1.326	0.392	0.447	0.645	0.228	0.057
DB08885	Aflibercept	-0.198	0.14	0.629	0.034	-0.252	0.442	0.59	0.668	0.297	0.339
DB00118	Ademetionine	-0.197	-0.632	-1.148	0.891	0.524	0.381	0.216	0.048	0.689	0.596
DB02264	O2-Sulfo-Glucuronic Acid	-0.196	0.391	1.039	0.233	-0.983	0.506	0.788	0.806	0.489	0.039
DB00030	Insulin Human	-0.195	-0.771	-0.801	0.653	1.278	0.379	0.191	0.127	0.7	0.877
DB02440		-0.195	-0.157	0.976	-0.16	-0.36	0.303	0.381	0.734	0.312	0.228
DB07766	(2Z,3E)-2,3'-BIINDOLE-2',3(1H,1'H)-DIONE 3-{O-[(3R)-3,4-DIHYDROXYBUTYL]OXIME}	-0.194	-0.387	0.049	1.146	0.355	0.383	0.313	0.169	0.708	0.574
DB00481	Raloxifene	-0.193	0.592	1.212	0.628	-0.545	0.342	0.693	0.852	0.706	0.223
DB01325	Quinethazone	-0.193	1.053	1.886	-1.184	-2.285	0.397	0.868	0.928	0.064	0.005
DB04899	Nesiritide	-0.192	0.234	-0.397	-0.313	-0.7	0.557	0.773	0.226	0.189	0.114
DB00112	Bevacizumab	-0.19	-0.552	0.156	-0.14	1.317	0.385	0.245	0.392	0.346	0.881
DB00507	Nitazoxanide	-0.19	0.16	0.264	0.296	-0.628	0.434	0.588	0.491	0.38	0.166
DB06213	Regadenoson	-0.189	0.392	-0.975	-0.219	-0.729	0.465	0.77	0.016	0.195	0.117
DB06786	Halcinonide	-0.189	0.236	-1.311	-1.581	0.463	0.289	0.522	0.072	0.045	0.627
DB08770	4-{2-[(7-amino-2-furan-2-yl[1,2,4]triazolo[1,5-a][1,3,5]triazin-5-yl)amino]ethyl}phenol	-0.189	0.392	-0.975	-0.219	-0.729	0.465	0.77	0.016	0.195	0.117
DB03435	Uridine-5'-Diphosphate	-0.188	0.274	-0.824	-0.161	-1.014	0.393	0.63	0.105	0.237	0.107
DB00467	Enoxacin	-0.187	0.417	0.911	-1.57	-0.292	0.371	0.601	0.712	0.036	0.307

DB07768	Epitestosterone	-0.186	-0.144	-1.473	-0.43	-0.168	0.303	0.348	0.059	0.258	0.315
DB08818	Hyaluronic acid	-0.185	0.799	1.224	-0.937	-2.263	0.385	0.756	0.837	0.126	0.014
DB00498	Phenindione	-0.178	0.499	-2.339	0.645	-0.244	0.444	0.784	0.002	0.529	0.35
DB00324	Fluorometholone	-0.177	0.149	-1.25	-1.046	-0.043	0.319	0.474	0.081	0.101	0.399
DB02377	Guanine	-0.177	-0.12	1.225	-1.135	-1.003	0.468	0.479	0.842	0.052	0.052
DB00735	Naftifine	-0.175	0.105	0.16	0.162	-0.477	0.425	0.548	0.459	0.312	0.218
DB00132	Alpha-Linolenic Acid	-0.174	0.119	0.571	0.679	-0.299	0.384	0.489	0.582	0.695	0.31
DB07340	N~6~-cyclohexyl-N~2~-(4-morpholin-4-ylphenyl)-9H-purine-2,6-diamine	-0.174	-0.139	-0.522	-0.219	-0.249	0.316	0.358	0.144	0.337	0.284
DB00627	Niacin	-0.173	0.059	0.525	-0.29	-0.487	0.646	0.693	0.662	0.216	0.237
DB00062	Human Serum Albumin	-0.17	0.143	0.17	0.401	-0.546	0.439	0.577	0.438	0.43	0.19
DB00064		-0.17	0.143	0.17	0.401	-0.546	0.439	0.577	0.438	0.43	0.19
DB02733	Purvalanol	-0.17	0.057	0.514	-0.619	-0.573	0.296	0.425	0.625	0.192	0.174
DB00779	Nalidixic acid	-0.168	-0.026	1.113	0.689	-0.324	0.502	0.553	0.807	0.589	0.337
DB09129	Chromic chloride	-0.168	0.427	1.029	0.604	-1.231	0.288	0.608	0.613	0.665	0.054
DB01055	Mimosine	-0.167	0.019	0.065	0.161	-0.439	0.465	0.527	0.394	0.348	0.237
DB06791	Lanreotide	-0.167	0.103	0.166	-0.193	-0.738	0.467	0.613	0.488	0.205	0.116
DB09099	Somatostatin	-0.167	0.103	0.166	-0.193	-0.738	0.467	0.613	0.488	0.205	0.116
DB09112	Nitrous acid	-0.167	1.239	0.855	-1.219	-1.419	0.385	0.866	0.689	0.081	0.058
DB08378	4-[4-(2,5-DIOXO-PYRROLIDIN-1-YL)-PHENYLAMINO]-4-HYDROXY-BUTYRIC ACID	-0.165	0.015	0.805	-0.392	-1.392	0.383	0.475	0.681	0.195	0.051
DB00233	Aminosalicylic Acid	-0.164	-0.34	-0.712	1.107	-1.153	0.317	0.282	0.168	0.832	0.09
DB02506	2,6,8-Trimethyl-3-Amino-9-Benzyl-9-Methoxynonanoic Acid	-0.163	0.121	-0.186	-0.59	-0.314	0.318	0.476	0.249	0.216	0.258
DB07121	4-({4-[(4-AMINOBUT-2-YNYL)OXY]PHENYL}SULFONYL)-N-HYDROXY-2,2-DIMETHYLTHIOMORPHOLINE-3-CARBOXAMIDE	-0.163	0.273	0.091	0.082	-0.819	0.487	0.708	0.418	0.293	0.108
DB08811	Tofisopam	-0.162	0.474	0.25	-0.313	-1.343	0.49	0.812	0.538	0.185	0.023
DB11605	Myrrh	-0.162	0.355	0.813	0.825	-1.365	0.411	0.588	0.667	0.579	0.06
DB01708	Prasterone	-0.161	0.498	-0.427	0.01	-0.42	0.325	0.656	0.28	0.442	0.246
DB03193	Stearic acid	-0.161	-0.024	2.034	1.356	-1.425	0.389	0.437	0.979	0.794	0.047
DB07858	(2S)-2-(4-chlorophenyl)-2-[4-(1H-pyrazol-4-yl)phenyl]ethanamine	-0.161	0.076	1.224	-0.115	-0.451	0.291	0.431	0.856	0.33	0.189
DB08070	2-[4-(3-METHYL-1H-PYRAZOL-4-YL)PHENYL]ETHANAMINE	-0.161	0.076	1.224	-0.115	-0.451	0.291	0.431	0.856	0.33	0.189
DB00278	Argatroban	-0.159	-0.246	0.697	-0.449	0.379	0.414	0.349	0.628	0.18	0.538
DB08815	Lurasidone	-0.157	-0.244	2.586	0.82	-1.545	0.397	0.337	0.981	0.597	0.04
DB00346	Alfuzosin	-0.154	-0.09	1.565	0.138	-1.213	0.463	0.456	0.893	0.298	0.045
DB04795	Thenoyltrifluoroacetone	-0.154	0.13	1.09	0.255	-0.875	0.518	0.666	0.808	0.415	0.082
DB00325	Nitroprusside	-0.152	0.156	-0.12	-0.159	-0.419	0.549	0.663	0.331	0.225	0.273
DB02354	4-{[1-Methyl-5-(2-Methyl-Benzoimidazol-1-Ylmethyl)-1h-Benzoimidazol-2-Ylmethyl]-Amino}-Benzamidine	-0.15	0.281	-0.361	-0.12	-0.553	0.388	0.564	0.107	0.284	0.226
DB04107	[(1-{2[(4-Carbamimidoyl-Phenylamino)-Methyl]-1-Methyl-1h-Benzoimidazol-5-Yl}-Cyclopropyl)-Pyridin-2-Yl-Methyleneaminooxy]-Acetic Acid Ethyl Ester	-0.15	0.281	-0.361	-0.12	-0.553	0.388	0.564	0.107	0.284	0.226
DB06414	Etravirine	-0.15	-0.087	-0.051	-0.724	0.056	0.406	0.404	0.3	0.128	0.429
DB07191		-0.15	-0.087	-0.051	-0.724	0.056	0.406	0.404	0.3	0.128	0.429
DB08905	Formestane	-0.15	-0.103	-1.195	-0.634	0.411	0.318	0.38	0.066	0.17	0.626
DB07186	4-(4-METHYLPIPERAZIN-1-YL)-N-[5-(2-THIENYLACETYL)-1,5-DIHYDROPYRROLO[3,4-C]PYRAZOL-3-YL]BENZAMIDE	-0.149	-0.532	0.89	-0.208	0.267	0.322	0.184	0.542	0.311	0.51

DB09276	Sodium aurothiomalate	-0.148	0.407	0.818	0.343	-1.013	0.342	0.605	0.581	0.575	0.091
DB00673	Aprepitant	-0.147	0.033	-0.155	-0.081	-0.338	0.542	0.607	0.314	0.27	0.302
DB04705	(3BETA)-CHOLEST-5-ENE-3,25-DIOL	-0.147	0.266	0.98	1.125	-1.434	0.395	0.548	0.751	0.704	0.053
DB01608	Periciazine	-0.144	-0.02	-1.33	-0.488	0.096	0.299	0.391	0.047	0.211	0.431
DB00797	Tolazoline	-0.142	0.32	2.739	0.222	-0.812	0.474	0.696	0.979	0.489	0.098
DB08908	Dimethyl fumarate	-0.142	-0.063	0.629	-0.972	0.436	0.305	0.384	0.392	0.109	0.595
DB00393	Nimodipine	-0.14	1.099	1.603	-0.055	-1.486	0.362	0.879	0.926	0.371	0.058
DB00038	Oprelvekin	-0.134	-0.168	0.087	-0.104	-0.314	0.33	0.318	0.39	0.35	0.303
DB00174	Asparagine	-0.134	0.712	0.238	-0.05	-1.051	0.407	0.72	0.417	0.332	0.109
DB06700	Desvenlafaxine	-0.134	0.208	0.37	-0.387	-0.61	0.587	0.734	0.614	0.163	0.164
DB06701	Dexmethylphenidate	-0.134	0.208	0.37	-0.387	-0.61	0.587	0.734	0.614	0.163	0.164
DB08544	(S)-Fluoxetine	-0.134	0.208	0.37	-0.387	-0.61	0.587	0.734	0.614	0.163	0.164
DB08918	Levomilnacipran	-0.134	0.208	0.37	-0.387	-0.61	0.587	0.734	0.614	0.163	0.164
DB09185	Viloxazine	-0.134	0.208	0.37	-0.387	-0.61	0.587	0.734	0.614	0.163	0.164
DB00095	Efalizumab	-0.131	-0.243	0.065	-0.196	1.242	0.395	0.338	0.357	0.349	0.853
DB03041	UDP-alpha-D-glucuronic acid	-0.131	0.111	1.082	0.014	-0.571	0.526	0.62	0.802	0.27	0.195
DB01395	Drospirenone	-0.13	0.099	-0.859	-0.608	-0.145	0.339	0.447	0.087	0.174	0.315
DB01105	Sibutramine	-0.129	1.484	-0.539	-1.37	-0.432	0.402	0.926	0.171	0.071	0.259
DB01211	Clarithromycin	-0.129	0.202	0.051	0.076	-0.582	0.513	0.659	0.392	0.292	0.19
DB02007	alpha-D-glucose 6-phosphate	-0.129	-0.421	1.85	0.832	-0.808	0.399	0.285	0.912	0.59	0.144
DB06176	Romidepsin	-0.128	0.054	0.687	-0.563	0.148	0.323	0.428	0.617	0.241	0.497
DB11594	Domiphen	-0.128	0.505	1.236	0.144	-1.338	0.508	0.822	0.852	0.323	0.021
DB02660	Filaminast	-0.125	-0.39	0.38	0.276	-0.422	0.339	0.252	0.331	0.479	0.228
DB00707	Porfimer sodium	-0.123	0.382	0.191	0.048	-0.898	0.537	0.782	0.477	0.305	0.083
DB01134	Desoxycorticosterone pivalate	-0.123	0.059	-1.116	-0.456	0.063	0.326	0.458	0.067	0.216	0.41
DB08860	Pitavastatin	-0.123	-0.137	-1.116	-0.456	0.623	0.326	0.363	0.067	0.216	0.701
DB01089	Deserpidine	-0.122	0.006	-1.293	-0.62	0.412	0.339	0.47	0.051	0.159	0.619
DB03444	(3e)-6'-Bromo-2,3'-Biindole-2',3(1h,1'h)-Dione 3-Oxime	-0.12	0.397	-1.461	0.055	-0.599	0.334	0.612	0.051	0.478	0.171
DB06694	Xylometazoline	-0.119	-0.495	3.37	0.838	-0.844	0.406	0.25	0.997	0.698	0.136
DB08079	AMG-208	-0.119	0.019	0.549	0.815	-0.188	0.502	0.557	0.575	0.605	0.401
DB04233	(Hydroxyethyloxy)Tri(Ethyloxy)Octane	-0.118	0.919	0.706	-0.403	-0.866	0.419	0.781	0.628	0.244	0.133
DB08564	(2E)-N-{4-[(3-bromophenyl)amino]quinazolin-6-yl}-4-(dimethylamino)but-2-enamide	-0.118	0.51	1.084	0.104	-1.015	0.316	0.65	0.643	0.457	0.08
DB00630	Alendronic acid	-0.115	0.053	-0.908	1.416	-1.058	0.411	0.468	0.126	0.831	0.116
DB01834	(9R,10R)-9-(S-glutathionyl)-10-hydroxy-9,10-dihydrophenanthrene	-0.114	0.008	0.798	-0.891	-0.91	0.596	0.619	0.752	0.086	0.071
DB04690	Camptothecin	-0.113	1.451	0.843	-3.625	-0.578	0.315	0.95	0.507	0.004	0.153
DB00040	Glucagon	-0.112	0.163	-0.386	-0.213	-0.402	0.593	0.693	0.196	0.251	0.277
DB00397	Phenylpropanolamine	-0.112	-0.137	0.28	0.912	-1.39	0.407	0.376	0.481	0.744	0.068
DB11363	Alectinib	-0.112	0.027	1.073	-0.389	-0.93	0.404	0.476	0.796	0.285	0.131
DB06262	Droxidopa	-0.11	0.071	0.944	1.151	-1.326	0.421	0.473	0.741	0.826	0.063
DB00924	Cyclobenzaprine	-0.108	-0.535	-0.268	1.143	-0.802	0.408	0.251	0.261	0.823	0.162
DB07970	5-[(2-methyl-5-{[3-(trifluoromethyl)phenyl]carbamoyl}phenyl)amino]pyridine-3-carboxamide	-0.107	0.062	0.428	0.293	-0.366	0.503	0.556	0.551	0.383	0.275
DB08067	4-[(2-{4-[(CYCLOPROPYLCARBAMOYL)AMINO]-1H-PYRAZOL-3-YL}-1H-BENZIMIDAZOL-6-YL)METHYL]MORPHOLIN-4-IUM	-0.106	-0.232	0.327	-0.866	0.271	0.352	0.34	0.526	0.157	0.545
DB09286	Pipamperone	-0.106	-0.452	1.495	1.532	-0.062	0.423	0.265	0.893	0.932	0.39
DB08577	3-[(3-(2-CARBOXYETHYL)-4-METHYLPYRROL-2-YL)METHYLENE]-2-INDOLINONE	-0.104	0.698	0.953	-1.175	-0.826	0.454	0.781	0.742	0.071	0.122

DB08912	Dabrafenib	-0.103	-0.708	0.53	2.538	-0.297	0.415	0.199	0.407	0.983	0.322
DB08300	1-methyl-3-naphthalen-2-yl-1H-pyrazolo[3,4-d]pyrimidin-4-amine	-0.101	-0.096	0.48	-0.403	-0.128	0.35	0.347	0.55	0.285	0.362
DB00449	Dipivefrin	-0.1	0.75	-0.177	0.461	-1.389	0.444	0.735	0.204	0.561	0.051
DB00536	Guanidine	-0.098	0.104	1.315	1.169	-1.378	0.417	0.48	0.834	0.751	0.055
DB07335	3-[4-AMINO-1-(1-METHYLETHYL)-1H-PYRAZOLO[3,4-D]PYRIMIDIN-3-YL]PHENOL	-0.095	-0.202	0.572	-0.165	-0.05	0.332	0.298	0.584	0.367	0.39
DB08053	1-cyclobutyl-3-(3,4-dimethoxyphenyl)-1H-pyrazolo[3,4-d]pyrimidin-4-amine	-0.095	-0.202	0.572	-0.165	-0.05	0.332	0.298	0.584	0.367	0.39
DB08054	1-(1-methylethyl)-3-quinolin-6-yl-1H-pyrazolo[3,4-d]pyrimidin-4-amine	-0.095	-0.202	0.572	-0.165	-0.05	0.332	0.298	0.584	0.367	0.39
DB01126	Dutasteride	-0.094	0.111	-0.079	-0.727	-0.33	0.56	0.55	0.49	0.256	0.525
DB00180	Flunisolide	-0.093	0.118	-1.204	-1.166	0.369	0.337	0.472	0.083	0.074	0.575
DB00253	Medrysone	-0.093	0.118	-1.204	-1.166	0.369	0.337	0.472	0.083	0.074	0.575
DB00394	Beclomethasone dipropionate	-0.093	0.118	-1.204	-1.166	0.369	0.337	0.472	0.083	0.074	0.575
DB01128	Bicalutamide	-0.092	0.228	-0.564	-0.42	0.184	0.357	0.547	0.196	0.239	0.461
DB09220	Nicorandil	-0.092	0.505	0.615	0.713	-0.892	0.525	0.772	0.646	0.699	0.089
DB03226	Trifluoroethanol	-0.086	0.175	1.021	0.005	0.403	0.522	0.574	0.763	0.442	0.653
DB08052	1-cyclopentyl-3-(1H-pyrrolo[2,3-b]pyridin-5-yl)-1H-pyrazolo[3,4-d]pyrimidin-4-amine	-0.086	-0.15	0.489	-0.074	-0.21	0.372	0.349	0.548	0.387	0.311
DB08653	2-(1H-INDOL-3-YL)ETHANAMINE	-0.086	-1.122	1.551	1.684	0.378	0.423	0.104	0.897	0.948	0.556
DB00128	Aspartic acid	-0.085	-0.322	0.768	1.151	-0.223	0.423	0.317	0.669	0.826	0.337
DB09123	Dienogest	-0.084	0.072	-1.042	-0.744	0.233	0.349	0.465	0.078	0.148	0.497
DB05234	LGD2941	-0.083	0.214	-1.229	-1.219	0.092	0.341	0.491	0.081	0.068	0.441
DB08674	(20S)-19,20,21,22-TETRAHYDRO-19-OXO-5H-18,20-ETHANO-12,14-ETHENO-6,10-METHENO-18H-BENZ[D]IMIDAZO[4,3-K][1,6,9,12]OXATRIAZA-CYCLOOCTADECOSINE-9-CARBONITRILE	-0.083	1.075	1.892	0.718	-1.915	0.427	0.843	0.925	0.532	0.012
DB08867	Ulipristal	-0.083	0.214	-1.229	-1.219	0.092	0.341	0.491	0.081	0.068	0.441
DB00878	Chlorhexidine	-0.082	0.523	0.266	0.727	0.171	0.424	0.662	0.455	0.697	0.488
DB04140	1-Benzyl-3-(4-Methoxy-Benzenesulfonyl)-6-Oxo-Hexahydro-Pyrimidine-4-Carboxylic Acid Hydroxyamide	-0.081	-0.075	0.55	0.704	0.282	0.441	0.397	0.567	0.518	0.512
DB00834	Mifepristone	-0.08	0.332	-0.625	-0.783	0.029	0.365	0.57	0.2	0.154	0.436
DB03499	Malate Ion	-0.08	0.139	0.745	-0.182	-0.461	0.658	0.732	0.742	0.231	0.249
DB00892	Oxybuprocaine	-0.077	0.063	0.446	-0.245	-0.299	0.343	0.401	0.438	0.267	0.242
DB00881	Quinapril	-0.075	0.392	-0.629	0.141	-0.412	0.588	0.813	0.053	0.36	0.267
DB00421	Spironolactone	-0.074	0.709	0.957	-0.111	-0.822	0.371	0.755	0.787	0.388	0.148
DB00158	Folic acid	-0.073	0.174	0.715	0.105	-0.498	0.64	0.742	0.834	0.65	0.35
DB01727	Isocitric Acid	-0.072	0.141	0.996	-0.081	-0.454	0.626	0.689	0.787	0.256	0.24
DB00243	Ranolazine	-0.069	0.306	0.639	0.802	-0.723	0.467	0.618	0.629	0.674	0.161
DB01583	Liotrix	-0.069	0.848	-0.078	-0.704	-1	0.455	0.789	0.139	0.182	0.113
DB03176	KB-141	-0.069	0.848	-0.078	-0.704	-1	0.455	0.789	0.139	0.182	0.113
DB07425	Sobetirome	-0.069	0.848	-0.078	-0.704	-1	0.455	0.789	0.139	0.182	0.113
DB07267	2-(6-methylpyridin-2-yl)-N-pyridin-4-ylquinazolin-4-amine	-0.068	-0.385	0.695	0.97	-0.215	0.358	0.251	0.425	0.777	0.285
DB08393	2-[(5,6-DIPHENYLFURO[2,3-D]PYRIMIDIN-4-YL)AMINO]ETHANOL	-0.068	0.402	0.695	-1.118	-0.513	0.358	0.628	0.425	0.082	0.191
DB00950	Fexofenadine	-0.064	0.143	-0.292	0.626	-0.545	0.484	0.597	0.257	0.66	0.208
DB02701	Nicotinamide	-0.064	-0.466	-1.532	0.543	0.266	0.368	0.243	0.055	0.652	0.544
DB01222	Budesonide	-0.062	0.295	-0.817	-0.2	0.151	0.379	0.554	0.132	0.356	0.472

DB01121	Phenacemide	-0.061	0.212	-0.027	-0.939	-0.332	0.382	0.448	0.395	0.296	0.345
DB01579	Phendimetrazine	-0.061	-0.056	0.991	-0.005	-0.986	0.487	0.47	0.773	0.268	0.076
DB02546	Vorinostat	-0.053	0.303	-0.209	-0.374	0.192	0.416	0.62	0.359	0.371	0.535
DB09236	Lacidipine	-0.052	1.207	1.131	0.538	-1.744	0.474	0.894	0.8	0.563	0.02
DB00451	Levothyroxine	-0.047	-0.077	0.756	0.544	0.022	0.432	0.412	0.568	0.64	0.429
DB01119	Diazoxide	-0.047	-0.211	1.951	-0.234	-0.875	0.443	0.368	0.957	0.314	0.149
DB00607	Nafcillin	-0.046	0.561	-0.409	-0.684	-0.11	0.5	0.735	0.063	0.13	0.387
DB07728	2-[2-(2-FLUOROPHENYL)PYRIDIN-4-YL]-1,5,6,7-TETRAHYDRO-4H-PYRROLO[3,2-C]PYRIDIN-4-ONE	-0.046	-0.463	0.781	-0.284	0.492	0.359	0.229	0.493	0.304	0.644
DB06742	Ambroxol	-0.045	0.438	-0.882	-0.427	-0.126	0.364	0.632	0.129	0.238	0.321
DB00331	Metformin	-0.044	0.369	-0.076	-0.183	-0.115	0.368	0.604	0.323	0.338	0.336
DB01032	Probenecid	-0.044	0.355	1.214	-1.118	-0.646	0.407	0.582	0.81	0.112	0.193
DB01977	6-(N-Phenylcarbamyl)-2-Naphthalenecarboxamidine	-0.042	0.31	0.617	-0.113	-0.49	0.448	0.572	0.588	0.296	0.226
DB07718	3-(4-HYDROXY-PHENYL)PYRUVIC ACID	-0.042	0.084	0.566	0.385	-0.267	0.53	0.563	0.59	0.419	0.331
DB09017	Brotizolam	-0.042	0.088	-1.176	-0.668	0.415	0.37	0.486	0.068	0.16	0.622
DB09389	Norgestrel	-0.042	0.112	-1.033	-0.753	0.257	0.388	0.477	0.074	0.142	0.514
DB04840	Debrisoquine	-0.04	0.155	1.218	0.709	-0.295	0.606	0.67	0.835	0.597	0.345
DB02873	1-(2,6-Dichlorophenyl)-5-(2,4-Difluorophenyl)-7-Piperazin-1-Yl-3,4-Dihydroquinazolin-2(1h)-One	-0.038	-0.348	0.469	0.394	-0.246	0.379	0.284	0.335	0.555	0.271
DB08941	Isoxsuprine	-0.038	0.067	-1.06	0.237	0.077	0.429	0.506	0.075	0.48	0.453
DB00153	Ergocalciferol	-0.037	0.328	-1.273	-0.346	0.327	0.376	0.573	0.057	0.275	0.525
DB07227	4-[(5-{[4-(3-CHLOROPHENYL)-3-OXOPIPERAZIN-1-YL]METHYL}-1H-IMIDAZOL-1-YL)METHYL]BENZONITRILE	-0.036	1.264	1.627	0.573	-1.981	0.479	0.906	0.873	0.469	0.009
DB00567	Cephalexin	-0.034	0.145	1.278	0.698	-0.227	0.567	0.629	0.873	0.603	0.379
DB01076	Atorvastatin	-0.034	-0.092	0.28	-0.074	0.586	0.371	0.363	0.521	0.4	0.686
DB08799	Antazoline	-0.034	0.444	-0.715	0.003	-0.479	0.688	0.859	0.048	0.362	0.236
DB00824	Enprofylline	-0.033	0.37	-1.044	0.224	-0.287	0.451	0.608	0.03	0.316	0.296
DB01001	Salbutamol	-0.033	0.297	-1.716	0.649	-0.572	0.445	0.567	0.021	0.643	0.217
DB06994	(2S,3S)-3-{3-[2-chloro-4-(methylsulfonyl)phenyl]-1,2,4-oxadiazol-5-yl}-1-cyclopentylidene-4-cyclopropyl-1-fluorobutan-2-amine	-0.032	0.143	0.227	0.161	-0.445	0.545	0.628	0.447	0.307	0.251
DB00194	Vidarabine	-0.027	1.147	-2.496	-0.41	0.084	0.397	0.892	0.01	0.283	0.458
DB00406	Gentian violet cation	-0.027	0.531	0.54	1.406	-0.89	0.391	0.665	0.658	0.914	0.124
DB01766	Beta-(2-Naphthyl)-Alanine	-0.027	-0.208	1.33	-0.692	0.869	0.356	0.334	0.9	0.168	0.788
DB04464	N-Formylmethionine	-0.026	0.667	-0.372	0.253	-1.427	0.403	0.74	0.26	0.529	0.067
DB00631	Clofarabine	-0.025	1.074	-1.369	-0.918	-0.178	0.443	0.822	0.058	0.121	0.342
DB02352	3-(Benzyloxy)Pyridin-2-Amine	-0.024	-0.428	0.601	-0.078	0.176	0.387	0.248	0.408	0.361	0.462
DB00484	Brimonidine	-0.023	-0.229	2.934	0.281	-1.12	0.452	0.342	0.989	0.361	0.087
DB00762	Irinotecan	-0.021	-0.172	0.237	-1.084	0.322	0.397	0.362	0.5	0.099	0.577
DB01212	Ceftriaxone	-0.021	0.465	1.159	-0.049	-0.689	0.468	0.642	0.807	0.353	0.191
DB01761	4-[5-[2-(1-Phenyl-Ethylamino)-Pyrimidin-4-Yl]-1-Methyl-4-(3-Trifluoromethylphenyl)-1h-Imidazol-2-Yl]-Piperidine	-0.019	-0.723	0.654	0.858	-0.016	0.365	0.144	0.398	0.749	0.383
DB01166	Cilostazol	-0.018	0.983	1.828	0.053	-1.758	0.546	0.899	0.929	0.281	0.005
DB00953	Rizatriptan	-0.017	-0.696	1.635	1.354	0.259	0.453	0.184	0.908	0.881	0.529
DB04059	8-(Pyrimidin-2-Ylamino)Naphthalene-2-Carboximidamide	-0.017	0.418	0.29	0.274	-0.198	0.454	0.605	0.42	0.508	0.352
DB08612	1,1,1-TRIFLUORO-3-(OCTYLTHIO)ACETONE	-0.015	0.201	0.091	-0.573	-0.114	0.721	0.721	0.573	0.227	0.651

DB00136	Calcitriol	-0.012	0.624	0.77	0.719	-1.225	0.454	0.681	0.652	0.589	0.076
DB08890	Linaclotide	-0.012	0.026	0.349	-0.095	-0.257	0.386	0.396	0.488	0.397	0.349
DB00143	Glutathione	-0.01	-0.466	0.428	0.543	0.034	0.45	0.247	0.708	0.658	0.433
DB01188	Ciclopirox	-0.01	-0.141	-1.688	-0.642	0.508	0.378	0.37	0.051	0.252	0.678
DB03541	10-Propargyl-5,8-Dideazafolic Acid	-0.01	0.328	1.054	-0.034	-0.538	0.641	0.756	0.798	0.286	0.207
DB04719	DIMETHYL-(4,5,6,7-TETRABROMO-1H-BENZOIMIDAZOL-2-YL)-AMINE	-0.009	-0.034	-0.155	-1.425	-0.411	0.395	0.386	0.261	0.044	0.249
DB07149	(7S)-2-(2-aminopyrimidin-4-yl)-7-(2-fluoroethyl)-1,5,6,7-tetrahydro-4H-pyrrolo[3,2-c]pyridin-4-one	-0.009	-0.291	0.08	-1.348	0.604	0.371	0.273	0.418	0.054	0.699
DB00744	Zileuton	-0.006	-0.056	0.804	0.848	-0.507	0.486	0.429	0.701	0.706	0.228
DB01177	Idarubicin	-0.004	-0.804	-0.585	-0.685	0.294	0.417	0.129	0.203	0.187	0.545
DB00151	L-Cysteine	-0.003	0.236	-0.589	-0.82	0.417	0.462	0.535	0.143	0.144	0.588
DB00296	Ropivacaine	0.001	0.125	1.618	1.255	-0.712	0.512	0.503	0.911	0.863	0.178
DB00961	Mepivacaine	0.001	0.125	1.618	1.255	-0.712	0.512	0.503	0.911	0.863	0.178
DB01086	Benzocaine	0.001	0.125	1.618	1.255	-0.712	0.512	0.503	0.911	0.863	0.178
DB06603	Panobinostat	0.002	-0.018	0.741	-0.193	0.436	0.406	0.406	0.634	0.372	0.644
DB08696		0.003	1.035	1.083	0.162	-0.739	0.475	0.817	0.785	0.416	0.168
DB06730	Gestodene	0.004	0.151	-1.011	-0.712	0.27	0.37	0.484	0.073	0.158	0.505
DB00201	Caffeine	0.009	-0.265	-1.679	0.47	0.37	0.405	0.318	0.031	0.642	0.591
DB01509	Tenamfetamine	0.009	0.355	0.397	-0.223	-0.561	0.644	0.747	0.575	0.201	0.191
DB08800	Chloropyramine	0.01	0.521	-0.269	-0.673	0.23	0.502	0.71	0.14	0.168	0.547
DB00711	Diethylcarbamazine	0.011	0.306	-0.399	-1.223	0.869	0.38	0.56	0.268	0.073	0.792
DB00997	Doxorubicin	0.013	0.067	0.769	-1.948	0.064	0.421	0.467	0.687	0.025	0.457
DB00547	Desoximetasone	0.014	0.26	-0.919	-0.834	0.198	0.4	0.56	0.137	0.153	0.511
DB00959	Methylprednisolone	0.014	0.26	-0.919	-0.834	0.198	0.4	0.56	0.137	0.153	0.511
DB03466	BMS184394	0.014	0.865	1.579	0.406	-1.715	0.551	0.866	0.887	0.407	0.011
DB04865	Omacetaxine mepesuccinate	0.014	0.343	-0.712	-0.422	0.541	0.365	0.563	0.189	0.256	0.673
DB01087	Primaquine	0.016	-0.185	-0.73	0.03	-0.083	0.459	0.353	0.163	0.45	0.397
DB00619	Imatinib	0.017	-0.032	1.005	0.674	-0.793	0.414	0.39	0.806	0.753	0.152
DB00465	Ketorolac	0.018	0.193	1.381	0.773	-0.826	0.413	0.5	0.873	0.689	0.122
DB06989	{4-[2-BENZYL-3-METHOXY-2-(METHOXYCARBONYL)-3-OXOPROPYL]PHENYL}SULFAMIC ACID	0.019	0.555	-0.076	-0.013	-0.425	0.487	0.683	0.138	0.345	0.255
DB07207	2-(4-HYDROXY-5-PHENYL-1H-PYRAZOL-3-YL)-1H-BENZOIMIDAZOLE-5-CARBOXAMIDINE	0.019	0.319	1.092	0.26	-0.823	0.543	0.672	0.798	0.35	0.13
DB09115	Diiodohydroxyquinoline	0.022	0.404	0.065	-1.162	-0.534	0.405	0.604	0.443	0.078	0.193
DB01821	L-N(Omega)-Nitroarginine-2,4-L-Diaminobutyric Amide	0.024	0.153	1.36	0.435	-0.91	0.606	0.623	0.866	0.438	0.1
DB02027	N-{(4s)-4-Amino-5-[(2-Aminoethyl)Amino]Pentyl}-N'-Nitroguanidine	0.024	0.153	1.36	0.435	-0.91	0.606	0.623	0.866	0.438	0.1
DB02077	L-N(omega)-Nitroarginine-(4R)-Amino-L-Proline Amide	0.024	0.153	1.36	0.435	-0.91	0.606	0.623	0.866	0.438	0.1
DB02141	S,S'-(1,4-Phenylene-Bis(1,2-Ethanediyl))Bis-Isothiourea	0.024	0.153	1.36	0.435	-0.91	0.606	0.623	0.866	0.438	0.1
DB03707	S-Ethyl-N-Phenyl-Isothiourea	0.024	0.153	1.36	0.435	-0.91	0.606	0.623	0.866	0.438	0.1
DB04018	S-Isopropyl-Isothiourea	0.024	0.153	1.36	0.435	-0.91	0.606	0.623	0.866	0.438	0.1
DB07107	(1S)-2-(1H-INDOL-3-YL)-1-[({5-[(E)-2-PYRIDIN-4-YLVINYL]PYRIDIN-3-YL}OXY)METHYL]ETHYLAMINE	0.024	-0.425	0.074	-0.221	0.356	0.404	0.217	0.361	0.338	0.566
DB08018	N-{(3S,4S)-4-[(6-AMINO-4-METHYLPYRIDIN-2-YL)METHYL]PYRROLIDIN-3-YL}-N'-(4-CHLOROBENZYL)ETHANE-1,2-DIAMINE	0.024	0.153	1.36	0.435	-0.91	0.606	0.623	0.866	0.438	0.1

DB08019	N-{(3R,4S)-4-[(6-amino-4-methylpyridin-2-yl)methyl]pyrrolidin-3-yl}-N'-(3-chlorobenzyl)ethane-1,2-diamine	0.024	0.153	1.36	0.435	-0.91	0.606	0.623	0.866	0.438	0.1
DB04854	Febuxostat	0.025	0.086	-1.135	-0.506	0.625	0.384	0.454	0.062	0.203	0.722
DB04821	Nomifensine	0.026	0.907	-1.414	-0.534	-0.801	0.465	0.778	0.034	0.176	0.153
DB01082	Streptomycin	0.028	0.318	0.121	-1.211	-0.339	0.577	0.562	0.531	0.019	0.522
DB03865	6-Chloro-2-(2-Hydroxy-Biphenyl-3-Yl)-1h-Indole-5-Carboxamidine	0.028	0.172	-0.253	-0.427	-0.163	0.481	0.505	0.136	0.272	0.33
DB02175	Malonic acid	0.03	0.483	0.599	-0.559	-0.506	0.399	0.672	0.373	0.19	0.196
DB02925	Piretanide	0.031	0.411	1.37	0.09	-0.771	0.473	0.635	0.852	0.401	0.158
DB04832	Zimelidine	0.031	0.461	1.263	-0.052	-0.975	0.563	0.744	0.836	0.241	0.08
DB09216	Tolfenamic Acid	0.031	-0.062	-0.048	-0.58	0.584	0.421	0.394	0.353	0.196	0.676
DB00247	Methysergide	0.032	-1.295	1.976	1.393	1.167	0.478	0.078	0.969	0.896	0.853
DB00756	Hexachlorophene	0.032	0.829	0.122	1.066	-0.871	0.426	0.778	0.503	0.838	0.145
DB00380	Dexrazoxane	0.034	-0.137	1.236	-0.392	1.186	0.467	0.397	0.865	0.268	0.838
DB01167	Itraconazole	0.035	-0.043	-0.436	-0.125	0.058	0.409	0.407	0.211	0.376	0.417
DB07297	5,6-DIPHENYL-N-(2-PIPERAZIN-1-YLETHYL)FURO[2,3-D]PYRIMIDIN-4-AMINE	0.035	0.896	0.356	-0.12	-0.527	0.477	0.766	0.247	0.354	0.237
DB00224	Indinavir	0.038	0.526	0.142	0.269	-0.248	0.686	0.823	0.464	0.501	0.355
DB02424	Geldanamycin	0.038	-0.364	-0.983	-0.208	0.545	0.413	0.247	0.117	0.311	0.633
DB02550	8-(2-Chloro-3,4,5-Trimethoxy-Benzyl)-2-Fluoro-9-Pent-4-Ylnyl-9h-Purin-6-Ylamine	0.038	-0.364	-0.983	-0.208	0.545	0.413	0.247	0.117	0.311	0.633
DB02754	9-Butyl-8-(3,4,5-Trimethoxybenzyl)-9h-Purin-6-Amine	0.038	-0.364	-0.983	-0.208	0.545	0.413	0.247	0.117	0.311	0.633
DB02840	4-(1,3-Benzodioxol-5-Yl)-5-(5-Ethyl-2,4-Dihydroxyphenyl)-2h-Pyrazole-3-Carboxylic Acid	0.038	-0.364	-0.983	-0.208	0.545	0.413	0.247	0.117	0.311	0.633
DB03749	4-(1h-Imidazol-4-Yl)-3-(5-Ethyl-2,4-Dihydroxy-Phenyl)-1h-Pyrazole	0.038	-0.364	-0.983	-0.208	0.545	0.413	0.247	0.117	0.311	0.633
DB04588	N-[4-(AMINOSULFONYL)BENZYL]-5-(5-CHLORO-2,4-DIHYDROXYPHENYL)-1H-PYRAZOLE-4-CARBOXAMIDE	0.038	-0.364	-0.983	-0.208	0.545	0.413	0.247	0.117	0.311	0.633
DB06956	N-(4-ACETYLPHENYL)-5-(5-CHLORO-2,4-DIHYDROXYPHENYL)-1H-PYRAZOLE-4-CARBOXAMIDE	0.038	-0.364	-0.983	-0.208	0.545	0.413	0.247	0.117	0.311	0.633
DB06957	4-CHLORO-6-(4-{4-[4-(METHYLSULFONYL)BENZYL]PIPERAZIN-1-YL}-1H-PYRAZOL-5-YL)BENZENE-1,3-DIOL	0.038	-0.364	-0.983	-0.208	0.545	0.413	0.247	0.117	0.311	0.633
DB06961	5-(5-chloro-2,4-dihydroxyphenyl)-N-ethyl-4-[4-(morpholin-4-ylmethyl)phenyl]isoxazole-3-carboxamide	0.038	-0.364	-0.983	-0.208	0.545	0.413	0.247	0.117	0.311	0.633
DB06964	5-(5-CHLORO-2,4-DIHYDROXYPHENYL)-N-ETHYL-4-(4-METHOXYPHENYL)ISOXAZOLE-3-CARBOXAMIDE	0.038	-0.364	-0.983	-0.208	0.545	0.413	0.247	0.117	0.311	0.633
DB06969	2-amino-4-[2,4-dichloro-5-(2-pyrrolidin-1-ylethoxy)phenyl]-N-ethylthieno[2,3-d]pyrimidine-6-carboxamide	0.038	-0.364	-0.983	-0.208	0.545	0.413	0.247	0.117	0.311	0.633
DB07495	5-(5-CHLORO-2,4-DIHYDROXYPHENYL)-N-ETHYL-4-(4-METHOXYPHENYL)-1H-PYRAZOLE-3-CARBOXAMIDE	0.038	-0.364	-0.983	-0.208	0.545	0.413	0.247	0.117	0.311	0.633

DB07594	4-[4-(2,3-DIHYDRO-1,4-BENZODIOXIN-6-YL)-3-METHYL-1H-PYRAZOL-5-YL]-6-ETHYLBENZENE-1,3-DIOL	0.038	-0.364	-0.983	-0.208	0.545	0.413	0.247	0.117	0.311	0.633
DB07601	4-chloro-6-{5-[(2-morpholin-4-ylethyl)amino]-1,2-benzisoxazol-3-yl}benzene-1,3-diol	0.038	-0.364	-0.983	-0.208	0.545	0.413	0.247	0.117	0.311	0.633
DB07877	8-(6-BROMO-BENZO[1,3]DIOXOL-5-YLSULFANYL)-9-(3-ISOPROPYLAMINO-PROPYL)-ADENINE	0.038	-0.364	-0.983	-0.208	0.545	0.413	0.247	0.117	0.311	0.633
DB08787	4-(2,4-dichlorophenyl)-5-phenyldiazenyl-pyrimidin-2-amine	0.038	-0.364	-0.983	-0.208	0.545	0.413	0.247	0.117	0.311	0.633
DB08788	3,6-DIAMINO-5-CYANO-4-(4-ETHOXYPHENYL)THIENO[2,3-B]PYRIDINE-2-CARBOXAMIDE	0.038	-0.364	-0.983	-0.208	0.545	0.413	0.247	0.117	0.311	0.633
DB08789	2-AMINO-4-(2,4-DICHLOROPHENYL)-N-ETHYLTHIENO[2,3-D]PYRIMIDINE-6-CARBOXAMIDE	0.038	-0.364	-0.983	-0.208	0.545	0.413	0.247	0.117	0.311	0.633
DB00384	Triamterene	0.039	0.554	0.099	-1.087	-0.244	0.427	0.687	0.439	0.095	0.313
DB00131	Adenosine phosphate	0.043	0.511	-0.767	-0.338	-0.318	0.436	0.66	0.19	0.298	0.29
DB01599	Probucol	0.043	0.236	1.263	0.875	-0.562	0.526	0.559	0.824	0.71	0.229
DB05109	Trabectedin	0.043	0.384	0.411	0.237	-0.41	0.471	0.686	0.328	0.48	0.224
DB00440	Trimethoprim	0.044	0.912	0.056	0.274	-0.576	0.47	0.784	0.322	0.521	0.221
DB00698	Nitrofurantoin	0.044	0.17	-1.068	-0.437	0.658	0.397	0.498	0.078	0.229	0.721
DB07676	3-({[(3S)-3,4-dihydroxybutyl]oxy}amino)-1H,2'H-2,3'-biindol-2'-one	0.044	-0.356	1.323	0.92	0.094	0.397	0.255	0.905	0.749	0.433
DB00509	Dextrothyroxine	0.052	1.149	0.342	-0.313	-0.98	0.481	0.865	0.411	0.256	0.122
DB02998	Metribolone	0.052	0.059	-0.747	-0.323	0.614	0.408	0.448	0.098	0.286	0.706
DB09223	Blonanserin	0.053	0.421	2.557	0.214	-1.478	0.555	0.69	0.974	0.33	0.025
DB07050	5-[(phenylsulfonyl)amino]-1,3,4-thiadiazole-2-sulfonamide	0.056	0.702	1.297	-1.676	-1.055	0.496	0.721	0.84	0.043	0.111
DB04083	N'-Pyridoxyl-Lysine-5'-Monophosphate	0.059	0.514	0.84	0.418	-1.306	0.486	0.652	0.702	0.426	0.073
DB01063	Acetophenazine	0.06	0.322	-1.067	-0.427	0.682	0.406	0.579	0.063	0.213	0.713
DB00466	Picrotoxin	0.061	0.308	0.739	0.591	-0.481	0.516	0.6	0.684	0.585	0.233
DB00678	Losartan	0.064	0.804	0.742	0.538	-0.658	0.505	0.758	0.67	0.461	0.178
DB02473	6-[N-(1-Isopropyl-3,4-Dihydro-7-Isoquinolinyl)Carbamyl]-2-Naphthalenecarboxamidine	0.064	0.217	0.715	-0.37	-0.164	0.488	0.521	0.632	0.288	0.336
DB07076	6-[(Z)-AMINO(IMINO)METHYL]-N-[3-(CYCLOPENTYLOXY)PHENYL]-2-NAPHTHAMIDE	0.064	0.217	0.715	-0.37	-0.164	0.488	0.521	0.632	0.288	0.336
DB00803	Colistin	0.067	0.781	1.644	0.458	-0.291	0.412	0.796	0.932	0.621	0.269
DB00504	Levallorphan	0.068	0.559	-0.525	0.369	-0.376	0.759	0.853	0.066	0.482	0.331
DB00913	Anileridine	0.068	0.559	-0.525	0.369	-0.376	0.759	0.853	0.066	0.482	0.331
DB01466	Ethylmorphine	0.068	0.559	-0.525	0.369	-0.376	0.759	0.853	0.066	0.482	0.331
DB01501	Difenoxin	0.068	0.559	-0.525	0.369	-0.376	0.759	0.853	0.066	0.482	0.331
DB01551	Dihydrocodeine	0.068	0.559	-0.525	0.369	-0.376	0.759	0.853	0.066	0.482	0.331
DB09049	Naloxegol	0.068	0.559	-0.525	0.369	-0.376	0.759	0.853	0.066	0.482	0.331
DB00328	Indometacin	0.069	0.039	-0.367	0.454	0.758	0.459	0.441	0.273	0.617	0.757
DB00768	Olopatadine	0.069	0.289	-1.038	0.989	0.143	0.476	0.553	0.048	0.76	0.46
DB01098	Rosuvastatin	0.069	0.231	1.15	0.32	-0.673	0.556	0.609	0.809	0.377	0.176
DB04895	Pegaptanib	0.069	0.317	0.599	0.533	-0.59	0.577	0.661	0.604	0.459	0.21
DB06843	2',5'-DIDEOXY-ADENOSINE 3'-MONOPHOSPHATE	0.071	0.378	0.687	0.293	-0.615	0.673	0.739	0.663	0.425	0.185
DB13149	Protein S human	0.071	0.39	1.866	0.028	-0.615	0.65	0.739	0.928	0.262	0.183
DB00113	Technetium Tc-99m arcitumomab	0.073	0.353	0.395	-1.05	0.024	0.621	0.658	0.645	0.037	0.629
DB00159	Icosapent	0.073	-0.264	1.951	1.351	-0.75	0.475	0.358	0.967	0.903	0.184

DB08356	4-[4-(4-methoxyphenyl)-5-methyl-1H-pyrazol-3-yl]benzene-1,3-diol	0.073	-0.287	-1.007	-0.156	0.62	0.428	0.295	0.108	0.326	0.7
DB02491	4-[4-(1-Amino-1-Methylethyl)Phenyl]-5-Chloro-N-[4-(2-Morpholin-4-Ylethyl)Phenyl]Pyrimidin-2-Amine	0.074	-0.156	-0.77	0.535	0.398	0.42	0.347	0.11	0.631	0.584
DB05186	SQ-109	0.075	0.832	0.305	0.077	-0.816	0.646	0.891	0.483	0.299	0.112
DB07274	N-cyclopropyl-6-[(6,7-dimethoxyquinolin-4-yl)oxy]naphthalene-1-carboxamide	0.078	-0.162	0.345	-0.618	0.534	0.417	0.343	0.464	0.215	0.667
DB09020	Bisacodyl	0.081	0.423	-0.616	-0.247	0.36	0.438	0.639	0.196	0.293	0.537
DB00378	Dydrogesterone	0.082	0.314	-0.871	-0.514	0.38	0.41	0.563	0.087	0.209	0.586
DB01285	Corticotropin	0.083	0.833	0.918	-0.666	-0.023	0.599	0.862	0.773	0.175	0.473
DB04147	Lauryl Dimethylamine-N-Oxide	0.083	0.184	-0.69	-1.315	-0.135	0.489	0.527	0.163	0.068	0.386
DB00860	Prednisolone	0.084	0.245	-0.729	-0.667	0.071	0.458	0.529	0.14	0.191	0.45
DB00455	Loratadine	0.085	1.035	-0.307	1.199	-1.264	0.48	0.81	0.227	0.819	0.07
DB00400	Griseofulvin	0.086	0.188	-1.287	-0.614	0.577	0.417	0.523	0.051	0.172	0.673
DB00857	Terbinafine	0.086	0.582	1.018	-0.073	-0.947	0.585	0.762	0.785	0.244	0.087
DB07045	(2R,3R,4S,5R)-2-[6-amino-8-[(3,4-dichlorophenyl)methylamino]purin-9-yl]-5-(hydroxymethyl)oxolane-3,4-diol	0.086	-0.269	0.984	0.498	-0.354	0.419	0.311	0.594	0.613	0.235
DB04297	7-[4-(Dimethylamino)Phenyl]-N-Hydroxy-4,6-Dimethyl-7-Oxo-2,4-Heptadienamide	0.087	0.056	-0.126	0.123	0.777	0.442	0.429	0.362	0.492	0.749
DB00665	Nilutamide	0.089	0.216	-1.016	-0.858	0.732	0.44	0.511	0.073	0.133	0.758
DB08553	(1E)-5-(1-piperidin-4-yl-3-pyridin-4-yl-1H-pyrazol-4-yl)-2,3-dihydro-1H-inden-1-one oxime	0.089	0.03	0.559	0.956	-0.387	0.44	0.41	0.361	0.751	0.213
DB07832	4-{4-[(5-hydroxy-2-methylphenyl)amino]quinolin-7-yl}-1,3-thiazole-2-carbaldehyde	0.09	-0.15	0.801	0.337	-0.516	0.42	0.363	0.485	0.553	0.181
DB00590	Doxazosin	0.092	-0.127	0.352	0.9	-0.814	0.506	0.403	0.532	0.749	0.15
DB00739	Hetacillin	0.092	0.357	1.533	0.407	-0.943	0.58	0.701	0.889	0.426	0.081
DB00990	Exemestane	0.092	0.194	-1.03	-0.721	0.831	0.455	0.519	0.063	0.149	0.778
DB02193		0.095	-0.013	0.445	0.409	0.252	0.584	0.488	0.559	0.382	0.551
DB03136	4-Iodobenzo[B]Thiophene-2-Carboxamidine	0.095	-0.013	0.445	0.409	0.252	0.584	0.488	0.559	0.382	0.551
DB01614	Acepromazine	0.097	-0.078	1.685	0.954	-1.685	0.482	0.418	0.91	0.658	0.042
DB00413	Pramipexole	0.101	-0.402	2.644	2.179	0.038	0.489	0.288	0.992	0.99	0.421
DB03649	[{(5-Chloro-2-Pyridinyl)Amino} Methylene]-1,1-Bisphosphonate	0.101	0.517	1.299	0.188	-0.808	0.635	0.815	0.844	0.336	0.112
DB07215	2-METHYL-2-(4-{[({4-METHYL-2-[4-(TRIFLUOROMETHYL)PHENYL]-1,3-THIAZOL-5-YL}CARBONYL)AMINO]METHYL}PHENOXY)PROPANOIC ACID	0.101	-0.155	1.357	1.747	-1.091	0.494	0.39	0.91	0.901	0.106
DB07724	Indeglitazar	0.101	-0.155	1.357	1.747	-1.091	0.494	0.39	0.91	0.901	0.106
DB01621	Pipotiazine	0.105	0.301	1.663	0.324	-1.021	0.539	0.58	0.9	0.352	0.099
DB06404	Human C1-esterase inhibitor	0.105	0.281	0.899	-0.172	-0.147	0.506	0.551	0.728	0.257	0.335
DB09228	Conestat alfa	0.105	0.281	0.899	-0.172	-0.147	0.506	0.551	0.728	0.257	0.335
DB00894	Testolactone	0.107	0.289	0.817	-0.948	-0.222	0.532	0.53	0.668	0.159	0.478
DB01217	Anastrozole	0.107	0.289	0.817	-0.948	-0.222	0.532	0.53	0.668	0.159	0.478
DB05773	Trastuzumab emtansine	0.109	0.414	1.004	0.143	-0.351	0.668	0.747	0.786	0.459	0.31
DB06366	Pertuzumab	0.109	0.414	1.004	0.143	-0.351	0.668	0.747	0.786	0.459	0.31
DB07073	5,5-dimethyl-2-morpholin-4-yl-5,6-dihydro-1,3-benzothiazol-7(4H)-one	0.109	0.278	0.731	0.709	-0.589	0.585	0.612	0.646	0.534	0.205

DB07503	(5E)-5-[(2,2-DIFLUORO-1,3-BENZODIOXOL-5-YL)METHYLENE]-1,3-THIAZOLIDINE-2,4-DIONE	0.109	0.278	0.731	0.709	-0.589	0.585	0.612	0.646	0.534	0.205
DB03383	5-Chloro-1h-Indole-2-Carboxylic Acid [1-(4-Fluorobenzyl)-2-(4-Hydroxypiperidin-1yl)-2-Oxoethyl]Amide	0.112	0.715	0.588	0.55	-1.3	0.532	0.734	0.583	0.457	0.059
DB03597	Gamma-Glutamyl[S-(2-Iodobenzyl)Cysteinyl]Glycine	0.112	0.672	-0.049	0.028	-0.858	0.593	0.832	0.347	0.272	0.089
DB04530	S,S-(2-Hydroxyethyl)Thiocysteine	0.113	-0.167	-0.368	0.206	0.352	0.464	0.347	0.258	0.488	0.564
DB07812	N-[(1S)-2-amino-1-phenylethyl]-5-(1H-pyrrolo[2,3-b]pyridin-4-yl)thiophene-2-carboxamide	0.114	-0.065	1.333	0.158	0.273	0.449	0.389	0.915	0.452	0.509
DB01880	3,4-Dihydroxycinnamic Acid	0.116	-0.032	0.681	-0.798	-0.063	0.51	0.424	0.642	0.157	0.393
DB01074	Perhexiline	0.117	0.332	0.694	0.858	-0.449	0.484	0.584	0.671	0.753	0.241
DB06623	Flupirtine	0.118	-0.741	1.482	1.826	0.378	0.448	0.153	0.899	0.965	0.596
DB00191	Phentermine	0.119	0.767	0.178	-0.11	-0.993	0.631	0.834	0.467	0.225	0.07
DB06973	4,4'-PROPANE-2,2-DIYLDIPHENOL	0.125	0.408	-1.508	0.756	-0.031	0.446	0.617	0.043	0.708	0.379
DB00949	Felbamate	0.127	0.547	0.106	0.235	-0.423	0.634	0.768	0.415	0.349	0.257
DB07290	(2R)-2-{[(4-FLUORO-3-METHYLPHENYL)SULFONYL]AMINO}-N-HYDROXY-2-TETRAHYDRO-2H-PYRAN-4-YLACETAMIDE	0.131	-0.5	-1.52	1.675	1.478	0.515	0.267	0.027	0.901	0.905
DB09010	Carmofur	0.131	0.153	0.128	0.626	-0.196	0.508	0.544	0.314	0.653	0.325
DB02153	3-sulfino-L-alanine	0.132	0.449	1.02	-0.384	-0.113	0.516	0.617	0.762	0.285	0.372
DB09274	Artesunate	0.132	0.309	-0.983	0.567	0.267	0.46	0.56	0.117	0.649	0.51
DB00598	Labetalol	0.134	0.483	-1.307	0.535	-1.628	0.528	0.626	0.051	0.595	0.046
DB09085	Tetracaine	0.135	-0.904	1.852	1.799	-0.182	0.523	0.154	0.937	0.975	0.343
DB02659	Cholic Acid	0.136	1.118	1.361	0.452	-1.09	0.504	0.849	0.866	0.587	0.112
DB08483		0.138	-0.066	1.275	1.622	-0.716	0.514	0.407	0.875	0.87	0.196
DB07307	N-cyclopropyl-4-methyl-3-[1-(2-methylphenyl)phthalazin-6-yl]benzamide	0.141	-0.355	-0.597	1.333	0.025	0.463	0.266	0.138	0.877	0.418
DB09073	Palbociclib	0.142	-0.123	0.885	-1.304	-0.01	0.507	0.392	0.735	0.063	0.436
DB00110	Palivizumab	0.143	0.086	0.078	-0.411	1.068	0.507	0.485	0.365	0.25	0.822
DB01171	Moclobemide	0.143	0.214	-1.497	-0.449	0.718	0.464	0.506	0.029	0.237	0.749
DB00056	Gemtuzumab ozogamicin	0.144	-0.053	-0.101	-0.165	1.246	0.51	0.402	0.288	0.338	0.86
DB00412	Rosiglitazone	0.144	0.158	0.559	0.409	0.062	0.517	0.521	0.586	0.606	0.439
DB06207	Silodosin	0.145	0.184	0.913	0.419	-0.916	0.549	0.539	0.719	0.392	0.123
DB03084	Cyclopropyl-{4-[5-(3,4-Dichlorophenyl)-2-[(1-Methyl)-Piperidin]-4-Yl-3-Propyl-3h-Imidazol-4-Yl]-Pyrimidin-2-Yl}Amine	0.148	-0.133	0.55	0.54	-0.064	0.468	0.368	0.355	0.605	0.346
DB08010	(3Z)-1-[(6-fluoro-4H-1,3-benzodioxin-8-yl)methyl]-4-[(E)-2-phenylethenyl]-1H-indole-2,3-dione 3-oxime	0.148	-0.133	0.55	0.54	-0.064	0.468	0.368	0.355	0.605	0.346
DB08015	(3Z)-1-[(6-fluoro-4H-1,3-benzodioxin-8-yl)methyl]-4-phenyl-1H-indole-2,3-dione 3-oxime	0.148	-0.133	0.55	0.54	-0.064	0.468	0.368	0.355	0.605	0.346
DB00470	Dronabinol	0.149	0.108	0.347	0.662	-0.329	0.548	0.519	0.536	0.667	0.295
DB07253	N'-(5-chloro-1,3-benzodioxol-4-yl)-N-(3-methylsulfonylphenyl)pyrimidine-2,4-diamine	0.149	0.214	0.453	0.579	0.018	0.468	0.506	0.367	0.637	0.373
DB01200	Bromocriptine	0.151	-0.305	1.619	1.224	-0.267	0.509	0.319	0.934	0.883	0.317
DB07702	17alpha-Estriol	0.156	0.359	-0.19	0.861	-1.432	0.465	0.601	0.345	0.735	0.047

DB02983	Para-Mercury-Benzenesulfonic Acid	0.158	0.536	-0.863	1.346	-1.149	0.532	0.812	0.16	0.867	0.074
DB06940	N-ethyl-4-{[5-(methoxycarbamoyl)-2-methylphenyl]amino}-5-methylpyrrolo[2,1-f][1,2,4]triazine-6-carboxamide	0.158	0.021	0.51	0.47	-0.134	0.49	0.417	0.338	0.591	0.312
DB06991	N-[2-methyl-5-(methylcarbamoyl)phenyl]-2-{[(1R)-1-methylpropyl]amino}-1,3-thiazole-5-carboxamide	0.158	0.021	0.51	0.47	-0.134	0.49	0.417	0.338	0.591	0.312
DB07834	N-(cyclopropylmethyl)-2'-methyl-5'-(5-methyl-1,3,4-oxadiazol-2-yl)biphenyl-4-carboxamide	0.158	0.021	0.51	0.47	-0.134	0.49	0.417	0.338	0.591	0.312
DB07941	PH-797804	0.158	0.021	0.51	0.47	-0.134	0.49	0.417	0.338	0.591	0.312
DB07943	2-{4-[5-(4-chlorophenyl)-4-pyrimidin-4-yl-1H-pyrazol-3-yl]piperidin-1-yl}-2-oxoethanol	0.158	0.021	0.51	0.47	-0.134	0.49	0.417	0.338	0.591	0.312
DB08044	ABT-341	0.158	0.404	0.312	0.062	-0.251	0.709	0.773	0.505	0.298	0.332
DB00073	Rituximab	0.159	-0.072	-0.001	-0.174	1.23	0.533	0.391	0.321	0.341	0.859
DB00078	Ibritumomab tiuxetan	0.159	-0.072	-0.001	-0.174	1.23	0.533	0.391	0.321	0.341	0.859
DB00635	Prednisone	0.16	0.218	-1.008	-0.778	0.539	0.477	0.535	0.125	0.163	0.658
DB05015	Belinostat	0.16	0.379	0.794	-0.197	0.747	0.478	0.605	0.653	0.366	0.754
DB01115	Nifedipine	0.164	-1.197	0.565	1.92	0.093	0.514	0.1	0.745	0.985	0.48
DB07453	2-PHENYL-4H-BENZO[H]CHROMEN-4-ONE	0.164	-0.034	-1.394	-0.223	0.702	0.478	0.378	0.048	0.307	0.695
DB00518	Albendazole	0.165	0.884	0.001	0.336	-0.824	0.477	0.808	0.409	0.549	0.122
DB06789	Hydroxyprogesterone caproate	0.165	-0.054	0.429	0.001	-0.637	0.483	0.379	0.459	0.406	0.179
DB01229	Paclitaxel	0.166	0.006	0.548	0.299	-0.032	0.509	0.434	0.619	0.57	0.398
DB06168	Canakinumab	0.166	0.492	-0.039	-0.745	0.11	0.636	0.697	0.523	0.085	0.654
DB03082	6-[(Z)-Amino(Imino)Methyl]-N-[4-(Aminomethyl)Phenyl]-4-(Pyrimidin-2-Ylamino)-2-Naphthamide	0.167	0.384	0.412	-0.018	0.153	0.525	0.603	0.452	0.335	0.471
DB00087	Alemtuzumab	0.169	0.174	-0.249	-0.386	1.415	0.51	0.496	0.242	0.258	0.913
DB00482	Celecoxib	0.17	0.263	1.374	-0.519	-0.096	0.504	0.541	0.901	0.242	0.349
DB00922	Levosimendan	0.171	1.059	1.782	0.956	-1.645	0.617	0.874	0.919	0.76	0.028
DB00759	Tetracycline	0.172	0.401	1.019	-0.297	-0.568	0.629	0.711	0.912	0.274	0.214
DB01130	Prednicarbate	0.172	0.4	-0.965	-1.063	0.37	0.477	0.595	0.121	0.103	0.601
DB01250	Olsalazine	0.174	0.562	0.31	1.141	-1.097	0.52	0.662	0.436	0.746	0.083
DB02482	Phosphonothreonine	0.174	0.346	1.176	-0.558	0.468	0.485	0.577	0.746	0.208	0.646
DB01054	Nitrendipine	0.176	0.006	2.542	1.441	-0.176	0.531	0.409	0.981	0.916	0.343
DB00534	Chlormerodrin	0.179	0.428	1.251	-0.518	-0.274	0.704	0.692	0.84	0.256	0.592
DB07690		0.179	0.554	0.467	-0.129	-0.641	0.7	0.836	0.616	0.269	0.159
DB00682	Warfarin	0.18	1.262	-0.925	0.022	-1.424	0.525	0.867	0.121	0.397	0.061
DB00773	Etoposide	0.18	0.108	-1.73	-0.307	0.83	0.509	0.497	0.031	0.331	0.777
DB00235	Milrinone	0.182	0.925	1.22	-1.115	-1.106	0.609	0.857	0.83	0.065	0.071
DB00499	Flutamide	0.186	-0.005	-0.761	0.23	0.919	0.51	0.39	0.142	0.516	0.808
DB00640	Adenosine	0.186	0.48	-1.923	-0.45	-0.59	0.536	0.636	0.012	0.242	0.215
DB03088	Pidolic Acid	0.187	0.061	2.193	0.161	0.168	0.542	0.466	0.977	0.451	0.463
DB06283	Ziconotide	0.188	0.175	1.221	0.427	0.039	0.747	0.653	0.846	0.454	0.525
DB06144	Sertindole	0.189	-0.749	1.598	1.97	0.032	0.551	0.182	0.927	0.989	0.453
DB01094	Hesperetin	0.19	-0.74	0.606	1.338	0.191	0.5	0.159	0.615	0.89	0.491
DB00120	L-Phenylalanine	0.192	0.226	-0.139	-0.911	-0.534	0.537	0.519	0.276	0.127	0.229
DB00566	Succimer	0.192	-0.431	0.636	1.586	0.591	0.508	0.252	0.603	0.93	0.685
DB07556	N-HYDROXY-2(R)-[[(4-METHOXYPHENYL)SULFONYL](3-PICOLYL)AMINO]-3-METHYLBUTANAMIDE HYDROCHLORIDE	0.194	-0.105	-0.817	0.234	1.08	0.534	0.408	0.118	0.454	0.826
DB00155	L-Citrulline	0.197	0.661	0.484	-0.03	-1.899	0.525	0.708	0.554	0.377	0.023

DB09014	Captodiame	0.198	0.288	2.498	0.638	-0.283	0.603	0.569	0.968	0.488	0.322
DB00250	Dapsone	0.199	0.51	1.387	0.369	-0.637	0.63	0.674	0.854	0.391	0.163
DB01864	5'-Guanosine-Diphosphate-Monothiophosphate	0.199	0.749	1.95	-0.716	-1.097	0.588	0.763	0.95	0.16	0.092
DB00722	Lisinopril	0.201	0.396	1.966	1.297	-0.142	0.668	0.689	0.935	0.872	0.397
DB03368	5-Methyl-5-(4-Phenoxy-Phenyl)-Pyrimidine-2,4,6-Trione	0.201	0.252	-0.508	0.926	0.696	0.538	0.533	0.083	0.63	0.672
DB02596	Alpha,Beta-Methyleneadenosine-5'-Triphosphate	0.203	0.319	1.983	0.032	0	0.59	0.643	0.948	0.328	0.427
DB06151	Acetylcysteine	0.204	-0.747	-0.984	0.482	0.983	0.536	0.183	0.09	0.605	0.808
DB00092	Alefacept	0.205	0.043	0.131	-0.235	1.205	0.535	0.453	0.391	0.323	0.847
DB04739	4-[(4-METHYL-1-PIPERAZINYL)METHYL]-N-[3-[[4-(3-PYRIDINYL)-2-PYRIMIDINYL]AMINO]PHENYL]-BENZAMIDE	0.205	0.615	0.57	-0.272	0	0.518	0.703	0.35	0.285	0.368
DB08325	2-(2-HYDROXYETHYLAMINO)-6-(3-CHLOROANILINO)-9-ISOPROPYLPURINE	0.207	0.028	-0.359	0.706	0.456	0.511	0.409	0.21	0.682	0.618
DB09143	Sonidegib	0.207	0.539	-0.311	-1.184	0.28	0.571	0.619	0.428	0.022	0.576
DB00308	Ibutilide	0.211	0.111	0.643	0.83	-0.242	0.56	0.474	0.634	0.68	0.302
DB03921	4-(3-Pyridin-2-Yl-1h-Pyrazol-4-Yl)Quinoline	0.211	-0.835	0.922	1.789	0.473	0.533	0.152	0.558	0.955	0.618
DB06732	beta-Naphthoflavone	0.211	0.288	0.847	0.288	-0.353	0.677	0.677	0.738	0.389	0.285
DB08351	N-cyclopropyl-4-methyl-3-{2-[(2-morpholin-4-ylethyl)amino]quinazolin-6-yl}benzamide	0.214	-0.376	-0.314	0.612	0.545	0.51	0.246	0.214	0.667	0.652
DB08816	Ticagrelor	0.217	0.51	2.049	0.198	-0.479	0.747	0.823	0.941	0.39	0.228
DB00344	Protriptyline	0.219	1.013	-1.329	-0.123	-0.491	0.54	0.835	0.079	0.365	0.234
DB02519	Indirubin-5-Sulphonate	0.219	0.292	-1.063	0.491	0.498	0.514	0.546	0.071	0.599	0.616
DB00227	Lovastatin	0.222	0.117	-0.539	0.374	0.765	0.527	0.478	0.217	0.584	0.774
DB00618	Demeclocycline	0.222	0.68	-0.072	1.25	-0.162	0.529	0.733	0.391	0.865	0.33
DB08162	Fasudil	0.222	-1.021	0.043	-0.71	-0.126	0.52	0.091	0.42	0.183	0.384
DB00608	Chloroquine	0.223	-0.079	-1.107	0.945	-0.346	0.543	0.431	0.104	0.792	0.29
DB02309	5--Monophosphate-9-Beta-D-Ribofuranosyl Xanthine	0.223	0.317	1.64	0.533	-0.59	0.664	0.661	0.896	0.459	0.21
DB00261	Anagrelide	0.224	0.871	1.688	0.329	-1.021	0.69	0.856	0.918	0.401	0.071
DB00994	Neomycin	0.225	0.847	1.408	0.94	-1.251	0.655	0.841	0.859	0.765	0.047
DB08413	METHYL-PHOSPHONIC ACID MONO-(4-NITRO-PHENYL) ESTER	0.228	0.138	1.554	0.29	-0.05	0.652	0.573	0.879	0.372	0.436
DB03766	Propanoic acid	0.229	0.102	1.194	0.463	-0.143	0.608	0.485	0.812	0.419	0.35
DB05260	Gallium nitrate	0.23	1.448	-1.414	-1.446	-0.707	0.586	0.913	0.053	0.053	0.178
DB00148	Creatine	0.231	-0.652	1.586	-0.173	-0.171	0.551	0.21	0.901	0.27	0.346
DB00605	Sulindac	0.232	0.03	1.788	0.205	0.223	0.521	0.422	0.958	0.509	0.509
DB00445	Epirubicin	0.236	0.759	-0.879	-2.117	-0.408	0.51	0.762	0.15	0.022	0.268
DB00268	Ropinirole	0.238	-0.637	2.522	1.963	0.248	0.56	0.218	0.988	0.98	0.507
DB07232	Veliparib	0.238	0.975	-1.501	-1.433	1.32	0.563	0.819	0.025	0.041	0.866
DB07330	trans-4-(7-carbamoyl-1H-benzimidazol-2-yl)-1-propylpiperidinium	0.238	0.975	-1.501	-1.433	1.32	0.563	0.819	0.025	0.041	0.866
DB00379	Mexiletine	0.239	1.105	1.681	0.847	-0.06	0.54	0.884	0.938	0.765	0.397
DB02379	Beta-D-Glucose	0.241	0.103	3.661	-1.241	-0.241	0.55	0.472	1	0.074	0.321
DB05332	Romiplostim	0.241	0.391	0.419	-0.381	-0.14	0.544	0.547	0.587	0.47	0.485
DB01014	Balsalazide	0.243	0.675	1.711	0.331	-1.25	0.547	0.715	0.926	0.473	0.074
DB09078	Lenvatinib	0.246	0.56	0.01	0.766	-0.48	0.547	0.703	0.322	0.721	0.265
DB00893	Iron Dextran	0.247	0.883	0.183	0.508	-0.398	0.55	0.765	0.194	0.527	0.267
DB00316	Acetaminophen	0.248	0.212	1.722	-2.137	-1.712	0.562	0.504	0.924	0.021	0.032

DB01809	1-Ter-Butyl-3-P-Tolyl-1h-Pyrazolo[3,4-D]Pyrimidin-4-Ylamine	0.248	0.666	1.494	-0.063	-1.142	0.55	0.743	0.848	0.408	0.078
DB04820	Nialamide	0.249	0.394	-0.188	0.653	0.131	0.662	0.709	0.539	0.622	0.486
DB00645	Dyclonine	0.25	0.465	-0.363	0.155	0.539	0.54	0.625	0.268	0.476	0.656
DB01327	Cefazolin	0.251	0.318	0.608	1.244	0.142	0.581	0.597	0.611	0.822	0.488
DB08029	N~2~-(biphenyl-4-ylsulfonyl)-N-hydroxy-N~2~-(2-hydroxyethyl)glycinamide	0.253	0.064	0.033	0.605	0.213	0.558	0.454	0.344	0.499	0.482
DB07929	N-(TERT-BUTYL)-3,5-DIMETHYL-N'-[(5-METHYL-2,3-DIHYDRO-1,4-BENZODIOXIN-6-YL)CARBONYL]BENZOHYDRAZIDE	0.254	0.652	1.624	0.595	-0.974	0.666	0.808	0.92	0.512	0.118
DB11616	Pirarubicin	0.266	0.552	-0.844	0.718	0.473	0.539	0.669	0.145	0.702	0.63
DB00248	Cabergoline	0.267	-0.185	1.764	1.831	-0.595	0.566	0.372	0.937	0.968	0.22
DB00688	Mycophenolate mofetil	0.272	0.641	-2.216	-0.765	1.113	0.523	0.72	0.015	0.155	0.874
DB04839	Cyproterone acetate	0.272	0.402	-1.085	-1.001	0.602	0.566	0.628	0.097	0.093	0.683
DB07266	8-ethyl-3,10,10-trimethyl-4,5,6,8,10,12-hexahydropyrazolo[4',3':6,7]cyclohepta[1,2-b]pyrrolo[2,3-f]indol-9(1H)-one	0.272	0.36	0.435	-0.535	0.19	0.547	0.593	0.502	0.235	0.488
DB00284	Acarbose	0.273	0.694	1.958	-0.272	-1.01	0.716	0.81	0.943	0.24	0.073
DB05018	Migalastat	0.274	1.394	1.187	0.333	-2.62	0.559	0.9	0.812	0.476	0.004
DB00528	Lercanidipine	0.275	0.23	0.792	0.667	0.294	0.522	0.468	0.782	0.73	0.517
DB08462	N-(4-PHENYLAMINO-QUINAZOLIN-6-YL)-ACRYLAMIDE	0.275	0.929	1.211	0.343	-0.68	0.547	0.823	0.708	0.555	0.125
DB00309	Vindesine	0.276	0.492	-1.029	-0.54	0.953	0.564	0.628	0.064	0.199	0.818
DB00865	Benzphetamine	0.276	0.81	1.499	-0.153	-0.699	0.749	0.881	0.885	0.22	0.157
DB00028	Immune Globulin Human	0.278	0.387	-0.508	-0.942	0.155	0.568	0.602	0.12	0.138	0.439
DB00580	Valdecoxib	0.278	0.931	1.545	-0.746	-0.763	0.559	0.777	0.905	0.15	0.176
DB01099	Flucytosine	0.278	0.253	-0.812	-0.313	0.654	0.549	0.537	0.1	0.3	0.717
DB07995	N-[2-(4-BROMOCINNAMYLAMINO)ETHYL]-5-ISOQUINOLINE SULFONAMIDE	0.279	0.433	-0.936	-1.255	0.316	0.558	0.627	0.137	0.082	0.549
DB00377	Palonosetron	0.282	0.853	0.564	0.654	-0.387	0.781	0.871	0.596	0.521	0.287
DB00757	Dolasetron	0.282	0.853	0.564	0.654	-0.387	0.781	0.871	0.596	0.521	0.287
DB00417	Phenoxymethylpenicillin	0.283	0.828	0.561	0.486	-0.141	0.737	0.856	0.576	0.464	0.397
DB07662	N-[4-(3-BROMO-PHENYLAMINO)-QUINAZOLIN-6-YL]-ACRYLAMIDE	0.283	0.908	1.266	1.045	-1.084	0.552	0.823	0.755	0.811	0.099
DB09205	Moxisylyte	0.283	0.495	1.115	-1.072	0.184	0.487	0.577	0.696	0.218	0.46
DB11615		0.285	0.78	-0.761	-0.055	0.604	0.604	0.809	0.14	0.364	0.729
DB00009	Alteplase	0.288	0.295	0.14	0.296	-0.034	0.583	0.569	0.192	0.45	0.405
DB00015	Reteplase	0.288	0.295	0.14	0.296	-0.034	0.583	0.569	0.192	0.45	0.405
DB00029	Anistreplase	0.288	0.295	0.14	0.296	-0.034	0.583	0.569	0.192	0.45	0.405
DB00427	Triprolidine	0.288	0.452	1.425	1.014	0.118	0.704	0.709	0.854	0.68	0.486
DB00385	Valrubicin	0.289	0.213	-0.558	-0.433	1.808	0.556	0.51	0.143	0.26	0.976
DB06480	Prucalopride	0.289	0.673	-0.083	0.306	-0.264	0.757	0.833	0.303	0.449	0.336
DB08165	indane-5-sulfonamide	0.289	0.795	0.693	-1.094	-0.729	0.687	0.854	0.678	0.071	0.137
DB08687	FG-2216	0.29	0.585	1.073	0.329	-0.437	0.577	0.667	0.792	0.492	0.265
DB01356	Lithium cation	0.292	0.015	0.808	0.895	0.264	0.562	0.427	0.668	0.752	0.502
DB01946	3-[1-(3-Aminopropyl)-1h-Indol-3-Yl]-4-(1-Methyl-1h-Indol-3-Yl)-1h-Pyrrole-2,5-Dione	0.292	0.662	-2.058	0.194	0.71	0.581	0.717	0.01	0.415	0.679
DB04786	Suramin	0.295	-0.104	0.519	1.285	0.864	0.56	0.356	0.598	0.879	0.79
DB03929	D-Serine	0.299	0.652	1.039	-0.094	-1.126	0.685	0.757	0.748	0.381	0.078
DB00999	Hydrochlorothiazide	0.301	0.911	0.985	-0.393	-0.934	0.565	0.761	0.764	0.236	0.124
DB01422	Nitroxoline	0.302	0.084	-0.335	-0.003	1.049	0.55	0.449	0.29	0.422	0.841
DB04099	Deamido-Nad+	0.302	0.831	1.905	-1.028	-0.326	0.705	0.812	0.927	0.079	0.275

DB08895	Tofacitinib	0.302	-0.698	0.52	-0.179	-0.987	0.586	0.161	0.632	0.383	0.124
DB00254	Doxycycline	0.305	0.653	-2.929	0.966	1.21	0.558	0.68	0.005	0.737	0.849
DB00898	Ethanol	0.305	1.253	2.825	0.418	-0.17	0.581	0.931	0.995	0.615	0.36
DB01375	Aluminium monostearate	0.306	-0.277	0.995	1.397	0.257	0.591	0.347	0.75	0.823	0.501
DB07013	TERT-BUTYL 4-({[4-(BUT-2-YN-1-YLAMINO)PHENYL]SULFONYL}METHYL)-4-[(HYDROXYAMINO)CARBONYL]PIPERIDINE-1-CARBOXYLATE	0.307	0.74	-0.498	0.805	0.042	0.585	0.744	0.072	0.58	0.412
DB07233	N-{[4-(but-2-yn-1-yloxy)phenyl]sulfonyl}-5-methyl-D-tryptophan	0.307	0.74	-0.498	0.805	0.042	0.585	0.744	0.072	0.58	0.412
DB13025	Tiapride	0.309	0.862	3.531	0.185	-0.837	0.669	0.809	0.992	0.313	0.126
DB04522	Dexfosfoserine	0.31	0.775	-0.508	0.787	-0.218	0.561	0.77	0.223	0.738	0.331
DB08036	6,7,12,13-tetrahydro-5H-indolo[2,3-a]pyrrolo[3,4-c]carbazol-5-one	0.311	0.726	-0.558	-0.102	-0.167	0.561	0.779	0.208	0.36	0.32
DB00740	Riluzole	0.313	0.168	0.343	0.521	-0.456	0.593	0.497	0.54	0.556	0.222
DB00554	Piroxicam	0.317	0.516	2.275	0.266	-0.574	0.604	0.654	0.975	0.497	0.211
DB01587	Ketazolam	0.323	0.123	1.816	0.246	-0.17	0.62	0.496	0.923	0.398	0.341
DB03959	N,O6-Disulfo-Glucosamine	0.323	0.938	1.725	0.809	-1.083	0.648	0.835	0.912	0.664	0.096
DB07218	6-CHLORO-9-HYDROXY-1,3-DIMETHYL-1,9-DIHYDRO-4H-PYRAZOLO[3,4-B]QUINOLIN-4-ONE	0.327	-0.041	0.512	0.674	0.768	0.578	0.431	0.314	0.688	0.718
DB07272	N-(4-AMINO-5-CYANO-6-ETHOXYPYRIDIN-2-YL)-2-(4-BROMO-2,5-DIMETHOXYPHENYL)ACETAMIDE	0.327	-0.041	0.512	0.674	0.768	0.578	0.431	0.314	0.688	0.718
DB09079	Nintedanib	0.328	-0.854	0.123	0.353	1.014	0.582	0.1	0.51	0.614	0.884
DB00494	Entacapone	0.329	0.721	1.071	0.315	-0.366	0.728	0.839	0.786	0.365	0.272
DB13150	Coagulation factor VII human	0.33	0.857	1.159	0.262	-0.739	0.745	0.861	0.796	0.36	0.147
DB00649	Stavudine	0.337	0.066	1.729	-0.029	-0.19	0.791	0.582	0.934	0.385	0.395
DB00879	Emtricitabine	0.337	0.066	1.729	-0.029	-0.19	0.791	0.582	0.934	0.385	0.395
DB01048	Abacavir	0.337	0.066	1.729	-0.029	-0.19	0.791	0.582	0.934	0.385	0.395
DB03431		0.339	-0.213	-0.306	1.091	0.357	0.6	0.314	0.325	0.861	0.584
DB09298	Silibinin	0.34	0.688	1.517	0.442	-1.012	0.604	0.716	0.894	0.572	0.106
DB08882	Linagliptin	0.341	0.731	-2.634	0.496	0.306	0.648	0.752	0.001	0.454	0.535
DB01026	Ketoconazole	0.347	0.475	-0.221	1.839	1.074	0.613	0.66	0.332	0.973	0.88
DB01381	Ginkgo biloba	0.347	0.199	2.238	0.665	-0.174	0.641	0.544	0.967	0.617	0.335
DB00415	Ampicillin	0.349	1.169	-0.823	0.341	-0.599	0.609	0.856	0.155	0.562	0.219
DB03448	2'-Deoxyuridine 3'-Monophosphate	0.349	0.474	0.818	-0.016	0.115	0.555	0.579	0.678	0.517	0.517
DB08339	6-(2,6-DICHLOROPHENYL)-2-{[3-(HYDROXYMETHYL)PHENYL]AMINO}-8-METHYLPYRIDO[2,3-D]PYRIMIDIN-7(8H)-ONE	0.349	0.551	0.695	0.171	-0.546	0.594	0.688	0.61	0.499	0.192
DB00176	Fluvoxamine	0.352	0.498	0.653	0.408	-0.484	0.603	0.638	0.647	0.392	0.235
DB01042	Melphalan	0.352	0.688	-0.46	1.043	0.654	0.592	0.705	0.249	0.746	0.688
DB06616	Bosutinib	0.352	-0.279	0.995	0.161	0.624	0.592	0.255	0.82	0.474	0.784
DB04367	Debromohymenialdisine	0.355	1.685	-1.769	-0.52	-1.09	0.599	0.951	0.016	0.224	0.084
DB03604	Tiratricol	0.356	0.957	0.35	-0.157	-0.909	0.596	0.795	0.252	0.352	0.136
DB00119	Pyruvic acid	0.358	0.219	-0.842	-0.33	-0.579	0.61	0.539	0.117	0.282	0.235
DB00441	Gemcitabine	0.358	0.743	-1.542	-0.258	0.685	0.593	0.762	0.043	0.304	0.751
DB01235	Levodopa	0.358	0.894	1.439	0.185	-0.235	0.611	0.809	0.894	0.529	0.314
DB00728	Rocuronium	0.368	0.581	-0.543	0.12	1.206	0.591	0.699	0.208	0.453	0.912
DB07655	3-AMINO-3-BENZYL-[4.3.0]BICYCLO-1,6-DIAZANONAN-2-ONE	0.369	0.599	-0.765	-0.283	0.38	0.603	0.72	0.119	0.297	0.592
DB00342	Terfenadine	0.371	0.091	0.533	1.517	0.27	0.612	0.471	0.63	0.929	0.536
DB00564	Carbamazepine	0.373	0.597	-0.53	0.448	0.321	0.598	0.711	0.218	0.587	0.551
DB03162	4-Hydroxy-1,2,5-Thiadiazole-3-Carboxylic Acid	0.373	0.447	1.915	-1.281	-1.327	0.703	0.686	0.953	0.059	0.049

DB03322	Dexpropranolol	0.373	0.35	0.621	0.454	0.106	0.527	0.511	0.609	0.577	0.461
DB03940	Oxamic Acid	0.373	0.447	1.915	-1.281	-1.327	0.703	0.686	0.953	0.059	0.049
DB04571	Trioxsalen	0.373	0.541	0.293	0.481	-0.064	0.711	0.751	0.479	0.437	0.401
DB04315	Guanosine-5'-Diphosphate	0.376	0.395	-0.597	-0.433	0.635	0.621	0.613	0.212	0.247	0.705
DB08183		0.38	-0.669	0.922	-0.753	-0.527	0.623	0.193	0.778	0.161	0.237
DB08312	6-CYCLOHEXYLMETHYLOXY-5-NITROSO-PYRIMIDINE-2,4-DIAMINE	0.381	0.342	-0.866	0.367	0.772	0.625	0.595	0.101	0.56	0.759
DB00156	L-Threonine	0.382	0.518	1.511	0.593	-0.363	0.68	0.673	0.877	0.473	0.267
DB00366	Doxylamine	0.382	0.744	1.197	1	-0.561	0.653	0.758	0.795	0.672	0.221
DB03003	Glutathione Sulfonic Acid	0.383	0.475	1.319	-0.703	-1.151	0.696	0.71	0.845	0.131	0.042
DB08239	(2S)-4-(2,5-DIFLUOROPHENYL)-N-METHYL-2-PHENYL-N-PIPERIDIN-4-YL-2,5-DIHYDRO-1H-PYRROLE-1-CARBOXAMIDE	0.383	0.79	1.755	-0.384	-0.456	0.757	0.86	0.919	0.231	0.239
DB08244	(1S)-1-CYCLOPROPYL-2-[(2S)-4-(2,5-DIFLUOROPHENYL)-2-PHENYL-2,5-DIHYDRO-1H-PYRROL-1-YL]-2-OXOETHANAMINE	0.383	0.79	1.755	-0.384	-0.456	0.757	0.86	0.919	0.231	0.239
DB08980	Fendiline	0.383	1.078	-0.991	0.252	-0.245	0.8	0.914	0.05	0.42	0.337
DB09054	Idelalisib	0.387	0.404	-0.806	0.179	0.447	0.627	0.632	0.104	0.476	0.618
DB09166	Etizolam	0.387	0.473	2.615	-0.104	-0.321	0.712	0.71	0.979	0.259	0.288
DB00643	Mebendazole	0.39	0.808	-0.05	-0.047	0.13	0.624	0.79	0.302	0.402	0.466
DB00409	Remoxipride	0.391	0.405	2.432	0.515	-1.274	0.614	0.608	0.974	0.432	0.061
DB00595	Oxytetracycline	0.392	0.23	-1.357	0.391	1.25	0.637	0.546	0.071	0.626	0.879
DB07364	6-PHENYL[5H]PYRROLO[2,3-B]PYRAZINE	0.393	0.045	1.577	1.558	-0.288	0.627	0.427	0.982	0.915	0.266
DB11617	Aclarubicin	0.394	0.837	-0.1	0.36	0.178	0.86	0.897	0.363	0.653	0.739
DB00675	Tamoxifen	0.395	0.389	0.162	1.046	0.645	0.612	0.587	0.52	0.877	0.731
DB03159	CRA_8696	0.395	0.447	-0.291	-0.251	0.098	0.615	0.606	0.144	0.333	0.431
DB08881	Vemurafenib	0.396	-0.18	1.377	0.982	0.578	0.617	0.374	0.954	0.775	0.66
DB00293	Raltitrexed	0.397	0.671	-0.216	0.04	-1.423	0.701	0.78	0.524	0.368	0.044
DB01129	Rabeprazole	0.397	0.53	0.559	-0.452	1.012	0.612	0.68	0.585	0.273	0.829
DB00081	Tositumomab	0.398	0.334	0.04	-0.186	1.169	0.591	0.582	0.35	0.325	0.839
DB01097	Leflunomide	0.398	0.257	0.27	0.205	0.309	0.625	0.553	0.452	0.507	0.55
DB07249	N-(5-chloro-1,3-benzodioxol-4-yl)-6-methoxy-7-(3-piperidin-1-ylpropoxy)quinazolin-4-amine	0.398	0.913	0.701	-0.155	-0.238	0.625	0.834	0.453	0.358	0.292
DB08841	Tyramine	0.398	0.853	0.564	0.654	-0.084	0.815	0.871	0.596	0.521	0.424
DB07118	Hymecromone	0.399	0.429	1.571	-0.404	-0.091	0.622	0.629	0.869	0.183	0.386
DB00795	Sulfasalazine	0.403	0.094	-0.524	-0.302	-0.892	0.635	0.475	0.222	0.316	0.127
DB08691	ethyl 3-[(E)-2-amino-1-cyanoethenyl]-6,7-dichloro-1-methyl-1H-indole-2-carboxylate	0.408	1.275	-0.536	-1.537	-0.861	0.605	0.867	0.229	0.03	0.134
DB00477	Chlorpromazine	0.413	0.539	1.74	2.398	0.641	0.627	0.685	0.938	0.998	0.728
DB00368	Norepinephrine	0.419	0.449	1.233	-0.097	-0.851	0.646	0.637	0.913	0.417	0.152
DB07010	N-BENZYL-4-[4-(3-CHLOROPHENYL)-1H-PYRAZOL-3-YL]-1H-PYRROLE-2-CARBOXAMIDE	0.419	0.24	1.339	0.54	0.501	0.656	0.558	0.916	0.605	0.66
DB03566	Spermidine	0.421	0.865	1.309	-0.396	-0.583	0.605	0.744	0.836	0.214	0.215
DB08873	Boceprevir	0.421	0.217	2.374	-0.101	1.754	0.618	0.514	0.982	0.379	0.955
DB00914	Phenformin	0.424	1.454	2.202	-0.319	-0.868	0.613	0.899	0.992	0.296	0.133
DB01162	Terazosin	0.426	-0.041	2.087	1.826	-0.683	0.628	0.409	0.977	0.967	0.166
DB01400	Neostigmine	0.426	0.678	0.479	-0.625	0.377	0.744	0.748	0.561	0.139	0.581
DB00093	Felypressin	0.429	0.63	-0.404	-0.939	0.576	0.541	0.644	0.348	0.296	0.585
DB00586	Diclofenac	0.43	-0.772	3.637	1.043	0.223	0.642	0.183	0.999	0.844	0.525
DB06820	Sulconazole	0.433	0.619	0.767	1.003	-0.04	0.641	0.705	0.717	0.818	0.408

DB07145	(2R)-N-HYDROXY-2-[(3S)-3-METHYL-3-{4-[(2-METHYLQUINOLIN-4-YL)METHOXY]PHENYL}-2-OXOPYRROLIDIN-1-YL]PROPANAMIDE	0.433	0.143	-1.823	0.579	0.448	0.631	0.513	0.022	0.58	0.574
DB01393	Bezafibrate	0.436	-0.031	1.508	0.715	0.198	0.623	0.432	0.935	0.686	0.508
DB01924	Benzhydroxamic Acid	0.436	0.29	0.732	-0.124	1.478	0.662	0.549	0.733	0.386	0.941
DB00936	Salicylic acid	0.438	1.063	2.155	-1.071	-0.844	0.622	0.819	0.963	0.101	0.144
DB09121	Aurothioglucose	0.438	0.793	0.638	0.742	-0.233	0.794	0.838	0.629	0.565	0.359
DB02058	SU4984	0.439	1.046	1.066	0.182	-1.403	0.669	0.863	0.796	0.454	0.046
DB03814	2-(N-Morpholino)-Ethanesulfonic Acid	0.439	-0.149	0.334	0.292	-0.374	0.625	0.387	0.452	0.489	0.28
DB01330	Cefotetan	0.441	0.407	-0.012	1.248	0.109	0.618	0.616	0.158	0.775	0.437
DB07959	3-(1H-BENZIMIDAZOL-2-YL)-1H-INDAZOLE	0.441	0.443	0.916	-0.398	0.246	0.672	0.625	0.546	0.268	0.52
DB07964	(3S)-4-{[4-(BUT-2-YNYLOXY)PHENYL]SULFONYL}-N-HYDROXY-2,2-DIMETHYLTHIOMORPHOLINE-3-CARBOXAMIDE	0.441	0.768	0.336	0.518	-0.397	0.658	0.761	0.492	0.442	0.268
DB03014		0.447	0.106	1.781	0.226	-0.29	0.649	0.49	0.956	0.514	0.336
DB00154	Dihomo-gamma-linolenic acid	0.458	0.814	3.215	-0.818	-0.6	0.743	0.826	0.991	0.114	0.197
DB00925	Phenoxybenzamine	0.459	0.397	0.667	1.959	-0.322	0.67	0.614	0.648	0.97	0.298
DB07835	N~3~-cyclopropyl-N~4~'-(cyclopropylmethyl)-6-methylbiphenyl-3,4'-dicarboxamide	0.459	0.466	1.447	0.452	-0.19	0.673	0.632	0.927	0.586	0.273
DB01113	Papaverine	0.46	0.743	-2.423	0.58	1.58	0.659	0.766	0.005	0.668	0.957
DB00471	Montelukast	0.462	0.91	0.406	1.278	-0.666	0.66	0.823	0.578	0.882	0.175
DB00774	Hydroflumethiazide	0.467	0.858	1.768	0.098	-0.653	0.622	0.757	0.93	0.411	0.174
DB00266	Dicoumarol	0.469	1.552	0.036	-0.43	-0.293	0.642	0.944	0.359	0.271	0.288
DB08030	3-[(4'-cyanobiphenyl-4-yl)oxy]-N-hydroxypropanamide	0.469	0.387	-0.337	0.964	0.795	0.652	0.594	0.099	0.649	0.712
DB00515	Cisplatin	0.471	0.713	1.365	1.566	-0.429	0.664	0.75	0.875	0.923	0.239
DB03783	Phenacetin	0.471	0.576	1.287	-0.336	0.236	0.539	0.59	0.748	0.375	0.458
DB07477	Felbinac	0.471	0.576	1.287	-0.336	0.236	0.539	0.59	0.748	0.375	0.458
DB06883	1-[1-(3-aminophenyl)-3-tert-butyl-1H-pyrazol-5-yl]-3-phenylurea	0.473	0.579	0.889	0.302	-0.13	0.666	0.705	0.578	0.534	0.313
DB00399	Zoledronic acid	0.474	0.549	-0.222	-0.128	0.706	0.642	0.643	0.168	0.324	0.683
DB06882	1-[1-(3-aminophenyl)-3-tert-butyl-1H-pyrazol-5-yl]-3-naphthalen-1-ylurea	0.477	0.257	0.987	0.724	0.064	0.661	0.524	0.626	0.706	0.429
DB06945	N-hydroxy-4-({4-[4-(trifluoromethyl)phenoxy]phenyl}sulfonyl)tetrahydro-2H-pyran-4-carboxamide	0.477	0.171	-0.595	1.106	0.747	0.638	0.491	0.118	0.755	0.7
DB11359	Guaiacol	0.478	0.148	2.105	-1.037	-0.73	0.697	0.525	0.969	0.082	0.149
DB05990	Obeticholic acid	0.479	0.839	0.923	-0.251	0.068	0.753	0.777	0.768	0.397	0.718
DB00906	Tiagabine	0.484	0.41	0.695	0.979	0.273	0.549	0.55	0.517	0.788	0.447
DB03946	3,4-Dihydroxybenzoic Acid	0.484	1.282	1.448	-1.137	-0.591	0.663	0.87	0.855	0.108	0.192
DB01044	Gatifloxacin	0.488	0.93	0.814	-0.986	-0.335	0.666	0.813	0.694	0.105	0.293
DB08930	Dolutegravir	0.489	0.793	1.357	-0.166	0.013	0.561	0.71	0.769	0.438	0.44
DB04725	Licofelone	0.491	0.751	1.654	-0.72	-0.435	0.737	0.754	0.913	0.141	0.256
DB01611	Hydroxychloroquine	0.492	0.668	0.991	1.31	-0.757	0.645	0.698	0.739	0.887	0.157
DB00125	L-Arginine	0.494	1.092	1.227	1.192	-2.206	0.65	0.822	0.83	0.861	0.009
DB00491	Miglitol	0.499	1.796	0.303	0.441	-1.854	0.667	0.952	0.488	0.561	0.024
DB06684	Vilazodone	0.5	0.721	1.733	0.093	-0.399	0.731	0.747	0.913	0.316	0.254

DB02367	(1n)-4-N-Butoxyphenylsulfonyl-(2r)-N-Hydroxycarboxamido-(4s)-Methanesulfonylamino-Pyrrolidine	0.501	0.506	-0.41	0.846	0.694	0.66	0.649	0.099	0.589	0.66
DB01025	Amlexanox	0.504	1.242	-0.322	0.949	-0.812	0.654	0.872	0.206	0.738	0.143
DB00549	Zafirlukast	0.505	0.553	2.209	1.46	-0.461	0.688	0.699	0.98	0.907	0.226
DB04338	SB220025	0.506	0.141	0.89	0.567	0.267	0.673	0.464	0.542	0.649	0.51
DB08521	4-[5-(4-FLUORO-PHENYL)-2-(4-METHANESULFINYL-PHENYL)-3H-IMIDAZOL-4-YL]-PYRIDINE	0.506	0.141	0.89	0.567	0.267	0.673	0.464	0.542	0.649	0.51
DB00214	Torasemide	0.507	0.58	1.05	0.179	0.273	0.566	0.641	0.872	0.429	0.447
DB06201	Rufinamide	0.508	1.278	-0.912	0.967	-0.117	0.793	0.919	0.067	0.747	0.402
DB01197	Captopril	0.51	0.132	1.538	1.087	1.145	0.646	0.505	0.911	0.844	0.827
DB02481	N-Benzylformamide	0.51	0.619	1.416	0.312	0.237	0.613	0.645	0.877	0.598	0.567
DB08080	LATRUNCULIN B	0.52	1.033	2.421	0.103	-1.197	0.708	0.835	0.979	0.404	0.073
DB08889	Carfilzomib	0.52	0.107	-0.256	-0.13	0.398	0.699	0.485	0.237	0.397	0.603
DB00583	Levocarnitine	0.521	0.312	1.175	0.574	-0.671	0.65	0.558	0.808	0.568	0.167
DB02071	WAY-151693	0.521	1.068	0.423	0.684	-0.246	0.701	0.843	0.52	0.516	0.337
DB00574	Fenfluramine	0.522	0	0.331	1.737	-0.776	0.66	0.441	0.539	0.956	0.168
DB01112	Cefuroxime	0.523	0.866	-0.052	0.231	0.303	0.648	0.77	0.139	0.404	0.527
DB02350	N-Hydroxy-4-[(4-Methoxylphenyl)Sulfonyl]-2,2-Dimethyl-Hexahydro-1,4-Thiazepine-3(S)-Carboxamide	0.523	0.321	-0.425	1.04	0.949	0.641	0.559	0.105	0.694	0.766
DB08567		0.525	1.147	0.583	0.427	-0.68	0.775	0.902	0.638	0.523	0.142
DB07767	Ferulic acid	0.526	1.277	0.925	-1.184	-0.667	0.673	0.873	0.722	0.099	0.186
DB08056	N-(2,6-dimethylphenyl)-5-phenylimidazo[1,5-a]pyrazin-8-amine	0.526	0.727	0.667	0.52	0.024	0.725	0.771	0.399	0.617	0.394
DB00133	Serine	0.527	-0.248	1.692	1.39	0.486	0.659	0.339	0.916	0.886	0.59
DB03148	Phosphomethylphosphonic Acid Adenosyl Ester	0.528	0.679	1.157	0.393	0.147	0.64	0.677	0.787	0.649	0.563
DB00636	Clofibrate	0.536	0.845	0.66	0.055	0.49	0.828	0.851	0.762	0.576	0.832
DB09422	Soybean oil	0.536	0.845	0.66	0.055	0.49	0.828	0.851	0.762	0.576	0.832
DB00252	Phenytoin	0.538	0.794	2.542	2.231	-1.522	0.654	0.739	0.988	0.994	0.058
DB00942	Cycrimine	0.541	0.839	0.358	-0.899	0.496	0.594	0.754	0.515	0.285	0.566
DB01852	Kaempherol	0.541	0.233	-1.692	0.898	0.819	0.698	0.529	0.028	0.778	0.789
DB00422	Methylphenidate	0.545	0.862	2.433	0.098	-0.523	0.746	0.795	0.976	0.281	0.212
DB06769	Bendamustine	0.546	0.453	0.717	-0.022	1.413	0.709	0.644	0.721	0.427	0.937
DB08809	Dichloroacetic acid	0.547	1.101	-0.108	0.455	-0.485	0.682	0.836	0.167	0.51	0.24
DB08828	Vismodegib	0.547	0.911	-0.111	-0.833	-0.139	0.696	0.8	0.153	0.127	0.359
DB07537	N'-(6-aminopyridin-3-yl)-N-(2-cyclopentylethyl)-4-methyl-benzene-1,3-dicarboxamide	0.549	0.443	1.045	-0.131	0.221	0.697	0.656	0.63	0.382	0.493
DB00401	Nisoldipine	0.55	0.093	1.139	1.526	0.265	0.666	0.486	0.815	0.908	0.507
DB00802	Alfentanil	0.551	1.017	-0.361	0.766	0.121	0.827	0.892	0.08	0.581	0.521
DB01209	Dezocine	0.552	1.059	-0.389	0.671	-0.005	0.868	0.905	0.087	0.542	0.491
DB02656	LY-294002	0.554	-0.679	-0.39	0.894	2.19	0.717	0.152	0.268	0.773	0.991
DB06896	1-(4-fluorophenyl)-N-[3-fluoro-4-(1H-pyrrolo[2,3-b]pyridin-4-yloxy)phenyl]-2-oxo-1,2-dihydropyridine-3-carboxamide	0.554	0.498	-1.143	0.51	0.691	0.679	0.638	0.05	0.605	0.693
DB01433	Methadyl acetate	0.557	1.192	-0.304	0.803	-0.15	0.822	0.914	0.083	0.586	0.401
DB00653	Magnesium sulfate	0.559	1.085	1.186	0.902	-0.597	0.713	0.834	0.806	0.716	0.196
DB00763	Methimazole	0.561	0.732	0.792	0.667	0.426	0.76	0.832	0.782	0.73	0.639

DB07211	(2R)-2-(5-CHLORO-2-THIENYL)-N-{(3S)-1-[(1S)-1-METHYL-2-MORPHOLIN-4-YL-2-OXOETHYL]-2-OXOPYRROLIDIN-3-YL}PROPENE-1-SULFONAMIDE	0.562	0.735	1.636	-0.612	0.241	0.711	0.736	0.879	0.146	0.511
DB01576	Dextroamphetamine	0.565	0.566	0.98	0.168	-0.844	0.737	0.686	0.763	0.309	0.115
DB08689	UBIQUINONE-1	0.568	0.756	0.488	1.005	-0.473	0.728	0.76	0.507	0.683	0.243
DB03977	N-Trimethyllysine	0.569	0.191	0.554	1.77	0.677	0.731	0.528	0.362	0.926	0.742
DB09473	Indium In-111 oxyquinoline	0.571	0.544	-0.219	0.095	1.092	0.711	0.664	0.256	0.445	0.856
DB00279	Liothyronine	0.572	0.239	1.077	0.573	1.492	0.706	0.526	0.778	0.652	0.954
DB07752		0.576	1.352	2.293	1.768	-0.713	0.675	0.891	0.995	0.926	0.18
DB00819	Acetazolamide	0.577	0.877	0.523	0.269	0.169	0.681	0.768	0.604	0.518	0.466
DB01962	Phosphonotyrosine	0.577	1.135	-0.713	0.197	0.134	0.737	0.892	0.107	0.485	0.448
DB08907	Canagliflozin	0.579	0.751	1.309	0.452	0.44	0.6	0.679	0.77	0.588	0.549
DB00822	Disulfiram	0.583	0.138	-0.576	1.039	0.44	0.705	0.483	0.238	0.843	0.622
DB08597	6-[4-(2-piperidin-1-ylethoxy)phenyl]-3-pyridin-4-ylpyrazolo[1,5-a]pyrimidine	0.583	1.278	1.264	-0.337	0.217	0.697	0.906	0.801	0.304	0.536
DB11561	Aminacrine	0.585	0.084	1.319	0.244	1.24	0.703	0.451	0.898	0.51	0.888
DB09016	Butriptyline	0.586	0.817	0.402	0.094	-0.901	0.799	0.813	0.562	0.284	0.116
DB00167	Isoleucine	0.592	1.459	0.718	0.195	-1.682	0.681	0.896	0.639	0.414	0.03
DB02944		0.594	-0.115	1.171	-0.121	1.085	0.699	0.388	0.849	0.384	0.86
DB01092	Ouabain	0.596	0.993	-1.073	-1.237	1.083	0.719	0.876	0.104	0.081	0.875
DB09421	Protirelin	0.598	0.707	1.05	-0.141	0.344	0.626	0.72	0.872	0.329	0.475
DB00512	Vancomycin	0.602	0.75	1.778	-0.854	0.717	0.634	0.674	0.873	0.171	0.653
DB04875	Pralnacasan	0.602	1.301	1.141	-0.251	-1.384	0.713	0.888	0.795	0.319	0.047
DB00943	Zalcitabine	0.604	0.352	-0.68	-0.516	0.38	0.709	0.597	0.194	0.223	0.542
DB00272	Betazole	0.605	0.816	1.138	0.667	0.36	0.796	0.88	0.906	0.73	0.586
DB08806	Roxatidine acetate	0.605	0.816	1.138	0.667	0.36	0.796	0.88	0.906	0.73	0.586
DB08568	(2S)-1-{[5-(3-METHYL-1H-INDAZOL-5-YL)PYRIDIN-3-YL]OXY}-3-PHENYLPROPAN-2-AMINE	0.607	0.047	-1.322	0.093	0.176	0.708	0.423	0.071	0.454	0.51
DB09211	Limaprost	0.608	1	-0.244	0.595	-0.036	0.811	0.862	0.251	0.532	0.418
DB00144	Phosphatidyl serine	0.609	1.244	-1.357	-0.256	0.396	0.741	0.903	0.065	0.273	0.583
DB07950	Indoleacetic acid	0.61	0.006	0.013	1.47	1.285	0.751	0.463	0.315	0.871	0.886
DB00390	Digoxin	0.611	0.497	-1.37	-0.879	0.817	0.736	0.674	0.057	0.14	0.762
DB04513	N-(6-Aminohexyl)-5-Chloro-1-Naphthalenesulfonamide	0.611	0.311	0.42	1.661	0.682	0.779	0.571	0.361	0.923	0.719
DB01268	Sunitinib	0.614	-0.569	-0.121	0.179	1.074	0.736	0.157	0.428	0.469	0.964
DB04898	Ximelagatran	0.616	0.675	0.693	-0.223	0.057	0.702	0.696	0.622	0.338	0.426
DB05521	Telaprevir	0.617	0.348	1.017	-0.259	0.6	0.678	0.589	0.782	0.311	0.663
DB00182	Amphetamine	0.62	0.936	0.163	0.566	-1.463	0.698	0.801	0.484	0.629	0.05
DB05265	Ecabet	0.621	0.58	0.695	0.979	0.485	0.642	0.641	0.517	0.788	0.571
DB06712	Nilvadipine	0.625	0.902	1.922	1.185	-0.693	0.687	0.79	0.946	0.83	0.189
DB04216	Quercetin	0.629	-0.447	-0.139	0.83	1.641	0.699	0.224	0.407	0.811	0.979
DB09068	Vortioxetine	0.629	0.252	-0.781	1.91	-0.082	0.693	0.537	0.14	0.97	0.395
DB00208	Ticlopidine	0.633	0.719	2.663	-0.078	-0.67	0.767	0.806	0.995	0.361	0.174
DB00219	Oxyphenonium	0.633	0.823	0.369	-0.54	-0.281	0.787	0.812	0.484	0.167	0.308
DB03585	Oxyphenbutazone	0.635	0.413	1.674	0.729	1.056	0.744	0.621	0.954	0.704	0.848
DB03777	Rbt205 Inhibitor	0.635	-0.031	-0.472	-0.582	1.81	0.746	0.386	0.214	0.236	0.967
DB07080	N-(2,2,2-TRIFLUOROETHYL)-N-{4-[2,2,2-TRIFLUORO-1-HYDROXY-1-(TRIFLUOROMETHYL)ETHYL]PHENYL}BENZENESULFONAMIDE	0.636	1.005	0.424	2.136	-1.445	0.707	0.798	0.341	0.968	0.06
DB00479	Amikacin	0.642	0.806	2.013	0.067	-0.006	0.779	0.795	0.923	0.54	0.649
DB07514	3-(2-aminoquinazolin-6-yl)-1-(3,3-dimethylindolin-6-yl)-4-methylpyridin-2(1H)-one	0.644	0.849	-0.529	0.261	0.366	0.754	0.818	0.159	0.522	0.552
DB08621	Thiamphenicol	0.649	0.9	0.792	0.667	0.426	0.818	0.906	0.782	0.73	0.639

DB01137	Levofloxacin	0.652	0.466	1.156	-0.284	0.787	0.693	0.609	0.799	0.325	0.699
DB07528	3-(2-aminoquinazolin-6-yl)-4-methyl-1-[3-(trifluoromethyl)phenyl]pyridin-2(1H)-one	0.655	0.64	0.037	0.353	0.447	0.741	0.724	0.343	0.578	0.631
DB04842	Fluspirilene	0.657	0.045	1.699	2.188	0.46	0.722	0.467	0.973	0.994	0.611
DB01002	Levobupivacaine	0.661	1.229	-1.847	-0.083	0.678	0.694	0.86	0.017	0.34	0.666
DB06186	Ipilimumab	0.663	0.91	0.184	-0.087	0.65	0.642	0.72	0.563	0.556	0.64
DB00089	Capromab pendetide	0.664	0.537	-0.293	0.403	0.748	0.67	0.602	0.296	0.518	0.704
DB00713	Oxacillin	0.665	1.064	1.583	-0.617	-0.641	0.726	0.834	0.901	0.16	0.2
DB09289	Tianeptine	0.665	1.281	-0.455	0.721	0.013	0.891	0.923	0.068	0.561	0.469
DB03740	N-acetyl-alpha-D-glucosamine	0.667	1.184	1.279	0.588	-0.856	0.727	0.855	0.833	0.561	0.148
DB00013	Urokinase	0.673	-0.091	0.226	0.778	1.38	0.724	0.413	0.261	0.734	0.9
DB00031	Tenecteplase	0.675	0.16	0.733	0.455	0.79	0.717	0.521	0.533	0.62	0.746
DB02587	Colforsin	0.679	0.693	1.209	0.428	0.098	0.767	0.747	0.809	0.496	0.467
DB01195	Flecainide	0.68	-0.082	2.642	1.967	0.432	0.702	0.396	0.986	0.991	0.585
DB04763	1-N-(4-SULFAMOYLPHENYL-ETHYL)-2,4,6-TRIMETHYLPYRIDINIUM	0.68	1.222	0.995	0.521	-0.329	0.788	0.905	0.761	0.586	0.271
DB06856	6-FLUORO-2-[2-HYDROXY-3-(2-METHYL-CYCLOHEXYLOXY)-PHENYL]-1H-INDOLE-5-CARBOXAMIDINE	0.681	1.378	-0.278	-0.192	-0.569	0.727	0.898	0.087	0.252	0.212
DB01179	Podofilox	0.682	0.364	-0.631	-0.014	0.399	0.751	0.604	0.179	0.442	0.597
DB00251	Terconazole	0.688	0.428	1.043	1.052	0.267	0.774	0.624	0.803	0.832	0.55
DB00468	Quinine	0.688	0.776	0.923	0.668	-0.458	0.722	0.738	0.871	0.661	0.267
DB03255	Phenol	0.688	0.452	1.996	-0.85	-0.242	0.719	0.618	0.959	0.135	0.308
DB07574	2-MERCAPTO-N-[1,2,3,10-TETRAMETHOXY-9-OXO-5,6,7,9-TETRAHYDRO-BENZO[A]HEPTALEN-7-YL]ACETAMIDE	0.692	1.015	2.03	0.565	-0.707	0.807	0.859	0.995	0.65	0.155
DB09069	Trimetazidine	0.692	0.913	0.758	0.333	-0.282	0.803	0.827	0.674	0.392	0.309
DB00036	Coagulation factor VIIa Recombinant Human	0.694	0.979	0.077	0.516	-0.391	0.748	0.814	0.402	0.438	0.244
DB01890	N,N-Bis(3-(D-gluconamido)propyl)deoxycholamide	0.697	1.254	0.326	0.828	0.13	0.907	0.933	0.527	0.761	0.589
DB08875	Cabozantinib	0.697	0.601	-0.865	0.883	0.87	0.727	0.672	0.087	0.737	0.759
DB09331	Daratumumab	0.7	0.793	-0.592	0.387	0.573	0.657	0.71	0.321	0.563	0.583
DB00398	Sorafenib	0.701	-0.05	-0.205	0.676	0.118	0.743	0.366	0.378	0.787	0.49
DB00589	Lisuride	0.704	0.284	1.651	2.169	-0.144	0.721	0.572	0.925	0.99	0.358
DB07235	N-[(1S)-2-AMINO-1-(2,4-DICHLOROBENZYL)ETHYL]-5-[2-(METHYLAMINO)PYRIMIDIN-4-YL]THIOPHENE-2-CARBOXAMIDE	0.705	0.552	-0.707	0.935	0.83	0.769	0.688	0.169	0.783	0.789
DB00842	Oxazepam	0.706	0.671	0.267	-0.159	1.114	0.781	0.747	0.479	0.364	0.866
DB00531	Cyclophosphamide	0.708	1.045	-0.86	1.519	0.355	0.741	0.823	0.148	0.856	0.55
DB01544	Flunitrazepam	0.711	0.177	2.066	-0.006	0.072	0.736	0.513	0.957	0.334	0.439
DB05665		0.712	0.81	2.525	-0.33	1.613	0.713	0.737	0.986	0.293	0.929
DB01039	Fenofibrate	0.713	-0.192	1.501	1.661	-0.81	0.728	0.384	0.906	0.942	0.165
DB01006	Letrozole	0.714	1.184	1.853	0.054	0.454	0.721	0.803	0.925	0.659	0.705
DB00255	Diethylstilbestrol	0.718	0.943	0.157	0.676	-0.134	0.77	0.827	0.515	0.73	0.344
DB01622	Thioproperazine	0.72	0.201	2.334	1.197	-0.277	0.736	0.513	0.977	0.76	0.321
DB00165	Pyridoxine	0.721	-0.161	1.223	1.014	0.587	0.734	0.383	0.834	0.685	0.63
DB00134	Methionine	0.722	0.682	0.791	0.585	-0.363	0.745	0.724	0.668	0.509	0.281
DB01346	Quinidine barbiturate	0.727	0.706	2.013	0.322	-0.148	0.776	0.754	0.945	0.412	0.343
DB01204	Mitoxantrone	0.73	0.868	-0.309	-0.298	0.062	0.76	0.801	0.323	0.297	0.453
DB04242	P-Hydroxybenzoic Acid	0.731	1.368	1.42	-0.635	-0.893	0.727	0.895	0.873	0.168	0.129
DB00524	Metolazone	0.734	1.558	1.005	-0.516	-1.194	0.729	0.933	0.77	0.193	0.083

DB08424	[5-AMINO-1-(4-FLUOROPHENYL)-1H-PYRAZOL-4-YL](3-{[(2R)-2,3-DIHYDROXYPROPYL]OXY}PHENYL)METHANONE	0.735	0.034	0.801	1.197	0.718	0.78	0.462	0.582	0.851	0.726
DB01149	Nefazodone	0.736	0.64	-0.072	0.456	0.838	0.814	0.724	0.362	0.603	0.794
DB01186	Pergolide	0.738	-0.267	2.318	1.951	0.27	0.737	0.332	0.978	0.992	0.504
DB00734	Risperidone	0.741	-0.045	1.478	2.505	-0.067	0.749	0.417	0.901	0.992	0.417
DB01064	Isoprenaline	0.741	0.931	-1.004	0.697	1.114	0.797	0.853	0.13	0.751	0.873
DB04160	Pyrophosphoric acid	0.741	0.886	1.782	0.606	-0.321	0.77	0.785	0.906	0.487	0.289
DB00259	Sulfanilamide	0.742	1.455	1.578	-0.299	-0.508	0.733	0.894	0.904	0.305	0.231
DB00311	Ethoxzolamide	0.742	1.455	1.578	-0.299	-0.508	0.733	0.894	0.904	0.305	0.231
DB00391	Sulpiride	0.742	0.687	3.284	-0.637	-0.352	0.725	0.706	0.997	0.187	0.263
DB00869	Dorzolamide	0.742	1.455	1.578	-0.299	-0.508	0.733	0.894	0.904	0.305	0.231
DB01144	Diclofenamide	0.742	1.455	1.578	-0.299	-0.508	0.733	0.894	0.904	0.305	0.231
DB01194	Brinzolamide	0.742	1.455	1.578	-0.299	-0.508	0.733	0.894	0.904	0.305	0.231
DB08329	Sulthiame	0.742	1.455	1.578	-0.299	-0.508	0.733	0.894	0.904	0.305	0.231
DB01110	Miconazole	0.743	0.418	0.549	1.224	0.355	0.79	0.637	0.678	0.936	0.585
DB11093	Calcium Citrate	0.743	-0.095	2.04	0.853	1.733	0.792	0.369	0.975	0.792	0.963
DB03468	1,2,3,4-Tetrahydro-Isoquinoline-7-Sulfonic Acid Amide	0.745	0.981	1.128	-0.582	0.578	0.719	0.773	0.809	0.238	0.704
DB03847	Gamma-Carboxy-Glutamic Acid	0.745	0.634	0.38	0.634	0.12	0.751	0.71	0.485	0.479	0.458
DB08550	7,8-Dichloro-1,2,3,4-tetrahydroisoquinoline	0.745	0.981	1.128	-0.582	0.578	0.719	0.773	0.809	0.238	0.704
DB00206	Reserpine	0.746	0.549	1.13	0.498	0.437	0.75	0.671	0.923	0.656	0.628
DB08985	Etilefrine	0.746	1.05	0.098	-0.409	0.701	0.714	0.781	0.567	0.393	0.71
DB00277	Theophylline	0.747	0.341	-0.079	0.623	2.314	0.801	0.575	0.365	0.666	0.998
DB07006	9-HYDROXY-6-(3-HYDROXYPROPYL)-4-(2-METHOXYPHENYL)PYRROLO[3,4-C]CARBAZOLE-1,3(2H,6H)-DIONE	0.748	1.104	-0.639	-1.003	-0.133	0.758	0.842	0.202	0.107	0.363
DB07265	3-(9-HYDROXY-1,3-DIOXO-4-PHENYL-2,3-DIHYDROPYRROLO[3,4-C]CARBAZOL-6(1H)-YL)PROPANOIC ACID	0.748	1.104	-0.639	-1.003	-0.133	0.758	0.842	0.202	0.107	0.363
DB00102	Becaplermin	0.75	0.865	1.29	1.593	-1.56	0.75	0.766	0.89	0.865	0.038
DB00995	Auranofin	0.75	0.71	0.617	0.199	0.524	0.806	0.746	0.607	0.507	0.653
DB02325	Isopropyl alcohol	0.75	0.059	0.081	0.163	0.535	0.738	0.449	0.368	0.487	0.65
DB00091	Ciclosporin	0.752	1.097	-0.115	-0.509	-0.753	0.738	0.82	0.142	0.23	0.17
DB00939	Meclofenamic acid	0.752	0.447	0.557	0.462	1.041	0.805	0.629	0.612	0.615	0.847
DB04844	Tetrabenazine	0.753	0.954	4.403	0.552	-0.357	0.789	0.794	0.999	0.46	0.283
DB00425	Zolpidem	0.754	0.863	1.909	0.19	0.208	0.799	0.78	0.931	0.349	0.479
DB00972	Azelastine	0.755	0.702	0.03	0.401	-0.924	0.769	0.703	0.37	0.468	0.119
DB06612	Mepolizumab	0.757	0.834	0.695	0.979	0.415	0.738	0.819	0.517	0.788	0.515
DB00831	Trifluoperazine	0.758	-0.223	1.793	1.779	1.106	0.802	0.318	0.951	0.963	0.88
DB01059	Norfloxacin	0.762	0.188	2.033	-0.285	1.392	0.757	0.517	0.976	0.296	0.903
DB08916	Afatinib	0.765	0.48	2.32	-0.301	1.035	0.774	0.644	0.985	0.318	0.833
DB03900	2-Methyl-2-Propanol	0.768	0.313	1.039	1.857	0.963	0.82	0.568	0.761	0.946	0.809
DB08584	6-{[6-(1-methyl-1H-pyrazol-4-yl)[1,2,4]triazolo[4,3-b]pyridazin-3-yl]sulfanyl}quinoline	0.768	0.381	1.29	0.729	1.164	0.822	0.61	0.815	0.707	0.88
DB04132	S-Hexylglutathione	0.769	0.65	1.257	-0.508	-1.074	0.773	0.714	0.82	0.175	0.093
DB01694	D-tartaric acid	0.771	0.634	0.169	-0.909	0.389	0.745	0.688	0.358	0.137	0.57
DB05271	Rotigotine	0.778	1.2	2.584	0.972	-0.465	0.765	0.856	0.985	0.762	0.246
DB06854		0.778	0.701	-0.068	-0.063	0.266	0.747	0.71	0.159	0.39	0.509
DB00900	Didanosine	0.781	-0.011	2.061	-0.326	-0.124	0.82	0.46	0.97	0.278	0.371
DB08399	PICEATANNOL	0.781	1.331	0.674	-1.638	0.064	0.74	0.872	0.388	0.029	0.467
DB00317	Gefitinib	0.785	0.313	0.661	0.296	0.798	0.778	0.574	0.704	0.59	0.803
DB01942	Formic acid	0.788	-0.031	0.898	0.583	0.917	0.745	0.43	0.738	0.641	0.768
DB00696	Ergotamine	0.792	0.689	0.713	1.264	0.55	0.826	0.751	0.69	0.89	0.675
DB08846	Ellagic Acid	0.795	0.319	0.928	0.36	0.923	0.808	0.585	0.779	0.601	0.795

DB01191	Dexfenfluramine	0.797	-0.025	1.203	1.592	-0.232	0.756	0.406	0.833	0.953	0.335
DB09067	Corticorelin ovine triflutate	0.799	0.711	1.348	0.761	0.789	0.687	0.647	0.76	0.678	0.655
DB07146	2,3-DIPHENYL-N-(2-PIPERAZIN-1-YLETHYL)FURO[2,3-B]PYRIDIN-4-AMINE	0.804	1.175	0.461	0.305	-0.341	0.755	0.85	0.289	0.548	0.29
DB01109	Heparin	0.807	0.849	0.786	1.341	-0.421	0.768	0.759	0.673	0.891	0.275
DB08996	Dimetacrine	0.812	0.809	0.959	0.437	0.724	0.69	0.687	0.73	0.652	0.654
DB11598	Antithrombin III human	0.813	1.032	1.379	0.76	0.664	0.727	0.786	0.837	0.753	0.692
DB00270	Isradipine	0.815	0.596	2.195	0.853	-0.292	0.762	0.662	0.968	0.637	0.296
DB00929	Misoprostol	0.817	1.175	0.033	0.702	-0.224	0.832	0.886	0.349	0.58	0.337
DB00966	Telmisartan	0.819	0.603	2.25	1.975	0.073	0.763	0.659	0.985	0.967	0.457
DB00793	Haloprogin	0.82	0.323	1.009	1.781	-0.033	0.759	0.56	0.778	0.967	0.411
DB00918	Almotriptan	0.821	0.936	0.631	0.586	1.024	0.728	0.746	0.705	0.718	0.774
DB01124	Tolbutamide	0.823	0.476	2.177	0.597	0.034	0.85	0.689	0.966	0.671	0.459
DB08059	Wortmannin	0.825	-0.369	1.027	0.592	2.026	0.838	0.244	0.693	0.664	0.989
DB00765	Metyrosine	0.827	1.078	0.676	-0.622	0.984	0.752	0.791	0.73	0.122	0.783
DB07772	(1R)-1-{[(4'-methoxy-1,1'-biphenyl-4-yl)sulfonyl]amino}-2-methylpropylphosphonic acid	0.829	0.535	-0.321	1.251	1	0.765	0.645	0.128	0.78	0.791
DB02721	4-Iodopyrazole	0.831	1.076	1.645	0.534	0.561	0.742	0.787	0.874	0.71	0.713
DB01363	Ephedra sinica root	0.832	1.316	-0.052	0.652	-0.636	0.77	0.878	0.362	0.655	0.197
DB00703	Methazolamide	0.834	0.977	1.766	0.424	0.404	0.75	0.798	0.93	0.577	0.593
DB03608	Diminazene	0.836	0.258	1.656	1.722	0.682	0.775	0.547	0.915	0.965	0.673
DB00220	Nelfinavir	0.838	1.084	-0.31	1.073	0.357	0.836	0.882	0.324	0.827	0.587
DB00423	Methocarbamol	0.838	0.678	1.227	0.741	0.748	0.759	0.682	0.913	0.665	0.704
DB00903	Etacrynic acid	0.838	0.417	2.545	0.924	-0.144	0.759	0.614	0.996	0.758	0.362
DB09395	Sodium acetate	0.838	0.678	1.227	0.741	0.748	0.759	0.682	0.913	0.665	0.704
DB00975	Dipyridamole	0.846	1.375	-3.205	-0.818	0.725	0.779	0.905	0	0.153	0.719
DB00173	Adenine	0.847	1.402	0.845	-0.074	-0.029	0.873	0.941	0.677	0.357	0.381
DB00055	Drotrecogin alfa	0.851	0.551	0.672	0.67	1.635	0.779	0.658	0.657	0.699	0.952
DB02731	Ethylmercurithiosalicylic acid	0.853	1.332	0.518	1.135	-0.413	0.769	0.886	0.6	0.825	0.263
DB01388	Mibefradil	0.857	0.897	1.097	1.264	0.968	0.85	0.819	0.875	0.894	0.842
DB00654	Latanoprost	0.86	1.588	0.274	0.653	-0.369	0.872	0.948	0.455	0.552	0.272
DB06999	N-{3-[(5-chloro-1H-pyrrolo[2,3-b]pyridin-3-yl)carbonyl]-2,4-difluorophenyl}propane-1-sulfonamide	0.86	0.079	0.683	0.075	1.173	0.817	0.452	0.721	0.452	0.888
DB01240	Epoprostenol	0.861	0.701	0.592	0.678	0.322	0.804	0.722	0.601	0.574	0.55
DB09029	Secukinumab	0.863	0.968	0.931	0.564	0.805	0.721	0.74	0.719	0.663	0.68
DB11569	Ixekizumab	0.863	0.968	0.931	0.564	0.805	0.721	0.74	0.719	0.663	0.68
DB08865	Crizotinib	0.869	0.241	0.256	0.577	0.439	0.812	0.534	0.578	0.739	0.655
DB07326	6-chloro-N-pyrimidin-5-yl-3-{[3-(trifluoromethyl)phenyl]amino}-1,2-benzisoxazole-7-carboxamide	0.87	1.143	-0.475	0.535	0.1	0.865	0.892	0.175	0.635	0.449
DB00738	Pentamidine	0.874	0.481	0.8	1.355	1.736	0.841	0.666	0.718	0.91	0.966
DB04876	Vildagliptin	0.877	1.098	-2.319	0.699	0.713	0.803	0.843	0.004	0.523	0.681
DB04884	Dapoxetine	0.877	1.09	1.157	-0.49	0.788	0.72	0.775	0.779	0.417	0.697
DB00297	Bupivacaine	0.879	1.423	-1.914	0.061	0.716	0.785	0.903	0.019	0.383	0.682
DB00969	Alosetron	0.881	1.316	0.298	1.081	-0.498	0.779	0.878	0.495	0.777	0.235
DB00044	Lutropin alfa	0.882	1.088	0.45	-0.325	0.909	0.726	0.796	0.597	0.48	0.713
DB09126	Chorionic Gonadotropin (Human)	0.882	1.088	0.45	-0.325	0.909	0.726	0.796	0.597	0.48	0.713
DB09283	Trapidil	0.882	1.105	-0.186	-0.204	0.231	0.779	0.833	0.343	0.32	0.494
DB00718	Adefovir dipivoxil	0.888	1.299	0.933	0.164	0.623	0.798	0.876	0.726	0.406	0.67
DB08954	Ifenprodil	0.89	0.694	0.236	1.049	0.668	0.779	0.69	0.416	0.697	0.659
DB00818	Propofol	0.891	0.348	2.583	0.112	0.095	0.801	0.587	0.994	0.452	0.458
DB01095	Fluvastatin	0.892	1.339	1.714	1.019	-0.675	0.773	0.879	0.909	0.664	0.178
DB00007	Leuprolide	0.894	0.918	1.405	0.979	0.556	0.856	0.864	0.96	0.788	0.63
DB00106	Abarelix	0.894	0.918	1.405	0.979	0.556	0.856	0.864	0.96	0.788	0.63
DB00594	Amiloride	0.894	-0.217	1.823	2.005	2.028	0.851	0.312	0.958	0.973	0.993

DB00644	Gonadorelin	0.894	0.918	1.405	0.979	0.556	0.856	0.864	0.96	0.788	0.63
DB00666	Nafarelin	0.894	0.918	1.405	0.979	0.556	0.856	0.864	0.96	0.788	0.63
DB03496	Alvocidib	0.894	0.498	0.059	0.095	0.181	0.811	0.666	0.464	0.464	0.52
DB06699	Degarelix	0.894	0.918	1.405	0.979	0.556	0.856	0.864	0.96	0.788	0.63
DB06785	Ganirelix	0.894	0.918	1.405	0.979	0.556	0.856	0.864	0.96	0.788	0.63
DB06788	Histrelin	0.894	0.918	1.405	0.979	0.556	0.856	0.864	0.96	0.788	0.63
DB06825	Triptorelin	0.894	0.918	1.405	0.979	0.556	0.856	0.864	0.96	0.788	0.63
DB00429	Carboprost tromethamine	0.895	0.91	0.349	1.086	0.652	0.789	0.803	0.488	0.809	0.625
DB06891	5-{[(4-AMINO-3-CHLORO-5-FLUOROPHENYL)SULFONYL]AMINO}-1,3,4-THIADIAZOLE-2-SULFONAMIDE	0.896	1.375	1.331	-0.181	-0.127	0.786	0.902	0.841	0.318	0.338
DB06795	Mafenide	0.897	1.438	1.218	-0.602	-0.511	0.781	0.896	0.825	0.168	0.227
DB06954	2-(cycloheptylmethyl)-1,1-dioxido-1-benzothiophen-6-yl sulfamate	0.897	1.438	1.218	-0.602	-0.511	0.781	0.896	0.825	0.168	0.227
DB08083	2-(1,3-thiazol-4-yl)-1H-benzimidazole-5-sulfonamide	0.897	1.438	1.218	-0.602	-0.511	0.781	0.896	0.825	0.168	0.227
DB00239	Oxiconazole	0.898	1.008	0.291	1.43	-1.018	0.779	0.811	0.534	0.928	0.114
DB00496	Darifenacin	0.898	1.473	-0.558	1.297	-0.594	0.772	0.901	0.191	0.868	0.228
DB01117	Atovaquone	0.899	1.623	-0.594	-0.217	0.568	0.803	0.933	0.086	0.263	0.629
DB07926	N-[3-(N'-HYDROXYCARBOXAMIDO)-2-(2-METHYLPROPYL)-PROPANOYL]-O-TYROSINE-N-METHYLAMIDE	0.902	0.506	-0.161	1.156	1.122	0.782	0.642	0.142	0.737	0.814
DB08507	N-[[2-METHYL-4-HYDROXYCARBAMOYL]BUT-4-YL-N]-BENZYL-P-[PHENYL]-P-[METHYL]PHOSPHINAMID	0.902	0.506	-0.161	1.156	1.122	0.782	0.642	0.142	0.737	0.814
DB00187	Esmolol	0.903	1.146	1.157	-0.514	0.791	0.744	0.805	0.787	0.386	0.683
DB07159	Tamatinib	0.907	-0.319	0.725	0.42	0.884	0.846	0.235	0.749	0.657	0.91
DB01446	Indopan	0.908	0.023	1.22	1.573	0.111	0.794	0.442	0.831	0.939	0.461
DB08943	Isoconazole	0.908	1.296	-0.307	1.222	0.735	0.833	0.887	0.141	0.776	0.703
DB00025	Antihemophilic factor, human recombinant	0.914	1.266	0.673	1.198	-0.488	0.837	0.898	0.579	0.851	0.232
DB00982	Isotretinoin	0.915	0.845	1.484	1.066	0.266	0.876	0.81	0.908	0.824	0.504
DB09034	Suvorexant	0.917	1.234	0.1	-0.332	0.829	0.734	0.853	0.525	0.499	0.694
DB01156	Bupropion	0.918	1.267	0.401	0.669	-0.288	0.792	0.876	0.546	0.625	0.298
DB04572	Thiotepa	0.918	1.344	-0.583	0.877	0.245	0.847	0.913	0.127	0.697	0.531
DB00287	Travoprost	0.922	1.262	0.551	0.572	-0.029	0.891	0.923	0.58	0.501	0.426
DB05379		0.922	1.262	0.551	0.572	-0.029	0.891	0.923	0.58	0.501	0.426
DB08877	Ruxolitinib	0.922	0.385	1.288	-0.042	0.102	0.832	0.603	0.873	0.373	0.489
DB00226	Guanadrel	0.924	0.938	0.608	0.608	0.544	0.752	0.75	0.701	0.724	0.684
DB00629	Guanabenz	0.924	0.458	1.517	0.894	-0.282	0.799	0.627	0.883	0.661	0.289
DB01088	Iloprost	0.926	0.502	0.109	0.5	1.961	0.794	0.65	0.432	0.607	0.979
DB00063	Eptifibatide	0.927	1.216	0.603	0.067	0.818	0.808	0.837	0.716	0.54	0.807
DB00161	L-Valine	0.93	1.195	1.805	0.758	-0.108	0.871	0.905	0.907	0.56	0.386
DB00579	Mazindol	0.933	1.295	0.21	0.154	-0.504	0.86	0.924	0.46	0.318	0.217
DB06016	Cariprazine	0.935	0.233	0.635	1.209	-0.078	0.809	0.514	0.613	0.799	0.385
DB06637	Dalfampridine	0.938	0.991	0.158	1.089	1.751	0.799	0.808	0.452	0.789	0.962
DB00403	Ceruletide	0.939	0.918	1.405	0.979	0.768	0.896	0.864	0.96	0.788	0.807
DB04856	Dexloxiglumide	0.939	0.918	1.405	0.979	0.768	0.896	0.864	0.96	0.788	0.807
DB08765	6-HYDROXY-1,3-BENZOTHIAZOLE-2-SULFONAMIDE	0.94	1.37	0.87	-0.034	-0.51	0.816	0.894	0.7	0.382	0.232
DB00561	Doxapram	0.943	1.305	1.354	-0.233	0.362	0.815	0.847	0.869	0.388	0.779
DB00163	Vitamin E	0.947	0.903	-0.198	0.96	1.275	0.84	0.814	0.307	0.782	0.902
DB00474	Methohexital	0.947	0.73	1.812	1.113	0.938	0.742	0.671	0.881	0.788	0.729
DB08965	Fusafungine	0.949	0.972	1.3	-0.348	1.284	0.892	0.854	0.913	0.267	0.912
DB01021	Trichlormethiazide	0.95	1.58	1.517	-0.054	-0.851	0.791	0.927	0.889	0.372	0.146
DB01303	Oxtriphylline	0.954	1.303	0.183	0.031	0.724	0.878	0.964	0.34	0.375	0.749
DB01073	Fludarabine	0.955	1.757	-1.156	-0.704	1.563	0.889	0.973	0.083	0.195	0.952
DB00213	Pantoprazole	0.959	1.063	2.345	-0.697	0.562	0.807	0.812	0.978	0.191	0.636

DB06643	Denosumab	0.96	1.089	0.314	-0.22	0.904	0.813	0.808	0.655	0.429	0.802
DB01445	Bufotenine	0.963	0.601	1.234	0.715	-0.115	0.86	0.685	0.795	0.532	0.378
DB00310	Chlorthalidone	0.965	1.368	1.42	-0.635	-0.479	0.808	0.895	0.873	0.168	0.226
DB00166	Lipoic Acid	0.967	1.007	-0.597	0.687	0.258	0.806	0.813	0.2	0.705	0.522
DB01161	Chloroprocaine	0.967	1.41	0.552	0.296	-0.114	0.907	0.94	0.589	0.439	0.382
DB00197	Troglitazone	0.969	0.307	0.389	0.802	-0.328	0.852	0.572	0.546	0.736	0.302
DB02078	Triglyme	0.969	0.744	0.424	1.1	0.881	0.877	0.759	0.398	0.812	0.824
DB04137	Guanosine-5'-Triphosphate	0.969	0.639	-0.01	-0.471	0.173	0.81	0.695	0.269	0.245	0.502
DB00330	Ethambutol	0.972	0.671	0.758	0.709	-0.488	0.845	0.724	0.69	0.542	0.238
DB00541	Vincristine	0.974	0.838	0.554	0.661	1.21	0.92	0.829	0.481	0.681	0.903
DB00021		0.976	1.087	0.215	0.818	1.135	0.774	0.821	0.51	0.705	0.839
DB04769	5-QUINOXALIN-6-YLMETHYLENE-THIAZOLIDINE-2,4-DIONE	0.976	0.735	0.817	1.091	-0.177	0.808	0.716	0.663	0.684	0.33
DB00273	Topiramate	0.977	0.45	1.856	0.386	0.188	0.813	0.617	0.948	0.586	0.505
DB00457	Prazosin	0.983	0.331	1.809	1.595	0.578	0.816	0.559	0.95	0.955	0.664
DB09038	Empagliflozin	0.983	1.303	1.743	0.97	0.844	0.754	0.851	0.881	0.758	0.706
DB00419	Miglustat	0.984	2.147	0.957	0.335	-2.243	0.813	0.981	0.729	0.456	0.011
DB00709	Lamivudine	0.984	0.641	1.926	0.171	-0.266	0.863	0.74	0.955	0.428	0.317
DB04829	Lysergic acid diethylamide	0.984	0.404	0.84	1.799	-0.17	0.806	0.608	0.721	0.952	0.366
DB06155	Rimonabant	0.984	1.57	1.423	-0.082	-0.452	0.806	0.925	0.872	0.336	0.235
DB01444	Dimethylthiambutene	0.985	1.629	-0.304	0.803	0.21	0.918	0.949	0.083	0.586	0.551
DB07194	2-{2-[(3,5-dimethylphenyl)amino]pyrimidin-4-yl}-N-[(1S)-2-hydroxy-1-methylethyl]-4-methyl-1,3-thiazole-5-carboxamide	0.985	0.944	0.227	1.571	0.115	0.809	0.802	0.207	0.858	0.475
DB08213	1-METHYL-5-(2-PHENOXYMETHYL-PYRROLIDINE-1-SULFONYL)-1H-INDOLE-2,3-DIONE	0.985	1.247	1.224	-0.023	-0.316	0.809	0.88	0.839	0.399	0.309
DB00940	Methantheline	0.995	1.378	0.765	-0.504	0.957	0.773	0.902	0.66	0.415	0.751
DB01223	Aminophylline	0.995	1.442	0.056	0.08	0.859	0.907	0.974	0.327	0.407	0.788
DB07794	5-(2-PHENYLPYRAZOLO[1,5-A]PYRIDIN-3-YL)-1H-PYRAZOLO[3,4-C]PYRIDAZIN-3-AMINE	0.996	0.751	1.211	1.076	0.638	0.911	0.757	0.708	0.827	0.733
DB08403	METHYLAMINO-PHENYLALANYL-LEUCYL-HYDROXAMIC ACID	1	0.713	0.556	1.15	1.169	0.813	0.711	0.552	0.732	0.83
DB00642	Pemetrexed	1.002	0.447	-0.663	0.828	-0.117	0.817	0.617	0.304	0.712	0.371
DB01238	Aripiprazole	1.002	0.276	0.652	1.63	0.241	0.83	0.557	0.67	0.954	0.516
DB01326	Cefamandole	1.004	1.314	0.881	-0.209	1.522	0.895	0.904	0.725	0.322	0.949
DB03991	2-Deoxy-2,3-Dehydro-N-Acetyl-Neuraminic Acid	1.004	1.346	0.896	0.296	0.86	0.766	0.863	0.748	0.657	0.72
DB07264	(S)-N-(1-(3-CHLORO-4-FLUOROPHENYL)-2-HYDROXYETHYL)-4-(4-(3-CHLOROPHENYL)-1H-PYRAZOL-3-YL)-1H-PYRROLE-2-CARBOXAMIDE	1.004	1.153	1.027	-0.311	0.681	0.902	0.922	0.653	0.309	0.738
DB01045	Rifampicin	1.005	0.631	-0.041	0.132	-0.963	0.819	0.688	0.311	0.468	0.13
DB02258	SR11254	1.005	2.519	1.131	-0.123	-0.735	0.816	0.988	0.794	0.349	0.171
DB02741	CD564	1.005	2.519	1.131	-0.123	-0.735	0.816	0.988	0.794	0.349	0.171
DB02877	Arotinoid acid	1.005	2.519	1.131	-0.123	-0.735	0.816	0.988	0.794	0.349	0.171
DB04942	Tamibarotene	1.005	2.519	1.131	-0.123	-0.735	0.816	0.988	0.794	0.349	0.171
DB01373	Calcium	1.006	0.241	0.739	2.273	1.337	0.861	0.521	0.626	0.994	0.959
DB00188	Bortezomib	1.007	1.345	0.168	1.79	0.122	0.874	0.901	0.45	0.95	0.479
DB00677	Isoflurophate	1.007	1.082	0.758	0.994	-0.87	0.833	0.824	0.644	0.654	0.14
DB00679	Thioridazine	1.008	1.107	2.057	2.752	0.252	0.884	0.889	0.974	1	0.554
DB02665	(1R,2S)-2-Phenylcyclopropanaminium	1.012	0.873	0.696	1.813	1.195	0.934	0.826	0.622	0.964	0.908
DB01091	Butenafine	1.013	1.386	1.019	0.311	-0.578	0.817	0.906	0.763	0.365	0.193
DB09140	Oxygen	1.014	2.043	2.292	0.376	-0.579	0.817	0.977	0.965	0.564	0.197

DB01018	Guanfacine	1.017	0.589	2.116	0.201	0.163	0.813	0.67	0.954	0.423	0.463
DB02772	Sucrose	1.017	0.765	0.758	1.257	0.598	0.831	0.766	0.659	0.774	0.663
DB00313	Valproic Acid	1.024	0.476	2.236	2.689	0.889	0.897	0.655	0.981	0.995	0.806
DB03260	1,6-Diaminohexane	1.024	1.652	1.035	0.297	-0.368	0.826	0.93	0.77	0.434	0.264
DB04665	Coumarin	1.024	1.652	1.035	0.297	-0.368	0.826	0.93	0.77	0.434	0.264
DB03902	Oxalic Acid	1.028	0.967	1.287	1.642	0.877	0.806	0.786	0.748	0.953	0.719
DB06715	Potassium Iodide	1.028	0.967	1.287	1.642	0.877	0.806	0.786	0.748	0.953	0.719
DB06794	Lodoxamide	1.028	1.065	1.287	0.383	0.957	0.806	0.821	0.748	0.524	0.778
DB09154	Sodium citrate	1.028	0.967	1.287	1.642	0.877	0.806	0.786	0.748	0.953	0.719
DB00862	Vardenafil	1.03	1.376	0.766	1.315	-1.321	0.813	0.892	0.697	0.877	0.077
DB00130	L-Glutamine	1.034	1.255	1.889	-0.454	0.295	0.861	0.901	0.938	0.184	0.54
DB00123	L-Lysine	1.038	1.288	1.843	0.505	0.407	0.856	0.879	0.924	0.625	0.566
DB00358	Mefloquine	1.039	1.04	0.36	1.162	0.808	0.906	0.865	0.483	0.841	0.767
DB03619	Deoxycholic Acid	1.041	1.555	1.365	-0.36	-1.276	0.844	0.935	0.835	0.205	0.067
DB00721	Procaine	1.042	0.628	-0.014	0.57	0.832	0.825	0.689	0.398	0.624	0.733
DB03854	Pentane-1,5-Diamine	1.047	1.587	0.761	0.049	-0.517	0.88	0.958	0.817	0.363	0.241
DB00905	Bimatoprost	1.056	1.589	-0.704	0.817	-0.083	0.871	0.939	0.076	0.602	0.382
DB00685	Trovafloxacin	1.059	0.809	0.14	0.622	1.687	0.92	0.803	0.446	0.671	0.966
DB08922	Perospirone	1.061	0.324	2.456	0.652	-0.807	0.825	0.566	0.977	0.522	0.154
DB08700		1.062	0.266	-0.3	0.47	0.577	0.883	0.557	0.333	0.677	0.725
DB02530	gamma-Aminobutyric acid	1.063	0.455	2.765	1.089	0.518	0.821	0.635	0.995	0.789	0.621
DB07213	(5-{3-[5-(PIPERIDIN-1-YLMETHYL)-1H-INDOL-2-YL]-1H-INDAZOL-6-YL}-2H-1,2,3-TRIAZOL-4-YL)METHANOL	1.066	1.474	1.264	-0.872	0.529	0.821	0.906	0.859	0.127	0.631
DB00258	Calcium acetate	1.07	1.308	0.667	0.945	0.324	0.938	0.939	0.399	0.783	0.561
DB03166	Acetic acid	1.07	1.308	0.667	0.945	0.324	0.938	0.939	0.399	0.783	0.561
DB08927	Amperozide	1.073	1.425	0.903	-0.175	0.949	0.84	0.874	0.806	0.394	0.846
DB00692	Phentolamine	1.078	0.154	1.924	0.501	0.226	0.832	0.495	0.952	0.576	0.501
DB02212		1.097	0.053	0.256	1.105	0.87	0.843	0.46	0.284	0.787	0.758
DB00199	Erythromycin	1.103	0.317	1.979	1.099	0.371	0.837	0.574	0.959	0.776	0.579
DB01127	Econazole	1.103	0.926	0.42	2.061	0.958	0.898	0.835	0.628	0.995	0.833
DB00190	Carbidopa	1.104	1.255	-0.07	0.657	0.201	0.94	0.921	0.313	0.708	0.511
DB01224	Quetiapine	1.104	0.146	0.979	2.646	0.324	0.853	0.501	0.797	0.998	0.577
DB07780	Farnesyl diphosphate	1.105	1.951	1.09	1.531	-0.758	0.828	0.966	0.807	0.877	0.163
DB09570	Ixazomib	1.114	0.903	2.259	0.83	-1.277	0.844	0.781	0.993	0.662	0.072
DB00617	Paramethadione	1.116	1.003	1.854	1.086	0.955	0.902	0.843	0.991	0.809	0.812
DB00298	Dapiprazole	1.12	0.324	1.863	0.86	-0.512	0.842	0.54	0.933	0.634	0.231
DB01301	Rolitetracycline	1.121	1.187	0.401	0.975	0.605	0.855	0.856	0.322	0.788	0.659
DB01262	Decitabine	1.122	1.734	-0.907	-0.28	0.777	0.852	0.938	0.135	0.287	0.73
DB01135	Doxacurium	1.124	1.434	0.379	0.075	1.231	0.79	0.878	0.64	0.597	0.813
DB01336	Metocurine	1.124	1.434	0.379	0.075	1.231	0.79	0.878	0.64	0.597	0.813
DB02292	Irosustat	1.124	1.396	1.785	0.089	-0.195	0.846	0.89	0.929	0.424	0.327
DB04129	Willardiine	1.126	1.543	1.356	0.286	-0.029	0.928	0.948	0.845	0.345	0.431
DB00376	Trihexyphenidyl	1.134	1.42	0.041	0.969	-0.474	0.845	0.894	0.381	0.747	0.252
DB00502	Haloperidol	1.137	0.072	1.377	3.482	0.82	0.902	0.464	0.9	1	0.774
DB04604	5-iodotubercidin	1.142	1.375	0.914	0.374	0.779	0.94	0.941	0.584	0.579	0.773
DB01023	Felodipine	1.143	1.169	1.611	2.602	-0.606	0.907	0.886	0.924	0.998	0.187
DB00720	Clodronic acid	1.147	1.298	1.299	0.438	0.39	0.854	0.866	0.889	0.599	0.594
DB00177	Valsartan	1.148	1.282	1.093	-0.097	1.227	0.845	0.85	0.825	0.46	0.84
DB00275	Olmesartan	1.148	1.282	1.093	-0.097	1.227	0.845	0.85	0.825	0.46	0.84
DB00364	Sucralfate	1.148	0.259	1.956	1.91	1.907	0.85	0.528	0.958	0.988	0.966
DB00876	Eprosartan	1.148	1.282	1.093	-0.097	1.227	0.845	0.85	0.825	0.46	0.84
DB08822	Azilsartan medoxomil	1.148	1.282	1.093	-0.097	1.227	0.845	0.85	0.825	0.46	0.84
DB09279	Fimasartan	1.148	1.282	1.093	-0.097	1.227	0.845	0.85	0.825	0.46	0.84
DB01180	Rescinnamine	1.149	1.428	-0.395	1.206	0.625	0.886	0.916	0.128	0.747	0.673
DB11560	Lesinurad	1.149	1.237	1.029	0.425	1.113	0.81	0.845	0.748	0.636	0.771
DB00575	Clonidine	1.152	0.775	2.887	0.892	-0.441	0.848	0.732	0.994	0.754	0.228
DB01007	Tioconazole	1.153	0.971	1.157	1.3	1.251	0.817	0.755	0.787	0.829	0.84
DB00121	Biotin	1.154	1.665	0.589	-1.006	1.299	0.933	0.965	0.564	0.119	0.906

DB00993	Azathioprine	1.155	1.096	-2.642	0.048	1.716	0.86	0.842	0.005	0.427	0.948
DB01267	Paliperidone	1.155	0.446	2.048	2.517	0.373	0.866	0.622	0.967	0.995	0.587
DB00184	Nicotine	1.157	2.195	-0.317	0.667	0.928	0.844	0.985	0.277	0.659	0.773
DB09235	Efonidipine	1.157	1.105	2.01	0.564	1.01	0.812	0.798	0.992	0.614	0.743
DB06216	Asenapine	1.158	0.45	1.751	2.477	-0.086	0.86	0.612	0.937	0.996	0.396
DB00530	Erlotinib	1.161	0.149	-0.245	0.483	0.301	0.895	0.489	0.364	0.663	0.559
DB08558	2-HYDROXYMETHYL-6-OCTYLSULFANYL-TETRAHYDRO-PYRAN-3,4,5-TRIOL	1.161	1.403	1.722	-0.239	0.835	0.818	0.853	0.893	0.514	0.747
DB00183	Pentagastrin	1.162	1.128	1.13	0.708	1.063	0.787	0.799	0.794	0.736	0.761
DB03389	alpha-D-Xylopyranose	1.162	1.452	1.747	0.977	0.535	0.824	0.907	0.864	0.748	0.608
DB09142	Sincalide	1.162	1.128	1.13	0.708	1.063	0.787	0.799	0.794	0.736	0.761
DB04419	D-norleucine	1.164	0.998	1.692	1.046	1.421	0.822	0.763	0.86	0.772	0.891
DB00931	Metacycline	1.167	1.124	-1.758	1.028	-0.038	0.858	0.855	0.033	0.817	0.447
DB00257	Clotrimazole	1.172	1.242	-0.702	1.492	0.099	0.901	0.937	0.201	0.97	0.483
DB00540	Nortriptyline	1.173	1.086	0.938	2.288	-0.626	0.869	0.856	0.781	0.993	0.207
DB08341	4-{[4-{[(1R,2R)-2-(dimethylamino)cyclopentyl]amino}-5-(trifluoromethyl)pyrimidin-2-yl]amino}-N-methylbenzenesulfonamide	1.175	1.624	0.275	-0.845	1.399	0.862	0.931	0.232	0.135	0.877
DB00476	Duloxetine	1.179	0.38	-0.033	1.937	0.906	0.87	0.612	0.39	0.986	0.779
DB09036	Siltuximab	1.18	1.114	1.677	1.282	1.117	0.893	0.845	0.978	0.883	0.857
DB01261	Sitagliptin	1.181	1.325	-2.35	0.787	0.832	0.856	0.887	0.002	0.563	0.734
DB00585	Nizatidine	1.182	1.27	1.409	0.991	1.025	0.801	0.824	0.844	0.796	0.763
DB00875	Flupentixol	1.19	1.086	2.121	1.436	-0.642	0.87	0.815	0.961	0.932	0.184
DB00527	Cinchocaine	1.191	0.91	0.911	1.212	1.218	0.939	0.816	0.667	0.846	0.883
DB03023	1-Tert-Butyl-3-(4-Chloro-Phenyl)-1h-Pyrazolo[3,4-D]Pyrimidin-4-Ylamine	1.195	1.353	0.927	1.297	0.529	0.955	0.913	0.552	0.879	0.655
DB07384	1-ACETYL-2-CARBOXYPIPERIDINE	1.197	1.647	0.456	-1.201	-0.096	0.863	0.937	0.275	0.063	0.387
DB07394	AUROVERTIN B	1.197	1.647	0.456	-1.201	-0.096	0.863	0.937	0.275	0.063	0.387
DB08629	N1-(2-AMINO-4-METHYLPENTYL)OCTAHYDRO-PYRROLO[1,2-A] PYRIMIDINE	1.197	1.647	0.456	-1.201	-0.096	0.863	0.937	0.275	0.063	0.387
DB00363	Clozapine	1.198	0.289	1.507	2.648	0.332	0.869	0.575	0.907	0.999	0.583
DB00751	Epinastine	1.198	1.17	2.348	1.374	-0.5	0.858	0.856	0.976	0.88	0.227
DB00778	Roxithromycin	1.212	1.242	1.227	1.248	1.051	0.953	0.955	0.913	0.872	0.906
DB01396	Digitoxin	1.216	1.391	-0.452	-0.129	1.837	0.953	0.944	0.245	0.377	0.976
DB00045	Lyme disease vaccine (recombinant OspA)	1.231	0.974	-0.306	2.37	1.005	0.841	0.791	0.331	0.949	0.801
DB11601	Tuberculin Purified Protein Derivative	1.231	0.974	-0.306	2.37	1.005	0.841	0.791	0.331	0.949	0.801
DB01992	Coenzyme A	1.236	0.467	0.708	0.341	0.242	0.876	0.624	0.619	0.535	0.516
DB05424	Canertinib	1.24	0.48	1.519	0.378	0.784	0.909	0.655	0.913	0.602	0.781
DB00752	Tranylcypromine	1.254	0.89	0.245	1.945	1.683	0.985	0.824	0.462	0.972	0.975
DB05013	Ingenol mebutate	1.255	0.938	-0.234	-0.079	1.353	0.937	0.848	0.239	0.398	0.926
DB00934	Maprotiline	1.257	0.285	1.9	2.089	0.119	0.881	0.566	0.961	0.988	0.47
DB11582	Thiocolchicoside	1.257	0.328	1.784	0.789	0.714	0.859	0.571	0.942	0.717	0.711
DB01698	Rutin	1.261	2.002	1.767	-0.825	-0.27	0.869	0.985	0.975	0.152	0.337
DB03685	Uridine monophosphate	1.264	1.656	0.155	0.567	-0.314	0.876	0.941	0.41	0.455	0.283
DB08960	Hexamethonium	1.264	0.834	1.64	1.336	1.278	0.846	0.707	0.854	0.844	0.85
DB00637	Astemizole	1.265	1.672	1.883	2.279	0.699	0.933	0.978	0.955	0.991	0.732
DB00948	Mezlocillin	1.268	1.261	0.14	0.231	-0.505	0.883	0.884	0.475	0.516	0.239
DB00149	L-Leucine	1.27	1.402	1.24	0.873	-0.598	0.872	0.901	0.832	0.606	0.203
DB08175	(2E,4E)-11-METHOXY-3,7,11-TRIMETHYLDODECA-2,4-DIENOIC ACID	1.27	1.362	1.1	1.177	-0.091	0.876	0.887	0.798	0.739	0.365
DB00168	Aspartame	1.273	1.52	1.231	1.071	0.878	0.835	0.882	0.857	0.801	0.746

DB01623	Thiothixene	1.277	0.336	2.525	2.482	0.024	0.896	0.569	0.991	0.998	0.456
DB13154	Parachlorophenol	1.279	1.148	0.578	1.863	1.292	0.893	0.853	0.596	0.959	0.918
DB00485	Dicloxacillin	1.28	1.977	-1.071	0.906	-0.791	0.878	0.98	0.071	0.717	0.162
DB00724	Imiquimod	1.28	2.413	-1.207	1.581	0.054	0.918	0.987	0.04	0.942	0.447
DB02255	GM6001	1.281	0.956	-1.09	1.373	1.068	0.882	0.793	0.089	0.888	0.824
DB00988	Dopamine	1.282	0.804	0.704	1.082	0.136	0.891	0.78	0.799	0.856	0.489
DB00246	Ziprasidone	1.284	0.479	0.742	2.351	0.112	0.899	0.655	0.704	0.996	0.469
DB06292	Dapagliflozin	1.288	1.526	2.122	1.455	0.986	0.833	0.9	0.93	0.871	0.753
DB00849	Methylphenobarbital	1.292	0.839	2.74	0.389	-0.331	0.881	0.757	0.989	0.513	0.274
DB00434	Cyproheptadine	1.293	0.312	2.299	2.989	1.071	0.942	0.576	0.986	1	0.854
DB00780	Phenelzine	1.295	1.62	-0.365	1.777	0.508	0.972	0.977	0.271	0.97	0.632
DB00610	Metaraminol	1.296	1.302	1.348	1.492	1.11	0.912	0.892	0.76	0.921	0.83
DB00699	Nicergoline	1.296	1.302	1.348	1.492	1.11	0.912	0.892	0.76	0.921	0.83
DB06764	Tetryzoline	1.296	1.302	1.348	1.492	1.11	0.912	0.892	0.76	0.921	0.83
DB00115	Cyanocobalamin	1.3	1.23	0.642	0.709	0.078	0.89	0.866	0.634	0.645	0.416
DB09238	Manidipine	1.304	1.043	3.026	0.314	-0.254	0.884	0.824	0.995	0.465	0.305
DB01488	Dimethyltryptamine	1.305	0.669	1.177	0.863	-0.313	0.878	0.695	0.812	0.611	0.29
DB07954	3-isobutyl-1-methyl-7H-xanthine	1.305	1.953	0.359	-0.204	0.364	0.885	0.967	0.528	0.257	0.541
DB11595	Atezolizumab	1.305	1.031	1.987	1.972	0.873	0.844	0.809	0.91	0.922	0.781
DB01941	6-[1-(3,5,5,8,8-Pentamethyl-5,6,7,8-Tetrahydronaphthalen-2-Yl)Cyclopropyl]Pyridine-3-Carboxylic Acid	1.309	2.453	1.407	0.076	0.132	0.893	0.989	0.95	0.449	0.513
DB08835	Spaglumic Acid	1.309	1.1	0.574	1.157	1.482	0.861	0.787	0.638	0.813	0.905
DB00523	Alitretinoin	1.312	1.55	1.548	0.204	-0.228	0.881	0.922	0.929	0.51	0.329
DB00604	Cisapride	1.316	0.106	1.277	1.525	0.561	0.882	0.483	0.852	0.924	0.644
DB01231	Diphenidol	1.317	1.042	1.403	1.198	-0.618	0.883	0.811	0.87	0.826	0.209
DB01537	4-Bromo-2,5-dimethoxyphenethylamine	1.317	1.271	1.067	1.454	1.117	0.887	0.852	0.754	0.871	0.784
DB03793	Benzoic Acid	1.323	1.176	1.207	0.968	0.427	0.884	0.848	0.817	0.672	0.561
DB04745	2-OXOQUINOLINE	1.323	1.786	1.678	0.583	0.032	0.889	0.958	0.919	0.583	0.41
DB00395	Carisoprodol	1.324	2.156	-0.676	0.684	0.711	0.929	0.972	0.078	0.537	0.683
DB01618	Molindone	1.328	0.506	3.035	1.38	-0.457	0.896	0.636	0.996	0.89	0.26
DB00833	Cefaclor	1.329	0.816	2.196	1.331	0.793	0.894	0.77	0.986	0.792	0.717
DB04908	Flibanserin	1.332	0.461	2.457	0.937	0.052	0.885	0.634	0.977	0.657	0.443
DB00464	Sodium Tetradecyl Sulfate	1.336	1.693	3.445	-0.848	0.788	0.864	0.897	0.98	0.084	0.832
DB08490	CTS-1027	1.337	0.72	-0.243	1.311	1.233	0.898	0.709	0.173	0.824	0.862
DB06150	Sulfadimethoxine	1.339	1.533	1.681	0.216	1.095	0.824	0.857	0.882	0.715	0.803
DB01216	Finasteride	1.352	1.948	1.759	0.522	0.59	0.906	0.932	0.926	0.696	0.833
DB00407	Ardeparin	1.354	1.522	1.359	0.834	1.174	0.856	0.869	0.859	0.82	0.824
DB06271	Sulodexide	1.354	1.522	1.359	0.834	1.174	0.856	0.869	0.859	0.82	0.824
DB01429	Aprindine	1.357	0.861	0.612	2.433	0.984	0.974	0.806	0.58	0.989	0.829
DB02098	Adenosine-2'-5'-Diphosphate	1.357	1.952	2.055	0.189	0.46	0.851	0.912	0.925	0.67	0.807
DB04825	Prenylamine	1.364	1.014	0.571	2.014	1.282	0.977	0.827	0.416	0.97	0.904
DB08271	N-ISOBUTYL-N-[4-METHOXYPHENYLSULFONYL]GLYCYL HYDROXAMIC ACID	1.364	0.948	1.192	1.981	1.781	0.887	0.786	0.813	0.986	0.962
DB00228	Enflurane	1.369	0.497	0.937	1.059	0.203	0.903	0.643	0.837	0.91	0.477
DB00656	Trazodone	1.369	0.578	2.1	1.848	0.516	0.909	0.666	0.971	0.979	0.628
DB00043	Omalizumab	1.373	1.381	1.556	0.852	1.674	0.858	0.851	0.888	0.816	0.896
DB00641	Simvastatin	1.376	1.181	0.462	1.264	0.169	0.89	0.85	0.531	0.778	0.467
DB00623	Fluphenazine	1.377	1.528	0.575	2.652	1.339	0.96	0.967	0.754	0.996	0.924
DB00772	Malathion	1.38	1.663	1.095	0.32	-0.073	0.907	0.933	0.79	0.487	0.383
DB01625	Isopropamide	1.38	1.448	0.491	0.861	-0.246	0.91	0.916	0.521	0.605	0.333
DB00599	Thiopental	1.381	1.017	2.24	0.557	-0.775	0.906	0.821	0.978	0.595	0.16
DB00114	Pyridoxal phosphate	1.387	0.892	-0.06	1.513	1.604	0.961	0.809	0.407	0.957	0.953
DB09053	Ibrutinib	1.4	1.038	1.972	1.28	0.538	0.954	0.865	0.953	0.888	0.68
DB02999	Quisqualate	1.405	1.34	0.771	0.617	0.312	0.899	0.89	0.679	0.505	0.505
DB00669	Sumatriptan	1.406	0.181	2.052	1.914	0.899	0.899	0.516	0.966	0.973	0.762

DB00915	Amantadine	1.406	1.179	3.235	1.216	0.674	0.912	0.864	0.994	0.77	0.674
DB00935	Oxymetazoline	1.41	0.606	1.659	1.538	0.617	0.903	0.684	0.922	0.936	0.654
DB02329	Carbenoxolone	1.413	1.512	1.069	1.412	0.841	0.839	0.863	0.833	0.867	0.777
DB09372	Tannic acid	1.421	0.917	1.673	0.908	0.73	0.981	0.835	0.926	0.768	0.751
DB07636	5-HEPTYL-6-HYDROXY-1,3-BENZOTHIAZOLE-4,7-DIONE	1.426	1.675	2.077	0.934	-0.636	0.908	0.946	0.946	0.629	0.19
DB00602	Ivermectin	1.433	1.755	0.487	1.181	-0.56	0.904	0.954	0.607	0.838	0.21
DB01227	Levacetylmethadol	1.434	1.874	0.485	1.005	0.292	0.926	0.96	0.514	0.665	0.538
DB06290	Simeprevir	1.437	1.401	1.747	1.309	1.372	0.945	0.939	0.979	0.858	0.94
DB03128	Acetylcholine	1.445	1.595	-0.056	0.808	-0.573	0.914	0.934	0.359	0.689	0.215
DB01016	Glyburide	1.448	1.446	1.945	2.319	-0.152	0.906	0.914	0.959	0.999	0.358
DB00370	Mirtazapine	1.452	0.717	0.677	1.672	-0.33	0.929	0.72	0.689	0.956	0.298
DB01008	Busulfan	1.452	1.675	1.208	0.008	1.549	0.884	0.897	0.846	0.542	0.884
DB08887	Icosapent ethyl	1.453	1.45	1.732	1.858	1.27	0.968	0.956	0.98	0.974	0.924
DB06772	Cabazitaxel	1.456	2.116	0.35	0.157	0.439	0.925	0.969	0.259	0.46	0.581
DB07615	Tranilast	1.458	1.456	2.068	1.642	1.357	0.985	0.976	0.996	0.953	0.963
DB00048	Collagenase clostridium histolyticum	1.462	1.341	2.922	1.027	0.885	0.91	0.881	0.989	0.669	0.736
DB01068	Clonazepam	1.462	0.436	1.72	0.598	0.308	0.923	0.599	0.939	0.626	0.541
DB03880	Batimastat	1.464	0.948	0.146	1.125	1.106	0.912	0.795	0.409	0.801	0.835
DB02567	PD173955	1.469	0.406	1.01	0.886	0.472	0.962	0.627	0.823	0.912	0.682
DB09225	Zotepine	1.469	0.733	1.961	2.518	0.36	0.918	0.74	0.968	1	0.593
DB00887	Bumetanide	1.481	1.707	2.312	0.281	0.072	0.921	0.952	0.984	0.529	0.451
DB00230	Pregabalin	1.483	1.407	0.596	0.895	0.577	0.931	0.904	0.581	0.601	0.647
DB04849	Cediranib	1.487	0.608	0.642	0.909	0.793	0.952	0.703	0.695	0.86	0.796
DB08382	Gosogliptin	1.488	1.71	-0.48	0.58	0.582	0.916	0.955	0.082	0.458	0.654
DB08429	N-({(2S)-1-[(3R)-3-amino-4-(3-chlorophenyl)butanoyl]pyrrolidin-2-yl}methyl)-3-(methylsulfonyl)benzamide	1.488	1.71	-0.48	0.58	0.582	0.916	0.955	0.082	0.458	0.654
DB00017	Salmon Calcitonin	1.489	1.323	0.977	1.525	1.261	0.893	0.86	0.738	0.878	0.837
DB00014	Goserelin	1.491	1.641	0.955	0.247	1.497	0.885	0.911	0.766	0.659	0.87
DB00050	Cetrorelix	1.491	1.641	0.955	0.247	1.497	0.885	0.911	0.766	0.659	0.87
DB00843	Donepezil	1.491	1.164	-0.393	0.726	-0.379	0.911	0.855	0.208	0.622	0.261
DB06719	Buserelin	1.491	1.641	0.955	0.247	1.497	0.885	0.911	0.766	0.659	0.87
DB01017	Minocycline	1.494	0.845	1.314	1.213	0.845	0.909	0.75	0.905	0.849	0.744
DB00555	Lamotrigine	1.496	1.347	1.17	1.821	0.102	0.926	0.893	0.825	0.968	0.435
DB00345	Aminohippuric acid	1.513	1.728	1.447	0.986	1.314	0.877	0.917	0.832	0.784	0.825
DB04272	Citric acid	1.513	1.532	0.045	1.314	1.351	0.976	0.965	0.438	0.904	0.921
DB00371	Meprobamate	1.515	0.758	1.603	0.44	0.692	0.936	0.724	0.919	0.548	0.687
DB01083	Orlistat	1.517	1.763	-0.686	0.002	0.101	0.924	0.957	0.158	0.412	0.463
DB00280	Disopyramide	1.519	2.415	-0.849	0.014	-0.046	0.923	0.991	0.105	0.36	0.367
DB07138	Neflamapimod	1.525	1.497	1.38	0.121	1.525	0.979	0.953	0.873	0.495	0.941
DB00343	Diltiazem	1.529	0.835	1.701	2.137	0.701	0.927	0.748	0.919	0.991	0.684
DB00212	Remikiren	1.534	1.411	2.01	1.508	1.503	0.951	0.926	0.992	0.909	0.974
DB09093	Chlortetracycline	1.538	1.867	0.871	0.518	0.32	0.921	0.954	0.685	0.555	0.544
DB04812	Benoxaprofen	1.543	1.601	2.018	1.075	1.5	0.899	0.901	0.926	0.885	0.903
DB00806	Pentoxifylline	1.552	1.269	0.204	0.679	0.53	0.924	0.867	0.477	0.551	0.606
DB00260	Cycloserine	1.554	1.528	0.145	1.57	0.795	0.929	0.915	0.383	0.923	0.722
DB08901	Ponatinib	1.554	1.132	0.814	1.536	0.065	0.981	0.892	0.691	0.92	0.465
DB07193	(2R,3R)-7-(methylsulfonyl)-3-(2,4,5-trifluorophenyl)-1,2,3,4-tetrahydropyrido[1,2-a]benzimidazol-2-amine	1.557	1.583	1.813	0.82	1.335	0.858	0.868	0.904	0.81	0.845
DB00302	Tranexamic acid	1.56	1.692	2.207	-0.128	1.579	0.907	0.912	0.944	0.295	0.924
DB00360	Sapropterin	1.56	1.336	2.045	1.273	0.243	0.93	0.882	0.96	0.796	0.514
DB00402	Eszopiclone	1.561	0.554	1.994	0.387	0.642	0.927	0.659	0.956	0.538	0.671
DB00172	Proline	1.562	0.838	0.911	1.407	0.006	0.926	0.764	0.732	0.922	0.435
DB04946	Iloperidone	1.57	0.501	2.118	2.029	0.774	0.938	0.64	0.968	0.991	0.754

DB01036	Tolterodine	1.572	1.56	0.507	1.23	-0.091	0.93	0.917	0.569	0.82	0.377
DB00203	Sildenafil	1.574	1.552	-0.372	0.228	0.898	0.929	0.928	0.26	0.479	0.768
DB00334	Olanzapine	1.575	0.778	1.779	2.574	0.291	0.943	0.776	0.941	0.998	0.572
DB00704	Naltrexone	1.575	1.894	-0.832	0.798	0.622	0.942	0.959	0.067	0.615	0.671
DB00420	Promazine	1.577	0.713	1.902	2.109	0.04	0.941	0.737	0.962	0.993	0.444
DB09304	Setiptiline	1.578	0.252	3.018	2.21	0.965	0.927	0.545	0.994	0.996	0.79
DB00104	Octreotide	1.579	1.967	-1.079	0.753	0.547	0.941	0.96	0.046	0.608	0.632
DB01035	Procainamide	1.579	1.044	0.753	0.253	1.441	0.93	0.809	0.643	0.523	0.889
DB00058	Alpha-1-proteinase inhibitor	1.582	1.565	1.665	0.226	1.632	0.868	0.876	0.882	0.691	0.875
DB04152	2-Amino-3-(3-Hydroxy-7,8-Dihydro-6h-Cyclohepta[D]-4-Isoxazolyl)Propionic Acid	1.584	2.216	1.289	0.52	-0.462	0.955	0.98	0.802	0.429	0.247
DB00231	Temazepam	1.605	0.833	1.666	0.583	0.273	0.936	0.77	0.925	0.627	0.513
DB05294	Vandetanib	1.607	0.398	1.49	0.276	1.166	0.961	0.607	0.913	0.575	0.926
DB00606	Cyclothiazide	1.608	2.117	2.379	0.31	-0.719	0.927	0.981	0.98	0.508	0.178
DB01520	Tenocyclidine	1.616	1.581	0.489	1.723	0.572	0.929	0.93	0.545	0.977	0.636
DB07621	(5-(PYRIDIN-3-YL)FURAN-2-YL)METHANAMINE	1.62	1.533	1.758	1.916	1.157	0.888	0.852	0.902	0.918	0.83
DB03461	2'-Monophosphoadenosine 5'-Diphosphoribose	1.624	0.406	-0.129	0.43	0.437	0.95	0.608	0.362	0.586	0.616
DB07919	7-METHOXY-1-METHYL-9H-BETA-CARBOLINE	1.629	0.848	0.804	0.017	0.425	0.94	0.784	0.687	0.439	0.61
DB01182	Propafenone	1.63	0.853	1.044	2.523	0.457	0.939	0.77	0.81	0.996	0.631
DB00845	Clofazimine	1.632	1.727	-1.692	1.572	1.474	0.937	0.944	0.018	0.876	0.902
DB00508	Triflupromazine	1.633	1.73	-0.864	1.017	-0.092	0.94	0.954	0.073	0.708	0.355
DB09119	Eslicarbazepine acetate	1.634	1.919	1.508	0.526	1.224	0.889	0.933	0.851	0.739	0.801
DB05311	Ecallantide	1.635	1.768	2.092	1.66	1.245	0.965	0.986	0.998	0.93	0.848
DB06273	Tocilizumab	1.636	1.821	1.529	1.724	1.181	0.893	0.923	0.856	0.889	0.803
DB00486	Nabilone	1.639	1.723	1.182	1.143	1.601	0.936	0.944	0.782	0.796	0.907
DB00755	Tretinoin	1.641	1.925	0.72	1.638	0.433	0.982	0.983	0.683	0.948	0.617
DB01100	Pimozide	1.641	0.308	2.544	2.111	0.809	0.971	0.594	0.995	0.991	0.793
DB00907	Cocaine	1.655	1.245	1.221	1.159	0.095	0.941	0.868	0.829	0.823	0.435
DB04473	Alpha-L-Fucose	1.657	1.636	2.227	0.182	2.301	0.868	0.882	0.946	0.522	0.931
DB04639	Biphenylalanine	1.657	1.636	2.227	0.182	2.301	0.868	0.882	0.946	0.522	0.931
DB01139	Cefapirin	1.663	1.012	0.402	1.309	1.452	0.947	0.83	0.57	0.887	0.921
DB00456	Cefalotin	1.664	1.171	0.505	0.827	1.094	0.935	0.844	0.549	0.72	0.819
DB00318	Codeine	1.666	1.878	-0.27	0.891	0.434	0.956	0.973	0.105	0.605	0.607
DB00327	Hydromorphone	1.666	1.878	-0.27	0.891	0.434	0.956	0.973	0.105	0.605	0.607
DB00611	Butorphanol	1.666	1.878	-0.27	0.891	0.434	0.956	0.973	0.105	0.605	0.607
DB00622	Nicardipine	1.666	0.752	1.231	2.381	0.422	0.971	0.773	0.854	0.998	0.6
DB00844	Nalbuphine	1.666	1.878	-0.27	0.891	0.434	0.956	0.973	0.105	0.605	0.607
DB00854	Levorphanol	1.666	1.878	-0.27	0.891	0.434	0.956	0.973	0.105	0.605	0.607
DB00899	Remifentanil	1.666	1.878	-0.27	0.891	0.434	0.956	0.973	0.105	0.605	0.607
DB00956	Hydrocodone	1.666	1.878	-0.27	0.891	0.434	0.956	0.973	0.105	0.605	0.607
DB01184	Domperidone	1.666	0.645	2.932	2.496	1.26	0.989	0.73	0.994	0.995	0.887
DB01192	Oxymorphone	1.666	1.878	-0.27	0.891	0.434	0.956	0.973	0.105	0.605	0.607
DB01439	3-Methylthiofentanyl	1.666	1.878	-0.27	0.891	0.434	0.956	0.973	0.105	0.605	0.607
DB01462	Etonitazene	1.666	1.878	-0.27	0.891	0.434	0.956	0.973	0.105	0.605	0.607
DB01535	Carfentanil	1.666	1.878	-0.27	0.891	0.434	0.956	0.973	0.105	0.605	0.607
DB01571	3-Methylfentanyl	1.666	1.878	-0.27	0.891	0.434	0.956	0.973	0.105	0.605	0.607
DB05302		1.666	1.878	-0.27	0.891	0.434	0.956	0.973	0.105	0.605	0.607
DB05508		1.666	1.878	-0.27	0.891	0.434	0.956	0.973	0.105	0.605	0.607
DB09272	Eluxadoline	1.666	1.878	-0.27	0.891	0.434	0.956	0.973	0.105	0.605	0.607
DB09396		1.666	1.878	-0.27	0.891	0.434	0.956	0.973	0.105	0.605	0.607
DB00799	Tazarotene	1.668	2.823	1.198	0.207	-0.2	0.938	0.997	0.844	0.5	0.332
DB06589	Pazopanib	1.671	0.011	0.529	0.755	1.188	0.969	0.423	0.653	0.818	0.921
DB04799	6-Hydroxy-5-undecyl-4,7-benzothiazoledione	1.672	1.533	0.766	-0.068	0.158	0.939	0.918	0.634	0.36	0.462

DB07401	METHYL (2Z)-2-(2-{[6-(2-CYANOPHENOXY)PYRIMIDIN-4-YL]OXY}PHENYL)-3-METHOXYACRYLATE	1.672	1.533	0.766	-0.068	0.158	0.939	0.918	0.634	0.36	0.462
DB07763	(5S)-3-ANILINO-5-(2,4-DIFLUOROPHENYL)-5-METHYL-1,3-OXAZOLIDINE-2,4-DIONE	1.672	1.533	0.766	-0.068	0.158	0.939	0.918	0.634	0.36	0.462
DB07778	FAMOXADONE	1.672	1.533	0.766	-0.068	0.158	0.939	0.918	0.634	0.36	0.462
DB08330	METHYL (2Z)-3-METHOXY-2-{2-[(E)-2-PHENYLVINYL]PHENYL}ACRYLATE	1.672	1.533	0.766	-0.068	0.158	0.939	0.918	0.634	0.36	0.462
DB08453	2-NONYL-4-HYDROXYQUINOLINE N-OXIDE	1.672	1.533	0.766	-0.068	0.158	0.939	0.918	0.634	0.36	0.462
DB08690	UBIQUINONE-2	1.672	1.533	0.766	-0.068	0.158	0.939	0.918	0.634	0.36	0.462
DB09194	Etoperidone	1.674	1.692	2.711	0.868	0.254	0.95	0.936	0.99	0.609	0.506
DB00200	Hydroxocobalamin	1.681	1.804	0.791	0.666	-0.036	0.954	0.954	0.691	0.622	0.371
DB06335	Saxagliptin	1.682	1.734	-0.445	0.791	0.813	0.943	0.954	0.082	0.56	0.735
DB00116	Tetrahydrofolic acid	1.685	1.218	0.907	1.415	1.293	0.993	0.937	0.717	0.897	0.901
DB04141	2-Hexyloxy-6-Hydroxymethyl-Tetrahydro-Pyran-3,4,5-Triol	1.686	1.286	-0.351	0.531	0.233	0.943	0.883	0.223	0.597	0.507
DB00800	Fenoldopam	1.698	1.196	1.316	1.92	0.981	0.944	0.85	0.936	0.993	0.796
DB02818	Iodo-Willardiine	1.703	2.152	1.116	0.352	0.195	0.965	0.975	0.782	0.362	0.498
DB02966	Fluoro-Willardiine	1.703	2.152	1.116	0.352	0.195	0.965	0.975	0.782	0.362	0.498
DB04000	Bromo-Willardiine	1.703	2.152	1.116	0.352	0.195	0.965	0.975	0.782	0.362	0.498
DB02621	Latrunculin A	1.709	1.209	1.196	0.489	0.071	0.951	0.872	0.835	0.564	0.439
DB01189	Desflurane	1.713	1.039	1.571	1.083	0.116	0.952	0.879	0.946	0.902	0.473
DB01236	Sevoflurane	1.713	1.039	1.571	1.083	0.116	0.952	0.879	0.946	0.902	0.473
DB01071	Mequitazine	1.716	1.632	0.17	0.929	0.116	0.95	0.941	0.443	0.706	0.441
DB00404	Alprazolam	1.722	0.698	1.083	0.885	0.918	0.951	0.724	0.815	0.762	0.762
DB00788	Naproxen	1.725	1.689	-0.15	0.018	1.28	0.949	0.943	0.23	0.409	0.862
DB00433	Prochlorperazine	1.735	1.299	1.659	1.304	1.216	0.991	0.898	0.936	0.901	0.898
DB00786	Marimastat	1.74	0.761	2.11	1.336	1.72	0.958	0.754	0.973	0.9	0.946
DB01351	Amobarbital	1.744	1.472	2.469	0.178	0.088	0.957	0.911	0.98	0.412	0.426
DB01352	Aprobarbital	1.744	1.472	2.469	0.178	0.088	0.957	0.911	0.98	0.412	0.426
DB01353	Butobarbital	1.744	1.472	2.469	0.178	0.088	0.957	0.911	0.98	0.412	0.426
DB01354	Heptabarbital	1.744	1.472	2.469	0.178	0.088	0.957	0.911	0.98	0.412	0.426
DB01355	Hexobarbital	1.744	1.472	2.469	0.178	0.088	0.957	0.911	0.98	0.412	0.426
DB01483	Barbital	1.744	1.472	2.469	0.178	0.088	0.957	0.911	0.98	0.412	0.426
DB01496	Barbituric acid derivative	1.744	1.472	2.469	0.178	0.088	0.957	0.911	0.98	0.412	0.426
DB06695	Dabigatran etexilate	1.744	1.785	1.952	-0.15	0.223	0.939	0.957	0.95	0.365	0.479
DB00189	Ethchlorvynol	1.753	0.973	2.139	0.326	0.483	0.951	0.79	0.971	0.503	0.6
DB01107	Methyprylon	1.753	0.973	2.139	0.326	0.483	0.951	0.79	0.971	0.503	0.6
DB01437	Glutethimide	1.753	0.973	2.139	0.326	0.483	0.951	0.79	0.971	0.503	0.6
DB01907		1.755	0.154	-0.986	0.821	1.244	0.96	0.523	0.133	0.807	0.902
DB00836	Loperamide	1.759	1.658	0.104	3.282	0.667	0.993	0.955	0.452	1	0.716
DB07376	5-(DIMETHYLAMINO)-1-NAPHTHALENESULFONIC ACID(DANSYL ACID)	1.76	2.266	1.661	0.539	0.729	0.927	0.947	0.912	0.683	0.871
DB06800	Methylnaltrexone	1.767	1.931	-0.603	0.83	0.524	0.955	0.966	0.104	0.616	0.641
DB01205	Flumazenil	1.778	1.038	1.707	-0.173	0.901	0.951	0.823	0.932	0.316	0.778
DB00186	Lorazepam	1.783	0.757	1.633	0.477	0.738	0.953	0.733	0.923	0.594	0.708
DB00628	Clorazepic acid	1.783	0.757	1.633	0.477	0.738	0.953	0.733	0.923	0.594	0.708
DB02556	D-Phenylalanine	1.787	1.517	1.996	2.165	2.023	0.901	0.859	0.919	0.934	0.932
DB00126	Ascorbic acid	1.793	1.36	1.238	-0.085	-0.307	0.962	0.898	0.845	0.377	0.31
DB05229	Beraprost	1.796	1.891	0.557	0.923	0.7	0.965	0.957	0.529	0.649	0.688
DB00695	Furosemide	1.798	1.624	1.768	0.145	0.197	0.966	0.942	0.932	0.419	0.476
DB04855	Dronedarone	1.798	0.543	2.022	0.771	0.886	0.959	0.664	0.982	0.746	0.772
DB00761	Potassium chloride	1.802	2.44	1.664	0.958	0.167	0.957	0.992	0.917	0.726	0.463
DB00141	N-Acetylglucosamine	1.804	1.986	0.573	0.91	-0.3	0.95	0.964	0.587	0.691	0.295
DB01244	Bepridil	1.806	0.489	1.554	2.592	2.363	0.98	0.651	0.922	0.993	0.998

DB04532	Indole	1.819	1.712	1.98	2.884	1.596	0.963	0.952	0.901	0.994	0.924
DB00652	Pentazocine	1.824	1.931	-0.217	1.254	0.681	0.96	0.97	0.136	0.766	0.658
DB01079	Tegaserod	1.831	0.615	-0.018	2.159	0.935	0.953	0.673	0.318	0.983	0.785
DB08190	N-[2-(2-iodo-5-methoxy-1H-indol-3-yl)ethyl]acetamide	1.833	1.758	1.084	1.467	0.28	0.966	0.947	0.792	0.832	0.521
DB06372	Rilonacept	1.834	2.201	1.702	-0.279	1.596	0.926	0.942	0.91	0.184	0.913
DB07841	Geranylgeranyl diphosphate	1.837	3.047	0.803	1.336	-1.214	0.954	1	0.678	0.805	0.095
DB00546	Adinazolam	1.844	1.042	1.54	0.25	0.427	0.957	0.817	0.893	0.469	0.572
DB00729		1.845	1.841	2.495	0.713	1.309	0.912	0.91	0.943	0.834	0.886
DB09357	Dexpanthenol	1.845	1.841	2.495	0.713	1.309	0.912	0.91	0.943	0.834	0.886
DB00926	Etretinate	1.846	2.927	1.339	-0.34	0.753	0.954	0.995	0.912	0.283	0.708
DB01577	Metamfetamine	1.847	1.473	2.06	0.65	0.214	0.957	0.925	0.954	0.59	0.476
DB01206	Lomustine	1.854	1.603	1.832	1.735	1.705	0.977	0.954	0.877	0.919	0.955
DB06691	Mepyramine	1.867	1.284	0.606	1.305	0.315	0.96	0.881	0.618	0.889	0.527
DB00829	Diazepam	1.873	1.112	1.461	0.61	0.557	0.97	0.85	0.897	0.637	0.615
DB02687		1.874	1.452	0.503	-0.394	1.097	0.97	0.949	0.756	0.273	0.908
DB00292	Etomidate	1.881	1.822	3.111	1.188	0.364	0.971	0.961	0.996	0.868	0.552
DB00392	Profenamine	1.883	2.098	-1.695	1.208	1.018	0.978	0.986	0.036	0.83	0.787
DB01425	Alizapride	1.885	1.787	4.283	0.404	1.629	0.907	0.905	0.995	0.706	0.9
DB04519	Caprylic acid	1.889	1.903	1.187	1.529	1.657	0.917	0.924	0.812	0.877	0.886
DB09118	Stiripentol	1.894	1.155	2.292	0.204	-0.446	0.971	0.847	0.981	0.495	0.246
DB00805	Minaprine	1.895	1.287	0.438	0.922	0.145	0.97	0.88	0.561	0.669	0.472
DB01452	Diamorphine	1.897	2.441	0.433	0.956	0.606	0.977	0.99	0.498	0.641	0.661
DB00001	Lepirudin	1.898	1.781	1.987	1.03	1.888	0.924	0.91	0.91	0.829	0.916
DB00006	Bivalirudin	1.898	1.781	1.987	1.03	1.888	0.924	0.91	0.91	0.829	0.916
DB01123	Proflavine	1.898	1.781	1.987	1.03	1.888	0.924	0.91	0.91	0.829	0.916
DB11095	Desirudin	1.898	1.781	1.987	1.03	1.888	0.924	0.91	0.91	0.829	0.916
DB01049	Ergoloid mesylate	1.899	0.509	2.768	1.9	1.089	0.978	0.662	0.995	0.977	0.861
DB00767	Benzquinamide	1.9	1.69	2.839	1.266	-0.308	0.969	0.942	0.993	0.865	0.296
DB00835	Brompheniramine	1.907	1.883	0.305	1.125	0.054	0.96	0.967	0.476	0.799	0.421
DB08801	Dimetindene	1.907	1.883	0.305	1.125	0.054	0.96	0.967	0.476	0.799	0.421
DB00753	Isoflurane	1.908	1.026	0.842	1.966	0.928	0.993	0.868	0.824	0.985	0.823
DB00726	Trimipramine	1.921	1.614	0.844	2.23	-0.189	0.971	0.946	0.733	0.992	0.34
DB00210	Adapalene	1.926	2.865	1.478	-0.035	0.687	0.965	0.999	0.922	0.395	0.693
DB01062	Oxybutynin	1.928	1.88	0.838	1.468	0.104	0.969	0.969	0.716	0.929	0.456
DB09167	Dosulepin	1.93	1.076	1.452	1.198	-0.268	0.972	0.828	0.887	0.843	0.326
DB00920	Ketotifen	1.932	1.023	0.844	1.126	0.648	0.977	0.833	0.751	0.839	0.69
DB00145	Glycine	1.935	0.959	-0.056	2.024	1.654	0.989	0.864	0.399	0.989	0.97
DB00897	Triazolam	1.942	0.804	1.544	0.562	0.874	0.961	0.759	0.909	0.625	0.773
DB01591	Solifenacin	1.947	1.879	0.495	1.262	0.088	0.962	0.964	0.547	0.837	0.45
DB06148	Mianserin	1.947	0.779	2.529	2.781	0.954	0.979	0.757	0.994	1	0.802
DB04275	N-acetylserotonin	1.952	1.704	1.041	1.46	0.246	0.967	0.937	0.769	0.829	0.517
DB01232	Saquinavir	1.958	1.232	-0.457	0.82	1.918	0.973	0.866	0.198	0.741	0.974
DB00503	Ritonavir	1.96	1.819	0.213	1.438	0.027	0.962	0.958	0.478	0.914	0.421
DB00381	Amlodipine	1.967	1.16	3.159	1.6	0.232	0.983	0.906	0.998	0.985	0.504
DB00497	Oxycodone	1.97	2.263	-0.546	1.049	0.789	0.959	0.984	0.096	0.685	0.712
DB00039	Palifermin	1.974	1.501	1.332	1.141	0.199	0.97	0.925	0.923	0.796	0.485
DB00813	Fentanyl	1.985	2.191	-0.205	1.157	0.651	0.966	0.982	0.124	0.725	0.664
DB01176	Cyclizine	1.992	1.48	-0.011	1.447	0.092	0.97	0.926	0.391	0.904	0.446
DB08039	(3Z)-N,N-DIMETHYL-2-OXO-3-(4,5,6,7-TETRAHYDRO-1H-INDOL-2-YLMETHYLIDENE)-2,3-DIHYDRO-1H-INDOLE-5-SULFONAMIDE	2.001	1.58	0.192	2.345	0.966	1	0.956	0.492	0.984	0.812
DB01873	Epothilone D	2.007	1.656	0.81	2.126	0.599	0.99	0.977	0.721	0.974	0.717
DB03010	Patupilone	2.007	1.656	0.81	2.126	0.599	0.99	0.977	0.721	0.974	0.717
DB01151	Desipramine	2.013	1.475	0.695	2.154	-0.171	0.976	0.935	0.692	0.993	0.352
DB03147	Flavin adenine dinucleotide	2.015	1.375	0.691	0.454	0.645	0.975	0.917	0.689	0.641	0.695
DB08222	METHOXYUNDECYLPHOSPHINIC ACID	2.028	2.173	3.303	2.252	1.727	0.986	0.989	1	0.968	0.92
DB00129	Ornithine	2.029	1.404	1.379	0.538	0.197	0.975	0.906	0.857	0.611	0.472

DB00804	Dicyclomine	2.03	1.693	1.103	1.667	-0.075	0.971	0.941	0.793	0.949	0.391
DB03773	alpha-D-quinovopyranose	2.035	1.819	2.355	2.169	2.01	0.986	0.955	0.997	0.966	0.977
DB00416	Metocurine Iodide	2.036	2.198	2.176	2.185	1.811	0.971	0.983	0.979	0.974	0.961
DB00565	Cisatracurium	2.036	2.198	2.176	2.185	1.811	0.971	0.983	0.979	0.974	0.961
DB00732	Atracurium besylate	2.036	2.198	2.176	2.185	1.811	0.971	0.983	0.979	0.974	0.961
DB00992	Methyl aminolevulinate	2.037	2.231	0.938	2.505	2.033	0.968	0.98	0.749	0.969	0.968
DB01548	Diprenorphine	2.04	2.15	-0.241	1.097	0.814	0.973	0.971	0.117	0.677	0.729
DB01142	Doxepin	2.042	1.042	2.235	2.329	0.49	0.982	0.835	0.977	0.998	0.639
DB11311	Prothrombin	2.046	1.881	1.441	0.907	0.675	0.976	0.97	0.869	0.616	0.679
DB00683	Midazolam	2.048	1.065	1.432	0.786	0.862	0.979	0.842	0.888	0.719	0.747
DB01160	Dinoprost tromethamine	2.049	1.371	0.204	1.18	1.119	0.979	0.902	0.462	0.821	0.824
DB00122	Choline	2.056	1.379	1.187	0.927	0.976	0.969	0.901	0.819	0.682	0.775
DB00550	Propylthiouracil	2.08	2.181	2.453	2.461	1.519	0.98	0.991	0.998	0.981	0.886
DB00408	Loxapine	2.094	0.895	2.699	2.683	0.89	0.985	0.782	0.994	0.999	0.781
DB09536	Titanium dioxide	2.102	2.281	2.868	1.882	1.943	0.974	0.985	0.99	0.962	0.965
DB00100	Coagulation Factor IX (Recombinant)	2.12	1.64	1.392	1.294	1.017	0.979	0.924	0.868	0.786	0.79
DB13152	Coagulation Factor IX Human	2.12	1.64	1.392	1.294	1.017	0.979	0.924	0.868	0.786	0.79
DB00349	Clobazam	2.121	1.181	1.786	0.378	0.606	0.977	0.871	0.946	0.538	0.663
DB00801	Halazepam	2.121	1.181	1.786	0.378	0.606	0.977	0.871	0.946	0.538	0.663
DB01215	Estazolam	2.121	1.181	1.786	0.378	0.606	0.977	0.871	0.946	0.538	0.663
DB01558	Bromazepam	2.121	1.181	1.786	0.378	0.606	0.977	0.871	0.946	0.538	0.663
DB01588	Prazepam	2.121	1.181	1.786	0.378	0.606	0.977	0.871	0.946	0.538	0.663
DB01589	Quazepam	2.121	1.181	1.786	0.378	0.606	0.977	0.871	0.946	0.538	0.663
DB00661	Verapamil	2.123	0.847	3.069	1.981	0.983	0.992	0.795	0.999	0.995	0.827
DB01254	Dasatinib	2.13	0.603	1.087	1.097	0.272	0.995	0.716	0.831	0.932	0.554
DB00289	Atomoxetine	2.154	1.148	0.124	2.869	1.435	0.987	0.861	0.454	0.999	0.924
DB00587	Cinalukast	2.156	2.027	1.554	2.346	1.963	0.979	0.98	0.914	0.98	0.97
DB06274	Alvimopan	2.169	2.322	-0.268	1.009	0.782	0.978	0.981	0.117	0.652	0.716
DB09146	Iron saccharate	2.178	2.031	2.329	1.481	0.983	0.948	0.948	0.959	0.928	0.887
DB00295	Morphine	2.181	2.604	1.232	2.294	0.99	0.984	0.992	0.824	0.993	0.791
DB00690	Flurazepam	2.19	1.251	2.097	0.439	0.689	0.983	0.88	0.963	0.576	0.684
DB00921	Buprenorphine	2.204	2.627	0.378	1.55	0.839	0.984	0.99	0.484	0.94	0.73
DB09089	Trimebutine	2.209	1.816	1.198	0.544	0.948	0.987	0.968	0.846	0.617	0.775
DB00475	Chlordiazepoxide	2.212	1.271	1.825	0.377	0.638	0.983	0.878	0.949	0.532	0.678
DB00917	Dinoprostone	2.212	1.28	1.754	2.592	2.328	0.985	0.881	0.925	1	0.988
DB01199	Tubocurarine	2.214	1.93	1.099	1.343	1.118	0.984	0.974	0.784	0.883	0.817
DB04841	Flunarizine	2.219	1.321	1.114	1.968	1.23	0.999	0.928	0.887	0.986	0.898
DB00904	Ondansetron	2.222	1.682	0.664	1.892	1.152	0.981	0.943	0.664	0.968	0.832
DB06707	Levonordefrin	2.222	2.139	3.76	-0.19	2.383	0.94	0.93	0.988	0.39	0.953
DB01028	Methoxyflurane	2.225	1.476	2.124	1.069	0.513	0.981	0.924	0.969	0.829	0.614
DB01454	Midomafetamine	2.225	1.433	1.588	1.366	0.253	0.985	0.9	0.915	0.88	0.519
DB01329	Cefoperazone	2.228	2.374	0.84	1.606	2.245	0.935	0.952	0.784	0.909	0.935
DB00493	Cefotaxime	2.229	1.743	1.212	0.688	0.22	0.985	0.942	0.819	0.666	0.517
DB01081	Diphenoxylate	2.243	2.466	-2.194	1.21	1.272	0.981	0.985	0.003	0.743	0.859
DB03109	N-Acetyl-D-Allosamine	2.247	2.164	1.939	1.66	1.953	0.963	0.95	0.908	0.893	0.929
DB04404	Allosamizoline	2.247	2.164	1.939	1.66	1.953	0.963	0.95	0.908	0.893	0.929
DB09224	Melperone	2.249	1.417	2.475	1.571	0.784	0.985	0.898	0.988	0.944	0.723
DB01349	Tasosartan	2.252	2.36	2.033	0.247	2.152	0.935	0.932	0.936	0.585	0.94
DB06817	Raltegravir	2.252	2.189	1.019	2.242	2.044	0.973	0.968	0.762	0.952	0.948
DB01069	Promethazine	2.254	1.531	2.245	2.948	1.112	0.999	0.957	0.981	1	0.873
DB01245	Decamethonium	2.254	2.966	0.859	0.087	1.57	0.955	0.973	0.741	0.402	0.939
DB01631	Methyl Nonanoate (Ester)	2.255	1.486	3.064	0.121	1.214	0.976	0.936	0.992	0.493	0.9
DB00708	Sufentanil	2.259	2.181	0.902	1.402	0.893	0.982	0.978	0.704	0.899	0.743
DB01595	Nitrazepam	2.271	1.251	1.663	0.406	0.685	0.985	0.886	0.923	0.55	0.683
DB00483	Gallamine Triethiodide	2.28	2.2	1.255	2.386	1.253	0.985	0.98	0.837	0.995	0.856
DB01233	Metoclopramide	2.282	1.406	1.847	1.701	0.18	0.989	0.909	0.94	0.947	0.472
DB00374	Treprostinil	2.283	1.531	0.731	1.34	0.856	0.99	0.924	0.642	0.839	0.739
DB02057	(S)-AMPA	2.287	2.873	0.96	0.623	-0.181	0.987	0.999	0.721	0.489	0.348

DB00450	Droperidol	2.295	1.229	2.903	1.453	0.832	0.989	0.872	0.995	0.907	0.735
DB04868	Nilotinib	2.295	0.853	0.261	1.161	1.633	0.998	0.807	0.544	0.896	0.967
DB09409	Magnesium acetate tetrahydrate	2.295	1.996	0.651	1.862	1.121	0.981	0.972	0.609	0.988	0.818
DB09481	Magnesium carbonate	2.295	1.996	0.651	1.862	1.121	0.981	0.972	0.609	0.988	0.818
DB00458	Imipramine	2.298	1.591	1.762	2.037	0.513	0.992	0.963	0.947	0.991	0.65
DB07374	Anisomycin	2.299	1.914	1.151	-2.128	1.728	0.985	0.957	0.726	0.004	0.941
DB08437	Puromycin	2.299	1.914	1.151	-2.128	1.728	0.985	0.957	0.726	0.004	0.941
DB00307	Bexarotene	2.305	3.003	1.413	0.616	0.489	0.987	0.999	0.944	0.678	0.632
DB02494	Alpha-Hydroxy-Beta-Phenyl-Propionic Acid	2.309	1.882	1.296	-2.032	1.561	0.982	0.96	0.817	0.011	0.926
DB02789	Pregnenolone	2.312	1.58	3.601	0.657	1.83	0.954	0.94	0.987	0.735	0.946
DB05679	Ustekinumab	2.322	2.206	2.986	2.165	2.774	0.967	0.957	0.97	0.934	0.986
DB01159	Halothane	2.327	1.334	1.718	1.327	0.697	0.993	0.916	0.958	0.958	0.716
DB01624	Zuclopenthixol	2.33	2.768	1.206	1.371	-0.265	0.982	0.999	0.816	0.918	0.289
DB09203	Synephrine	2.336	2.139	2.391	2.705	2.339	0.984	0.976	0.94	0.979	0.989
DB00353	Methylergometrine	2.346	1.363	2.511	1.801	1.237	0.986	0.892	0.988	0.956	0.854
DB01565	Dihydromorphine	2.35	2.255	-0.255	1.165	1.059	0.987	0.978	0.119	0.724	0.787
DB00490	Buspirone	2.352	1.229	3.331	1.189	0.545	0.992	0.866	0.999	0.812	0.604
DB01559	Clotiazepam	2.357	1.64	1.56	0.182	0.407	0.986	0.949	0.9	0.439	0.57
DB01567	Fludiazepam	2.357	1.64	1.56	0.182	0.407	0.986	0.949	0.9	0.439	0.57
DB01594	Cinolazepam	2.357	1.64	1.56	0.182	0.407	0.986	0.949	0.9	0.439	0.57
DB01175	Escitalopram	2.36	1.8	1.165	0.775	0.976	0.979	0.954	0.798	0.607	0.781
DB00908	Quinidine	2.362	0.849	3.165	2.743	1.145	0.988	0.773	0.999	0.999	0.849
DB00545	Pyridostigmine	2.366	1.927	-0.642	0.773	1.031	0.993	0.962	0.202	0.661	0.785
DB09000	Cyamemazine	2.379	1.496	1.08	1.812	0.442	0.982	0.915	0.795	0.964	0.592
DB00215	Citalopram	2.382	1.382	0.977	1.337	0.311	0.989	0.898	0.759	0.882	0.532
DB00543	Amoxapine	2.389	0.722	1.707	1.892	1.157	0.996	0.739	0.941	0.985	0.872
DB00647	Dextropropoxyphene	2.393	2.473	0.313	1.118	0.769	0.986	0.994	0.46	0.733	0.691
DB00245	Benzatropine	2.397	1.671	1.582	1.809	0.479	0.992	0.954	0.922	0.969	0.617
DB00775	Tirofiban	2.417	2.524	1.233	0.758	2.293	0.947	0.956	0.829	0.715	0.949
DB06743	Ginkgolide A	2.436	2.504	2.214	3.293	2.414	0.965	0.964	0.942	0.986	0.961
DB00909	Zonisamide	2.437	1.837	3.614	2.043	0.967	0.993	0.976	1	0.989	0.8
DB00572	Atropine	2.443	1.826	0.995	1.847	-0.006	0.992	0.959	0.767	0.969	0.425
DB01403	Methotrimeprazine	2.451	1.531	3.117	2.1	0.632	0.994	0.931	0.998	0.986	0.68
DB00285	Venlafaxine	2.476	1.94	0.279	1.423	0.617	0.991	0.975	0.486	0.922	0.665
DB01118	Amiodarone	2.481	2.244	1.458	2.251	1.863	0.999	0.994	0.906	0.993	0.978
DB00234	Reboxetine	2.492	1.88	0.698	0.886	0.776	0.99	0.959	0.633	0.644	0.709
DB06731	Seproxetine	2.494	1.786	0.097	1.392	0.384	0.993	0.954	0.39	0.899	0.563
DB04465	Lactose	2.504	2.201	0.162	0.858	1.222	0.989	0.972	0.263	0.69	0.86
DB00777	Propiomazine	2.513	1.469	1.154	1.482	0.187	0.993	0.915	0.816	0.92	0.482
DB01776	M-Cresol	2.525	2.219	0.648	1.566	2.87	0.948	0.936	0.711	0.934	0.97
DB04838	Cyclandelate	2.534	2.902	2.263	0.92	2.529	0.947	0.973	0.946	0.848	0.96
DB00967	Desloratadine	2.552	2.143	1.049	1.642	0.193	0.991	0.986	0.788	0.945	0.476
DB13151	Anti-inhibitor coagulant complex	2.571	1.741	1.545	1.359	1.47	0.991	0.937	0.893	0.819	0.909
DB08879	Belimumab	2.595	2.43	2.126	2.9	2.369	0.964	0.961	0.932	0.973	0.946
DB00578	Carbenicillin	2.6	2.489	2.003	0.81	1.743	0.958	0.948	0.94	0.823	0.915
DB00979	Cyclopentolate	2.6	2.489	2.003	0.81	1.743	0.958	0.948	0.94	0.823	0.915
DB00418	Secobarbital	2.603	1.756	1.397	1.676	1.726	0.995	0.958	0.877	0.953	0.958
DB00459	Acitretin	2.609	3.444	0.242	0.073	1.213	0.992	0.998	0.333	0.452	0.84
DB01104	Sertraline	2.609	1.769	1.952	2.023	0.698	0.999	0.969	0.956	0.987	0.707
DB04016	2-[3-({Methyl[1-(2-Naphthoyl)Piperidin-4-Yl]Amino}Carbonyl)-2-Naphthyl]-1-(1-Naphthyl)-2-Oxoethylphosphonic Acid	2.62	2.866	2.598	-0.277	2.388	0.952	0.971	0.957	0.207	0.946
DB00372	Thiethylperazine	2.625	1.51	2.022	2.06	0.898	0.993	0.927	0.959	0.993	0.769
DB09018	Bromopride	2.655	2.278	3.493	3.144	2.113	0.96	0.934	0.984	0.979	0.934
DB01497	Etorphine	2.663	2.504	0.407	1.571	1.489	0.99	0.994	0.491	0.941	0.92
DB00985	Dimenhydrinate	2.666	1.798	0.562	1.46	0.206	0.996	0.965	0.604	0.914	0.499

DB06717	Fosaprepitant	2.702	2.382	1.422	3.543	2.396	0.979	0.958	0.845	0.994	0.964
DB09048	Netupitant	2.702	2.382	1.422	3.543	2.396	0.979	0.958	0.845	0.994	0.964
DB09291	Rolapitant	2.702	2.382	1.422	3.543	2.396	0.979	0.958	0.845	0.994	0.964
DB00142	Glutamic Acid	2.718	1.513	0.85	1.975	1.069	0.999	0.972	0.773	0.995	0.894
DB00312	Pentobarbital	2.721	1.35	1.582	1.621	1.205	0.995	0.894	0.912	0.961	0.887
DB00283	Clemastine	2.723	2.281	1.118	2.127	0.638	0.997	0.99	0.818	0.994	0.689
DB00237	Butabarbital	2.724	1.598	2.193	0.557	0.919	0.996	0.944	0.971	0.621	0.779
DB00241	Butalbital	2.724	1.598	2.193	0.557	0.919	0.996	0.944	0.971	0.621	0.779
DB00306	Talbutal	2.724	1.598	2.193	0.557	0.919	0.996	0.944	0.971	0.621	0.779
DB00463	Metharbital	2.724	1.598	2.193	0.557	0.919	0.996	0.944	0.971	0.621	0.779
DB00794	Primidone	2.724	1.598	2.193	0.557	0.919	0.996	0.944	0.971	0.621	0.779
DB00933	Mesoridazine	2.726	1.367	1.32	1.815	1.088	0.998	0.901	0.86	0.98	0.83
DB01043	Memantine	2.747	2.375	1.004	1.564	1.265	0.993	0.988	0.737	0.934	0.873
DB01228	Encainide	2.748	2.563	3.901	2.171	1.026	0.992	0.99	1	0.985	0.795
DB00514	Dextromethorphan	2.75	1.501	-0.832	2.533	1.942	0.998	0.923	0.143	0.995	0.976
DB01075	Diphenhydramine	2.752	1.932	0.427	1.694	0.389	0.997	0.967	0.577	0.952	0.576
DB04819	Methapyrilene	2.753	2.004	0.955	1.104	0.385	0.996	0.98	0.76	0.807	0.565
DB00723	Methoxamine	2.755	2.711	1.01	0.602	2.148	0.958	0.953	0.828	0.775	0.929
DB00568	Cinnarizine	2.758	1.669	2.595	1.845	0.979	0.999	0.959	0.987	0.986	0.825
DB00770	Alprostadil	2.765	1.909	1.565	2.161	2.173	0.994	0.966	0.907	0.985	0.982
DB00160	L-Alanine	2.766	2.084	0.285	0.842	0.676	0.997	0.978	0.496	0.697	0.675
DB02517	D-Glutamic Acid	2.768	2.08	1.589	0.786	0.726	0.997	0.982	0.902	0.658	0.692
DB02504	[3-(1-Benzyl-3-Carbamoylmethyl-2-Methyl-1h-Indol-5-Yloxy)-Propyl-]-Phosphonic Acid	2.772	2.532	2.573	4.258	2.927	0.972	0.951	0.956	0.994	0.978
DB00424	Hyoscyamine	2.79	1.941	0.324	1.375	0.359	0.993	0.966	0.481	0.869	0.547
DB00889	Granisetron	2.792	2.703	2.476	1.33	0.743	0.996	0.994	0.983	0.851	0.692
DB00996	Gabapentin	2.796	2.135	0.698	1.914	1.61	1	0.985	0.657	0.99	0.951
DB08235	N-[2-(2-methyl-1H-indol-3-yl)ethyl]thiophene-2-carboxamide	2.799	1.317	1.409	1.38	2.352	0.996	0.871	0.937	0.859	0.987
DB08236	(2S)-2-(3-bromophenyl)-3-(5-chloro-2-hydroxyphenyl)-1,3-thiazolidin-4-one	2.799	1.317	1.409	1.38	2.352	0.996	0.871	0.937	0.859	0.987
DB05777	Thrombomodulin Alfa	2.808	2.652	2.052	2.004	2.652	0.963	0.96	0.938	0.946	0.958
DB00333	Methadone	2.851	2.34	0.208	1.881	1.317	0.998	0.985	0.416	0.974	0.861
DB01242	Clomipramine	2.869	1.544	2.469	1.75	0.348	0.998	0.94	0.983	0.97	0.555
DB01338	Pipecuronium	2.887	3.153	1.87	1.631	2.031	0.963	0.975	0.943	0.942	0.958
DB00010	Sermorelin	2.897	2.697	1.76	2.9	3.03	0.997	0.995	0.978	0.997	0.999
DB01844	N,N-dimethylformamide	2.897	2.3	3.208	2.955	2.93	0.982	0.944	0.988	0.97	0.978
DB04953	Ezogabine	2.897	2.971	1.906	2.527	1.807	0.957	0.967	0.931	0.966	0.923
DB08869	Tesamorelin	2.897	2.697	1.76	2.9	3.03	0.997	0.995	0.978	0.997	0.999
DB00659	Acamprosate	2.901	1.554	2.166	1.407	1.713	0.998	0.94	0.972	0.935	0.965
DB01972	Guanosine-5'-Monophosphate	2.903	2.799	1.25	1.188	0.543	0.998	0.997	0.829	0.804	0.604
DB07475		2.91	1.604	1.807	1.131	-0.513	0.999	0.922	0.972	0.838	0.268
DB04896	Milnacipran	2.917	2.229	0.291	1.794	1.385	0.998	0.981	0.468	0.981	0.895
DB01114	Chlorphenamine	2.919	1.928	0.933	2.053	0.89	1	0.98	0.765	0.993	0.772
DB03092	5-Hydroxymethyl-Chonduritol	2.921	2.57	3.073	2.956	3.024	0.992	0.98	0.972	0.98	0.988
DB03439	4,6-dideoxy-4-amino-alpha-D-glucose	2.921	2.57	3.073	2.956	3.024	0.992	0.98	0.972	0.98	0.988
DB01221	Ketamine	2.923	1.675	0.441	2.469	1.553	0.998	0.953	0.585	0.998	0.94
DB02952	Alpha-Aminoisobutyric Acid	2.923	2.735	1.423	4.1	3.215	0.981	0.969	0.879	0.991	0.991
DB00478	Rimantadine	2.929	3.418	4.393	2.137	2.125	0.973	0.991	0.993	0.937	0.93
DB01239	Chlorprothixene	2.936	1.986	1.396	1.968	0.421	0.997	0.98	0.876	0.981	0.584
DB09035	Nivolumab	3.004	3.145	2.645	1.864	2.857	0.955	0.969	0.961	0.924	0.952
DB09037	Pembrolizumab	3.004	3.145	2.645	1.864	2.857	0.955	0.969	0.961	0.924	0.952
DB02341	Mdl 101,146	3.05	2.806	2.152	0.828	3.114	0.968	0.957	0.944	0.827	0.974
DB03925	Freselestat	3.05	2.806	2.152	0.828	3.114	0.968	0.957	0.944	0.827	0.974
DB02448	N-Tridecanoic Acid	3.067	3.05	2.695	4.832	3.43	0.972	0.976	0.97	0.995	0.987
DB01174	Phenobarbital	3.092	2.07	1.775	1.649	0.932	0.998	0.979	0.941	0.951	0.783

DB08100	2,6-dimethyl-4-[(E)-2-phenylethenyl]phenol	3.14	1.971	2.871	1.909	3.154	0.978	0.967	0.984	0.978	0.985
DB00733	Pralidoxime	3.158	2.429	2.525	2.537	2.989	0.975	0.949	0.958	0.963	0.968
DB00941	Hexafluronium	3.158	2.429	2.525	2.537	2.989	0.975	0.949	0.958	0.963	0.968
DB00944	Demecarium	3.158	2.429	2.525	2.537	2.989	0.975	0.949	0.958	0.963	0.968
DB00989	Rivastigmine	3.158	2.429	2.525	2.537	2.989	0.975	0.949	0.958	0.963	0.968
DB01010	Edrophonium	3.158	2.429	2.525	2.537	2.989	0.975	0.949	0.958	0.963	0.968
DB01057	Echothiophate	3.158	2.429	2.525	2.537	2.989	0.975	0.949	0.958	0.963	0.968
DB01122	Ambenonium	3.158	2.429	2.525	2.537	2.989	0.975	0.949	0.958	0.963	0.968
DB02845	Methylphosphinic Acid	3.158	2.429	2.525	2.537	2.989	0.975	0.949	0.958	0.963	0.968
DB04892	Phenserine	3.158	2.429	2.525	2.537	2.989	0.975	0.949	0.958	0.963	0.968
DB00193	Tramadol	3.179	2.857	-0.053	1.806	0.957	1	0.997	0.3	0.957	0.785
DB08964	Gemeprost	3.249	3.225	1.088	1.054	2.761	0.979	0.976	0.803	0.813	0.978
DB08342	S-PALMITOYL-L-CYSTEINE	3.255	2.498	1.806	1.188	0.981	0.998	0.994	0.944	0.756	0.767
DB06209	Prasugrel	3.26	3.252	3.498	3.802	2.803	0.993	0.993	0.981	0.985	0.966
DB01996	3-Methylpyridine	3.296	3.046	1.431	2.256	3.289	0.967	0.967	0.883	0.954	0.972
DB02697	Hydroxyaminovaline	3.296	3.046	1.431	2.256	3.289	0.967	0.967	0.883	0.954	0.972
DB03033	1-Methyloxy-4-Sulfone-Benzene	3.296	3.046	1.431	2.256	3.289	0.967	0.967	0.883	0.954	0.972
DB00758	Clopidogrel	3.365	4.022	3.224	1.423	1.466	0.975	0.987	0.983	0.912	0.94
DB01337	Pancuronium	3.418	3.179	1.715	1.069	0.475	0.991	0.992	0.914	0.767	0.615
DB09290	Ramosetron	3.447	3.548	3.052	4.08	2.334	0.996	0.997	0.984	0.995	0.944
DB08515	(3AR,6R,6AS)-6-((S)-((S)-CYCLOHEX-2-ENYL)(HYDROXY)METHYL)-6A-METHYL-4-OXO-HEXAHYDRO-2H-FURO[3,2-C]PYRROLE-6-CARBALDEHYDE	3.457	2.268	1.26	1.158	-0.864	1	0.985	0.75	0.841	0.175
DB01597	Cilastatin	3.468	3.329	2.645	2.58	3.502	0.98	0.981	0.961	0.946	0.979
DB06204	Tapentadol	3.473	3.275	-0.242	1.266	1.398	1	0.999	0.174	0.794	0.892
DB00674	Galantamine	3.48	3.298	3.057	2.59	1.88	1	1	0.999	0.999	0.972
DB02763	5-Mercapto-2-Nitro-Benzoic Acid	3.488	3.009	4.368	3.598	3.77	0.984	0.983	0.995	0.983	0.994
DB00209	Trospium	3.498	4.121	1.665	1.012	2.304	0.976	0.987	0.907	0.821	0.965
DB01289	Glisoxepide	3.545	3.548	2.934	4.884	3.671	1	1	0.971	1	1
DB00332	Ipratropium	3.553	3.084	1.519	0.718	1.917	0.98	0.971	0.877	0.704	0.96
DB00383	Oxyphencyclimine	3.553	3.084	1.519	0.718	1.917	0.98	0.971	0.877	0.704	0.96
DB00462	Methscopolamine bromide	3.553	3.084	1.519	0.718	1.917	0.98	0.971	0.877	0.704	0.96
DB00505	Tridihexethyl	3.553	3.084	1.519	0.718	1.917	0.98	0.971	0.877	0.704	0.96
DB00517	Anisotropine methylbromide	3.553	3.084	1.519	0.718	1.917	0.98	0.971	0.877	0.704	0.96
DB00771	Clidinium	3.553	3.084	1.519	0.718	1.917	0.98	0.971	0.877	0.704	0.96
DB08997	Dexetimide	3.553	3.084	1.519	0.718	1.917	0.98	0.971	0.877	0.704	0.96
DB09262	Imidafenacin	3.553	3.084	1.519	0.718	1.917	0.98	0.971	0.877	0.704	0.96
DB06594	Agomelatine	3.555	2.599	0.876	2.233	1.247	0.998	0.996	0.668	0.994	0.863
DB01273	Varenicline	3.574	3.666	1.66	1.905	2.304	0.977	0.984	0.912	0.953	0.956
DB09028	Cytisine	3.574	3.666	1.66	1.905	2.304	0.977	0.984	0.912	0.953	0.956
DB00139	Succinic acid	3.586	2.585	1.837	0.879	0.945	1	0.993	0.958	0.776	0.796
DB04843	Mepenzolate	3.616	3.73	1.823	1.132	2.14	0.98	0.981	0.925	0.849	0.965
DB01333	Cefradine	3.758	4.438	2.121	1.196	2.417	0.988	0.993	0.95	0.825	0.98
DB00980	Ramelteon	3.766	3.416	1.167	1.956	1.926	0.994	0.995	0.811	0.98	0.957
DB09071	Tasimelteon	3.766	3.416	1.167	1.956	1.926	0.994	0.995	0.811	0.98	0.957
DB01173	Orphenadrine	3.776	2.579	1.367	2.408	0.667	1	0.997	0.881	0.999	0.705
DB06738	Ketobemidone	3.801	3.125	-0.001	2.139	2.036	1	1	0.323	0.996	0.979
DB00157	NADH	3.809	1.631	-1.714	2.259	1.335	1	0.989	0.034	1	0.969
DB00964	Apraclonidine	3.833	3.327	4.784	0.141	3.172	0.986	0.979	0.997	0.489	0.982
DB00981	Physostigmine	3.84	3.391	2.544	1.263	2.232	0.983	0.982	0.975	0.869	0.967
DB06441	Cangrelor	3.84	3.834	3.82	3.165	2.769	0.992	0.998	0.99	0.972	0.966
DB04853	Binodenoson	3.862	3.858	1.796	0.686	3.551	0.989	0.992	0.926	0.788	0.984
DB00633	Dexmedetomidine	3.931	2.608	2.79	0.233	2.062	0.993	0.987	0.984	0.337	0.975
DB01170	Guanethidine	3.931	2.608	2.79	0.233	2.062	0.993	0.987	0.984	0.337	0.975
DB04688	Methylecgonine	3.958	4.015	2.884	1.38	4.067	0.984	0.979	0.976	0.909	0.986
DB01339	Vecuronium	3.966	4.51	4.525	3.082	2.541	0.99	0.996	0.993	0.98	0.95

DB07441	3-{[(9-CYANO-9,10-DIHYDRO-10-METHYLACRIDIN-9-YL)CARBONYL]AMINO}PROPANOIC ACID	4.011	3.568	2.841	1.19	3.735	0.983	0.983	0.976	0.841	0.987
DB07909	(1S,2S,5S)2-(4-GLUTARIDYLBENZYL)-5-PHENYL-1-CYCLOHEXANOL	4.011	3.568	2.841	1.19	3.735	0.983	0.983	0.976	0.841	0.987
DB08562	4-(4-STYRYL-PHENYLCARBAMOYL)-BUTYRIC ACID	4.011	3.568	2.841	1.19	3.735	0.983	0.983	0.976	0.841	0.987
DB00454	Meperidine	4.103	3.163	-0.025	2.107	1.563	1	0.999	0.362	0.991	0.943
DB01251	Gliquidone	4.139	3.961	3.428	5.294	3.879	1	1	0.984	1	1
DB01243	Chloroxine	4.175	4.068	1.99	3	2.732	0.984	0.987	0.939	0.993	0.975
DB01090	Pentolinium	4.209	4.106	2.667	5.419	4.169	1	0.997	0.964	1	0.999
DB00229	Cefotiam	4.304	4.183	1.522	1.007	3.805	0.987	0.99	0.881	0.783	0.987
DB00382	Tacrine	4.314	3.753	2.462	2.046	1.848	1	1	0.986	0.989	0.97
DB04835	Maraviroc	4.33	3.952	2.963	1.347	3.95	0.993	0.987	0.974	0.916	0.985
DB08409	4-NITRO-BENZYLPHOSPHONOBUTANOYL-GLYCINE	4.373	3.577	3.501	0.987	3.948	0.986	0.976	0.986	0.797	0.984
DB05316	Pimavanserin	4.389	3.493	2.423	1.028	3.547	0.986	0.979	0.97	0.819	0.981
DB00202	Succinylcholine	4.731	4.398	2.275	0.852	2.616	0.989	0.993	0.958	0.716	0.986
DB00747	Scopolamine	5.091	3.741	1.556	0.671	1.834	0.998	1	0.897	0.615	0.96
DB01226	Mivacurium	5.102	4.517	2.414	2.093	3.161	0.997	0.995	0.964	0.984	0.99
DB00657	Mecamylamine	5.259	4.947	2.412	2.818	4.114	0.99	0.997	0.976	0.993	0.995
DB01287		5.333	4.495	4.366	0.814	3.107	0.995	0.99	0.999	0.713	0.985
DB02365	1,10-Phenanthroline	5.36	5.22	1.571	2.095	5.617	0.994	0.999	0.908	0.976	0.998
DB01060	Amoxicillin	5.366	3.801	1.947	1.15	3.841	0.992	0.986	0.942	0.82	0.987
DB01465	2,5-Dimethoxyamphetamine	5.366	3.801	1.947	1.15	3.841	0.992	0.986	0.942	0.82	0.987
DB01516	3,4,5-Trimethoxyamphetamine	5.366	3.801	1.947	1.15	3.841	0.992	0.986	0.942	0.82	0.987
DB00513	Aminocaproic Acid	5.431	3.901	1.395	0.021	4.401	0.999	0.991	0.867	0.426	0.999
DB01038	Carphenazine	5.487	4.461	3.911	6.027	3.956	0.996	0.998	0.99	1	0.993
DB09097	Quinagolide	5.487	4.461	3.911	6.027	3.956	0.996	0.998	0.99	1	0.993
DB00501	Cimetidine	5.692	4.903	3.829	5.358	4.64	0.998	0.996	0.994	0.996	0.995
DB00411	Carbamoylcholine	5.803	4.534	1.554	1.343	2.357	1	1	0.899	0.888	0.994
DB01253	Ergometrine	5.941	4.377	4.193	1.479	2.442	1	1	1	0.944	0.995
DB04871	Lorcaserin	5.963	4.15	1.103	0.619	3.513	0.997	0.992	0.774	0.503	0.993
DB01484	4-Bromo-2,5-dimethoxyamphetamine	6.16	4.61	2.717	1.226	4.063	0.999	0.996	0.988	0.84	0.993
DB01278	Pramlintide	6.177	6.638	4.399	7.164	6.292	0.999	1	0.995	1	0.999
DB00670	Pirenzepine	6.361	5.554	0.286	1.581	2.103	0.998	0.999	0.494	0.941	0.981
DB01019	Bethanechol	6.431	5.239	1.097	1.275	3.205	0.999	0.998	0.768	0.879	0.996
DB06709	Methacholine	6.431	5.239	1.097	1.275	3.205	0.999	0.998	0.768	0.879	0.996
DB09300	Butylscopolamine	6.431	5.239	1.097	1.275	3.205	0.999	0.998	0.768	0.879	0.996
DB07375	5-BETA-ANDROSTANE-3,17-DIONE	6.528	4.709	2.933	1.372	5.102	0.997	0.997	0.991	0.906	0.999
DB00340	Metixene	6.861	4.956	0.635	1.959	3.009	1	1	0.609	0.983	0.999
DB00809	Tropicamide	7.011	6.229	2.095	1.909	1.75	0.999	1	0.956	0.976	0.951
DB00863	Ranitidine	7.058	6.462	4.764	3.759	5.871	0.998	0.998	0.998	1	0.999
DB00185	Cevimeline	7.204	4.947	1.198	1.552	3.376	1	0.996	0.81	0.929	0.997
DB00387	Procyclidine	7.204	4.947	1.198	1.552	3.376	1	0.996	0.81	0.929	0.997
DB00725	Homatropine methylbromide	7.204	4.947	1.198	1.552	3.376	1	0.996	0.81	0.929	0.997
DB00785	Cryptenamine	7.204	4.947	1.198	1.552	3.376	1	0.996	0.81	0.929	0.997
DB00986	Glycopyrronium	7.204	4.947	1.198	1.552	3.376	1	0.996	0.81	0.929	0.997
DB01085	Pilocarpine	7.204	4.947	1.198	1.552	3.376	1	0.996	0.81	0.929	0.997
DB01409	Tiotropium	7.204	4.947	1.198	1.552	3.376	1	0.996	0.81	0.929	0.997
DB02161	Hydroxy-Phenyl-Acetic Acid 8-Methyl-8-Aza-Bicyclo[3.2.1]Oct-3-Yl Ester	7.204	4.947	1.198	1.552	3.376	1	0.996	0.81	0.929	0.997
DB04365	Arecoline	7.204	4.947	1.198	1.552	3.376	1	0.996	0.81	0.929	0.997
DB06702	Fesoterodine	7.204	4.947	1.198	1.552	3.376	1	0.996	0.81	0.929	0.997
DB06787	Hexocyclium	7.204	4.947	1.198	1.552	3.376	1	0.996	0.81	0.929	0.997

DB08897	Aclidinium	7.204	4.947	1.198	1.552	3.376	1	0.996	0.81	0.929	0.997
DB09076	Umeclidinium	7.204	4.947	1.198	1.552	3.376	1	0.996	0.81	0.929	0.997
DB02586	4,7-Dimethyl-[1,10]Phenanthroline	7.213	5.534	2.642	2.315	6.087	1	0.999	0.98	0.99	1
DB03759	FG-9041	7.456	6.954	3.974	6.135	4.505	1	0.999	0.995	1	0.993
DB00810	Biperiden	7.63	5.774	1.383	1.86	3.818	1	0.999	0.856	0.967	1
DB01053	Benzylpenicillin	8.385	7.361	5.206	1.923	5.072	0.999	1	1	0.971	0.996
DB04599	Aniracetam	10.281	7.398	4.571	4.86	4.326	1	1	1	1	0.999
DB01140	Cefadroxil	11.503	8.844	4.305	3.805	6.491	1	1	0.998	0.998	0.998

Supplementary Table S6. Network-predicted drug combinations for all the drug pairs from the top 16 high-confidence repurposable drugs.

Drug A		Drug B		Separation	Number of targets overlapped
DrugBank ID	Name	DrugBank ID	Name		
DB00700	Eplerenone	DB07715	Emodin	0.88	0
DB00700	Eplerenone	DB01136	Carvedilol	0.84	0
DB00700	Eplerenone	DB00877	Sirolimus	0.76	0
DB00700	Eplerenone	DB00970	Dactinomycin	0.75	0
DB00700	Eplerenone	DB01103	Quinacrine	0.74	0
DB00700	Eplerenone	DB02187	Equilin	0.70	0
DB00700	Eplerenone	DB00715	Paroxetine	0.67	0
DB00700	Eplerenone	DB01065	Melatonin	0.65	0
DB00877	Sirolimus	DB00970	Dactinomycin	0.65	0
DB00700	Eplerenone	DB01033	Mercaptopurine	0.55	0
DB00244	Mesalazine	DB00700	Eplerenone	0.55	0
DB00700	Eplerenone	DB01394	Colchicine	0.54	0
DB00539	Toremifene	DB00700	Eplerenone	0.53	0
DB00877	Sirolimus	DB01065	Melatonin	0.51	0
DB00700	Eplerenone	DB01744	Camphor	0.50	0
DB00877	Sirolimus	DB01033	Mercaptopurine	0.50	0
DB01136	Carvedilol	DB07715	Emodin	0.49	0
DB00877	Sirolimus	DB01136	Carvedilol	0.43	1
DB00877	Sirolimus	DB01103	Quinacrine	0.42	0
DB00877	Sirolimus	DB06412	Oxymetholone	0.42	0
DB00877	Sirolimus	DB01394	Colchicine	0.41	2
DB00877	Sirolimus	DB07715	Emodin	0.40	0
DB01136	Carvedilol	DB06412	Oxymetholone	0.39	1
DB00715	Paroxetine	DB07715	Emodin	0.39	0
DB00877	Sirolimus	DB01744	Camphor	0.33	0
DB00877	Sirolimus	DB02187	Equilin	0.33	0
DB01103	Quinacrine	DB07715	Emodin	0.32	0
DB01065	Melatonin	DB07715	Emodin	0.31	1
DB01033	Mercaptopurine	DB07715	Emodin	0.28	0
DB00700	Eplerenone	DB06412	Oxymetholone	0.27	0
DB00877	Sirolimus	DB01029	Irbesartan	0.27	0
DB00970	Dactinomycin	DB01136	Carvedilol	0.25	0
DB01033	Mercaptopurine	DB01065	Melatonin	0.24	0
DB01136	Carvedilol	DB01744	Camphor	0.22	0
DB00715	Paroxetine	DB00877	Sirolimus	0.22	1
DB00715	Paroxetine	DB06412	Oxymetholone	0.22	0
DB00244	Mesalazine	DB00877	Sirolimus	0.21	0
DB01103	Quinacrine	DB06412	Oxymetholone	0.21	1
DB01136	Carvedilol	DB02187	Equilin	0.19	0
DB00244	Mesalazine	DB00970	Dactinomycin	0.19	0
DB00715	Paroxetine	DB00970	Dactinomycin	0.19	0

DB00970	Dactinomycin	DB01065	Melatonin	0.19	0
DB00244	Mesalazine	DB01065	Melatonin	0.18	1
DB00970	Dactinomycin	DB01394	Colchicine	0.18	0
DB00539	Toremifene	DB01136	Carvedilol	0.17	1
DB00700	Eplerenone	DB01029	Irbesartan	0.17	0
DB01033	Mercaptopurine	DB01136	Carvedilol	0.17	0
DB01065	Melatonin	DB01103	Quinacrine	0.15	0
DB00970	Dactinomycin	DB01033	Mercaptopurine	0.15	0
DB00244	Mesalazine	DB01136	Carvedilol	0.15	0
DB01065	Melatonin	DB06412	Oxymetholone	0.15	1
DB00715	Paroxetine	DB01033	Mercaptopurine	0.15	1
DB00244	Mesalazine	DB07715	Emodin	0.14	0
DB01394	Colchicine	DB07715	Emodin	0.13	0
DB01033	Mercaptopurine	DB01103	Quinacrine	0.13	2
DB00970	Dactinomycin	DB01103	Quinacrine	0.13	0
DB01065	Melatonin	DB01136	Carvedilol	0.13	1
DB00539	Toremifene	DB00877	Sirolimus	0.11	0
DB01065	Melatonin	DB01744	Camphor	0.10	1
DB01136	Carvedilol	DB01394	Colchicine	0.10	1
DB01394	Colchicine	DB06412	Oxymetholone	0.10	2
DB01103	Quinacrine	DB01394	Colchicine	0.09	1
DB00539	Toremifene	DB07715	Emodin	0.08	1
DB00715	Paroxetine	DB01065	Melatonin	0.08	0
DB00715	Paroxetine	DB01394	Colchicine	0.08	0
DB01103	Quinacrine	DB01744	Camphor	0.07	0
DB01033	Mercaptopurine	DB06412	Oxymetholone	0.07	2
DB01065	Melatonin	DB01394	Colchicine	0.07	0
DB00715	Paroxetine	DB01744	Camphor	0.05	0
DB00715	Paroxetine	DB02187	Equilin	0.05	0
DB00539	Toremifene	DB00970	Dactinomycin	0.04	0
DB00715	Paroxetine	DB01136	Carvedilol	0.04	9
DB01065	Melatonin	DB02187	Equilin	0.04	1
DB00244	Mesalazine	DB00715	Paroxetine	0.03	0
DB00539	Toremifene	DB02187	Equilin	0.03	2
DB00539	Toremifene	DB01065	Melatonin	0.02	1
DB06412	Oxymetholone	DB07715	Emodin	0.02	1
DB00539	Toremifene	DB00715	Paroxetine	0.02	0
DB00715	Paroxetine	DB01103	Quinacrine	0.01	10
DB01103	Quinacrine	DB01136	Carvedilol	0.01	7
DB00970	Dactinomycin	DB06412	Oxymetholone	0.01	0
DB01029	Irbesartan	DB01136	Carvedilol	0.00	0
DB00970	Dactinomycin	DB07715	Emodin	0.00	0
DB00970	Dactinomycin	DB01029	Irbesartan	0.00	0
DB01029	Irbesartan	DB07715	Emodin	0.00	0
DB01744	Camphor	DB02187	Equilin	0.00	1
DB01744	Camphor	DB07715	Emodin	0.00	1
